# The effectiveness of champions in implementing innovations in health care: a systematic review

**DOI:** 10.1186/s43058-022-00315-0

**Published:** 2022-07-22

**Authors:** Wilmer J. Santos, Ian D. Graham, Michelle Lalonde, Melissa Demery Varin, Janet E. Squires

**Affiliations:** 1 School of Nursing, Faculty of Health Sciences, University of Ottawa, Ottawa, ON Canada; 2 Clinical Epidemiology Program, Ottawa Hospital Research Institute, Ottawa, ON Canada; 3 School of Epidemiology and Public Health, School of Nursing, University of Ottawa, Ottawa, ON Canada; 4 Institut du Savoir Montfort, Hôpital Montfort, Ottawa, Canada

**Keywords:** Champions, Systematic review, Effectiveness, Implementation, Health care

## Abstract

**Background:**

Champions have been documented in the literature as an important strategy for implementation, yet their effectiveness has not been well synthesized in the health care literature. The aim of this systematic review was to determine whether champions, tested in isolation from other implementation strategies, are effective at improving innovation use or outcomes in health care.

**Methods:**

The JBI systematic review method guided this study. A peer-reviewed search strategy was applied to eight electronic databases to identify relevant articles. We included all published articles and unpublished theses and dissertations that used a quantitative study design to evaluate the effectiveness of champions in implementing innovations within health care settings. Two researchers independently completed study selection, data extraction, and quality appraisal. We used content analysis and vote counting to synthesize our data.

**Results:**

After screening 7566 records titles and abstracts and 2090 full text articles, we included 35 studies in our review. Most of the studies (71.4%) operationalized the champion strategy by the presence or absence of a champion. In a subset of seven studies, five studies found associations between exposure to champions and increased use of best practices, programs, or technological innovations at an organizational level. In other subsets, the evidence pertaining to use of champions and innovation use by patients or providers, or at improving outcomes was either mixed or scarce.

**Conclusions:**

We identified a small body of literature reporting an association between use of champions and increased instrumental use of innovations by organizations. However, more research is needed to determine causal relationship between champions and innovation use and outcomes. Even though there are no reported adverse effects in using champions, opportunity costs may be associated with their use. Until more evidence becomes available about the effectiveness of champions at increasing innovation use and outcomes, the decision to deploy champions should consider the needs and resources of the organization and include an evaluation plan. To further our understanding of champions’ effectiveness, future studies should (1) use experimental study designs in conjunction with process evaluations, (2) describe champions and their activities and (3) rigorously evaluate the effectiveness of champions’ activities.

**Registration:**

Open Science Framework (https://osf.io/ba3d2). Registered on November 15, 2020.

**Supplementary Information:**

The online version contains supplementary material available at 10.1186/s43058-022-00315-0.

Contributions to the literature

- We identified 35 studies. We found sufficient studies (defined as ≥ four studies) to make conclusions regarding deployment of champions and (1) innovation use by providers; (2) system/facility instrumental innovation use and (3) patient outcomes. In a subset of seven studies, five studies reported an association between use of champions and increased uptake of innovations by health systems/facilities. The evidence is either mixed or unexamined pertaining to deployment of champions and innovation use by patients or providers, or at improving outcomes
- We found four additional scales previously not cited in reviews about champions. Despite this, there is limited description of champions or their activities in the included studies, and we did not find a complete measure that is reflective of the champion construct.
- Our results reinforce the need for experimental studies conducted in conjunction with process evaluations that describes and evaluates the champions and their activities using valid and reliable measures.

## Introduction

Evidence-based practice (EBP) refers to the development and provision of health services according to best research evidence, health care providers’ expertise and patients’ values and preferences [1]. Adoption of EBP by organizations can create safer practices, better patient outcomes and decrease health care costs [2]. Best practice and technology can be defined as an innovation [3, 4]. However, some authors reported that health services and practices are not always based on best evidence [5–9]. Braithwaite and colleagues summarized that 60% of health services in the USA, England and Australia follow best practice guidelines; about 30% of health services are of low value; and 10% of patients globally experience iatrogenic harm [10].

To implement innovations, research evidence must be synthesized, adapted and applied in a specific health care context, and this adoption must be evaluated [11]. The adoption of innovations is improved when devoted individuals, often referred to as champions, facilitate implementation [3, 12, 13]. Champions are individuals (health care providers, management [14, 15], or lay persons [16, 17]) who volunteer or are appointed to enthusiastically promote and facilitate implementation of an innovation [13, 18, 19]. There is confusion and overlap between the concept of champion and other concepts, such as opinion leaders, facilitators, linking agents, change agents [19, 20], coaches and knowledge brokers [19]. Some studies have attempted to clarify these different roles that are intended to facilitate implementation [19, 20]. Despite this, these terms are sometimes used synonymously, while at other times treated as different concepts [19, 21]. Hence, we sought to only examine champions in this study.

There are at least four recent published reviews that reported on the effectiveness of champions [21–24]. In 2016, Shea and Belden [24] performed a scoping review (*n* = 42) to collate the characteristics and impacts of health information technology champions. They reported that in a subset of studies (24 qualitative and three quantitative), 23 of the 27 studies reported that champions had a positive impact during the implementation of health information technology [24]. In 2018, Miech and colleagues [21] conducted an integrative review (*n* = 199) of the literature on champions in health care. They reported a subset of 11 quantitative studies (four studies that randomly allocated the presence and absence of champions and seven studies that reported an odds ratio) that evaluated the effectiveness of champions [21]. They reported that despite some mixed findings in the subset of studies, use of champions was reported to generally influence adoption of innovations [21]. In 2020, Wood and colleagues [23] conducted a systematic review (*n* = 13) on the role and efficacy of clinical champions in facilitating implementation of EBPs in settings providing drug and alcohol addiction and mental health services. They reported that champions influenced health care providers use of best practices or evidence-based resources in four qualitative studies [23]. In 2021, Hall and colleagues [22] performed a systematic review and metanalysis of randomized controlled trials (RCT; *n* = 12) that evaluated the effectiveness of champions, as a part of a multicomponent intervention, at improving guideline adherence in long-term care homes. They concluded from three RCTs that there is low certainty evidence suggesting that the use of champions may improve staff adherence to guidelines in long-term care settings [22].

According to Tufanaru and colleagues [25], synthesizing the effectiveness of an intervention requires the summary of quantitative studies using a systematic process. As described above, two of the previous reviews discussing champions’ effectiveness were primarily composed of qualitative studies [23, 24]. Synthesizing qualitative studies may highlight relationships that exist between champions and aspects of implementation, but does not inform champions’ effectiveness based on the definition outlined by Tufanaru and colleagues [25]. Furthermore, some of the previous reviews examining champions’ effectiveness were limited to the following: (1) types of innovations (i.e. health information technology [24]); (2) setting (i.e. long-term care settings [22] or health care settings providing mental health and addiction treatment [23]); or study design/effect size (i.e. only including experimental design studies [21, 22] or studies reporting odd ratios [21]). Moreover, as some of the previous reviews sought to examine other aspects pertaining to champions in addition to champions’ effectiveness, they utilized study designs (i.e. integrative review [21], scoping review [24]) that did not require the performance of some conventional steps for systematic reviews as outlined by the JBI manual [25] and the Cochrane handbook [26]. For example, grey literature was not included, or the methodological quality of included studies was not appraised in the two cited reviews [21, 24].

To build on the four reviews describing champions’ effectiveness [21–24], we conducted a systematic review to determine whether the use of champions, tested in isolation from other implementation strategies, are effective at increasing the use of innovations across health care settings and innovation types. Our review is rooted in a post-positivist paradigm [27] because it focused on the relationships between measurable components of champions and implementation and emphasized the rigour attributed to study design (e.g. experimental studies are more rigorous than quasi-experimental and observational studies). The research questions were as follows: (1) How are champions described and operationalized in the articles that evaluates their effectiveness? (2) What are the effects of champions on the uptake of innovations (knowledge use) by patients, providers and systems/facilities? (3) What are the effects of champions on patient, provider and system/facility outcomes?

## Methods

The research team followed the JBI approach to conducting systematic review of effectiveness [25] and used the Preferred Reporting Items for Systematic Reviews and Meta-Analyses (PRISMA) [28] and the Synthesis without meta-analysis (SWiM) in systematic reviews reporting guidelines [29]. The research team registered the review in Open Science Framework as part of a broader scoping review [30]. See Additional files 1 and 2 for the PRISMA and the SWiM checklists respectively.

### Search strategy and study selection

#### Search strategy

WJS devised a search strategy with a health sciences librarian for a larger scoping review that aimed to describe champions in health care. A second health science librarian assessed the search strategy using the Peer Review of the Electronic Search Strategy (PRESS) checklist [31]. The search strategy (outlined in Additional file 3) consisted of Boolean phrases and medical subject headings (MESH) terms for the following concepts and their related terms: champions, implementation and health care/health care context. Eight electronic databases (Business Source Complete, CINAHL, EMBASE, Medline, Nursing and Allied Health, PsycINFO, ProQuest Thesis and Dissertations, and Scopus) were searched from inception to October 26, 2020, to identify relevant articles. Further, WJS identified and assessed additional records retrieved from the reference lists of included studies and synthesis studies that were captured by the search strategy and from forward citation searches of included studies.

### Eligibility criteria

#### Inclusion

We included all published studies and unpublished theses and dissertations that used a quantitative study design to evaluate the effectiveness of individuals explicitly referred to as champions at either increasing the use of innovation or improving patient, provider, or system/facility outcomes within a health care setting. English language articles were included regardless of date of publication or type of quantitative study design.

#### Exclusion

Synthesis studies, qualitative studies, study protocols, conference abstracts, editorials and opinion papers, case studies, studies not published in English, articles without a full text available, and articles that are not about knowledge translation or EBP were excluded.

#### Study selection

We used Covidence [32] to deduplicate records; WJS and MDV independently assessed the title and abstract of these deduplicated records. Records were included if the title and abstract mentioned champions within health care. All potentially relevant articles and articles that had insufficient information were included for full text screening. WJS and MDV independently assessed the inclusion of full text articles in accordance with the eligibility criteria detailed above. Discrepancies were resolved through consensus or if necessary, through consultation of a third senior research team member (ML, IDG, JES). WJS and MDV piloted the eligibility criteria on 100 records and 50 full text articles.

### Data extraction

WJS and MDV extracted data in duplicate using a standardized and piloted extraction form created by the research team in DistillerSR [33]. The following data were extracted: (1) study characteristics: first author, year of publication, study design, country, setting, details on the innovation being implemented, study limitations, funding, and conflict of interest; (2) study participant demographics: sample size, age, sex and gender identity, and professional role; (3) champion demographics: number of champions, age, sex and gender identity, and professional role; (4) operationalization of champions: quantitative measures relative to the presence or influence of champions and the reliability and validity of these measures; and (5) study outcome: the dependent variable evaluated with use of champions, method of measurement of dependent variable, reliability and validity of measure(s), statistical analysis/approach undertaken, and statistical results and significance of results at *p*-value of 0.05 or less. WJS and MDV resolved discrepancies through discussion or through consultation of a senior research team member. WJS contacted authors for missing data integral to the analysis (e.g. to clarify statistical test results when integers are not reported in a figure in an article).

### Quality appraisal

WJS and MDV independently appraised study methodological quality using five JBI critical appraisal tools for (1) case–control studies [34], (2) cohort studies [34], (3) cross-sectional studies [34], (4) quasi-experimental (non-randomized experimental) studies [25] and (5) randomized control trials [25]. Non-controlled before and after studies and interrupted time series were assessed using the critical appraisal tool for quasi-experimental studies [25]. Discrepancies were resolved through consensus. Each question response was attributed a score according to a scoring system from a recently published JBI systematic review [35] (Yes = 2; Unclear = 1; and No = 0). A quality score between 0 and 1 was calculated for each included study by dividing the total score with the total number of available scores. According to this quality score, the research team classified each study as either weak (quality score < 0.5), moderate (quality score between 0.5 and 0.74), or strong (quality score between 0.75 and 1) [36]. Studies were included in the data synthesis regardless of the quality score. We also examined the total percentage of “Yes” responses for each critical appraisal question to determine the areas of variability in quality between studies with the same study design.

### Data synthesis

Through visually examining the data in tables, we found methodological and topic heterogeneity amongst the included studies (apparent from the varying types of innovations and study outcomes), which warranted the need for a narrative synthesis of the data. WJS used both inductive and deductive content analysis [37] to aggregate study outcomes into categories as detailed below. Two senior research team members (IDG and JES) evaluated and confirmed the accuracy of the performed categorization. WJS deductively categorized each extracted study outcome as either innovation use or as outcomes as described by Straus and colleagues [38]. We specifically defined innovation use in this study as comprising (1) conceptual innovation use: an improvement in knowledge (enlightenment) or attitude towards an innovation (best practices, research use, or technology) (often referred to as conceptual knowledge use [38]); and (2) the use or adoption of an innovation (instrumental knowledge use [38]). WJS further categorized study outcomes as either patient, provider and system/facility outcomes. Examples of patient outcomes included changes in patient’s health status and quality of life. Provider outcomes included provider satisfaction with practice. System/facility outcomes included system-level indicators such as readmission rates, length of stays and access to training [38]. Differing from Straus and colleagues [38], we also stratified innovation use into patient, provider and system/facility innovation use according to the level of analysis and intended target for change in the study. Patient and provider innovation use was defined as uptake of an innovation by patients and providers [38]. System/facility innovation use was defined as the adoption of an innovation throughout a whole system or facility; this included, for example, adoption of programs which entailed changes in work culture, policies and workflows [39–41]. Moreover, WJS used inductive content analysis to further categorize study outcomes within their respective category of innovation use or outcome according to the type of practice or technology being implemented. For example, the implementation of transfer boards, hip protectors and technology were grouped together, as these innovations pertain to the introduction of new equipment in clinical practice. Study outcomes that could not be coded according to the above classifications were grouped into an “other outcomes” category (e.g. whether formal evaluations were more likely to be conducted).

To answer research question 1, we inductively organized the measures used to identify exposure to champions into three categories: (1) studies that used a single dichotomous (“Yes or No”) or Likert scale, (2) studies that appointed a champion for their study and (3) studies that used more nuanced measures for champion exposure. To answer research questions 2 and 3, we used a predetermined set of vote-counting rules used in published systematic reviews [42–44] as outlined on Table 1. As previously suggested by Grimshaw and colleagues [45], we reported the study design, sample sizes, significant positive, significant negative and non-significant relationships, and the magnitude of effect (if reported by the study) for all the studies. We also performed a sensitivity analysis to determine whether the number of categories for which a conclusion can be made, or the conclusion for any category would change when weak studies are removed from the analysis [43, 46]. Lastly, we conducted a sub-group analysis of the data to evaluate whether our conclusions would change, or if there are differences in conclusions, between studies that used a dichotomous (presence/absence) measure and studies that appointed champions or used more nuanced measures of the champion strategy.

**Table 1:** Vote-counting rules

(1)To make conclusions pertaining to champions’ effectiveness at increasing innovation use or outcomes (patient, provider, or system/facility) four or more studies must have evaluated a relationship or correlation between champions and innovation use or the outcome of innovation use
(2)Champions’ effectiveness at increasing innovation use or outcomes of innovation use were coded as follows: a.Champions are effective if 60% or more of the studies demonstrated a positive significant relationship between exposure to champions and either innovation use or outcome of innovation use b.Champions are ineffective if 60% or more of the studies demonstrated a non-significant or significant negative relationship between exposure to champions and either innovation use or outcome of innovation use c.Champions’ effectiveness is mixed if less than 60% of the studies reported a non-significant/significant relationship between exposure champions and either innovation use or outcome of innovation use
(3)We applied the same rules as above (rule number 2) to determine whether individual studies demonstrated a significant, non-significant, or mixed relationship between exposure to champions and either innovation use or outcome of innovation use. The analysis was based on percentage of statistical results reported in a study. We performed these evaluations to counteract double counting articles with multiple study outcomes
(4)When both bivariate and multivariate statistics are reported in a study, we used the more robust multivariate findings in our synthesis
(5)We assessed categories examined in three or less studies to determine trends in champion effectiveness using the same rules detailed above

## Results

### Search results

**Fig. 1:**
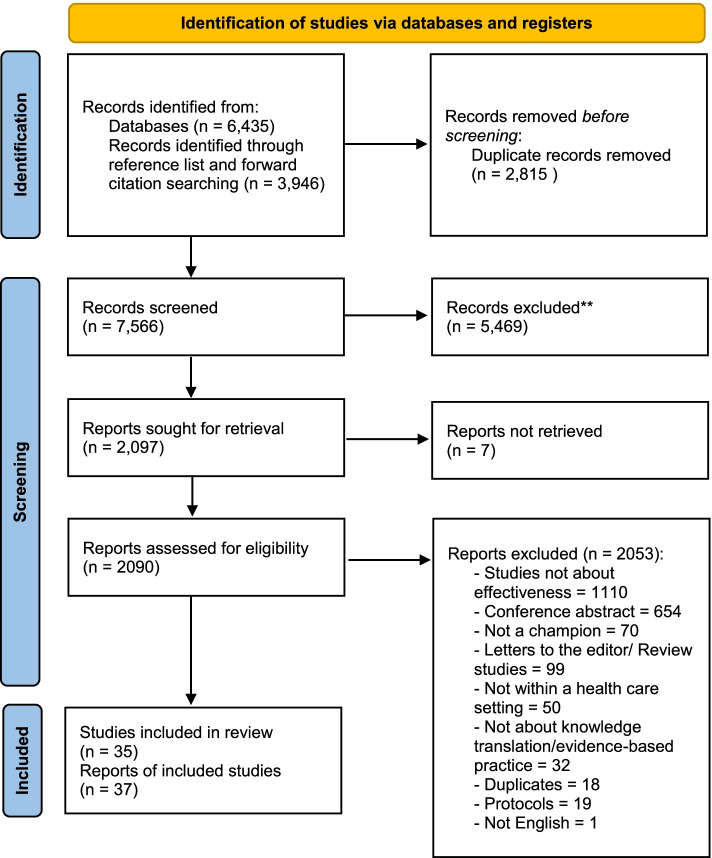
PRISMA 2020 flow diagram

As demonstrated in the flowchart (Fig. 1), the database search identified 6435 records and the additional citation analysis identified 3946 records. Duplicates (*n* = 2815) were removed using Covidence [32], leaving 7566 articles to screen. After titles and abstracts were screened, 2097 articles were identified to potentially met eligibility criteria. The full text of these 2090 articles was reviewed (seven articles could not be retrieved), with 35 studies (37 articles) [39–41, 47–80] meeting all the inclusion criteria (Additional file 4 lists excluded full text articles and reasons for exclusion).

### Characteristics of included studies

The included studies in our systematic review were primarily conducted in the last 10 years (2010–2020), with the highest proportion of studies conducted in North America (*n* = 28) and in acute care/tertiary settings (*n* = 20). The number of health care settings per article ranged from one to 1174 settings and sample sizes ranged from 80 to 6648 study participants. Table 2 summarizes study characteristics, and Table 3 provides more detailed descriptions of each study.

**Table 2:** Summary of included studies (*n* = 35)

Characteristic		*N* (%) of studies or frequency (%)^b^
Publication year	2010–2020	24 (68.6%)
	2000–2010	10 (28.6%)
	1997	1 (2.9%)
Country	United States of America	22 (62.9%)
	Canada	5 (14.3%)
	England	1 (2.9%)
	India	1 (2.9%)
	Israel	1 (2.9%)
	Italy	1 (2.9%)
	Taiwan	1 (2.9%)
	Uganda	1 (2.9%)
	United States of America and Puerto Rico	1 (2.9%)
	“18 European countries”	1 (2.9%)
Setting^a^	Acute care/tertiary	20 (57.1%)
	Primary	11 (31.4%)
	Community/Home	4 (11.4%)
	Long-term Care	2 (5.7%)
Number of settings/institutions	One setting/institution	3 (8.6%)
	2–10 settings/institutions	2 (5.7%)
	11–50 settings/institutions	11 (31.4%)
	51–100 settings/institutions	3 (8.6%)
	101–500 settings/institutions	10 (28.6%)
	> 500 settings/institutions	2 (5.7%)
	Not reported	4 (11.4%)
Study design	Cross-sectional	23 (65.7%)
	Interrupted time series	3 (8.6%)
	Non-controlled before and after	3 (8.6%)
	Cohort	2 (5.7%)
	Mixed methods (qualitative interview and cross-sectional)	1 (2.9%)
	Case control	1 (2.9%)
	Cluster randomised trial	1 (2.9%)
	Mixed methods (qualitative interview and cohort)	1 (2.9%)
Study participants^a^	Health care providers	17 (48.6%)
	Patients	8 (22.9%)
	Managers or administrators	5 (14.3%)
	Not reported	7 (20%)
Sample size	1–100	2 (5.6%)
	101–500	13 (36.1%)
	501–1000	7 (19.4%)
	> 1000	6 (16.7%)
	Not reported	8 (22.2%)
Sex and gender of study participants	**Reported sex** (*n* = # studies)	3 (8.3%)
	Female	5052/8305 (60.8%)^b^
	Male	3253/8305 (39.2%)^b^
	Sex category interpreted as sex by extractor	3/3 (100%)
	**Reported gender identity** (*n* = # studies)	10 (27.8%)
	Female	5228/7026 (74.4%)^b^
	Male	1771/7026 (25.2%)^b^
	Non-binary	1/7026 (0.0%)^b^
	Missing/Not reported	26/7026 (0.4%)^b^
	Gender identity interpreted as sex by extractor	9/10 (90%)
	Gender identity interpreted as gender by extractor	1/10 (10%)
	**Study did not specify if reported category was sex or gender identity** (*n* = #studies)	2 (5.26%)
	Male	3992/7047 (56.6%)
	Female	3055/7047 (43.3%)
	Interpreted as sex	2/2 (100%)
	**No report of sex or gender** (*n* = #studies)	21 (58.3%)

^a^Some studies are counted in more than one setting and study participants category; therefore, numbers do not add to *n* = 35 (100%);

^b^Data refers to frequency (%) of study participants rather than number (%) of studies.

**Table 3:** Description of included articles

First author, year	Country	Setting	Design	Study participants (age, sex and gender and professions if applicable)	Innovation, Implementation Outcome Measurement (Measure Reliability and Validity)
Albert, 2012 [47]	USA	Clinic(s) (number not reported)	Cross-sectional study	Physicians who reported consistent use of standard order programs = 502 Age: Mean (SD) = 50.4 (10.1) years Sex and Gender: Not reported Physicians who consistently use SOPs for influenza vaccine only = 175 Age: Mean (SD) = 50.2 (9.4) years Sex and Gender: Not reported Physicians who consistently use SOPs for influenza and pneumococcal polysaccharide vaccine = 203 Age: Mean (SD) = 51.8 (9.9) years Sex and Gender: Not reported	Innovation: Standard order programs are facility policies allowing non-physician health care providers to assess patient’s immunization status and administer vaccines without a physician order **Study outcome measurement** Measure: Single item asking how often non- physician staff utilize a standard order program for administering influenza, pneumococcal polysaccharide vaccine, or both types of vaccines at their clinic. Options range from: a) inexistence or lack of interest in implementing standard order programs; b) inexistence but interest in implementing standard order programs; c) existence but inconsistent use of standard order programs; or d) consistent use of standard order programs Reliability: Not reported; Validity: Not reported
Alidina, 2018 [48]	USA	Hospital(s) (number not reported)	Cross-sectional study	Operating room staff = 368 Age: Not reported Sex & Gender: Not reported Professions: Anesthesiology = 311 (84.5%); Surgery = 13 (3.5%); operating room staff = 24 (6.5%); Other = 20 (5.4%)	Innovation: Operating room cognitive aids are tools (e.g. checklist or emergency operating procedures) that provide information to facilitate and standardize decision making, action and information sharing between health care providers during crises **Study outcome measurement** Measure: Single survey item asking operating room staff about the regular use of operating room cognitive aids at their facility on a 5-point Likert scale from “strongly disagree to strongly agree” Reliability: Not reported Validity: The survey was piloted survey with 21 operating room staff to assess readability and comprehensibility of questions
Anand, 2017 [49]	18 European countries	203 neonatal intensive care units	Prospective cohort study	Neonatal intensive care patients = 6648 Age: Mean (SD) = 35.0 (4.6) weeks Not specified Sex or Gender: Male = 3753 (56.5%); Female = 2895 (44.5%) Interpreted as: Sex	Innovation: The use of measurement scales that measure continuous pain proceeding invasive procedures may enhance the quality pain management in neonatal patients (e.g. prevents untreated pain, under or overdosing of analgesics, or the development of drug tolerance) **Study outcome measurement** Measure: Chart audit to measure the use of pain assessments tools/scales designed to measure continuous pain (e.g. Echelle Douleur Inconfort Nouveau-ne (EDIN) scale, COMFORT scale) for 1 month in participating NICUs Reliability: A random 10% of the data was double checked by a local data quality manager. If 1% or more errors is present, then another random 10% would be double checked. If 1% or more errors continued, then all data entries for that NICU would be double checked Validity: Not reported
Ash, 1997 [50]	USA	65 academic health sciences centres	Cross-sectional study	Informatics professionals and library workers = 534^a^ Age: Not reported Sex and Gender: Not reported Professions: Informatics professionals = 195 (31% of 629); library workers = 339 (48% of 706) ^a^	Innovation: Electronic mail is a communication method whereby an individual sends a message to another individual via a computer or other technological devices **Study outcome measurement** Measures: Two single items scales measuring electronic mail infusion [81] and diffusion [82] on a 4-point scale (low to high). Infusion is the extent of which an innovation is implemented, while diffusion is the breadth of implementation within an organization Reliability: Not reported; Validity: Not reported
Ben-David, 2019 [51]	Israel	24 medical surgical intensive care units	Cross-sectional study	Sample information not reported	Innovation: Central-line-associated bloodstream infection prevention practice bundles include measures that decreases risk of infection during insertion (e.g. hand hygiene and use of maximal sterile barriers) and measures that minimize infection risk during ongoing catheter use (e.g. aseptic technique for tubing and dressing changes and the prompt removal of central line catheters when no longer necessary) **Study outcome measurement** Measure: Monthly incidence rates of central-line-associated bloodstream infection collected as part of routine national surveillance in Israel hospitals Reliability: Not reported; Validity: Not reported
Bentz, 2007 [52]	USA	19 (10 intervention, 9 control) clinics	Cluster randomised trial	(1) Control clinic patients = Not reported Age: Mean (SD) = 50.7 (5.6) years Reported Gender: Male = 33.5%; Female = 76.5% Interpreted as: Sex (2) Physicians in control clinics = 55 Age: Not reported Reported Gender: Male = 49.2%; Female = 50.8% Interpreted as: Sex 3) Intervention clinic patients = Not reported Age: Mean (SD) = 54.2 (6.7) years Reported Gender: Male = 34%; Female = 76% Interpreted as: Sex 4)Physicians in intervention clinics = 57 Age: Not reported Reported Gender: Male = 51.6%; Female = 48.4% Interpreted as: Sex	Innovation: The delivery of electronic health record generated feedback, rather than peer feedback to health care providers to increase the delivery of tobacco cessation assistance and referrals to the Oregon Tobacco Quitline **Study outcome measurement** Measure: Monthly rates of clients referred, reached, or counseled regarding tobacco cessation using the Oregon Tobacco Quitline according to electronic health records Reliability: Not reported; Validity: Not reported
Bradley, 2012 [53]	USA	533 hospitals	Cross-sectional study	Hospitals’ chief executive officers = 533 Age: Not reported Sex & Gender: Not reported Professions: Not reported	Innovation: There was no specific innovation in this study. The purpose of this study was to identify and determine the relationships between hospital strategies and hospital risk-standardized mortality rates **Study outcome measurement** Measure: Thirty-day risk-standardized mortality rates: “predicted number of deaths within 30 days of admission at a hospital divided by the expected number of deaths within 30 days of admission at the same hospital multiplied by the overall 30-day mortality rate of the cohort” [53] (p.3) Reliability: Not reported; Validity: Not reported
Campbell, 2008 [54]	USA	One hospital	Non-controlled before and after study	Intensive care unit patients = 120 Age: Range = 32–93 years old Reported Gender: Male = 53%; Female = 47% Interpreted as: Sex	Innovation: The Keystone ICU Sepsis project aims at improving the quality of care, decreasing length of stay, eliminating unnecessary cost and creating a culture centred on safety in participating Michigan hospital’s intensive care units. The Keystone ICU Sepsis project seeks to increase the identification of patients with or at risk of sepsis and the implementation of appropriate of sepsis protocols **Study outcome measurement** Measures: Chart documentation of (1) intensive care unit nurses’ compliance with sepsis-screening protocols and (2) the proportion of patients with severe sepsis that physicians initiated the sepsis protocol on Reliability: Not reported; Validity: Not reported
Chang, 2012 [40]	USA	225 primary care practices	Cross-sectional study	Primary care directors: sample details not reported	Innovation: Depression care improvement models are evidence-based models that guides screening and management of common mental health disorders in a primary care setting. These models include the collocation of mental health specialists, the Translating Initiatives in Depression (TIDES) model and the Behavioural Health Laboratory (BHL) model **Study outcome measurement** Measure: Primary care directors’ responses to a single item in the 2007 VA Clinical Practice Organization Survey (CPOS) Primary Care [83]. This single item asks the degree of implementation of three depression care improvement models (collocation, TIDES and BHL). The authors dichotomized the responses into fully or partially implemented versus planned but not yet implemented or not implemented. Some clinics may have implemented multiple depression improvement models. The authors used a hierarchal coding system to assign each clinic to only a single model; prioritizing BHL, then TIDES, then collocation Reliability: Not reported; Validity: Not reported
Ellerbeck, 2006 [55]	USA	44 hospitals	Cross-sectional study	Sample details not reported	Innovation: Consistent use of aspirin and beta-blockers during the hospitalization or at the time of discharge in patients with acute myocardial infarction **Study outcome measurement** Measures: Audit of hospital records and supplemental Medicare billing records of a random sample of Medicare patients admitted between April 1, 1998, and May 31, 2001, with a principal diagnosis of acute myocardial infarction. Outcome data was the use of aspirin and beta-blockers at admission and at discharge Reliability: Not reported; Validity: Not reported
Foster, 2017 [56]	USA and Puerto Rico	1174 hospitals	Non-controlled before and after study	Sample details not reported	Innovation: Innovations were not clearly outlined in this paper. The purpose of the paper is to assess the relationships between engagement or knowledge translation strategies and the change in a composite measure of quality of care according to 10 harm topics (e.g. readmissions). Examples of these engagement or knowledge translation strategies includes improvement events targeted to staff, and improvement fellows (a subset of which comprises of champions) **Study outcome measurement** Measure: A weighted composite score of quality of care calculated by adding a ratio of occurrence of the 10 harm topics for 1 month. A low score means higher quality. These measures are based on self-reports submitted by hospitals Reliability: Not reported; Validity: Not reported
Goff, 2019 [57]	USA	80 pediatric primary care practices	Cross-sectional study	Practice leaders = 80 Age in years (*n* (%)): 26–35 = 8 (10%); 36–45 = 17 (21.3%); 46–55 = 17 (21.3%); 56–65 = 31 (38.8%); > 65 = 3 (3.75%); No response = 4 (5%) Reported Gender: Female = 66 (82.5%); Male = 10 (12.5%); Non-binary = 1 (1.25%); No response = 3 (3.75%) Interpreted as: Gender Professions: Practice manager = 58 (72.5%); Nurse manager = 6 (7.5%); Physician owner = 1 (1.25%); Physician leader = 4 (5%); Other = 9 (11.3%); No response = 2 (2.5%)	Innovation: This study did not have an innovation, rather the study assessed the relationships between the organizational characteristics of primary care practices in the Massachusetts Health Quality Partners and their reported clinical quality and patient experience scores **Study outcome measurement** Measures: The authors translated clinical quality and patient experience scores from Massachusetts Health Quality Partners website to a scale from zero to three points. Average patient experience scores and clinical quality scores were calculated for practices reporting four or more patient experience or clinical quality scores Reliability: Not reported; Validity: Not reported
Granade, 2020 [58]	USA	Primary care clinics and pharmacies (number not reported)	Cross-sectional study	(1) Clinicians = 4911 Age in years (*n* (%)): < 40 = 1497 (30.5%); 40–49 = 1503 (26.8%); 50–59 = 1156 (23.4%); ≥ 60 = 736 (19.3%) Reported Sex: Male = 1858 (48.5%); Female = 3053 (51.5%) Interpreted as: Sex Professions: Physician = 2349 (71.5%); Nurse practitioner = 1293 (15.7%); Physician assistant = 1269 (12.8%) (2) Pharmacists = 793 Age in years (*n* (%)): < 40 = 310 (45.3%); 40–49 = 194 (19.4%); 50–59 = 161 (17.5%); ≥ 60 = 125 (17.7%) Reported Sex: Male = 1858 (48.5%); Female = 3053 (51.5%) Interpreted as: Sex	Innovation: The Standards for Adult Immunization Practice emphasizes that health care providers should routinely perform assessments, strongly recommend, administer, or provide referrals, and document in electronic health care systems the administration of all necessary vaccines in adult patients **Study outcome measurement** Measure: A survey developed by Centers for Disease Control and Prevention and Abt Associates Inc. to assess primary care clinicians and pharmacists’ self reported adherence to the Standards for Adult Immunization Practice and factors (e.g. presence of champions) related to implementation of these standards. A composite score of vaccination process standard adherence was calculated by the authors Reliability: Not reported Validity: Survey question phrasing were revised for better readability and comprehension
Hsia, 2019 [59]	Taiwan	119 hospitals	Cross-sectional study	Top managers = 119 Age: Not reported Sex and Gender: Not reported Professions: Not reported	Innovation: E-Health innovations are forms of information technology that are designed to aid with the delivery of health care related activities. Examples of E-Health innovations are electronic health record computerized provider order entry, and picture archiving and communication systems **Study outcome measurement** Measure: A seven-item subscale within a 28-item questionnaire that is intended to measure the extent that hospital medical services and work processes are performed using E-Health technologies. The questionnaire was created by the authors. Scoring of items were on a five-point Likert scale ranging from strongly disagree to strongly agree Reliability: Composite reliability = 0.95; *α* = 0.934 Validity: Factor loadings range = 0.728–1.053, which is above the 0.707 threshold
Hung, 2008 [60]	USA	57 primary care practice-based research networks	Cross-sectional study	Patients = 4735 Age in years (*n* (%)): 18–39 = 1348 (28.9%); 40–54 = 1476 (31.6%); 55–64 = 925 (19.8%); ≥ 65 = 921 (19.7%) Reported Gender: Male = 1319 (27.9%); Female = 3377 (71.3%) Interpreted as: Sex	Innovation: The Chronic Care Model is a system-level framework consisting of six main areas with a focus on prevention and health behaviour counselling in primary care practices. These six main areas include (1) establishing a health system and organization of care centred on chronic care, (2) supporting patient participation in their own care, (3) a proactive delivery system that identifies and addresses health needs, (4) availability of evidence-based decision supports for health care providers, (5) implementing an electronic health care information system and (6) established networks with community resources to support continuity of care. This study was interested on understanding how the Chronic Care Model related to quality-of-life measures **Study outcome measurement** Measures: Three survey items based on the Center for Disease Control and Prevention’s Healthy Days core measures [84–86]: (1) number of unhealthy days in the past 30 days, (2) number limiting days in the past 30 days, (3) general health status. Number of unhealthy days and limiting days was measured on a three-point ordinal scale (0 days, 1–13 days and 14–30 days). General health status is rated on a five-point scale from poor to excellent Reliability: Not reported; Validity: Not reported
Kabukye, 2020 [61]	Uganda	One tertiary oncology centre	Cross-sectional study	Survey Participants = 146 Age in years (*n* (%)): ≤ 30 = 47 (32.2%);31–40 = 58 (39.7%);41–50 = 20 (13.7%); ≥ 50 = 13 (8.9%); Missing = 8 (5.5%) Reported Gender: Female = 86 (58.9%); Male = 53 (36.3%); Missing = 7 (4.8%) Interpreted as: Sex Profession(s): Oncologist = 9 (6.2%); Doctor = 27 (18.5%); Nurse = 24 (16.4%); Allied health worker (lab, imaging, pharmacy, medical records officers) = 61 (41.8%); Biostatistics/Data manager/IT = 13 (8.9%); Administrator = 12 (8.2%)	Innovation: Electronic health record is the use of information technology to assist with health care related processes **Study outcome measurement** Measure: A four-item subscale measuring organizational readiness in implementing electronic health records in low- and middle-income countries using a 5-point Likert scale ranging from strongly agree to strongly disagree adapted from a study by Paré et al. [68] Reliability: Dillon- Goldstein’s rho = 0.79; *α* = 0.64 Validity: Convergent validity: Average variance extracted (AVE) = 0.48
Kenny, 2005 [62]	USA	Three army medical treatment facilities	Cross-sectional study	Registered nurses = 290 Age: Not reported Reported Gender: Male = 60 (20.7%); Female = 229 (79.0%); Missing = 1 (0.3%) Interpreted as: Sex	Innovation: This study did not have an explicit innovation. The purpose of this study was to examine individual and organization factors related to research use by nurses. Research use was defined as the use of research findings to guide nursing practice **Study outcome measurement** Measures: (1) Adapted Research Utilization survey by Estabrooks [87] to measure direct, persuasive and overall research use. All types of research use were single survey items scored using a 7-point Likert scale from "never" to "nearly every shift” Reliability: α (range) = 0.77–0.91; Validity: Not reported
Khera, 2018 [63]	USA	108 transplant centres	Cross-sectional study	Physicians = 316 Age: Not reported Sex and Gender: Not reported Professions: Physicians = 230 (77.4); Program Medical Director = 67 (22.6)	Innovation: The findings of a phase three, multicentre randomized control trial titled Blood and Marrow Transplant Clinical Trials Network (BMT CTN) 0201 [88] found that the use of bone marrow stem cells for unrelated donor hematopoietic cell transplantation is related to similar survival rates and less chronic graft versus host disease in patients with hematologic malignancies than the use of peripheral blood stem cells **Study outcome measurement** Measure: A 26-item survey developed by the authors according to the literature and key informant interviews with three researchers from BMT CTN 0201 study [88]. Outcome variables include physician reported personal and facility-level change in preference regarding unrelated donor graft use from peripheral blood source to bone marrow. These survey items were scored on a 5 -point Likert scale from very important to very unimportant Reliability: Not reported; Validity: Not reported
Korall, 2017, 2018 [64, 65] One study—two reports	Canada	13 long-term care homes	Cross-sectional study	Paid care providers = 529 Age in years (*n* (%)): 20–29 = 42 (7.9%); 30–39 = 87 (16.4%); 40–49 = 149 (28.2%); 50–59 = 187 (35.3%); 60–69 = 46 (8.7%); Missing/unknown = 18 (3.4%) Reported Gender: Female = 474 (89.6%); Male = 40 (7.6%); Missing/unknown = 15 (2.8%) Interpreted as: Sex Professions: Health care assistant/resident care aide = 290 (54.8%); Licensed practical nurse = 84 (15.9%); Registered nurse = 40 (7.6%) Resident care coordinator = 13 (2.4%); Manager = 14 (2.6%); Recreational/occupational/ physiotherapist = 24 (4.5%); Unit/program clerk = 18 (3.4%); Missing/unknown = 49 (9.3%)	Innovation: Hip protectors are protective undergarments with either a hard shield or soft pads sewn into its sides to cover the skin over the lateral aspects of the proximal femur. The purpose of hip protectors is to minimize the injury to the hip resulting from a fall **Study outcome measurement** Measures: A 15-item questionnaire titled as C-Hip Index, developed and tested for psychometric properties by authors [64] to measure affective and cognitive, behavioural and overall hip protector commitment Reliability: *α* (range) = 0.87–0.97 Validity: (1) Construct validity: Authors reported a two-factor structure as the result of an exploratory factor analysis: Factor 1 (affective and cognitive commitment) and Factor 2 (behavioural commitment) which loaded to a higher order factor called "commitment to hip protectors" with an eigen value of 1.386. *R*^2^ = 0.693. Both subscales had a factor matrix coefficient of 0.833 (2) Content validity index (CVI): Twelve items in C-Hip index had a CVI = 0.79 for both clarity and relevance. Range of item CVI = 0.55–0.82 (3) Convergent validity: Increase in self reported championing is associated with increase scores for the affective/cognitive, behavioural subscales and the entire C-Hip index (*p* < 0.01) (4) Concurrent validity: Significant lower median responses for individual subscales or entire C-Hip index amongst participants that responded that they were aware of a resident breaking a hip while wearing a hip protector (*p* < 0.01). Significant higher median responses for individual subscales or entire C-Hip index amongst individuals who responded that there was a champion at their long-term care home
Lago, 2013 [66]	Italy	103 neonatal intensive care units	Cross-sectional study	Sample details not reported	Innovation: The implementation of effective neonatal pain prevention programs according to best practice guidelines. These programs should include training and strategies to routinize the assessment of pain and the appropriate use of pharmacological and non-pharmacological therapies to prevent and treat pain **Study outcome measurement** Measure: A 58- item questionnaire created by the authors assessing neonatal intensive care units’ characteristics, availability pain control guidelines and neonatal intensive care units’ routine use of non-pharmacological and pharmacological pain-relieving interventions during invasive procedures. Frequency of pain-relieving interventions was measured on 4-point Likert scale from never (0–15%) to always (> 90%) Reliability: Not reported; Validity: Not reported
Papadakis, 2014 [67]	Canada	40 family health team clinics	Cross-sectional study	(1) Health care providers = 288 Age: Mean (SD) = 39.5 (17.3) years Sex and Gender: Not reported Profession(s): Practising physician = 80.7%; Medical resident = 5%; Nurse practitioner = 12.7% (2) Patient = 2501 Age: Mean (SD) = 47.7 (14.7) years Reported Sex: Male = 952 (38.1%); Female = 1549 (61.9%) Interpreted as: Sex	Innovation: Evidence-based smoking cessation treatments is composed of five strategies (denoted as 5 As): ask patients about their smoking status, advise patients to quit smoking, assess patient’s readiness to quit, assist with a quitting attempt using behavioural counselling or smoking cessation medications, and to arrange follow-up pertaining to smoking cessation **Study outcome measurement** Measures: (1) A health care provider survey created by the authors to assess family health teams characteristics and providers’ attitudes and believes towards evidence-based smoking cessation treatments (2) A patient evaluation survey created by the authors asking on a binary scale (yes or no) if the patient’s physician or other health care providers asked, advised, or assessed readiness to quit, and if the provider assisted, or arranged follow-up regarding smoking cessation Reliability: Not reported; Validity: Not reported
Paré, 2011 [68]	Canada	(1) Study 1: 11 home care organizations (2) Study 2: one hospital	Cross-sectional study	(1) Study 1: Registered nurses = 134 Age in years (%): ≤ 29: 14%; 30–39 = 23%; 40–49 = 35%; 50–59 = 26%; ≥ 60 years = 2% Reported Gender: Male = 2%; Female = 98% Interpreted as: Sex (2) Study 2: Clinicians = 237 Age (%): ≤ 29 years = 10%; 30 to 39 years = 21%; 40 to 49 years = 28% 50 to 59 years = 34%; ≥ 60 years = 7% Reported Gender: Male = 32%; Female = 68% Interpreted as: Sex Professions: Registered nurse = 57%; Social worker = 9%; Occupational therapist = 4%; Clinician (others) = 19%; Physicians = 12%	Innovation: The innovations in the two studies pertain to the implementation of clinical information system. In study 1, the innovation was a mobile computing project. The mobile computing project contains home care nursing policies and procedures and allows home care nurses to create individualized care plans for their clients and to document the care they provided. The innovation in study two was the electronic medical record. The purpose of this study was to determine the factors related to the readiness of the staff in implementing these technological innovations **Study outcome measurement** Measures: The authors created a survey according to Holt and colleagues’ conceptual model of organizational readiness [89]. The questionnaire has 39 items. Organizational readiness was measured on 4- item subscale, scored on a 5-point Likert scale ranging from strongly agree to strongly disagree adopted two studies [90, 91] Reliability: (1) Organizational readiness—*α*: Study 1 = 0.89; Study 2 = 0.88 Validity: (1) Construct validity: exploratory factor analyses showed that all scale items loaded highly (> 0.68) on a single factor (2) Convergent validity: Average variance extracted (study 1 = 0.88; study 2 = 0.86) was higher than inter-construct correlations (3) Discriminant validity: Cross-loadings (study 1 range = 0.85–0.91; study 2 range = 0.78–0.89) loaded more highly on their own factor than on other factors
Patton, 2013 [69]	England	153 emergency departments	Cross-sectional study	Lead clinicians = 153 Age: Not reported Sex and Gender: Not reported Professions: Not reported	Innovation: The assessment of alcohol consumption and provision of advice to decrease alcohol use by health care providers in the emergency department is an effective and cost-effective way of decreasing alcohol consumption and alcohol related harm **Study outcome measurement** Measure: A follow-up survey based on questions from a national emergency survey distributed in England in 2006 [92].The dependent variables were two survey items asking about emergency staff's access to training for screening and brief advice regarding alcohol consumption Reliability: Not reported; Validity: Not reported
Sharkey, 2013 [39]	USA	14 nursing homes	Non-controlled before and after study	Sample details were not reported	Innovation: The On-Time pressure ulcer quality improvement based on the integration of health information technology tools has three primary objectives: (1) utilize the knowledge and train certified nursing assistants to document and communicate their assessments to licensed staff through an electronic health system; (2) support collaborative and multidisciplinary clinical decision making through clinical decision support systems that summarize resident data from daily staff documentations; and (3) to establish a proactive practice focused on prevention and early treatment of pressure ulcers **Study outcome measurement** Measures: On-Time facilitators’ reports tracked implementation milestones achieved every 9 months and documented facility team characteristics, team skills and capacity. Milestones were tracked according to three levels: levels 1 to 3. The level equates to the number of process improvements implemented facility wide Reliability: Not reported; Validity: Not reported
Shea, 2016 [70]	USA	37 ambulatory clinics	Cross-sectional study	Health care providers = 596 Age: Not reported Sex and Gender: Not reported Professions: Not reported	Innovation: The innovation in this study was the meaningful use of electronic health records, or the ability to maximize the capacity of the electronic health record to improve quality, safety and efficiency of health care services. Meaningful use of the electronic health records is implemented in three stages. However, the authors were interested in the Stage 1 meaningful use because Medicare services must attest to this level of meaningful use 90 days post implementation of the electronic health records to receive monetary incentives. Stage 1 meaningful objective criteria includes 14 required core objectives (e.g. having an updated medication lists for patients) and 5 menu objectives selected from a set of 10 options (e.g. providing patient- specific educational materials) **Study outcome measurement** Measure: Survey created and administered by authors to clinics’ senior leaders. Meaningful use of electronic health records was quantified as the percentage of eligible providers in each clinic demonstrating all Stage 1 meaningful use objective criteria Reliability: Not reported; Validity: Not reported
Sisodia, 2020 [71]	USA	205 medical, surgical and mental and behavioural health clinics	Retrospective cohort study	Sample details not reported	Innovation: Patient-reported outcomes are questionnaires that is distributed to patients to assess their general health, quality of life, or health/symptoms pertaining to a specific disease **Study outcome measurement** Measure: Patient-reported outcomes collection rates were extracted from project logs within an enterprise data warehouse. These logs contained the number and type of patient related questionnaires administered to collect patient related outcomes by participating clinics in the most recent 6 months Reliability: Not reported; Validity: Not reported
Slaunwhite, 2009 [72]	Canada	46 units within one acute care facility 23 units with champions 23 units with no champions	Case–control study	Sample details not reported	Innovation: The introduction of unit champions can facilitate the uptake of the influenza vaccine amount hospital staff **Study outcome measurement** Measure: Annual influenza vaccination rates in matched hospital units (matched according to previous years influenza vaccination rates, physical size and primary function). Secondly, the authors assessed the change in annual influenza vaccination rates from the previous year for each hospital unit Reliability: Not reported; Validity: Not reported
Soni, 2016 [73]	India	One neonatal intensive care unit (NICU)	Interrupted time series	NICU patients = 648 Percentage of sample when KMC champions were absent in the NICU = 43.1% Age: Not reported Not specified Sex and Gender: Female % = 37.3%; Male % = 62.7% Interpreted as: Sex	Innovation: Kangaroo mother care has two main components: skin-to-skin care and breastfeeding. Kangaroo mother care is a safe and low-cost measure to reduce neonatal mortality **Study outcome measurement** Measures: Chart audits to determine overall use and initiation rate (neonates/30 days) of skin-to-skin care and breastfeeding documented on standardized forms. Average duration (hours/day) was only measured for skin-to-skin care because of the difficulty in differentiating between breastfeeding attempts and successful breastfeeding Reliability: Not reported; Validity: Not reported
Strasser, 2003 [74]	USA	203 cystic fibrosis care centres	Cross-sectional study	Clinic directors and coordinators of CF care centres = 289 Age: Not reported Reported Gender: Male: 114 (39.6%); Female: 174 (60.4%); Missing = 1 (0.3%) Interpreted as: Sex Profession(s): Director = 150 (52.1%); Nurse coordinator = 112 (38.9%); Nurse practitioner = 20 (6.9%); Nurse health educator = 6 (2.1%)	Innovation: The Agency for Healthcare Research and Quality (AHRQ) 5A Smoking Cessation Clinical Practice Guideline refers to five steps: ask, advise, assess readiness to quit, assist patients with quitting and to arrange follow-up regarding smoking cessation **Study outcome measurement** Measure: A survey developed by authors to examine factors reported by directors and coordinators of cystic fibrosis centres that may affect smoking cessation guideline implementation. The AHRQ 5 A (ask, advise, assess, assist and arrange follow-up) model smoking cessation guideline was the guideline assessed by the survey. The outcome variable was measured with a dichotomous (yes/no) question asking whether the AHRQ 5 A has been implemented to address cystic fibrosis patient’s parentals smoking behaviours Reliability: Test–retest survey reliability (*n* = first 30 respondents): Kendall’s tau = 1.00, *p* < .01; Spearman’s *r* = 1.00, *p* < .01 Validity: The survey was approved for content validity by an expert panel (a pulmonologist and two doctoral trained researchers in medical education and health behaviour)
Tierney, 2003 [75]	USA	Public health clinics and pediatrician practices (number not reported)	Mixed study (generic qualitative and cross-sectional)	(1) Public Health Clinics providers = 440 (2) Pediatricians = 434 Age: Not reported Sex and Gender: Not reported Profession(s): Not reported	Innovation: Reminder and recall immunization systems are routine communication processes (via telephone or mail) with children’s parents at preselected ages to remind them of an upcoming or past-due immunization or wellness check up. Routine immunization assessments refer to the measurement of immunization coverage rates at least every 2 years **Study outcome measurement** Measure: A 21-item survey created by the authors to assess five domains: messages to parents, barriers to implementation of reminder or recall messaging systems, other immunization practices (assessments, feedback), practice attitudes about immunization and characteristics and demographics Reliability: Not reported; Validity: Not reported
Ward, 2004 [76]	USA	109 Veterans Affairs medical centres	Cross-sectional study	Quality managers = 109 Age: Not reported Sex and Gender: Not reported Profession(s): Not reported Patients = not reported Age: Mean (range) = 66 (59 - 73) years Gender: Males: 96% Females: 4%; Range of males in all centres = 90–99% Interpreted as: Sex	Innovation: The implementation and health care providers' adherence to diabetes guidelines pertains to glycemic, lipid and blood pressure screening and control **Study outcome measurement** Measures: (1) A 31-item questionnaire distributed to quality managers assessing organizational context related to diabetes guideline implementation. Provider process measures in the survey included performing the following: HbA_1c_ screen (annually), foot screening (annually), lipid screening (biannually), renal screening (biannually), eye screen (annually) (2) Patient outcome measures include glycemic control (HbA_1c_ < 9.5%), non-smoker status, Lipid control (LDL ≤ 130 md/dL) and hypertension control < 140/90 mm Hg). These data were extracted from the 1999 Veterans Health Survey and the 2001 Veterans Satisfaction Survey Reliability: Not reported; Validity: Not reported
One study, two reports: Weiler, 2012, 2013 [77, 78]	USA	3 private ambulance companies and 3 public fire departments	Interrupted time series	Emergency Medical Service workers = 190 [77]; 221 [78] Age: Range = 18–65 years old Sex and Gender: Not reported Professions: Not reported	Innovation: Patient transfer board or slide board is a foldable board that aids with lateral transfers by bridging the gap between the bed and hospital stretcher and facilitate sliding of the patient from the stretcher to the bed and vice versa **Study outcome measurement** Measures: This study used scales that the authors formulated according to existing validated instruments: (1) “Intention to use the transfer board” scale (3 item scale) was based on Dishaw and Strong [93] (2) Ergonomic advantage of transfer boards (5 item scale) was based on Moore and Benbasat [94] Reliability: Not reported Validity: Ergonomic advantage- Factor loadings ranged from 0.62 to 0.81. Validity not reported for intention to use scale
Westrick, 2009 [79]	USA	104 community pharmacies	Cross-sectional study	Pharmacy staff = 104 Age: Not reported Reported Sex: Male = 65 (64.0%); Female = 35 (36.0%) Interpreted as: Sex Professions: Staff pharmacist = 13 (14.1%); Manager = 67 (72.8%); Owner/partner = 12 (13%)	Innovation: Pharmacy-based in-house immunization services is the administration of vaccines by pharmacists at their designated health care setting **Study outcome measurement** Measure: A questionnaire created by the authors that assesses pharmacy staff’s perspectives regarding the following criteria relevant to the sustainability of an in-house pharmacy immunization services (5 subscales): champion effectiveness (strategic and operational), formal evaluation, degree of modification, compatibility and sustainability of immunization services. The sustainability scale was based on Goodman and colleagues [95]. The subscales composed of either 4 to 6 items or scored on a 5-point Likert scale except for formal evaluation, which is a single dichotomous item. Reliability: *α* (range for all five subscales except formal evaluation) = 0.71–0.85. Formal evaluation was not assessed for reliability Validity: Not reported
Whitebird, 2014 [41]	USA	42 clinics from 14 medical groups	Mixed study (Generic qualitative and prospective cohort)	Patients in the Depression Improvement Across Minnesota: Offering a New Direction (DIAMOND) program at 6 months follow-up = 5258 Age: Not reported Sex and Gender: Not reported	Innovation: The DIAMOND program aims to provide collaborative depression care and consultive support to primary care clinics throughout Minnesota and Western Wisconsin. The DIAMOND program is composed of six aspects: (1) the use of the Patient Health Questionnaire-9 (PHQ-9) [96] to assess and monitor patient’s condition and progress; (2) systematic tracking of patients; (3) use of evidence-based guidelines to guide treatment; (4) dissemination of relapse prevention education to health care staff; (5) the presence of a care manager whose role is to educate, coordinate and support care services; and (6) the presence of a consulting psychiatrist collaborating with the care manager to review cases and provide treatment change recommendations **Study outcome measurement** Measure: Standardized monthly data reports regarding the number of eligible patients enrolled into the DIAMOND program (patients with a PHQ-9 ≥ 10) and remission rates (patients with a PHQ-9 < 5) every 6 months Reliability: Not reported; Validity: Not reported
Zavalkoff, 2015 [80]	Canada	1 pediatric intensive care unit (PICU)	Interrupted time series	Sample: Pediatric patients = 3100 Age: Not reported Sex and Gender: Not reported	Innovation: The introduction of a champion lead and an interdisciplinary policy dictating health care teams to systematically assess and discuss daily the appropriateness of continued use and/or removal of urinary catheters in patients **Study outcome measurement** Measures: Secondary data analysis of urinary catheter device utilization ratio in children admitted to the PICU between April 1, 2009, and June 29, 2013, according to hospital acquired surveillance database. Urinary catheter device utilization ratio was calculated by taking the number of days that a patient was exposed to a urinary catheter divided by the number of days that the patient was admitted in the PICU Reliability: Not reported; Validity: Not reported

^a^(Ash, 1997 [50]): This is a calculated sample size based on the reported response rate (31 and 48% response rate for informatics professionals (*n* = 629) and library workers (*n* = 706)). However, this calculated sample size only equates to 40% response rate, while the authors state having a 41% response rate

### Methodological quality

Of the 35 included studies, 19 (54.3%) were rated as strong [47, 48, 52, 58–65, 67, 68, 73–80], 11 (33.3%) were rated as moderate [39–41, 49, 50, 54, 56, 57, 70–72] and 5 (13.9%) were rated as weak [51, 53, 55, 66, 69] (See Additional file 5). Lower methodological quality was generally attributed to the lack of description of study participants and setting, lack of reliable and valid measures used to assess exposure to champions and study outcomes and the lack of processes used for random allocation and concealment of participant allocation to groups.

### Description and operationalization of champions

Overall, there was a scarcity of demographic information reported on the champions. None of the included studies reported the age of the champions, and only one study reported the sex of the champion [80]. Nine studies identified the profession of the champions as either nursing or medicine [49, 51, 54, 55, 66, 70, 72, 74, 75].

Most studies (*n* = 25 of 35, 71.4%) operationalized champions as the perceived presence or absence of champions by survey respondents measured by single dichotomous (“Yes/No responses) or Likert items. Tables 5 and 6 detail operationalization of champions for each included study.

Four of the 35 studies (11.4%) described the appointment of champions in their study setting [54, 72, 73, 80]. There was a range of one champion [80] to six champions [54] in these studies [54, 72, 73, 80]. Two of these studies described the activities performed by the champions: (1) training nurses in the Kangaroo mother care and providing educational videos to mothers of neonatal intensive care patients [73] and (2) creating and implementing a protocol related to appropriate urinary catheter use and auditing urinary catheter use [80]. The other two studies detailed the training provided to champions but not their activities [54, 72].

The remaining 6 of 35 studies (17.1%) operationalized champions using five unique subscales (two studies used the same subscale) that assessed the presence of a champion who possessed or performed particular attributes, roles, or activities [50, 59, 61, 68, 77–79]. Overall, these measures demonstrate that champions can perform differing roles and activities from enthusiastically promoting or role modelling behaviour towards a particular innovation, to broader leadership roles (e.g. managing or acquiring resources). In 4 of the 6 studies (66.7%) [59, 61, 68, 79], the used champion subscale had acceptable internal consistency (*α* ≥ 0.70 [97]); one study (16.7%) reported that the used champion subscale had low internal consistency (*α* = 0.43) [50]. In 2 of the 6 studies (33.3%), the authors conducted an exploratory factor analysis and reported that the champion items loaded highly to a single factor [68, 77, 78]. The champion subscales were part of five larger questionnaires that measured another construct: (1) organizational readiness in adopting electronic health technologies [56, 63]; (2) organizational factors affecting adoption of electronic mail [45], transfer boards [72, 73] and e-health usage [54]; (3) sustainability of pharmacy-based innovations [74]. Furthermore, none of the included studies reported performing an evaluation on whether the champions’ activities were perceived to be helpful by the individuals who were intended to use the innovation. Also, none of the included studies assessed whether there was adequate exposure to champions to produce an effect.

### Categorization of study outcomes

Across all 35 studies, we extracted and categorized 66 instances for which the relationships between champions and innovation use or patient, provider, or facility/system outcome were evaluated. Some studies evaluated the relationships between champions and more than one dependent variable. Table 4 presents the relationships between champions and innovation use and/or the resulting impact of innovation use pertaining to patients, providers and systems/facilities for each of the included studies.

**Table 4:** Summary of champions’ effectiveness in increasing innovation use and improving outcomes

First author, year	Innovation Use		Outcome (impact)
	**Conceptual (knowledge)**	**Instrumental (adherence)**	
Albert, 2012 [47]		(?) H (Consistent use of standard orders)	
Alidina, 2018 [48]		(?) H (Regular use of operating cognitive aids)	
Anand, 2017 [49]		( +) H (Continuous pain assessments)	
Ash, 1997 [50]		(?) S (Implementation of electronic mail)	
Ben-David, 2019 [51]			( +) Patient (Decrease incidence of central-line-associated blood stream infection)
Bentz, 2007 [52]		( +) H (Referrals of patients to the Oregon Tobacco Quitline)	
Bradley, 2012 [53]			( +) Patient (Decrease 30-day risk-standardized mortality rate post myocardial infarction)
Campbell, 2008 [54]		(?) H (Adoption of sepsis protocol)	
Chang, 2012 [40]		(ø) S (Depression care programs in primary care)	
Ellerbeck, 2006 [55]		(ø) H (Medications prescribed during and after myocardial infarction)	
Foster, 2017 [56]			( +) System (Decreased harm topics to quality of care (e.g. readmission)
Goff, 2019 [57]		( +) H (Adherence to best practices for medication/intervention prescribing)	(ø) Patient (Patient experience)
Granade, 2020 [58]		(?) H (Adherence to adult vaccination standards)	
Hsia, 2019 [59]		( +) S (Hospital medical services and processes performed using E-health technology)	
Hung, 2008 [60]			( +) P (Quality of life measures)
Kabukye, 2020 [61]	( +) H (Attitudes towards implementing electronic health record)		
Kenny, 2005 [62]		( +) H (Instrumental research use)	
Khera, 2018 [63]		(?) H (Preferred unrelated graft source for hematologic malignancies)	
One study, two reports: Korall, 2017, 2018 [64, 65]	( +) H (Commitment to hip protectors)		
Lago, 2013 [66]		(?) H (Non-pharmacological and pharmacological interventions during invasive procedures)	
Papadakis, 2014 [67]		( +) H (Delivery of evidence-based smoking cessation treatments)	
Paré, 2011 [68]	(?) H (Attitudes towards implementing electronic health record)		
Patton, 2013 [69]			( +) S (Provider’s access to training for screening and giving brief advice regarding alcohol use)
Sharkey, 2013 [39]		( +) S (Facility-wide health information clinical decision support system for preventing pressure ulcers)	
Shea, 2016 [70]		(ø) H (Meaningful use of electronic health records)	
Sisodia, 2020 [71]		( +) S (Success of patient-reported outcome collection program)	
Slaunwhite, 2009 [72]		( +) H (Uptake of influenza vaccine)	
Soni, 2016 [73]		( +) P (Kangaroo mother care: breastfeeding and skin-skin)	
Strasser, 2003 [74]		( −) H (Application of smoking cessation guideline)	
Tierney, 2003 [75]	( +) S (Intent by pediatrician practices to adopt reminder recall and immunization coverage rates)	( +) S (Pediatrician practices’ and public health clinics’ use of reminder recall and immunization coverage rate assessments)	
Ward, 2004 [76]		( +) H (Adherence to diabetes guidelines)	(ø) P (Improvement in patient parameters outlines by diabetes guideline)
One study, two reports: Weiler, 2012, 2013 [77, 78]	(?) H (Intention to use transfer boards)		( +) H (Ergonomic advantage of transfer boards)
Westrick, 2009 [79]			(ø) S (Adaption and sustainability of in-house pharmacy immunization services)
Whitebird, 2014 [41]		( +) S (Uptake of depression program)	(ø) P (Improvement in depression remission rates)
Zavalkoff, 2015 [80]		( +) H (Urinary catheter use)	

P = patient, H = provider, S = system/facility; ( +) = champions significantly increased innovation use/outcome of innovation use; ( −) = champions significantly decreased innovation use/outcome of innovation use; (?) = mixed findings related to champions effect on innovation use/outcome of innovation use; ø = no significant effect in increasing or decreasing innovation use/outcome of innovation use

### Champions’ effectiveness in increasing innovation use

Twenty-nine studies evaluated the effectiveness of champions in increasing innovation use: five studies evaluated the effectiveness of champions in increasing conceptual innovation use [61, 64, 65, 68, 75, 77, 78], and 25 studies evaluated the effectiveness of champions in increasing instrumental innovation use [39–41, 47–50, 52, 54, 55, 57–59, 62, 63, 66, 67, 70–76, 80]. One study evaluated both conceptual and instrumental innovation use [75]. Based on our vote-counting rules, we were able to draw conclusions between the use of champions and the following three categories: (1) providers’ knowledge and attitudes towards an innovation (conceptual innovation use); (2) providers’ use of an innovation (instrumental innovation use); and (3) system/facility’s establishment of processes that encourages use of best practices, programs and technology throughout an organization (instrumental innovation use). A description of each conclusion relative to these three categories of innovation use is detailed below. We present the study outcomes organized into their respective innovation use categories, the statistical analysis and approach and test statistics and measure of magnitude supporting our conclusions in Table 5.

**Table 5:** Champions’ effectiveness in increasing patient, provider and system/facility’s innovation use

Subcategory (# of studies)	First author, year	Study design	Champion operationalization	Outcome extracted from included study		Statistical analysis/approach		Test statistic (measure of magnitude)		*p*-value
**Conceptual innovation use** (knowledge/enlightenment)										
**Provider** (*n* = 4) **Conclusion**: Across four studies, there are mixed findings with respect to use of champions and improvement in providers’ conceptual innovation use										
Implementation of new technology or equipment (*n* = 4)	One study two reports: Korall, 2017, 2018 [64, 65]	Cross-sectional study	Existence of a champion of hip protectors (single item scored on a 5-point Likert scale)	Overall commitment to hip protectors		Bayesian Model Averaging logistic model		Logistic regression coefficient (95% CI) = 0.24 (0.17–0.31)		** < .05**
	Kabukye, 2020 [61]	Cross-sectional study	Presence of an effective champion (3-item survey scale by Paré et al.[68]	Organizational readiness in a low-resource setting		Structural equation model using a partial least square method		Path coefficient = 0.15		**.0299**
	Paré, 2011 [68]	Cross-sectional study	Presence of an effective champion (3-item survey scale)	Organizational readiness in a large teaching hospital		Structural equation model using a partial least squares method		Path coefficient = 0.23		** < .05**
				Organizational readiness in implementing a mobile computing system for home care				Path coefficient = 0.05		> .05
	One study, two reports: Weiler, 2012, 2013 [77, 78]	Interrupted time series	Endorsed by champions (three items rated at a 6-point Likert scale based on Mullins et al. [98]	Intention to use transfer boards 2 months post-introduction of transfer boards		Stepwise logistic regression		Partial *R*^*2*^*a* = 0.036 *C(p)* = − .041 *F* = 16.25		** < .0001**
						Structural equation model using a maximum likelihood method		Path coefficients (95 CI) = 0.27 (− .0156–.5556)		> 0.05^a^
**System/Facility** (*n* = 1) **Conclusion**: There is a study suggesting that the use of champions is related to system/facility’s conceptual innovation use										
Implementation of best practices related to vaccination processes (*n* = 1)	Tierney, 2003 [75]	Mixed study (generic qualitative and cross-sectional)	Presence of a champion lead (“Yes/No” survey item)	Pediatrician practices’ likelihood or intent to adopt reminder and recall system in their practice in a year	Multivariable linear regression		Test statistic not reported		** < .03**	
				Pediatrician practices’ likelihood or intent to adopt immunization coverage rates assessments in their practice in a year			Test statistic not reported		**.002**	
**Instrumental Innovation Use** (adherence in using the innovation (evidence-based practice or technology))										
**Patient** (*n* = 1) **Conclusion**: There is a study suggesting that the use of champions is related to improving patients’ instrumental innovation use										
Implementation of Kangaroo-Mother Care (*n* = 1)	Soni,2016 [73]	Interrupted time series	Absence of champions (two champion were present from January 5, 2010–July 31, 2011; transition period from August 1, 2011, to July 31, 2012; champion was absent from August 1, 2012, to October 7, 2014)	Initiation rate of skin to skin by mothers of neonatal intensive care unit (NICU) patients		Competing-risks regression model and observation-weighted linear polynomial test		Subhazard rate ratios (SHR)^c^ (95 CI) = 0.62 (0.47 − 0.82)		** < .001**^**b**^
				Overall use of skin to skin by mothers of NICU patients		Multivariate logistic regression and observation-weighted linear polynomial test		OR (95 CI) = 0.49 (0.34–0.70)		**.004**^**b**^
				Average duration of skin to skin provided by mothers of NICU patients		Multivariate linear regression and observation-weighted linear polynomial test		*β* (95 CI) = − 1.47 (− 2.07 to − 0.86)		** < .001**^**b**^
				Initiation rate of breastfeeding by mothers of NICU patients		Competing-risks regression model and observation-weighted linear polynomial test		SHR (95 CI) = 0.88 (0.68–1.14)		.30^b^
				Overall use of “breastfeeding” by mothers of NICU patients		Multivariate logistic regression and observation-weighted linear polynomial test		OR (95 CI) = 0.89 (0.55–1.44)		0.61^b^
**Provider** (*n* = 17) **Conclusion**: Across 17 studies, there are mixed findings with respect to use of champions and improvement in providers’ instrumental innovation use										
Implementation of best practices for smoking cessation (*n* = 3)	Bentz, 2007 [52]	Cluster randomised trial	Presence of a champion (“Yes/No” item determined through structured interviews with clinic managers or lead nurses)	Monthly rates of documented clients connected by health care providers to the Oregon Tobacco Quitline		Generalized estimating equations		OR (95 CI) = 3.44 (2.35–5.03)		** < .05**
	Papadakis, 2014 [67]	Cross-sectional study	Presence of physician champion (“Yes/No” survey item)	Frequency of evidence-based smoking cessation treatments delivered by health care providers		Multivariable logistic regression		OR (95 CI) = 2.0 (1.1–3.6)		** < .01**
	Strasser, 2003 [74]	Cross-sectional study	Presence of a designated champion (single item rated on a 6-point Likert scale)	Extent that health care providers apply smoking cessation guideline to help parents of cystic fibrosis patients quit smoking		Multivariable logistic regression		*β* (SE) = − .7570 (0.2110) OR (95 CI) = 0.469 (0.310–0.709)		**0.0003**
Implementation of best practices related to vaccination processes *(n* = 3)	Albert, 2012 [47]	Cross-sectional study	Presence of an immunization champion on site (“Yes/No” survey item)	Consistent use of standard orders for influenza vaccines only by non-physician staff		Multivariable logistic regression		OR (95% CI) = 1.12 (0.72–1.76)		> .05
				Consistent use of standard orders for both influenza vaccine and PPV by non-physician staff				OR (95% CI) = 1.67 (1.01–4.54)		**.046**
	Granade, 2020 [58]	Cross-sectional study	Presence of immunization champions (“Yes/No” survey item)	Primary care clinicians’ adherence to adult vaccination standards		Multivariable logistic regression		APR (95% CI) = 1.40 (1.26–1.54)		** < .05**
				Pharmacist’s adherence to adult vaccination standards				APR (95% CI) = 1.20 (0.96–1.49)		> .05
	Slaunwhite, 2009 [72]	Case–control study	23 champions randomly allocated to 23 hospital units versus 23 matched units with no champion	Difference in overall health care providers vaccination rates between champion and non champion units		*t* -test		*t* (22) = 2.86 (11% higher vaccination rate in champion units)		** < .03**
				Percentage change in health care provider vaccination rates from previous year in champion units				*t* (21) = 4.38 (increase from 44 to 54%)		** < .001**
Implementation of new technology/equipment (*n* = 2)	Alidina, 2018 [48]	Cross-sectional study	Presence of an implementation champion for cognitive aids (selected as an important facilitator from a list of facilitators)	Regular use of operating cognitive aids during applicable clinical events		Chi square		Test statistic not reported		0.8968
			Absence of an implementation champion for cognitive aids (selected as important barrier from a list of barriers)	Regular use of operating cognitive aids during applicable clinical events		Multivariable logistic regression		OR (95% CI) = 0.44 (0.23–0.84)		**.0126**
	Shea, 2016 [70]	Cross-sectional study	Presence of nurse champions (“Yes/No” survey item)	Percentage of providers in a clinic demonstrating Stage 1 meaningful use of electronic health records		Multivariable logistic regression		OR (95 CI) = 0.99 (0.60–1.65)		.983
Implementation of best practices related to pain management in neonatal intensive care units (*n* = 2)	Anand, 2017 [49]	Prospective cohort study	Presence of a nurse^d^ champion (“Yes/No” survey item)	Number of continuous pain assessments performed and documented by nurses per day for 1 month in neonatal intensive care units		Generalized estimating equations		OR (95 CI) = 2.54 (1.27–5.11)		**0.009**
	Lago, 2013 [66]	Cross-sectional study	Presence of a local champion (single item asking whether a physician champion, a nurse champion, both types of champions, or no champion was present)	Routine use (> 90% of the time) of non-pharmacological and pharmacological pain management interventions during invasive procedures in neonatal intensive care units		Stepwise logistic regression		Six out of 11 interventions: (1) Heel prick: OR (95 CI) = 2.78 (1.2–6.43) (2) Venipuncture: OR (95 CI) = 2.59 (1.13–5.96) (3) PICC insertion: OR (95 CI) = 3.33 (1.38–8.02) (4) Tracheal intubation: OR (95 CI) = 2.68 (1.17–6.16) (5) Mechanical ventilation: OR (95 CI) = 3.74 (1.5–9.32) (6) Chest tube insertion: OR (95 CI) = 3.26 (1.31–8.1)		** < 0.05**
								Five out of 11 interventions: (1) Tracheal Aspiration: OR (95 CI) = 1.96 (0.82–4.66) (2) Nasal CPAP: OR (95 CI) = 1.98 (0.87–4.53) (3) Lumbar puncture: OR (95 CI) = 1.99 (0.86–4.59) (4) ROP screening: OR (95 CI) = 2.35 (0.96–5.8) (5) Postoperative pain: OR (95 CI) = 1.58 (0.56–4.43)		> 0.05
Implementation of best practices related to prevention, identification and management of infections (*n* = 2)	Campbell, 2008 [54]	Non-controlled before and after study	Appointment of six nurses (two for each shift) champions for 4 weeks	Intensive care unit nurses’ compliance with sepsis-screening protocols		Chi square		*χ*^2^ = 30.86		** < .001**
				Physician’s initiation of sepsis protocol for patients with severe sepsis				*χ*^2^ = 0.563		.453
	Zavalkoff, 2015 [80]	Interrupted time series	Appointment of a single physician champion to lead projects decreasing catheter associated urinary tract infections	Urinary catheter-use ratio in a pediatric intensive care		Binomial regression (PROC GENMOD, binomial distribution, canonical link)		OR (95% CI) = 0.83 (0.77–0.90)		** < .05**
Generic implementation of best research evidence (*n* = 2)	Kenny, 2005 [62]	Cross-sectional study	Presence of a champion (“Yes/No” survey item)	Nurses’ direct (instrumental) research use		Pearson’s correlation coefficient		*r* = .250		**.001**
	Goff, 2019 [57]	Cross-sectional study	Presence of a designated quality champion (“Yes/No” survey item)	Average clinical quality scores (adherence of providers to best practices in prescribing treatments for diseases (e.g. asthma, diabetes)		ANOVA		Test statistics not reported (Mean difference = 0.2 favouring presence of a champion)		**.03**
Implementation of diabetes guideline (*n* = 1)	Ward, 2004 [76]	Cross-sectional study	Presence of champion (single item rated on a 5-point Likert scale)	Provider process measures relative to guideline-based diabetes management		Multivariable predictor generalized estimating equation		*β* (SE) = 1.24 (0.51)		**.02**
Implementation of best practices related to medications prescribed during or after an acute myocardial infarction (*n* = 1)	Ellerbeck, 2006 [55]	Cross-sectional study	Presence of a physician champion (“Yes/No” survey item)	Aspirin use at admission		Generalized estimating equations		OR (95% CI) = 1.31 (0.87–2.01)		> .05
				Aspirin use at discharge				OR (95% CI) = 1.17 (0.69–2.02)		> .05
				Beta-blockers use at admission				OR (95% CI) = 1.45 (0.91–2.31)		> .05
				Beta-blockers use at discharge				OR (95% CI) = 4.14 (1.66–11.66)		** < .05**
Implementation of the findings of a phase III, multicentre randomized control trial (BMT CTN 0201) [88] study *(n* = 1)	Khera, 2018 [63]	Cross-sectional study	Engagement of local champions (single item scored on a 5-point Likert scale)	Physician reported personal change in preferred unrelated donor graft source for patients with hematologic malignancies from peripheral blood source to bone marrow		Multivariable logistic regression		OR (95 CI) = 1.91 (0.87–4.19)		.11
				Physician reported transplant centre change in preferred unrelated donor graft source for patients with hematologic malignancies from peripheral blood source to bone marrow				OR (95 CI) = 3.18 (1.29–7.85)		**.01**
**System/Facility** (*n* = 7) **Conclusion:** Across seven studies, **t**he use of champions was reported to be related to increase in system/facility instrumental innovation use										
Implementation of technology /equipment (*n* = 3)	Ash, 1997 [50]	Cross-sectional study	Champion scale formulated from existing measures (unknown number of items and lack of detail on items reported (rated on a 5-point Likert scale)	Infusion of electronic mail		Multivariable linear regression		*β* = 0.09		.52
				Diffusion of electronic mail				*β* = 0.34		**.01**
	Hsia, 2019 [59]	Cross-sectional study	Presence of leadership's e-health championing behaviour (6-item survey scale)	Extent of hospital medical services and work processes are performed by health care providers using E-health technologies		Structural equation model using a partial least square method		Path Coefficient = 0.280		** < .05**
	Sharkey, 2013 [39]	Non-controlled before and after study	Presence of an internal champion (“Yes/No” question in facilitator reports)	Facility-wide implementation of at least two process improvements focused on using health information technology as a medium for clinical decision support to prevent pressure ulcers in nursing homes (labelled as “Level 2 outcome” by authors)		Nonparametric Spearman correlation		*ρ* = 0.65		**.013**
				Facility-wide implementation of three or more process improvements focused on using health information technology as a medium for clinical decision support to prevent pressure ulcers in nursing homes (labelled as “Level 3 outcome” by authors)				*ρ* = 0.75		**0.002**
Implementation of a depression care programs (*n* = 2)	Chang, 2012 [40]	Cross-sectional study	Presence of clinical champion (“Yes/No” survey item)	Collocation model implemented		Multivariable logistic regression models		OR (95 CI) = 2.36 (1.14–4.88)		** < .05**
				TIDES model implemented		Bivariate regression analysis		OR (95 CI) = 0.59 (0.20–1.78)		> .05
				BHL model implemented				OR (95 CI) = 0.65 (0.14–2.98)		> .05
				No depression care improvement model implemented				OR (95 CI) = 0.63 (0.31–1.29)		> .05
	Whitebird, 2014 [41]	Mixed study (Generic qualitative and prospective cohort)	Presence of a strong primary care provider champion (“Yes/No” extracted from quality improvement narrative reports)	Average monthly activation rate (patients entering the program per number of full-time health care provider)		Pearson’s correlation coefficient		*r* (95 CI) = 0.60 (0.10–0.86)		** < .05**
Implementation of patient-reported outcomes collection program (*n* = 1)	Sisodia, 2020 [71]	Retrospective cohort study	Presence of a clinician champion (“Yes/No” survey item)	Patient-reported outcomes (PRO) collection rate per clinic in the most recent 6 months		Multivariable linear regression		Collection rate change (95 CI) = 11.2 (2.5–20.0)		**.01**
				PRO successful collection rate (50% or greater) in a 6-month period		Multivariable logistic regression		OR (95 CI) = 3.36 (1.06–10.61)		**.04**
Implementation of best practices related to vaccination processes (*n* = 1)	Tierney, 2003 [75]	Mixed study (generic qualitative and cross-sectional)	Presence of a champion lead (“Yes/No” survey item)	Pediatrician practices’ current use of reminder and recall systems	Multivariable logistic regression		OR (95% CI) = 1.85 (1.08–3.18)		** < .05**	
				Public health clinic’s current use reminder and recall systems	Multivariable logistic regression		OR (95% CI) = 3.01 (1.34–6.73)		** < .05**	
				Pediatrician practices’ current use of immunization coverage rates assessments			OR (95% CI) = 1.38 (0.89–2.13)		** < .05**	
				Public health clinic’s current use of immunization coverage rates assessments			OR (95% CI) = Not reported		> .05	

^a^The authors reported a path coefficient that they stated is significant at a *p*-value of 0.1. Manual calculation of the 95% CI was done by JES to determine significance of both ergonomic advantage and intention to use at a *p*-value of .05

^b^These *p*-values were denoted as *p*(trend) by authors because an observation-weighted linear polynomial test was conducted to determine trends for differences in estimates across all the different models

^c^Subhazard rate ratios were calculated separately using separate competing risk regression models to consider discharge against medical advice prior to initiation of breast feeding and skin to skin

^d^In bivariate testing, both physician and nurse champions were significantly correlated with continuous pain assessments; the physician champion variable was not included in the multivariate testing because it was highly correlated with the nurse champion variable

*APR* adjusted prevalence ratio; *CI* confidence interval; *OR* odds ratio; *SE* standard error; *SHR* subhazard rate ratios

### Champions’ effectiveness in increasing provider conceptual innovation use

Four studies evaluated the effectiveness of champions in improving providers’ attitudes and awareness of new technology or equipment (conceptual innovation use) [61, 64, 65, 68, 77, 78]. One of the 4 studies used a quasi-experimental design [77, 78], while the other three studies were cross-sectional observational studies [61, 68, 77, 78]. Two of the 4 studies (50%) reported that champions were effective in increasing provider conceptual innovation use [61, 64, 65], and 2 of the 4 studies (50%) reported mixed findings regarding the effectiveness of champions in increasing provider conceptual innovation use [68, 77, 78]. Therefore, our findings suggest that the use of champions in these four studies [61, 64, 65, 68, 77, 78] was, overall, not consistently related to providers’ conceptual innovation use of new technology or equipment.

### Champions’ effectiveness in increasing provider instrumental innovation use

Seventeen studies evaluated the effectiveness of champions in increasing health care provider use of innovations (instrumental innovation use) [47–49, 52, 54, 55, 57, 58, 62, 63, 66, 67, 70, 72, 74, 76, 80]. One of the 17 studies was a clustered RCT, while 2 of the 17 studies used a quasi-experimental design [54, 80], and the remaining 14 studies were observational studies [47–49, 55, 57, 58, 62, 63, 66, 67, 70, 72, 74, 76]. Eight of the 17 studies (47.1%) reported that champions were effective in increasing provider’s use of innovations [49, 52, 57, 62, 67, 72, 76, 80]. Six of the 17 studies (35.3%) reported that mixed findings exist regarding the effectiveness of champions in increasing provider’s use of innovations [47, 48, 54, 58, 63, 66]. Two of the 17 (11.8%) studies reported that no relationship exists between champions and providers’ use of innovations [55, 70] and one of the 17 (5.9%) studies reported that champions decreased provider’s use of an innovation [74]. Therefore, our findings suggests that the use of champions in these 17 studies [47–49, 52, 54, 55, 57, 58, 62, 63, 66, 67, 70, 72, 74, 76, 80] was overall, not consistently related to providers’ use of best practice or technological innovations.

### Champions’ effectiveness in increasing system/facility instrumental innovation use

Seven studies evaluated the effectiveness of champions in increasing systems/facilities’ adoption of technology, best practices and programs (instrumental innovation use) [39–41, 50, 59, 71, 75]. One of the 7 studies used a quasi-experimental design [39], while the remaining studies used observational study designs [40, 41, 50, 59, 71, 75]. Five of the 7 (71.4%) studies reported that champions were effective in increasing the formation of policies and processes and increasing uptake of technology at hospitals [59] and nursing homes [39], best practices in public health and pediatric practices [75] and programs in primary care clinics [41, 71]. One of the 7 (14.3%) studies reported that mixed findings exist regarding the effectiveness of champions in increasing the adoption of a depression program in primary care clinics [40] and 1 of the 7 (14.3%) studies reported that champions had no effect in increasing uptake of electronic mail at academic health science centres [50]. Therefore, across these seven studies [39–41, 50, 59, 71, 75], the use of champions was overall related to increased use of technological innovations, best practices and programs by systems/facilities.

### Champions’ influence on outcomes

Ten studies evaluated the effectiveness of champions at improving outcomes. Six of the 10 studies evaluated the effectiveness of champions in improving patient health status or experience (patient outcomes) [41, 51, 53, 57, 60, 76]. One of the 10 studies evaluated the effectiveness of champions in improving provider’s satisfaction with the innovation [77, 78], and three studies evaluated the effectiveness of champions in improving system/facility-wide outcomes such as quality indicators [56], the establishment of organizational training programs [69], or sustainability of programs [79]. Based on our vote-counting rules, we drew conclusions between the use of champions and patient outcomes (see Table 6).

**Table 6:** Champions’ effectiveness on patient, provider and system/facility’s outcomes

Subcategory (# of studies)	First author, year	Study Design	Champion operationalization	Outcome extracted from included study	Statistical analysis/approach	Test statistic (measure of magnitude)	*p*-value
**Patient Outcomes** (*n* = 6) **Conclusion**: Across six studies, there are mixed findings pertaining to use of champions and improvement in patients’ outcomes related to innovation use							
Improvement in patient’s health outcomes (*n* = 4)	Ben-David, 2019 [51]	Cross-sectional study	Presence of ward infection control champions (survey item asking if a nurse or/and physician champion was present)	Monthly incidence rates of central-line-associated bloodstream infection	Negative binomial regression	Incidence rate ratio (95% CI) = 0.47 (0.31–0.71)	** < .001**
	Bradley, 2012 [53]	Cross-sectional study	Presence of one or more physician/nurse/ both/no champions (two “Yes/No” survey items asking the presence of physician/nurse champions)	30 days risk-standardized mortality rate post acute myocardial infarction in hospitals	Multivariate linear regression	*β* (95% CI) = − 0.695 (− 1.253 to − 0.137) (No champion vs nurse champion only)^a^	**.015**
						*β* (95% CI) = − 0.731 (− 1.404 to − 0.059) (Physician champions vs nurse champion only)	**.033**
						*β* (95% CI) = − 0.880 (− 1.442 to − 0.318) (Both physician and nurse champions vs nurse champion only)	**.002**
	Ward, 2004 [76]	Cross-sectional study	Presence of champion (single item rated on a 5-point Likert scale)	Patient outcome measures relative to guideline-based diabetes management	Single predictor generalized estimating equations	*β* (SE) = − 0.38 (0.39)	.3202
	Whitebird, 2014 [41]	Prospective cohort	Presence of a strong primary care provider champion (“Yes/No” extracted from quality improvement narrative reports)	Average monthly remission rates at 6 months (number of patients with a score of < 5 on the PHQ-9)	Pearson's correlation coefficient	*r* (95 CI) = 0.40 (− 0.16 to 0.77)	> .05
Quality of life (*n* = 1)	Hung, 2008 [60]	Cross-sectional study	Presence of practice (health promotion) champions (single item rated on a 5-point Likert scale)	Fewer numbers of unhealthy days in the past 30 days	Hierarchal generalized linear modelling	*β* (SE): 0.34 (0.07) OR (95 CI) = 1.41 (1.22–1.64)	** < .001**
				Fewer numbers of limiting days in the past 30 days		*β* (SE): 0.53 (0.19) OR (95 CI) = 1.71 (1.16–2.53)	** < .01**
				General health status		*β* (SE): 0.38 (0.09) OR (95 CI) = 1.47 (1.20–1.79)	** < .001**
Patient Experience (*n* = 1)	Goff, 2019 [57]	Cross-sectional study	Presence of a designated quality champion (“Yes/No” survey item)	Average patient experience scores of clinics that are part of the Massachusetts Health Quality Partners (MHQP)	ANOVA	Test statistics not reported (Mean difference = 0.09 favouring presence of a champion)	.29
**Provider Outcomes** (*n* = 1) **Conclusion**: There is a single study suggesting that the use champions is related to improvements in provider outcomes related to innovation use							
Satisfaction with practice (*n* = 1)	One study, two reports: Weiler, 2012, 2013 [77, 78]	Interrupted time series	Endorsed by champions (three items rated at a 6-point Likert scale based on Mullins et al. [98]	Reported ergonomic advantage 1-month post-introduction of transfer boards	Structural equation model using a maximum likelihood method	Path coefficients (95 CI) = 0.63 (.0664–1.1936)	** < 0.05**^**b**^
**System/Facility Outcomes (***n* = 3**)** **Conclusion:** Across three studies, there is a trend suggesting that use of champions is related to improvement in system/facility outcomes related to innovation use							
Hospital quality of care indicators (*n* = 1)	Foster, 2017 [56]	Non-controlled before and after study	An average of 0.1 champion fellows in 1160 hospitals (number of champion fellows)	Weighted composite score of quality of care—occurrence of 10 harm topics (e.g. readmissions) for 1 month	Multivariate linear regression	Adjusted effect over time: *β* = − 0.9 (negative *β* = more effective in this study)	**.008**
Access to training for alcohol cessation screening and advice (*n* = 1)	Patton, 2013 [69]	Cross-sectional study	Presence of champion (“Yes/No” survey item)	Emergency staff’s access to training for screening for alcohol consumption	Chi square	*χ*^2^ = 36.64	** < 0.001**
				Emergency staff’s access to training for providing brief advice regarding alcohol consumption		*χ*^2^ = 29.93	** < 0.001**
Compatibility and sustainability of in-house pharmacy immunization services (*n* = 1)	Westrick, 2009 [79]	Cross-sectional study	Strategic champion effectiveness (4-item scale on champion’s commitment, advocacy and ability to manage and acquire resources) adapted from Hays et al. [99]	Compatibility between immunization services and host pharmacy	Multivariable linear regression	*β* = 0.12	.300
				Sustainability of in-house pharmacy immunization services		*β* = 0.00	.978
			Operational champion effectiveness (4-item scale on champion’s knowledge, ability to manage an in-house immunization service, and to resolve conflicts) adapted from Hays et al. [99]	Compatibility between immunization services and host pharmacy	Multivariable linear regression	*β* = 0.31	**.005**
				Sustainability of in-house pharmacy immunization services		*β* = 0.09	.419
**Other Outcomes** (*n* = 1)^c^							
Adaptation and evaluation of in-house pharmacy immunization services (*n* = 1)	Westrick, 2009 [79]	Cross-sectional study	Strategic champion effectiveness (4-item scale on champion’s commitment, advocacy and ability to manage and acquire resources) adapted from Hays et al. [99]	Degree of modifications made to in-house pharmacy immunization services	Multivariable linear regression	*β* = 0.05	.705
				Formal evaluation of in-house pharmacy immunization services		*β* = 0.26	**.038**
			Operational champion effectiveness (4-item scale on champion’s knowledge, ability to manage an in-house immunization service, and to resolve conflicts) adapted from Hays et al. [99]	Degree of modifications made to in-house pharmacy immunization services	Multivariable linear regression	*β* = 0.05	.698
				Formal evaluation of in-house pharmacy immunization services		*β* = 0.09	.419

^a^In this study, groups exposed to only nurse champions had the highest risk-standardized mortality rate (RSMR; RSMR = 16.2); hence, it was the reference variable

^b^The authors reported a path coefficient that they stated is significant at a *p*-value of 0.1. Manual calculation of the 95% CI was done by JES to determine significance of both ergonomic advantage and intention to use at a *p*-value of .05

^c^Other outcomes were not considered in analysis

### Champions’ influence on patient outcomes

Six studies evaluated the effectiveness of champions in improving patient outcomes [41, 51, 53, 57, 60, 76]. All six studies used observational study designs. Three of the 6 studies (50%) reported that champions were effective in decreasing adverse patient outcomes [51, 53] or improving patients’ quality of life [60], while the other three studies (50%) reported that champions did not have a significant effect in improving patients’ standardized depression scale scores [41], patients’ laboratory tests and other markers related to diabetes [76] or their satisfaction with health services [57]. Therefore, across these six studies [41, 51, 53, 57, 60, 76], the use of champions was overall, not consistently related to improvements in patient outcomes.

### Champions’ effectiveness on innovation use and outcomes

Three of the 35 studies evaluated the effectiveness of champions in increasing both innovation use and outcomes [41, 57, 76]. In these three studies, the use of champions improved health care providers’ use of best practices [57, 76] and the uptake of a depression program by facilities [41] but did not impact patient outcomes.

### Sensitivity analysis and sub-group analysis of data

We found that when weaker quality studies were removed, the number of categories that we can make conclusions on, or their respective conclusions, did not change. Further, our conclusions did not change when we examined study findings across studies (*n* = 25 of 35, 71.4%) that operationalized champions using dichotomous (presence/absence) measures. We could not make conclusions but observed trends across studies that used more nuanced measures or appointed champions for their study (*n* = 10 of 35, 28.6%), because the categories of innovation use and outcomes in this subset had less than four included studies. In this subset of studies, a positive trend was suggested between use of champions and improvement in provider instrumental innovation use, according to three quasi-experimental studies [54, 72, 80].

## Discussion

### Summary of study findings

In this review, we aimed to summarize how champions are described and operationalized in studies that evaluate their effectiveness. Secondly, we assessed whether champions are effective at increasing innovation use or improving patient, provider and system/facility outcomes.

#### Description and operationalization of champions

We found that most studies evaluating the effectiveness of champions operationalized exposure to champions using a single item scale that asked whether participants perceived a presence or absence of a champion. Furthermore, we found that there was minimal demographic information provided regarding the champions in the included studies. Our findings add to the review by Miech and colleagues [21], revealing four additional subscales [50, 59, 77–79] measuring champions to the three champion subscales [40, 68, 100] they cited in their review. Our results reinforce Miech and colleagues’ [21] claim that more nuanced measures are needed to examine champions, as our review also only found champion subscales and did not locate a full instrument intended to measure the champion construct.

#### Champions’ effectiveness

Our review demonstrates that causal relationships between deployment of champions and improvement in innovation use and outcomes in health care settings cannot be drawn from the included studies because of the methodological issues (i.e. lack of description of champions, lack of valid and reliable measures used and use of observational study designs) present in most of these studies. Hall and colleagues also found low confidence evidence pertaining to champions’ effectiveness in guideline implementation in long-term care [22]. When we tried to make sense of the evidence, we found that across seven studies, champions were related to increased use of innovations at an organizational level [39–41, 50, 59, 71, 75]. Our findings indicate that champions do not consistently improve provider’s attitudes and knowledge across four studies [61, 64, 65, 68, 77, 78], provider’s use of innovations across 17 studies [47–49, 52, 54, 55, 57, 58, 62, 63, 66, 67, 70, 72, 74, 76, 80], or patient outcomes across six studies [41, 51, 53, 57, 60, 76]. We only found one study suggesting that the use of champions is associated with decreased provider instrumental innovation use [74], and none of the studies reported that the use of champions resulted in worse outcomes or harms. Damschroder and colleagues [101] reported that a single champion may be adequate in facilitating the implementation of technological innovations, but a group of champions composed of individuals from different professions may be required to encourage providers to change their practices. Furthermore, the myriad of mixed findings pertaining to the effectiveness of champions could be related to the lack of (1) description of the champions; (2) fidelity of the champion strategy; (3) evaluation of champion’s activities and level of exposure to champions; and (4) assessments of confounding contextual factors affecting champions’ performance. According to Shaw and colleagues [102], champions can undertake many roles and activities and that the assumption that champions operate in the similar manner may make comparisons difficult if these distinctions are not clarified.

Our results draw similar conclusions from the four cited published reviews on champions [21–24]. However, as detailed in this ‘Discussion’ section, our review (1) synthesized more quantitative evidence across varying health care settings and innovation types to reinforce the conclusions made from the past reviews; (2) highlighted areas where adequate research is conducted around champions and innovation use and outcomes; (3) identified four additional scales used in champion effectiveness studies not previously cited in previous reviews; and (4) provided implications of our findings in research and deployment of champions.

### Implications of study findings

One implication of our study findings is that it provides a summary of 35 studies that evaluate the effectiveness of champions across varied health care settings and innovation types. Furthermore, we identified areas for which the effectiveness of champions was not well examined: (1) patients’ innovation use, (2) organizational conceptual innovation use, (3) provider outcomes, (4) and system/facility outcomes. In addition, our findings suggest that individuals who are thinking of mobilizing champions should begin by reflecting on their intended implementation goal (innovation use or outcomes by patients, providers, or by systems/facilities). If the goal is to increase organizational use of innovations, then there is some evidence to support the position that the use of champions may be beneficial. However, if the goal is to improve innovation use by providers and patients, or improve outcomes, individuals implementing EBP should be mindful when using champions until more conclusive evidence exists to support their effectiveness pertaining to these goals. Although there is a lack of evidence suggesting that the use of champions can be harmful, there are opportunity costs that come with deploying champions (e.g. clinician time and sometimes monetary costs) that may be better used to deploy a different implementation strategy. Furthermore, our findings imply that future effectiveness studies should examine whether champions perform distinct roles or activities depending on the innovation type or level of implementation (i.e. system/facility or individual (providers or patients)). To differentiate between several types of champions present in implementation requires future studies to provide more detailed descriptions of the champion strategy. One way to achieve this objective is through the development and use of valid, reliable and pragmatic tools that evaluate champions’ activities and exposure to champions. A second means is through the conduct of process evaluations in conjunction with experimental studies. Strauss and colleagues [38] defined process evaluations as qualitative or mixed method studies that are intended to describe the process of implementation, and the experiences of the individuals involved in implementation. Michie and colleagues [103] also highlighted that triangulation of qualitative data with findings of experimental studies would increase the validity of conclusions that an observed change is due to the applied knowledge translation strategy. Lastly, process evaluations may also help inform the optimal dose of champions required to achieve an implementation goal [38].

### Limitations

#### Limitations of our review

Apart from theses and dissertations, we did not consider other grey literature in this study. Moreover, our eligibility criteria excluded studies that were not written in English. Further, our conclusions, made through vote counting, does not consider the effect size and the sensitivity of each individual study in estimating these effect sizes [45]. We tried to mitigate this limitation by reporting both the effect sizes and the sample sizes for each study. Moreover, as we only included studies that explicitly called the individual a champion, our review excluded other studies that deployed an individual that could have performed similar roles or activities as a champion but was not labelled a champion.

#### Limitations in the primary studies

The methodological, outcome measure and topic heterogeneity across the included studies did not allow us to conduct a meta-analysis to calculate the magnitude of champions’ effectiveness. The lack of description or evaluation of the champions’ attributes, roles and activities in most of the studies makes it difficult to decipher why the effectiveness of champions was found primarily mixed. In addition, the minimal use of both experimental research designs and reliable and valid measures to assess exposure to champions across the included studies makes it impossible to draw causal conclusions. Lastly, the included studies were mostly conducted in North American or European countries; hence, these findings may not be pertinent to other continents.

## Conclusions

We aimed to evaluate the effectiveness of champions in improving innovation use and patient, provider and system/facility outcomes in health care settings. In 5 of 7 studies, champions and use of innovations by systems/facilities was positively associated. The effectiveness of champions in improving innovation use by providers and patients, or outcomes was either inconclusive or unexamined. There was little evidence that champions were harmful to implementation. To mitigate the uncertainty related to champions’ effectiveness, their deployment should be accompanied by a plan: (1) on how the use of champions will achieve goals or address barriers to implementation; (2) defining and evaluating fidelity of champion’s activities; and (3) evaluating champions’ effectiveness.

## Supplementary Information

**Additional file 1.** PRISMA 2020 Checklists.

**Additional file 2.** Synthesis Without Meta-analysis (SWIM) in Systematic Reviews Reporting Guideline Checklist.

**Additional file 3.** All Accessed Databases and Peer Review Assessment.

**Additional file 4.** Excluded Articlesand Reasons for Exclusion.

**Additional file 5.** Quality Appraisal Assessments.

## Data Availability

The search strategy, the list of excluded articles, the quality assessment and sensitivity analysis are provided as additional files. The datasets used and/or analysed during the current study are available from the corresponding author on reasonable request.

## Abbreviations

EBP: Evidence-based practice
MESH: Medical subject headings
PRISMA: Preferred Reporting Items for Systematic Reviews and Meta-Analyses
PRESS: Peer Review of the Electronic Search Strategy
RCT: Randomized controlled trial
