# Supplementary material for: The effectiveness of champions in implementing innovations in health care: a systematic review
Source: Implement Sci Commun. 2022 Jul 22;3:80. doi: 10.1186/s43058-022-00315-0 (PMC9308185; doi:10.1186/s43058-022-00315-0)
Supplement: Supplementary file 4 — Additional file 4. Excluded Articlesand Reasons for Exclusion. [file 43058_2022_315_MOESM4_ESM.docx]

**Additional File 4**: Excluded Articles and Reasons for Exclusion

| **Number** | **Reference** | **Exclusion Reason** |
| --- | --- | --- |
| 1 | Aagaard EM, Gonzales R, Camargo Jr CA, Auten R, Levin SK, Maselli J, Metlay J. Physician champions are key to improving antibiotic prescribing quality. The Joint Commission Journal on Quality and Patient Safety. 2010 Mar 1;36(3):109-16. | Study not about effectiveness |
| 2 | Aarons GA, Green AE, Trott E, Willging CE, Torres EM, Ehrhart MG, Roesch SC. The roles of system and organizational leadership in system-wide evidence-based intervention sustainment: a mixed-method study. Administration and Policy in Mental Health and Mental Health Services Research. 2016 Nov;43(6):991-1008. | Not within a health care setting |
| 3 | Aarts M, Conn LG, Govindarajan A, Mccluskey S, McKenzie M, Okrainec A, Pearsall E, Rotstein O, McLeod R. Improved outcomes following implementation of an enhanced recovery after surgery (eras) program across the province of Ontario. Indiseases of the colon & rectum 2016 may 1 (vol. 59, no. 5, pp. E374-e375). Two commerce sq, 2001 market st, Philadelphia, pa 19103 USA: Lippincott Williams & Wilkins. | Conference abstract |
| 4 | Abejirinde IO, Zweekhorst M, Bardají A, Abugnaba-Abanga R, Apentibadek N, De Brouwere V, van Roosmalen J, Marchal B. Unveiling the black box of diagnostic and clinical decision support systems for antenatal care: realist evaluation. JMIR mHealth and uHealth. 2018 Dec 21;6(12):e11468. | Study not about effectiveness |
| 5 | Abendstern M, Hughes J, Tucker S, Clarkson P, Challis D. Self-assessment and personalization in occupational therapy services: a managerial perspective on the challenges and opportunities of a service innovation. British Journal of Occupational Therapy. 2014 Oct;77(10):499-506. | Study not about effectiveness |
| 6 | Abernethy AP, Wheeler JL, Bull J. Development of a health information technology–based data system in community-based hospice and palliative care. American journal of preventive medicine. 2011 May 1;40(5):S217-24. | Study not about effectiveness |
| 7 | Abrahamian Y, Watson H. Strategies for Health System Implementation of Guidelines on Overweight and Obesity. BMJ Qual Saf [Internet]. 2013 Aug 15; 22 (Suppl1): A34. 2-A34. | Conference abstract |
| 8 | Abrahamson V, Jaswal S, Wilson PM. An evaluation of the clinical microsystems approach in general practice quality improvement. Primary health care research & development. 2020;21. | Study not about effectiveness |
| 9 | Abrams MN, Cummings S, Hage D. Clinical care paths a role for finance in clinical decision-making: hospital finance leaders can play an important role in the development of clinical care paths designed to guide clinicians in the delivery of high-quality, cost-effective care, but they should always defer to physicians to lead the effort. Healthcare Financial Management. 2012 Dec 1;66(12):86-92. | Study not about effectiveness |
| 10 | Abuhejleh AA, Dulaimi M, Ellahham S. Using Lean management to leverage innovation in healthcare projects: case study of a public hospital in the UAE. BMJ Innovations. 2016 Feb 3;2(1):22-32. | Not a champion |
| 11 | Acolet D, Allen E, Houston R, Wilkinson AR, Costeloe K, Elbourne D. Improvement in neonatal intensive care unit care: a cluster randomised controlled trial of active dissemination of information. Archives of Disease in Childhood-Fetal and Neonatal Edition. 2011 Nov 1;96(6):F434-9. | Study not about effectiveness |
| 12 | Adams A, Wilson S, Simons R. The Use of Coaches to Enhance the Infection Prevention Program at a Large Teaching Hospital: Presentation Number 8-091. Ajic (american Journal of Infection Control). 2010 Jun;38(5). | Conference abstract |
| 13 | Adams, C. "Creating improvements in perinatal mental health services through the institute of health visiting perinatal mental health champions." Arch Womens Ment Health. 2015. 18(2): 386-387. | Conference abstract |
| 14 | Adelgais KM, Sholl JM, Alter R, Gurley KL, Broadwater-Hollifield C, Taillac P. Challenges in statewide implementation of a prehospital evidence-based guideline: An assessment of barriers and enablers in five states. Prehospital Emergency Care. 2019 Mar 4;23(2):167-78. | Not within a health care setting |
| 15 | Adsul P, Wray R, Gautam K, Jupka K, Weaver N, Wilson K. Becoming a health literate organization: Formative research results from healthcare organizations providing care for undeserved communities. Health services management research. 2017 Nov;30(4):188-96. | Study not about effectiveness |
| 16 | Agarwal G, Angeles RN, Dolovich L, Kaczorowski J, Gaber J, Guenter D, Arnuco FD, Lam HY, Thabane L, O’Reilly D, Agbulos RM. The Community Health Assessment Program in the Philippines (CHAP-P) diabetes health promotion program for low-to middle-income countries: study protocol for a cluster randomized controlled trial. BMC public health. 2019 Dec;19(1):1-2. | Protocol |
| 17 | Agbakoba R, McGee-Lennon M, Bouamrane MM, Watson N, Mair F. Implementing a national Scottish digital health & wellbeing service at scale: a qualitative sudy of stakeholders' views. | Not within a health care setting |
| 18 | Agrell-Kann M. Improving quality outcomes using a champion model for ancillary nursing staff. The Journal of Continuing Education in Nursing. 2015 Dec 1;46(12):539-41. | Study not about effectiveness |
| 19 | Ahamed MF, Campbell D, Horan S, Rosen O. Noise reduction in the neonatal intensive care unit: A quality improvement initiative. American Journal of Medical Quality. 2018 Mar;33(2):177-84. | Study not about effectiveness |
| 20 | Ahern C, McKinnon MC, Bieling PJ, McNeely H, Langstaff K. Overcoming the challenges inherent in conducting design research in mental health settings: lessons from St. Joseph’s healthcare, Hamilton’s pre and post-occupancy evaluation. HERD: Health Environments Research & Design Journal. 2016 Jan;9(2):119-29. | Not a champion |
| 21 | Ahluwalia I, Dean A, Cotter M, Julie G, Hemedez-Gonzalez R, Tait F. Partnering with National Pediatric Associations to promote Tobacco Control in the Philippines. | Conference abstract |
| 22 | Ahroni JH. Developing a wound and skin care program. Journal of Wound Ostomy & Continence Nursing. 2014 Nov 1;41(6):549-55. | Study not about effectiveness |
| 23 | Aitken LM, Hackwood B, Crouch S, Clayton S, West N, Carney D, Jack L. Creating an environment to implement and sustain evidence based practice: A developmental process. Australian Critical Care. 2011 Nov 1;24(4):244-54. | Study not about effectiveness |
| 24 | Akgün KM, Gruenewald DA, Smith D, Wertheimer D, Luhrs C. A National VA Palliative Care Quality Improvement Project for Improving Intensive Care Unit Family Meetings (ICU-FMs). Journal of pain and symptom management. 2019 Dec 1;58(6):1075-80. | Study not about effectiveness |
| 25 | Akhter LS, Monkman JL, Vang G, Pfeiffer J. Improving asthma control through asthma action plans: a quality improvement project at a midwest community clinic. Journal of community health nursing. 2017 Jul 3;34(3):136-46. | Study not about effectiveness |
| 26 | Alagadan, G., Knaus, N., Pulliam, J., Anantiyo, W., Givens, T. Engaging the Inter Disciplinary Team in Critical Thinking and Process Improvements to Improve Peritoneal Dialysis Adequacy. Peritoneal Dialysis International. 2020; 40(IS):2S-18S | Conference abstract |
| 27 | Alaniz VI, Riley M, Smith YR, Vash-Margita A, Lane JC, Patterson V, Quint EH. Improving Adolescent Health Care in an OB/GYN Clinic: The Adolescent Champion Model. Journal of Pediatric and Adolescent Gynecology. 2017 Apr 1;30(2):324-5. | Conference abstract |
| 28 | Alaradi LK. Assessing the impact of healthcare accreditation from the perspective of professionals’ in primary healthcare centres: A mixed methods case study from Kuwait (Doctoral dissertation, University of Glasgow). | Study not about effectiveness |
| 29 | Albany JM, Borton D, Yerkes A, Oni J, Reynolds C, Jagiela S, Zuckerman J. Multidisciplinary collaboration and use of evidence-based bundle reduce surgical site infections associated with hip and knee arthroplasty. American Journal of Infection Control. 2016 Jun 2;44(6):S97. | Conference abstract |
| 30 | Albert NM. They're Watching You!: Performance Measurement, Staffing, and Facilities Requirements. Critical pathways in cardiology. 2006 Mar 1;5(1):18-24. | Study not about effectiveness |
| 31 | Aldiss S, Cass H, Ellis J, Rose L, Gibson F. Benchmarks for Transition: Informing and Supporting Change. InPEDIATRIC BLOOD & CANCER 2016 Nov 1 (Vol. 63, pp. S85-S85). 111 RIVER ST, HOBOKEN 07030-5774, NJ USA: WILEY-BLACKWELL. | Conference abstract |
| 32 | Alfonso AR, Hutzler L, Lajam C, Bosco J, Goldstein J. Institution-wide blood management protocol reduces transfusion rates following spine surgery. International journal of spine surgery. 2019 Jun 1;13(3):270-4. | Study not about effectiveness |
| 33 | Alghafes R. The role of champions in healthcare innovations (Doctoral dissertation, University of Southampton). | Study not about effectiveness |
| 34 | Alia KA. Planning for Healthcare Quality using Procurement as a Strategy: A Case Study Exploring an Outcomes-Oriented Approach to Healthcare Procurement (Doctoral dissertation, University of South Carolina). | Study not about effectiveness |
| 35 | Alidina S, Hur HC, Berry WR, Molina G, Guenthner G, Modest AM, Singer SJ. Narrative feedback from OR personnel about the safety of their surgical practice before and after a surgical safety checklist intervention. International Journal for Quality in Health Care. 2017 Aug 1;29(4):461-9. | Study not about effectiveness |
| 36 | Allen D, Weinhold M, Miller J, Joswiak ME, Bursiek A, Rubin A, O'Hara S, Grubbs P. Nurses as champions for patient safety and interdisciplinary problem solving. Medsurg Nursing. 2015 Mar 1;24(2):107. | Not a champion |
| 37 | Allen KA, Dittmann KR, Hutter JA, Chuang C, Donald ML, Enns AL, Hovanec N, Hunt AW, Kellowan RS, Linkewich EA, Patel AS. Implementing a shared decision‐making and cognitive strategy‐based intervention: Knowledge user perspectives and recommendations. Journal of evaluation in clinical practice. 2020 Apr;26(2):575-81. | Study not about effectiveness |
| 38 | Al-Qirim N. Championing telemedicine adoption and utilization in healthcare organizations in New Zealand. International journal of medical informatics. 2007 Jan 1;76(1):42-54. | Study not about effectiveness |
| 39 | Al-Qirim NA. Teledermatology: the case of adoption and diffusion of telemedicine health Waikato in New Zealand. Telemedicine Journal and e-health. 2003 Jun 1;9(2):167-77. | Study not about effectiveness |
| 40 | AlShimemeri A. How to initiate noninvasive ventilation program in your hospital. Critical Care & Shock. 2013 Nov 1;16(4). | Study not about effectiveness |
| 41 | Altomare A, Eisenberg E, McClure A, Mecchella JN. IMPROVING ADULT PNEUMOCOCCAL VACCINATION COVERAGE IN PRIMARY CARE CLINICS IN NEW HAMPSHIRE: CONTEXT MATTERS. InJOURNAL OF GENERAL INTERNAL MEDICINE 2013 Jun 1 (Vol. 28, pp. S438-S439). 233 SPRING ST, NEW YORK, NY 10013 USA: SPRINGER. | Conference abstract |
| 42 | Alvarado JL, Homaifar N, Nyakudarika N, Autry M, Chen L. Improving Documentation of Medical Proxy and Code Status. Gynecologic Oncology. 2017 Oct 1;147(1):221. | Conference abstract |
| 43 | Alvarez E, Lavis JN, Brouwers M, Schwartz L. Developing a workbook to support the contextualisation of global health systems guidance: a case study identifying steps and critical factors for success in this process at WHO. Health research policy and systems. 2018 Dec;16(1):1-1. | Study not about effectiveness |
| 44 | Amed S, Shea S, Pinkney S, Wharf Higgins J, Naylor PJ. Wayfinding the live 5-2-1-0 initiative—At the intersection between systems thinking and community-based childhood obesity prevention. International journal of environmental research and public health. 2016 Jun;13(6):614. | Not within a health care setting |
| 45 | Amer HA, Amer HA, Alzoman HA, Abdallah H, Jomaa H. Reducing central line associated blood stream infections at intensive care unit. Antimicrobial Resistance and Infection Control. 2015 Dec;4(1):1-. | Conference abstract |
| 46 | An LC, Bluhm J, Foldes S, Kirch MA, Bernhardt T, Center B, Finstad D, Manley M. Clinical system elements and patient reports of tobacco cessation counseling. Journal of Clinical Outcomes Management. 2008 Oct 1;15(10):485-92. | Study not about effectiveness |
| 47 | Anand M., Dong, C., Totev, V., Stewart, L. Medication error minimization project. Australian & New Zealand Journal of Psychiatry. 2018. 52 (S1): 84. | Conference abstract |
| 48 | Anderson C. An investigation of leadership as a factor in quality improvement implementation in United States hospitals. University of La Verne; 2000. | Study not about effectiveness |
| 49 | Anderson J, Kotagal U. Quality in an academic setting. Cincinnati Children's teams execs, physicians, families to improve care systems. Modern healthcare. 2005 Feb 14;35(7):36-8. | Not a champion |
| 50 | Anekwe D, de Marchie M, Spahija J. Mobilizing the Patient in the ICU: Survey of the Barriers in Translating Knowledge to Practice. Chest. 2015 Oct 1;148(4):218A. | Conference abstract |
| 51 | Anekwe DE, Milner SC, Bussières A, de Marchie M, Spahija J. Intensive care unit clinicians identify many barriers to, and facilitators of, early mobilisation: a qualitative study using the Theoretical Domains Framework. Journal of physiotherapy. 2020 Apr 1;66(2):120-7. | Study not about effectiveness |
| 52 | Ang E, Chow YL. General pain assessment among patients with cancer in an acute care setting: a best practice implementation project. International Journal of Evidence‐Based Healthcare. 2010 Jun;8(2):90-6. | Study not about effectiveness |
| 53 | Ang L, Ooi KL, Lim A, Ong B, Tambyah P, Fisher D. Improving hand hygiene compliance in Singapore via innovation. InBMC Proceedings 2011 Dec (Vol. 5, No. 6, pp. 1-1). BioMed Central. | Conference abstract |
| 54 | Angell B, Pares J, Mooney G. Implementing priority setting frameworks: Insights from leading researchers. Health Policy. 2016 Dec 1;120(12):1389-94. | Not a champion |
| 55 | Anglin G, Tu HA, Liao K, Sessums L, Taylor EF. Strengthening multipayer collaboration: lessons from the Comprehensive Primary Care Initiative. The Milbank Quarterly. 2017 Sep;95(3):602-33. | Not a champion |
| 56 | Anne MK. Implementing a Pain Management Program. InWeiner’s Pain Management 2005 Aug 31 (pp. 1509-1516). CRC Press. | Not a champion |
| 57 | Anonymous (2008). "What if physicians don't believe your quality data?" Healthcare benchmarks and quality improvement. 2008. 15(7): 67-69. | Study not about effectiveness |
| 58 | Anonymous. A crash course in Six Sigma. Health Care Strategic Management. 2005; 23 (12): 14 | Not a champion |
| 59 | Anonymous. AGS 2019 Annual Meeting. Journal of the American Geriatrics Society / 2019;67(Supplement 1): 1-384 | Conference abstract |
| 60 | Anonymous. Benchmarking develops buy-in for pathways. Hospital Case Management. 1997 Dec. 172 - 175. | Study not about effectiveness |
| 61 | Anonymous. Clinical pathways: A special report CM cooperation skills help doctors to 'buy in'. Hospital Case Management. 2001. 1 -3 | Not a champion |
| 62 | Anonymous. establishing successful value-based partnerships: a report from HFMA's ANI 2017 Executive Experience session. Healthcare Financial Management 2017;71(10):78-87 | Study not about effectiveness |
| 63 | Anonymous. Health Care, Hospitals; 2nd Annual International Patient Safety Symposium. Hospital & Nursing Home Week. 2011. | Study not about effectiveness |
| 64 | Anonymous. 'Modest' benefits seen with use of hospitalists. 2008. Healthcare Benchmarks and Quality Improvement. 2008. 34-35. | Study not about effectiveness |
| 65 | Anonymous. MS pathway encourages physician buy-in. Hospital Case Management. 1997 Aug; 140. | Study not about effectiveness |
| 66 | Anonymous. New findings underscore value of palliative care consultations ED management : the monthly update on emergency department management. 2014;26(3):27-30 | Study not about effectiveness |
| 67 | Anonymous. NHS trusts must work harder to improve worker wellbeing. Occupational Health. 2013;65(1):5 | Study not about effectiveness |
| 68 | Anonymous. SABM 2018 Abstracts Anesthesia and Analgesia / 2018;127(3 Supplement 2): 1- 78. | Conference abstract |
| 69 | Anonymous. Towards an optimal care pathway for post-myocardial infarction heart failure. Heart. 2005;91(Suppl II):ii43–ii48 | Letters to the editor/ Review studies |
| 70 | Anynomous.A2059:D2061Getting from yes to use with CPGs in evidence-based medicine: multidisciplinary teams with clinician champions are pushing across the last frontier of guideline implementation...clinical practice guidelines Joint Commission Benchmark Dec 2001;3(12):1-10 | No full text available |
| 71 | Apisarnthanarak A, Khawcharoenporn T, Greene MT, Kennedy E, Krein S, Saint S. National survey of Thai infection preventions in the era of patient safety. American journal of infection control. 2013 Apr 1;41(4):362-4. | Study not about effectiveness |
| 72 | Ardery G, Herr K, Hannon BJ, Titler MG. Lack of opioid administration in older hip fracture patients (CE). Geriatric Nursing. 2003 Nov 1;24(6):353-60. | Study not about effectiveness |
| 73 | Ariyabuddhiphongs, K.D., Building an academic primary care-based center for integrative medicine: The beth israel deaconess medical center (BIDMC) experience. Journal of General Internal Medicine / 2012;27(SUPPL. 2):S518 | Conference abstract |
| 74 | Aronson C, Halperin E, Fantus R, Roy L, Barrionuevo M. First Year for Blood Management in a Midwest Hospital System: AP7. Transfusion. 2013 Sep;53. | Conference abstract |
| 75 | Ash JS, Stavri PZ, Dykstra R, Fournier L. Implementing computerized physician order entry: the importance of special people. International journal of medical informatics. 2003 Mar 1;69(2-3):235-50. | Conference abstract |
| 76 | Aslalcson R, Coyle M, Wyskiel R, Copley C, Young K, Ahuja N, Pronovost P. STANDARDIZED INTERDISCIPLINARY FAMILY MEETINGS FOR LONG-STAY SURGICAL INTENSIVE CARE UNIT PATIENTS-RESULTS OF A FEASIBILITY PILOT. InCRITICAL CARE MEDICINE 2010 Dec 1 (Vol. 38, No. 12, pp. U226-U226). 530 WALNUT ST, PHILADELPHIA, PA 19106-3621 USA: LIPPINCOTT WILLIAMS & WILKINS. | Conference abstract |
| 77 | Asselin JD, Osunlana A, Ogunleye A, Sharma AM, Campbell-Scherer D. Mapping the knowledge-to-action framework in a primary care obesity study. Canadian Journal of Diabetes. 2015 Apr 1;39:S23. | Conference abstract |
| 78 | Association of periOperative Registered Nurses. Surgical smoke evacuation: the official voice of perioperative nursing. AORN Journal 2018;108(3):298-302,304-305 | Conference abstract |
| 79 | Asurakkody TA, Shin SY. Innovative behavior in nursing context: a concept analysis. Asian Nursing Research. 2018 Dec 1;12(4):237-44. | Study not about effectiveness |
| 80 | Aupont O. Exploring the relative importance of practice characteristics on adoption of a quality improvement program in primary care: identification of conditions accelerating dissemination of guideline concordant care for depression. Brandeis University, The Heller School for Social Policy and Management; 2001. | Not a champion |
| 81 | Austin L, Ewing G, Grande G. Facilitating a shift to comprehensive carer-led assessment in palliative home care: the CSNAT approach. BMJ Supportive & Palliative Care. 2014 Mar 1;4(1):114-5. | Conference abstract |
| 82 | Axford A, Carter D. Building workforce capacity for ethical reflection in health promotion: a practitioner’s experience. Health Promotion Journal of Australia. 2015 Dec 23;26(3):222-30. | Not about knowledge translation/evidence-based practice |
| 83 | Ayton DR, Barker AL, Morello RT, Brand CA, Talevski J, Landgren FS, Melhem MM, Bian E, Brauer SG, Hill KD, Livingston PM. Barriers and enablers to the implementation of the 6-PACK falls prevention program: A pre-implementation study in hospitals participating in a cluster randomised controlled trial. PloS one. 2017 Feb 16;12(2):e0171932. | Study not about effectiveness |
| 84 | Babiker A, Amer YS, Osman ME, Al‐Eyadhy A, Fatani S, Mohamed S, Alnemri A, Titi MA, Shaikh F, Alswat KA, Wahabi HA. Failure Mode and Effect Analysis (FMEA) may enhance implementation of clinical practice guidelines: An experience from the Middle East. Journal of evaluation in clinical practice. 2018 Feb;24(1):206-11. | Not a champion |
| 85 | Babor TF, Del Boca F, Bray JW. Screening, brief intervention and referral to treatment: implications of SAMHSA's SBIRT initiative for substance abuse policy and practice. Addiction. 2017 Feb;112:110-7. | Study not about effectiveness |
| 86 | Backman C, Hebert PC, Jennings A, Neilipovitz D, Choudhri O, Iyengar A, Rigal R, Forster AJ. Implementation of a multimodal patient safety improvement program “SafetyLEAP” in intensive care units: A cross-case study analysis. International journal of health care quality assurance. 2018 Mar 12. | Study not about effectiveness |
| 87 | Baig AA, Benitez A, Campbell A, Schaefer CT, Heuer LJ, Quinn MT, Burnet DL, Chin MH. " They Said, No, You Can't Bill": Safety Net Clinics' Barriers to Implementing and Sustaining Diabetes Group Visits. InDIABETES 2014 Jun 1 (Vol. 63, pp. A308-A309). 1701 N BEAUREGARD ST, ALEXANDRIA, VA 22311-1717 USA: AMER DIABETES ASSOC. | Conference abstract |
| 88 | Bailey D, Kerlin L. Can health trainers make a difference with difficult-to-engage clients? A multisite case study. Health Promotion Practice. 2015 Sep;16(5):756-64. | Not about knowledge translation/evidence-based practice |
| 89 | Bailey F, Tanqueray T. NELA at Homerton Hospital: creating a new culture in peri-operative care: 156. Anaesthesia. 2016 Jan;71. | Conference abstract |
| 90 | Bailey JE, Surbhi S, Bell PC, Jones AM, Rashed S, Ugwueke MO. SafeMed: using pharmacy technicians in a novel role as community health workers to improve transitions of care. Journal of the American Pharmacists Association. 2016 Jan 1;56(1):73-81. | Study not about effectiveness |
| 91 | Bailey, F.A., Woodby, L., Williams, B., Burgio, K.Formative evaluation of a multi-component, education-based intervention to improve processes of end-of-life care Journal of Pain and Symptom Management / 2014;47(2):512 | Conference abstract |
| 92 | Bakitas M, Allen Watts K, Malone E, Dionne-Odom JN, McCammon S, Taylor R, Tucker R, Elk R. Forging a new frontier: providing palliative care to people with cancer in rural and remote areas. Journal of Clinical Oncology. 2020 Mar 20;38(9):963-73. | Not a champion |
| 93 | Baldwin LM, Keppel GA, Davis A, Guirguis‐Blake J, Force RW, Berg AO. Developing a practice‐based research network by integrating quality improvement: challenges and ingredients for success. Clinical and translational science. 2012 Aug;5(4):351-5. | Study not about effectiveness |
| 94 | Ballard C, Corbett A, Orrell M, Williams G, Moniz-Cook E, Romeo R, Woods B, Garrod L, Testad I, Woodward-Carlton B, Wenborn J. Impact of person-centred care training and person-centred activities on quality of life, agitation, and antipsychotic use in people with dementia living in nursing homes: A cluster-randomised controlled trial. PLoS medicine. 2018 Feb 6;15(2):e1002500. | Study not about effectiveness |
| 95 | Ballard C, Orrell M, Moniz-Cook E, Woods R, Whitaker R, Corbett A, Aarsland D, Murray J, Lawrence V, Testad I, Knapp M. Improving mental health and reducing antipsychotic use in people with dementia in care homes: the WHELD research programme including two RCTs. | Conference abstract |
| 96 | Ballard DJ, Nicewander DA, Qin H, Fullerton C, Winter Jr FD, Couch CE. Improving delivery of clinical preventive services: a multi-year journey. American journal of preventive medicine. 2007 Dec 1;33(6):492-7. | Study not about effectiveness |
| 97 | Banavage AJ, Lugar TM. A Novel Approach to Falls Reduction in the BMTU. Biology of Blood and Marrow Transplantation. 2011 Feb 1;17(2):S367-8. | Conference abstract |
| 98 | Bansod VA. Promoting action on research implementation in health services (PARIHS) framework: Application to the fracture fighters program. University of Toronto; 2009 Dec 16. | Letters to the editor/ Review studies |
| 99 | Barchitta M, Quattrocchi A, Maugeri A, La Rosa MC, La Mastra C, Basile G, Giuffrida G, Mazzeo Rinaldi F, Murolo G, Agodi A. The “Obiettivo Antibiotico” Campaign on Prudent Use of Antibiotics in Sicily, Italy: The Pilot Phase. International journal of environmental research and public health. 2020 Jan;17(9):3077. | Not a champion |
| 100 | Bardenheier BH, Lindley MC, Ball SW, de Perio MA, Laney S, Gravenstein S. Cluster Analysis: Vaccination Attitudes and Beliefs of Healthcare Personnel. American journal of health behavior. 2020 May 1;44(3):302-12. | Study not about effectiveness |
| 101 | Bardosh KL, Murray M, Khaemba AM, Smillie K, Lester R. Operationalizing mHealth to improve patient care: a qualitative implementation science evaluation of the WelTel texting intervention in Canada and Kenya. Globalization and health. 2017 Dec;13(1):1-5. | Not a champion |
| 102 | Barker SG, Sachs R, Louden C, Linnard D, Abu-Own A, Buckland J, Murphy S. Integrated care pathways for vascular surgery. European journal of vascular and endovascular surgery. 1999 Sep 1;18(3):207-15. | Study not about effectiveness |
| 103 | Barlow R.C., Willson A., Baker M. The all wales enhanced recovery after surgery programme. Clinical Nutrition, Supplement / 2011;6(1):138 | Conference abstract |
| 104 | Barnes S. Surgical site infection prevention in 2018 and beyond. AORN journal. 2018 May;107(5):547-50. | Not a champion |
| 105 | Barnett J, Vasileiou K, Djemil F, Brooks L, Young T. Understanding innovators' experiences of barriers and facilitators in implementation and diffusion of healthcare service innovations: a qualitative study. BMC Health Services Research. 2011 Dec;11(1):1-2. | Study not about effectiveness |
| 106 | Barnett S, Jones SC, Caton T, Iverson D, Bennett S, Robinson L, Robinson L. Implementing a Virtual Community of Practice for Family Physician Training: A Case Study. Research Online.:118. | Study not about effectiveness |
| 107 | Barrett JS. Paediatric models in motion: requirements for model‐based decision support at the bedside. British journal of clinical pharmacology. 2015 Jan;79(1):85-96. | Study not about effectiveness |
| 108 | Barrette R, Wehlage M, Albrecht A. TAKING THE PRESSURE OFF: DECREASING THE RISK OF MEDICAL DEVICE-RELATED PRESSURE INJURIES IN CHILDREN. InJOURNAL OF WOUND OSTOMY AND CONTINENCE NURSING 2020 May 1 (Vol. 47, pp. S50-S51). TWO COMMERCE SQ, 2001 MARKET ST, PHILADELPHIA, PA 19103 USA: LIPPINCOTT WILLIAMS & WILKINS. | Conference abstract |
| 109 | Barry L. Building and maintaining a statewide clinical network: the Queensland Child Development Sub-Network 2009-2017. International Journal of Integrated Care (IJIC). 2018 Apr 2;18. | Conference abstract |
| 110 | Baryashaba A, Musimenta A, Mugisha S, Binamungu LP. Investigating the adoption of an integrated hospital information system in rural Uganda: A case of Kisiizi Hospital. InInternational Conference on Social Implications of Computers in Developing Countries 2019 May 1 (pp. 316-325). Springer, Cham. | Not a champion |
| 111 | Basaza R, Kinegyere A, Mutatina B, Sewankambo N. National framework for the sustainability of health knowledge translation initiatives in Uganda. International journal of technology assessment in health care. 2018;34(1):120-8. | Study not about effectiveness |
| 112 | Basnett J, Eapen V, Asghari M. Quality improvement intervention in monitoring rate of cardiometabolic risk factors in a community mental health centre. InAUSTRALIAN AND NEW ZEALAND JOURNAL OF PSYCHIATRY 2019 Apr 1 (Vol. 53, pp. 140-140). 1 OLIVERS YARD, 55 CITY ROAD, LONDON EC1Y 1SP, ENGLAND: SAGE PUBLICATIONS LTD. | Conference abstract |
| 113 | Basrai Z., Celedon M., Preston-Suni K.,Hsiao J.J., Graber C., Goetz M. Decreasing antimicrobial prescribing via an audit and feedback with peer-to-peer antimicrobial stewardship program. Academic Emergency Medicine / 2020;27(Supplement 1):S263-S264 | Conference abstract |
| 114 | Bastin K, Akers C, Glazebrook B, Bielby L, Beard P, Daly J. How the blood champion role is contributing to patient safety in Victoria. InINTERNAL MEDICINE JOURNAL 2019 Dec 1 (Vol. 49, pp. 19-19). 111 RIVER ST, HOBOKEN 07030-5774, NJ USA: WILEY. | Conference abstract |
| 115 | Battista RN, Williams JI, Boucher J, Rosenberg E, Stachenko SJ, Adam J, Levinton C, Suissa S. Testing various methods of introducing health charts into medical records in family medicine units. CMAJ: Canadian Medical Association Journal. 1991 Jun 1;144(11):1469. | Study not about effectiveness |
| 116 | Battistelli MF, Magnusson S, Biggs MA, Freedman L. Expanding the abortion provider workforce: a qualitative study of organizations implementing a new California policy. Perspectives on sexual and Reproductive Health. 2018 Mar;50(1):33-9. | Study not about effectiveness |
| 117 | Baxter PE, Hewko SJ, Pfaff KA, Cleghorn L, Cunningham BJ, Elston D, Cummings GG. Leaders’ experiences and perceptions implementing activity-based funding and pay-for-performance hospital funding models: A systematic review. Health policy. 2015 Aug 1;119(8):1096-110. | Letters to the editor/ Review studies |
| 118 | Bayley M, Mayo NE, Richards C, Eng J, Wood-Dauphinee S. Stroke Canada optimization of rehabilitation by evidence (score): detailed analysis of the effect on outcomes of practice by guidelines vs. practice by outcomes, a clustered randomized trial. InStroke 2012 Nov 1 (Vol. 43, No. 11, pp. E119-E120). 530 WALNUT ST, PHILADELPHIA, PA 19106-3621 USA: LIPPINCOTT WILLIAMS & WILKINS. | Conference abstract |
| 119 | Beal J. Occupational therapy lead the journey to smoke free living. InBRITISH JOURNAL OF OCCUPATIONAL THERAPY 2016 Aug 1 (Vol. 79, pp. 105-105). 1 OLIVERS YARD, 55 CITY ROAD, LONDON EC1Y 1SP, ENGLAND: SAGE PUBLICATIONS LTD. | Conference abstract |
| 120 | Beaney P, Hatfield R, Hughes A, Schmid M, Chambers R. Creating digitally ready nurses in general practice. Nursing Management. 2019 May 28;26(3). | Study not about effectiveness |
| 121 | Bearman G, Stevens MP. Pushing beyond resistors and constipators: implementation considerations for infection prevention best practices. Current infectious disease reports. 2014 Jan 1;16(1):388. | Study not about effectiveness |
| 122 | Beath CM. Supporting the information technology champion. MIS quarterly. 1991 Sep 1:355-72. | Not within a health care setting |
| 123 | Beaulieu RJ, Albright J, Jeruzal E, Mansour MA, Aziz A, Mouawad NJ, Osborne NH, Henke PK. A statewide quality collaborative significantly improves quality metric adherence and physician engagement in vascular surgery. Journal of Vascular Surgery. 2021 Sep 3. | Conference abstract |
| 124 | Becker S, Hagle M, Amrhein A, Bispo J, Hopkins S, Kogelmann M, Porras E, Smith MM. Implementing and Sustaining Bedside Shift Report for Quality Patient-Centered Care. Journal of Nursing Care Quality. 2021 Apr 1;36(2):125-31. | Study not about effectiveness |
| 125 | Becker SJ, Kelly LM, Kang AW, Escobar KI, Squires DD. Factors associated with contingency management adoption among opioid treatment providers receiving a comprehensive implementation strategy. Substance abuse. 2019 Jan 2;40(1):56-60. | Study not about effectiveness |
| 126 | Becker WC, Mattocks KM, Frank JW, Bair MJ, Jankowski RL, Kerns RD, Painter JT, Fenton BT, Midboe AM, Martino S. Mixed methods formative evaluation of a collaborative care program to decrease risky opioid prescribing and increase non-pharmacologic approaches to pain management. Addictive behaviors. 2018 Nov 1;86:138-45. | Study not about effectiveness |
| 127 | Beckham, J.D. New Product Development. Hospital Forum 1985;28(4):59 | Not about knowledge translation/evidence-based practice |
| 128 | Beckman D, Wardian J, Sauerwein TJ, True MW. Evaluation of an interprofessional continuing professional development course on comprehensive diabetes care: A mixed‐methods approach. Journal of evaluation in clinical practice. 2019 Feb;25(1):148-54. | Not a champion |
| 129 | Beddingfield H., Sutton K., Kim E., Batara D., Hirsch K. The role for neuro champions in a mixed intensive care unit. Neurocritical Care / 2017;27(2 Supplement 1):S304 | Conference abstract |
| 130 | Bee P, Lovell K, Airnes Z, Pruszynska A. Embedding telephone therapy in statutory mental health services: a qualitative, theory-driven analysis. BMC psychiatry. 2016 Dec;16(1):1-1. | Study not about effectiveness |
| 131 | Beffa A, Farr A. Unit practice council champions support clinical nurses to improve outcomes. Nursing management. 2019 Apr 1;50(4):17-8. | Study not about effectiveness |
| 132 | Begum R, Liu J, Sun C. Always InforMED: Nurse champion-led intervention to improve medication communication among nurses and patients. Applied nursing research: ANR. 2020 Jun;53:151264. | Study not about effectiveness |
| 133 | Behr C, Mercier C, Schriefer J. Strategies for involving physicians in continuous quality improvement. Journal for Healthcare Quality. 1996 Jul 8;18(4):21-3. | Not a champion |
| 134 | Beidas RS, Paciotti B, Barg F, Branas AR, Brown JC, Glanz K, DeMichele A, DiGiovanni L, Salvatore D, Schmitz KH. A hybrid effectiveness-implementation trial of an evidence-based exercise intervention for breast cancer survivors. Journal of the National Cancer Institute Monographs. 2014 Nov 1;2014(50):338-45. | Study not about effectiveness |
| 135 | Belden CM, Proeschold-Bell RJ. A comparison of the adoption of electronic health records in North Carolina and South Carolina HIV systems. Southern medical journal. 2010 Nov 1;103(11):1115-8. | Study not about effectiveness |
| 136 | Bell MM, Alaestante G, Finch C. A multidisciplinary intervention to prevent catheter-associated urinary tract infections using education, continuum of care, and systemwide buy-in. Ochsner Journal. 2016 Mar 20;16(1):96-100. | Study not about effectiveness |
| 137 | Belnap TW, Sause WT, Rowley BD, Jones C, Ruckdeschel JC. The role, use, and nuances of clinical data systems in oncology quality improvement. | Conference abstract |
| 138 | Belton PR, Roughton SE. The ideal compliance world: integrating physicians into the compliance program. QRC advisor. 1999 Dec 1;16(2):4-10. | Study not about effectiveness |
| 139 | Benard VB, Saraiya M, Greek A, Hawkins NA, Roland KB, Manninen D, Ekwueme DU, Miller JW, Unger ER. Overview of the CDC Cervical Cancer (Cx3) Study: an educational intervention of HPV testing for cervical cancer screening. Journal of Women's Health. 2014 Mar 1;23(3):197-203. | Not a champion |
| 140 | Bendito Barber C, Calvo Rubio García F, Cerdà Adrian J, Donoso Pedrero A, Martínez Pallí G. App “Guía Terapia Intravenosa”: evidencia a pie de cama. Revista Rol de Enfermería. 2020;43(4):298-302. | No full text available |
| 141 | Beneciuk JM, George SZ, Greco CM, Schneider MJ, Wegener ST, Saper RB, Delitto A. Targeted interventions to prevent transitioning from acute to chronic low back pain in high-risk patients: development and delivery of a pragmatic training course of psychologically informed physical therapy for the TARGET trial. Trials. 2019 Dec;20(1):1-4. | Study not about effectiveness |
| 142 | Bennett SF. The relationship between barcode medication administration satisfaction and the use of workarounds among registered nurses. Duquesne University; 2012. | Study not about effectiveness |
| 143 | Bennett-Levy J, Singer J, DuBois S, Hyde K. Translating e-mental health into practice: what are the barriers and enablers to e-mental health implementation by Aboriginal and Torres Strait Islander health professionals?. Journal of medical Internet research. 2017;19(1):e1. | Study not about effectiveness |
| 144 | Bennetts S, Campbell‐Brophy E, Huckson S, Doherty S, National Health and Medical Research Council's National Institute for Clinical Studies National Emergency Care Pain Management Initiative. Pain management in Australian emergency departments: current practice, enablers, barriers and future directions. Emergency Medicine Australasia. 2012 Apr;24(2):136-43. | Not a champion |
| 145 | Benoit B, Semenic S. Barriers and facilitators to implementing the baby-friendly hospital initiative in neonatal intensive care units. Journal of Obstetric, Gynecologic & Neonatal Nursing. 2014 Sep 1;43(5):614-24. | Not a champion |
| 146 | Bensadon B, Rojido M, Wolf D, Shutes J, Tappen R, Ouslander J. Barriers & Approaches to Implementing the INTERACT Quality Improvement Program: B126. Journal of the American Geriatrics Society. 2014 Mar;62. | Conference abstract |
| 147 | Berge, L. The Effect of a Multi-faceted Educational Intervention upon Staff Nurses' Knowledge Related to Adherence to a No-Lift Policy. Catholic University of America. 2017 | Not a champion |
| 148 | Bergh AM, Charpak N, Ezeonodo A, Udani RH, Van Rooyen E. Education and training in the implementation of kangaroo mother care. South African Journal of Child Health. 2012;6(2):38-45. | Study not about effectiveness |
| 149 | Bergh AM, de Graft-Johnson J, Khadka N, Om’Iniabohs A, Udani R, Pratomo H, De Leon-Mendoza S. The three waves in implementation of facility-based kangaroo mother care: a multi-country case study from Asia. BMC international health and human rights. 2016 Dec;16(1):1-3. | Study not about effectiveness |
| 150 | Bergh AM, Kerber K, Abwao S, Johnson JD, Aliganyira P, Davy K, Gamache N, Kante M, Ligowe R, Luhanga R, Mukarugwiro B. Implementing facility-based kangaroo mother care services: lessons from a multi-country study in Africa. BMC health services research. 2014 Dec;14(1):1-0. | Study not about effectiveness |
| 151 | Bergquist-Beringer S, Derganc K, Dunton N. Embracing the use of skin care champions. Nursing management. 2009 Dec 1;40(12):19-24. | Study not about effectiveness |
| 152 | Berlan E, Valenti O, Long W, Abenaim A, Maciejewski H, Toth C, Gowda C. 12. Practice Facilitation Improves Adolescent Reproductive Health Preventive Services in Pediatric Primary Care. Journal of Pediatric and Adolescent Gynecology. 2020 Apr 1;33(2):182. | Conference abstract |
| 153 | Bernstein E, Topp D, Shaw E, Girard C, Pressman K, Woolcock E, Bernstein J. A preliminary report of knowledge translation: lessons from taking screening and brief intervention techniques from the research setting into regional systems of care. Academic emergency medicine. 2009 Nov;16(11):1225-33. | Study not about effectiveness |
| 154 | Bernstein J, Gebel C, Vargas C, Geltman P, Walter A, Garcia R, Tinanoff N. Integration of Oral Health into the Well-Child Visit at Federally Qualified Health Centers: Study of 6 Clinics. Prev Chronic Disease. 2016;12:E58. | Study not about effectiveness |
| 155 | Best A, Berland A, Herbert C, Bitz J, van Dijk MW, Krause C, Cochrane D, Noel K, Marsden J, McKeown S, Millar J. Using systems thinking to support clinical system transformation. Journal of health organization and management. 2016 May 16. | Study not about effectiveness |
| 156 | Beukelman T, Bingham CA, Gottlieb BS, Griffin N, Laxer R, Marsolo K, Passo MH, Lannon C, Margolis P, DeWitt EM. AB1207 Increasing quality of JIA treatment: The pediatric rheumatology care and outcomes improvement network (PR-COIN). Annals of the Rheumatic Diseases. 2013 Jun 1;71(Suppl 3):706-. | Conference abstract |
| 157 | Bhangu N, Shields O, Pryde K. G407 (P) Improving management of prolonged seizures in children. | Conference abstract |
| 158 | Bialek R, Carden J, Duffy GL. Supporting public health departments' quality improvement initiatives: Lessons learned from the Public Health Foundation. Journal of Public Health Management and Practice. 2010 Jan 1;16(1):14-8. | Study not about effectiveness |
| 159 | Bidwell, P. Perineal trauma: the power of partnership. Midwives 2018;21():46-47 | Study not about effectiveness |
| 160 | Bielby L., Flores C., Akers C., Van Diemen J., Glazebrook B., Beard P., Hogan C. 15 years experience of a collaborative program to improve blood management practices in australia. Vox Sanguinis 2017;112(Supplement 2):158-159 | Conference abstract |
| 161 | Bielby L.J., Akers C., Francis S., Darby S., Campbell L., Hollis L., Quested B., Hogan C. The role of the transfusion practitioner in Australia. Vox Sanguinis 2015;109(SUPPL. 1):8- | Conference abstract |
| 162 | Bikson K, Lorenz K, Milch M. Organizational Challenges to Implementing Hospital-Based Palliative Care. Journal of Pain and Symptom Management. 2010 Feb 1;39(2):386-7. | Conference abstract |
| 163 | Bikson KL. Understanding innovation in end-of-life care: A comparative case study of hospital-based palliative care programs. University of California, Los Angeles; 2007. | Letters to the editor/ Review studies |
| 164 | Billings JA, Keeley A, Cist A, Coakley E, Dahlin C, Montgomery P, Thompson BT. Merging cultures: Palliative care specialists in the medical intensive care unit. Critical care medicine. 2006 Nov 1;34(11):S388-93. | Study not about effectiveness |
| 165 | Binns W (2002). Champions kickstart best practice guidelines. Registered Nurse Journal 2002;14(4):20-21 | Study not about effectiveness |
| 166 | Birbeck. Clinical pharmacists supporting inpatient diabetes patients...15th National Conference of the Primary Care Diabetes Society, November 7–8, 2019, Birmingham, England. Diabetes & Primary Care 2019;21(5):180-180 | Conference abstract |
| 167 | Birdas TJ, Hazen D, Kelley K, Waters JA, Schmidt CM. Innovative Method Engages Providers across Two Academic Health Institutions in an Expedient ERAS Deployment. Journal of the American College of Surgeons. 2020 Oct 1;231(4):e152. | Conference abstract |
| 168 | Birken SA, Clary AS, Bernstein S, Bolton J, Tardif-Douglin M, Mayer DK, Deal AM, Jacobs SR. Strategies for successful survivorship care plan implementation: Results from a qualitative study. Journal of oncology practice. 2018 Aug;14(8):e462-83. | Study not about effectiveness |
| 169 | Birken SA, Deal AM, Mayer DK, Weiner BJ. Determinants of survivorship care plan use in US cancer programs. Journal of Cancer Education. 2014 Dec;29(4):720-7. | Study not about effectiveness |
| 170 | Bischoff K, Goel A, Hollander H, Ranji SR, Mourad M. The Housestaff Incentive Program: improving the timeliness and quality of discharge summaries by engaging residents in quality improvement. BMJ quality & safety. 2013 Sep 1;22(9):768-74. | Study not about effectiveness |
| 171 | Blaber B., Woltz P., Bautistia M.A. Bedside research nurse champions: Enhancing research knowledge and multidisciplinary research culture. Neurocritical Care 2013;19(1 SUPPL. 1):S305 | Conference abstract |
| 172 | Blackman T, Wistow J, Byrne D. A qualitative comparative analysis of factors associated with trends in narrowing health inequalities in England. Social Science & Medicine. 2011 Jun 1;72(12):1965-74. | Study not about effectiveness |
| 173 | Blackstock OJ, Moore BA, Berkenblit GV, Calabrese SK, Cunningham CO, Fiellin DA, Patel VV, Phillips KA, Tetrault JM, Shah M, Edelman EJ. A cross-sectional online survey of HIV pre-exposure prophylaxis adoption among primary care physicians. Journal of general internal medicine. 2017 Jan;32(1):62-70. | Study not about effectiveness |
| 174 | Blankenship JS, Denby AS. Empowering UAP to champion pressure ulcer prevention. Nursing2021. 2010 Aug 1;40(8):12-3. | Study not about effectiveness |
| 175 | Blaum CS, Rosen J, Naik AD, Smith CD, Dindo L, Vo L, Hernandez‐Bigos K, Esterson J, Geda M, Ferris R, Costello D. Feasibility of implementing patient priorities care for older adults with multiple chronic conditions. Journal of the American Geriatrics Society. 2018 Oct;66(10):2009-16. | Study not about effectiveness |
| 176 | Blaza J, Wiley J, Lehmann W, Stearns J, Simpson D. Aurora Health Care, Milwaukee, WI Disparities in Colorectal Cancer Screening. Ochsner Journal. 2018 Mar 20;18(S1):10-1. | Study not about effectiveness |
| 177 | Boast G, Green J, Chambers R, Calderwood R. Improving assessment and management of lower limb wounds. Journal of Community Nursing. 2019 Oct 1;33(5). | Study not about effectiveness |
| 178 | Boehmer KR, Holland DE, Vanderboom CE. Identifying and addressing gaps in the implementation of a community care team for care of Patients with multiple chronic conditions. BMC health services research. 2019 Dec;19(1):1-8. | Study not about effectiveness |
| 179 | Bogan C, Jennings L, Haynes L, Barth K, Moreland A, Oros M, Goldsby S, Lane S, Funcell C, Brady K. Implementation of emergency department–initiated buprenorphine for opioid use disorder in a rural southern state. Journal of substance abuse treatment. 2020 Mar 1;112:73-8. | Not a champion |
| 180 | Boland C, Mullaney K. Transport Workers as Champions in Improvement Process:“The Fanny Pack Project”. Ajic (american Journal of Infection Control). 2009 Jun;37(5). | Conference abstract |
| 181 | Boltz M, Resnick B, Chippendale T, Galvin J. Testing a family‐centered intervention to promote functional and cognitive recovery in hospitalized older adults. Journal of the American Geriatrics Society. 2014 Dec;62(12):2398-407. | Study not about effectiveness |
| 182 | Bonawitz K, Wetmore M, Heisler M, Dalton VK, Damschroder LJ, Forman J, Allan KR, Moniz MH. Champions in context: which attributes matter for change efforts in healthcare?. Implementation Science. 2020 Dec;15(1):1-0. | Study not about effectiveness |
| 183 | Bonevski B, Guillaumier A, Shakeshaft A, Farrell M, Tzelepis F, Walsberger S, D’Este C, Paul C, Dunlop A, Searles A, Kelly P. An organisational change intervention for increasing the delivery of smoking cessation support in addiction treatment centres: study protocol for a randomized controlled trial. Trials. 2016 Dec;17(1):1-9. | Study not about effectiveness |
| 184 | Bonner A, MacCulloch P, Gardner T, Chase CW. A student-led demonstration project on fall prevention in a long-term care facility. Geriatric Nursing. 2007 Sep 1;28(5):312-8. | Study not about effectiveness |
| 185 | Boon HS, Kachan N. Integrative medicine: a tale of two clinics. BMC Complementary and Alternative Medicine. 2008 Dec;8(1):1-8. | Study not about effectiveness |
| 186 | Borlaug G, Edmiston Jr CE. Implementation of a Wisconsin Division of Public Health surgical site infection prevention champion initiative. AORN journal. 2018 May;107(5):570-8. | Duplicate |
| 187 | Borlaug G, Edmiston Jr CE. Implementation of a Wisconsin Division of Public Health surgical site infection prevention champion initiative. AORN journal. 2018 May;107(5):570-8. | Study not about effectiveness |
| 188 | Botje D, Ten Asbroek G, Plochg T, Anema H, Kringos DS, Fischer C, Wagner C, Klazinga NS. Are performance indicators used for hospital quality management: a qualitative interview study amongst health professionals and quality managers in The Netherlands. BMC health services research. 2016 Dec;16(1):1-9. | Study not about effectiveness |
| 189 | Bottari C, Kairy D, Lam P, Shun W, Ouellet C, Magnan C, Poissant L, Dawson D, Swaine B. Using a participatory action research approach to facilitate the implementation of the IADL profile in clinical practice. InBRAIN INJURY 2016 Jan 1 (Vol. 30, No. 5-6, pp. 593-593). 530 WALNUT STREET, STE 850, PHILADELPHIA, PA 19106 USA: TAYLOR & FRANCIS INC. | Conference abstract |
| 190 | Bottari, C., Kairy, D., Shun, P.L.-W., Ouellet, C., Magnan C., Poissant L.; Dawson D., Swaine B. Using a participatory action research approach to facilitate the implementation of the instrumental activities of daily living profile in clinical practice. Brain Impairment 2018;19(3):297-298 | Conference abstract |
| 191 | Boudreaux AM, Vetter TR. The creation and impact of a dedicated section on quality and patient safety in a clinical academic department. Academic Medicine. 2013 Feb 1;88(2):173-8. | Study not about effectiveness |
| 192 | Bouk M, Mutterer M, Schore M, Alper P. Use of an electronic hand hygiene compliance system to improve hand hygiene, reduce MRSA, and improve financial performance. American Journal of Infection Control. 2016 Jun 2;44(6):S100-1. | Conference abstract |
| 193 | Bovee M, Robicsek A, Semerdjian N, Langwell D, Wright MO. Implementation of a Process Surveillance Program in a Hospital System. American Journal of Infection Control. 2011 Jun 1;39(5):E85-6. | Conference abstract |
| 194 | Bowie P, Ferguson J, MacLeod M, Kennedy S, de Wet C, McNab D, Kelly M, McKay J, Atkinson S. Participatory design of a preliminary safety checklist for general practice. British Journal of General Practice. 2015 May 1;65(634):e330-43. | Study not about effectiveness |
| 195 | Bowley DM, Lamb D, Rumbold P, Hunt P, Kayani J, Sukhera AM. Nursing and medical contribution to defence healthcare engagement: initial experiences of the UK defence medical services. BMJ Military Health. 2019 Jun 1;165(3):143-6. | Study not about effectiveness |
| 196 | Bowman C, Luck J, Gale RC, Smith N, York LS, Asch S. A qualitative evaluation of web-based cancer care quality improvement toolkit use in the Veterans Health Administration. Quality management in health care. 2015 Jul 1;24(3):147-61. | Study not about effectiveness |
| 197 | Box TL. Factors associated with the implementation and full use of a nationwide health information technology system (Doctoral dissertation, University of Colorado at Denver, Anschutz Medical Campus). | Study not about effectiveness |
| 198 | Boyd L, Hastrup F. 822: USING PHYSICIAN CHAMPIONS TO COORDINATE SPONTANEOUS AWAKENING AND BREATHING TRIALS. Critical Care Medicine. 2015 Dec 1;43(12):207. | Conference abstract |
| 199 | Boyle F., Andrews K., Shaw J. No title received. Supportive Care in Cancer 2016;24(1 Supplement 1):S31-S32 | Conference abstract |
| 200 | Braddock CH, Szaflarski N, Forsey L, Abel L, Hernandez-Boussard T, Morton J. The TRANSFORM Patient safety project: a microsystem approach to improving outcomes on inpatient units. Journal of general internal medicine. 2015 Apr 1;30(4):425-33. | Study not about effectiveness |
| 201 | Bradley EH, Curry LA, Spatz ES, Herrin J, Cherlin EJ, Curtis J, Thompson JW, Ting HH, Wang Y, Krumholz HM. Effective Hospital Strategies for Reducing Risk-Standardized Mortality Rates in Acute Myocardial Infarction. | Conference abstract |
| 202 | Bradley EH, Nembhard IM, Yuan CT, Stern AF, Curtis JP, Nallamothu BK, Brush Jr JE, Krumholz HM. What is the experience of national quality campaigns? Views from the field. Health services research. 2010 Dec;45(6p1):1651-69. | Study not about effectiveness |
| 203 | Bradley K, Smith R, Hughson JA, Atkinson D, Bessarab D, Flicker L, Radford K, Smith K, Strivens E, Thompson S, Blackberry I. Let’s CHAT (community health approaches to) dementia in Aboriginal and Torres Strait Islander communities: protocol for a stepped wedge cluster randomised controlled trial. BMC health services research. 2020 Dec;20(1):1-3. | Protocol |
| 204 | Branagan LG. An exploratory investigation of physician champions for hospital-based information technology implementations. Alliant International University, San Francisco Bay; 2010. | Letters to the editor/ Review studies |
| 205 | Bratzler DW, Bumpus LJ, Shook C, McCargar PA, Ruane TJ, Stalhandske EJ, Albrich MJ, Medak RE, Pathak J, Shine ME, Cochran B. Improving care for acute myocardial infarction: experience from the Cooperative Cardiovascular Project. The Joint Commission Journal on Quality Improvement. 1998 Sep 1;24(9):480-90. | Study not about effectiveness |
| 206 | Breitenstein SM, Laurent S, Pabalan L, Risser HJ, Roper P, Saba MT, Schoeny M. Implementation findings from an effectiveness-implementation trial of tablet-based parent training in pediatric primary care. Families, Systems, & Health. 2019 Dec;37(4):282. | Study not about effectiveness |
| 207 | Brennan RA, Callaway S. Innovations in practice: supporting the breastfeeding dyad in Labor & Delivery. Journal of Obstetric, Gynecologic & Neonatal Nursing. 2014 Jun 1;43:S62-3. | Conference abstract |
| 208 | Brewer SE, Barnard J, Pyrzanowski J, O'Leary ST, Dempsey AF. Use of electronic health records to improve maternal vaccination. Women's Health Issues. 2019 Jul 1;29(4):341-8. | Study not about effectiveness |
| 209 | Brieger D. Optimising acute care and secondary prevention for patients with acute coronary syndrome. Medical Journal of Australia. 2014 Nov;201(S10):S88-90. | Study not about effectiveness |
| 210 | Briggs AM, Page CJ, Shaw BR, Bendrups A, Philip K, Cary B, Choong PF. A model of care for osteoarthritis of the hip and knee: development of a system-wide plan for the health sector in Victoria, Australia. Healthcare Policy. 2018 Nov;14(2):47. | Duplicate |
| 211 | Briggs AM, Page CJ, Shaw BR, Bendrups A, Philip K, Cary B, Choong PF. A model of care for osteoarthritis of the hip and knee: development of a system-wide plan for the health sector in Victoria, Australia. Healthcare Policy. 2018 Nov;14(2):47. | Study not about effectiveness |
| 212 | Briggs AM, Towler SC, Speerin R, March LM. Models of care for musculoskeletal health in Australia: now more than ever to drive evidence into health policy and practice. Australian Health Review. 2014 Aug 4;38(4):401-5. | Study not about effectiveness |
| 213 | Brooks E, Manson SM, Bair B, Dailey N, Shore JH. The diffusion of telehealth in rural American Indian communities: a retrospective survey of key stakeholders. Telemedicine and e-Health. 2012 Jan 1;18(1):60-6. | Study not about effectiveness |
| 214 | Brooks Holliday S, Hepner KA, Farmer CM, Ivany C, Iyiewuare P, McGee-Vincent P, McCaslin S, Rosen CS. A qualitative evaluation of Veterans Health Administration’s implementation of measurement-based care in behavioral health. Psychological services. 2020 Aug;17(3):271. | Study not about effectiveness |
| 215 | Brown AH, Cohen AN, Chinman MJ, Kessler C, Young AS. EQUIP: implementing chronic care principles and applying formative evaluation methods to improve care for schizophrenia: QUERI Series. Implementation Science. 2008 Dec;3(1):1-2. | Study not about effectiveness |
| 216 | Brown BB, Haines M, Middleton S, Paul C, D’Este C, Klineberg E, Elliott E. Development and validation of a survey to measure features of clinical networks. BMC health services research. 2016 Dec;16(1):1-1. | Study not about effectiveness |
| 217 | Brown VB, Harris M, Fallot R. Moving toward trauma-informed practice in addiction treatment: A collaborative model of agency assessment. Journal of Psychoactive Drugs. 2013 Nov 1;45(5):386-93. | Study not about effectiveness |
| 218 | Brown-Johnson C, Haverfield MC, Giannitrapani KF, Lo N, Lowery JS, Foglia MB, Walling AM, Bekelman DB, Shreve ST, Lehmann LS, Lorenz KA. Implementing goals-of-care conversations: lessons from high-and low-performing sites from a VA national initiative. Journal of Pain and Symptom Management. 2021 Feb 1;61(2):262-9. | Study not about effectiveness |
| 219 | Bruce R, Forry C. Integrating a mobility champion in the intensive care unit. Dimensions of Critical Care Nursing. 2018 Jul 1;37(4):201-9. | Study not about effectiveness |
| 220 | Brunkert T, Ruppen W, Simon M, Zúñiga F. A theory‐based hybrid II implementation intervention to improve pain management in Swiss nursing homes: A mixed‐methods study protocol. Journal of advanced nursing. 2019 Feb;75(2):432-42. | Protocol |
| 221 | Brunkert T, Simon M, Ruppen W, Zúñiga F. Pain Management in Nursing Home Residents: Findings from a Pilot Effectiveness‐Implementation Study. Journal of the American Geriatrics Society. 2019 Dec;67(12):2574-80. | Study not about effectiveness |
| 222 | Bryce C, Fleming J, Reeve J. Implementing change in primary care practice: lessons from a mixed-methods evaluation of a frailty initiative. BJGP open. 2018 Apr 1;2(1). | Study not about effectiveness |
| 223 | Bryce K, Tai CK, Murray S, Fearn R. PTU-080 Should we all be looking for Marginal Gains in Endoscopy Efficiency?. | Conference abstract |
| 224 | Bunce AE, Gruß I, Davis JV, Cowburn S, Cohen D, Oakley J, Gold R. Lessons learned about the effective operationalization of champions as an implementation strategy: results from a qualitative process evaluation of a pragmatic trial. Implementation Science. 2020 Dec;15(1):1-2. | Study not about effectiveness |
| 225 | Burgess LH, Miller K, Cooper M, Moody J, Englebright J, Septimus E. Phased implementation of an antimicrobial stewardship program for a large community hospital system. American journal of infection control. 2019 Jan 1;47(1):69-73. | Study not about effectiveness |
| 226 | Burke J (2007). Champions change the way nursing care is delivered. Registered Nurse Journal 2007;19(1):21-21 | Study not about effectiveness |
| 227 | Burnett K, Miller R, McElroy S, Friel A. An investigation of prescriber perceptions on the benefits of, and barriers to, the successful implementation of antimicrobial prescribing guidelines in secondary care. International Journal of Pharmacy Practice. 2012 Apr 24;20(Suppl):13. | Conference abstract |
| 228 | Burrell M. A Case Study: Fostering Leadership of Champion Nurses for IHI Bundle Implementation with Graduate Student Collaboration. University of California, Davis; 2012. | Study not about effectiveness |
| 229 | Busch A.B., Laband A., Kos A.I., Weigel T. Implementing electronic patient reported outcomes measurement (ePROMs) in usual care psychiatric settings. Journal of Mental Health Policy and Economics 2016;19(Supplement 1):S2- | Conference abstract |
| 230 | Butler D. Initiating palliative care in rural cancer center. Journal of Palliative Medicine 2013;16(4):A19- | Conference abstract |
| 231 | Button T, Sreeramoju P, Smith EA, Rivers B, Snapp M, Couger D. Effectiveness of Reinventing System-Wide Hand Hygiene Program Utilizing Revised Hand Hygiene Observation Tool, Education, Hand Hygiene Champions and Development of an Interactive Intranet Based Data Entry Tool. American Journal of Infection Control. 2011 Jun 1;39(5):E164-5. | Conference abstract |
| 232 | Buxton H, Flynn E, Oluyinka O, Cumming O, Mills JE, Shiras T, Sara S, Dreibelbis R. Barriers and opportunities experienced by staff when implementing infection prevention and control guidelines during labour and delivery in healthcare facilities in Nigeria. Journal of Hospital Infection. 2019 Dec 1;103(4):428-34. | Study not about effectiveness |
| 233 | Byatt N, Moore Simas TA, Biebel K, Sankaran P, Pbert L, Weinreb L, Ziedonis D, Allison J. PRogram In Support of Moms (PRISM): a pilot group randomized controlled trial of two approaches to improving depression among perinatal women. Journal of Psychosomatic Obstetrics & Gynecology. 2018 Oct 2;39(4):297-306. | Study not about effectiveness |
| 234 | Byers JF, Beaudin CL. The relationship between continuous quality improvement and research. Journal for healthcare quality: official publication of the National Association for Healthcare Quality. 2002 Jan 1;24(1):4-8. | Study not about effectiveness |
| 235 | Byers V. The challenges of leading change in health‐care delivery from the front‐line. Journal of Nursing Management. 2017 Sep;25(6):449-56. | Not a champion |
| 236 | Byock I. Affecting Change: Putting Theory to Practice (FR400). Journal of Pain and Symptom Management. 2016 Feb 1;51(2):345. | Conference abstract |
| 237 | Byrne A, Nelson A, Moore B, Woodward A, Evans M, Shankland S. Raising Awareness of Research in Palliative Care to Clinical Teams-The Roadshow. Palliative Medicine. 2012 Jun;26(4). | Conference abstract |
| 238 | Byrnes J. Physician champions drive value-but how do you find them? The environment of healthcare reform and the drive for quality and cost containment have created a critical need for physician champions who can deliver on the value proposition. Healthcare Financial Management. 2012 Feb 1;66(2):100-2. | Study not about effectiveness |
| 239 | Byron G, Ziedonis DM, McGrath C, Frazier JA, deTorrijos F, Fulwiler C. Implementation of mindfulness training for mental health staff: Organizational context and stakeholder perspectives. Mindfulness. 2015 Aug;6(4):861-72. | Study not about effectiveness |
| 240 | Cabilan CJ. Falls risk assessment and falls prevention strategies in private oncology and neurosurgical setting: a best practice implementation project. JBI Evidence Synthesis. 2014 Oct 1;12(10):218-33. | Study not about effectiveness |
| 241 | Cabral M, Bartula D, Dellagrotta E. WOUND/OSTOMY NURSE FELLOWSHIP PROGRAM IN THE ACUTE CARE SETTING. InJOURNAL OF WOUND OSTOMY AND CONTINENCE NURSING 2020 May 1 (Vol. 47, pp. S25-S25). TWO COMMERCE SQ, 2001 MARKET ST, PHILADELPHIA, PA 19103 USA: LIPPINCOTT WILLIAMS & WILKINS. | Conference abstract |
| 242 | Cadilhac D.A., Moss K. Factors that influence the delivery of evidence-based care in stroke. Cerebrovascular Diseases 2010;29(SUPPL. 2):229- | Conference abstract |
| 243 | Caguioa J. Project Hands: Standardising Intravascular Practice and Preventing Infections. Journal of Vascular Access 2014;15(3):219- | Conference abstract |
| 244 | Cahill L.S., Turville M.L., Mak-Yuen Y., Carey L.M. Building champion neuro-therapists: The SENSe implement study. International Journal of Stroke 2015;10(SUPPL. 3):57. | Conference abstract |
| 245 | Cahill LS, Lannin NA, Mak-Yuen YY, Turville ML, Carey LM. Changing practice in the assessment and treatment of somatosensory loss in stroke survivors: protocol for a knowledge translation study. BMC health services research. 2018 Dec;18(1):1-8. | Protocol |
| 246 | Call KT, Cleary J, Harwood EM, Manser ST, Rogers EA. UNDERSTANDING THE" WHYAND HOW" OF INTEGRATING COMMUNITY HEALTH WORKERS INTO PRIMARY CARE TEAMS. InJOURNAL OF GENERAL INTERNAL MEDICINE 2016 May 1 (Vol. 31, pp. S448-S448). 233 SPRING ST, NEW YORK, NY 10013 USA: SPRINGER. | Conference abstract |
| 247 | Callister LC. Ethics of infant relinquishment, cultural considerations, and obstetric conveniences. MCN: The American Journal of Maternal/Child Nursing. 2011 May 1;36(3):171-7. | Study not about effectiveness |
| 248 | Cameron E, Haque M, Schwartz N, Khan S, Truscott R, Evans W. OA09. 01 5As to 3As: Evolution of the Systematic Approach to Smoking Cessation in Ontario’s Regional Cancer Centres. Journal of Thoracic Oncology. 2018 Oct 1;13(10):S340. | Conference abstract |
| 249 | Campbell MK, Tessaro I, Gellin M, Valle CG, Golden S, Kaye L, Ganz PA, McCabe MS, Jacobs LA, Syrjala K, Anderson B. Adult cancer survivorship care: experiences from the LIVESTRONG centers of excellence network. Journal of Cancer Survivorship. 2011 Sep 1;5(3):271. | Study not about effectiveness |
| 250 | Campbell MR. The Effect of an Early Mobility Protocol in Critically Ill Mechanically Ventilated Patients on Incidence and Duration of Delirium and Length of Stay. | Conference abstract |
| 251 | Carayon P. Human factors in patient safety as an innovation. Applied ergonomics. 2010 Sep 1;41(5):657-65. | Not a champion |
| 252 | Carey L, Cahill L, Lannin N. SENSe Implement: Changing clinical practice in sensory rehabilitation of the arm after stroke. InINTERNATIONAL JOURNAL OF STROKE 2017 Aug 1 (Vol. 12, pp. 15-15). 1 OLIVERS YARD, 55 CITY ROAD, LONDON EC1Y 1SP, ENGLAND: SAGE PUBLICATIONS LTD. | Conference abstract |
| 253 | Carlsen DR, True MW, Morrow CC. The Diabetes Champion Course: A Novel Outreach Program with Global Impact. InDIABETES 2014 Jun 1 (Vol. 63, pp. A311-A311). 1701 N BEAUREGARD ST, ALEXANDRIA, VA 22311-1717 USA: AMER DIABETES ASSOC. | Conference abstract |
| 254 | Carrigan R, Lahiri S. How easily can we access anaesthesia guidelines on the trust intranet? A quality improvement project to improve accessibility to clinical resources: 42. Anaesthesia. 2015 Jun;70. | Conference abstract |
| 255 | Carrillo M, Sias J, Navarrete JP, Aboud S, Valenzuela E. EXPANSION of diabetes education in a United States–Mexico border community (Expanding Services for Patients to Acquire New Skills, Set Goals, and Improve Overall Knowledge). Journal of the American Pharmacists Association. 2018 Jan 1;58(1):30-5. | Study not about effectiveness |
| 256 | Carroll JK, Pulver G, Dickinson LM, Pace WD, Vassalotti JA, Kimminau KS, Manning BK, Staton EW, Fox CH. Effect of 2 clinical decision support strategies on chronic kidney disease outcomes in primary care: a cluster randomized trial. JAMA network open. 2018 Oct 5;1(6):e183377-. | Study not about effectiveness |
| 257 | Carta T, Gawaziuk JP, Cristall N, Forbes L, Logsetty S. Evaluation of a multidisciplinary burn care journal club: lessons learned. Burns. 2018 May 1;44(3):560-5. | Study not about effectiveness |
| 258 | Carter A, Harrison M, Kryworuchko J, Kekwaletswe T, Wong S, Goldstein J, Warner G. P091: Essential elements to implementing the paramedics providing palliative care at home program: an application of the Consolidated Framework for Implementation Research (CFIR). Canadian Journal of Emergency Medicine. 2020 May;22(S1):S97-. | Conference abstract |
| 259 | Carter EJ, Pallin DJ, Mandel L, Sinnette C, Schuur JD. A qualitative study of factors facilitating clinical nurse engagement in emergency department catheter-associated urinary tract infection prevention. JONA: The Journal of Nursing Administration. 2016 Oct 1;46(10):495-500. | Study not about effectiveness |
| 260 | Carter M, Garvey A, Gibson L, McDevitt S. GP293 Developing MARSIPAN integrated care pathways for children and adolescents with eating disorders-real world challenges and recommendations. | Conference abstract |
| 261 | Carter R, Weiss K, Manrique R, Le C, Fleming S, Romanowski G, Golembeski D, Song R, Moyer L. REDUCING LENGTH OF STAY IN NEONATAL ABSTINENCE SYNDROME THROUGH QUALITY IMPROVEMENT. InJOURNAL OF INVESTIGATIVE MEDICINE 2020 Jan 1 (Vol. 68, pp. A119-A120). BRITISH MED ASSOC HOUSE, TAVISTOCK SQUARE, LONDON WC1H 9JR, ENGLAND: BMJ PUBLISHING GROUP. | Conference abstract |
| 262 | Casey CM, Parker EM, Winkler G, Liu X, Lambert GH, Eckstrom E. Lessons learned from implementing CDC’s STEADI falls prevention algorithm in primary care. The Gerontologist. 2017 Aug 1;57(4):787-96. | Study not about effectiveness |
| 263 | Castaldi M, Kappus M, Samuels S, Parsikia A, Parmalee T, McNelis J. 110: CLINICAL DOCUMENTATION SPECIALISTS IMPROVE REVENUE COLLECTION IN SURGICAL TRAUMA/SICU. Critical Care Medicine. 2018 Jan 1;46(1):38. | Conference abstract |
| 264 | Castaldi M, McNelis J. Introducing a clinical documentation specialist to improve coding and collectability on a surgical service. The Journal for Healthcare Quality (JHQ). 2019 May 1;41(3):e21-9. | Study not about effectiveness |
| 265 | Castaldo J, MacKenzie RS, Barbour PJ, Spikol L, Isayev Y. Safe Emergency Management of Acute Ischemic Stroke: An Academic Community Hospital Decade Experience. InSTROKE 2009 Apr 1 (Vol. 40, No. 4, pp. E206-E206). 530 WALNUT ST, PHILADELPHIA, PA 19106-3621 USA: LIPPINCOTT WILLIAMS & WILKINS. | Conference abstract |
| 266 | Caterson SA, Singh M, Orgill D, Ghazinouri R, Ciociolo G, Laskowski K, Greenberg JO. Development of Standardized Clinical Assessment and Management Plans (SCAMPs) in plastic and reconstructive surgery. Plastic and Reconstructive Surgery Global Open. 2015 Sep;3(9). | Study not about effectiveness |
| 267 | Cates JR, Calo W, Trogdon J, Diehl SJ, Stockton LL, Gurbani A, Coyne-Beasley T. Application of the Consolidated Framework for Implementation Research (CFIR) to identify factors that may influence implementation of a practice-based communication intervention to normalize HPV vaccination among preteens. Journal of Adolescent Health. 2018 Feb 1;62(2):S86-7. | Conference abstract |
| 268 | Catherwood A, Goodwin J, Quested B. CLINICAL CHAMPIONS AT THE BEDSIDE IMPROVING TRANSFUSION SAFETY FOR PATIENTS: P-791. Vox Sanguinis. 2015 Jun;109:358-9. | Conference abstract |
| 269 | Cavallari LH, Lee CR, Duarte JD, Nutescu EA, Weitzel KW, Stouffer GA, Johnson JA. Implementation of inpatient models of pharmacogenetics programs. American Journal of Health-System Pharmacy. 2016 Dec 1;73(23):1944-54. | Study not about effectiveness |
| 270 | Cave CE. Evidence‐Based Continence Care: An Integrative Review. Rehabilitation Nursing. 2016 Aug. | Letters to the editor/ Review studies |
| 271 | Centeno M., Fullerton C. Quality improvement efforts to reduce severe hypoglycemia in a large hospital system. International Journal for Quality in Health Care 2017;29(Supplement 1):48- | Conference abstract |
| 272 | Centofanti J, Duan E, Hoad N, Waugh L, Perri D, Cook D. RESIDENTS’PERSPECTIVES ON A DAILY GOALS CHECK-LIST: A MIXED-METHODS STUDY. InCRITICAL CARE MEDICINE 2012 Dec 1 (Vol. 40, No. 12, pp. U167-U167). 530 WALNUT ST, PHILADELPHIA, PA 19106-3621 USA: LIPPINCOTT WILLIAMS & WILKINS. | Conference abstract |
| 273 | Centofanti JE, Duan EH, Hoad NC, Swinton ME, Perri D, Waugh L, Cook DJ. Use of a daily goals checklist for morning ICU rounds: a mixed-methods study. Critical care medicine. 2014 Aug 1;42(8):1797-803. | Study not about effectiveness |
| 274 | Chaillet N, Dubé E, Dugas M, Francoeur D, Dubé J, Gagnon S, Poitras L, Dumont A. Identifying barriers and facilitators towards implementing guidelines to reduce caesarean section rates in Quebec. Bulletin of the World Health Organization. 2007;85:791-7. | Study not about effectiveness |
| 275 | Chakrabarti AK. The role of champion in product innovation. California management review. 1974 Dec;17(2):58-62. | Not within a health care setting |
| 276 | Chalk A, Page S. Dementia RED (Respect Empathy Dignity): Collaborating to build dementia supportive communities in North Wales–reporting on a pilot project (innovative practice). Dementia. 2016 Mar;15(2):257-62. | Study not about effectiveness |
| 277 | Chanchlani N. 70 Sustaining and developing movements, like evidence live, in medicine: lessons to be learnt when does a campaign become a movement–and how do we implement a movement into practice?. | Conference abstract |
| 278 | Chan-Dominy, A., Liyanage, K., Forbat, E., Hart, T., Matemera, J., Peters R. Quality improvement project on patient diary in intensive care unit to build the picture on critical illness events for the road of survival and rehabilitation. Intensive Care Medicine Experimental 2018;6(Supplement 2):- | Conference abstract |
| 279 | Chang C, Baharlou S, Briones A, Jacome M, Alfonso F, Berns S. Redesigning the Electronic Health Record to Improve Advance Care Planning: C136. Journal of the American Geriatrics Society. 2015 May;63. | Conference abstract |
| 280 | Charafeddine L, Masri S, Ibrahim P, Badin D, Cheayto S, Tamim H. Targeted educational program improves infant positioning practice in the NICU. International Journal for Quality in Health Care. 2018 Oct 1;1:7. | Study not about effectiveness |
| 281 | Charlebois A, Quinlan B, Twyman K. N057 The Role of Registered Nurse Champions in the Implementation of an In-Patient Diabetes Management Program Within a Tertiary Cardiac Care Institution. Canadian Journal of Cardiology. 2012 Sep 1;28(5):S438. | Conference abstract |
| 282 | Charnaya O, Ahn SY. Quality Improvement Initiative to Reduce Admissions for Nephrotic Syndrome Relapse in Pediatric Patients. Frontiers in pediatrics. 2019 Mar 29;7:112. | Study not about effectiveness |
| 283 | Chatfield A, Caglia JM, Dhillon S, Hirst J, Cheikh Ismail L, Abawi K, Kac G, Al Dhaheri AS, Villar J, Kennedy S, Langer A. Translating research into practice: the introduction of the INTERGROWTH‐21st package of clinical standards, tools and guidelines into policies, programmes and services. BJOG: An International Journal of Obstetrics & Gynaecology. 2013 Sep;120:139-42. | Study not about effectiveness |
| 284 | Chaves G, Turk-Adawi K, Supervia M, Santiago de Araújo Pio C, Abu-Jeish AH, Mamataz T, Tarima S, Lopez Jimenez F, Grace SL. Cardiac rehabilitation dose around the world: Variation and correlates. Circulation: Cardiovascular Quality and Outcomes. 2020 Jan;13(1):e005453. | Study not about effectiveness |
| 285 | Cheadle A, Cromp D, Krieger JW, Chan N, McNees M, Ross-Viles S, Kellogg R, Rahimian A, MacDougall E. Promoting policy, systems, and environment change to prevent chronic disease: lessons learned from the king county communities putting prevention to work initiative. Journal of Public Health Management and Practice. 2016 Jul 1;22(4):348-59. | Not within a health care setting |
| 286 | Cheah J, Heng BH. Implementing chronic disease management in the public healthcare sector in Singapore: the role of hospitals. World hospitals and health services: the official journal of the International Hospital Federation. 2001 Jan 1;37(3):19-23. | Study not about effectiveness |
| 287 | Chen JC, Goetz MB, Feld JE, Taylor A, Anaya H, Burgess J, de Mesa Flores R, Gidwani RA, Knapp H, Ocampo EH, Asch SM. A provider participatory implementation model for HIV testing in an ED. The American journal of emergency medicine. 2011 May 1;29(4):418-26. | Study not about effectiveness |
| 288 | Chen K., Goldberg T. Provider barriers to linking patients with community health coaches at an urban clinic. Journal of General Internal Medicine 2019;34(2 Supplement):S761- | Conference abstract |
| 289 | Chen X., Levitt C., Ivers N. Capacity building in a cross-jurisdictional primary care research team. Canadian Family Physician 2015;61(2 Supplement 1):S61- | Conference abstract |
| 290 | Chen Y, Lin F, Marshall A. Implementing same day discharge following percutaneous coronary intervention: A process evaluation. Journal of nursing care quality. 2019 Jan 1;34(1):54-60. | Conference abstract |
| 291 | Chen Y, Marshall A, Lin F. Implementation strategies for same day discharge post percutaneous coronary intervention: an integrative review. Worldviews on Evidence‐Based Nursing. 2016 Oct;13(5):371-9. | Letters to the editor/ Review studies |
| 292 | Cheng C, Dewa C, Goering P. Diffusion of innovation: the experiences in Ontario with EIP community mental health services in the Matryoshka Project. Early Intervention in Psychiatry. 2010 Nov;4. | Conference abstract |
| 293 | Cheong C.P., Siah C.J. Explorati on of older nurses' perspectives as end-users during the implementati on of clinical informati on system-a qualitative study. Proceedings of Singapore Healthcare 2010;19(SUPPL. 2):S275- | Conference abstract |
| 294 | Chernetsky Tejedor SG, Garrett G, Jacob J, Stein J, Phillips L, Meyer E, Dent Reyes M, Robichaux C, Steinberg JP. Electronic documentation of central line-days: validation is essential. InProgram and abstracts of the 2011 Society for Healthcare Epidemiologists of America (SHEA) Annual Meeting 2011 Apr 1. | Conference abstract |
| 295 | Cherney RL, Pandian V, Ninan A, Eastman D, Barnes B, King E, Miller B, Judkins S, Smith IV AE, Smith NM, Hanley J. The trach trail: a systems-based pathway to improve quality of tracheostomy care and interdisciplinary collaboration. Otolaryngology–Head and Neck Surgery. 2020 Aug;163(2):232-43. | Study not about effectiveness |
| 296 | Cherney, R., Pandian, V., Brenner, M.J. A tracheostomy pathway to enhance interdisciplinary collaboration and outcomes. Otolaryngology - Head and Neck Surgery 2019;161(2 Supplement):P125- | Conference abstract |
| 297 | Chernitskiy V, DeVito A, Neeman N, Sehgal N, Yazdany J, Gross A. Integrating Collection of Rheumatoid Arthritis Disease Activity and Physical Function Scores into an Academic Rheumatology Practice to Improve Quality of Care.: 1353. Arthritis & Rheumatology. 2014 Oct;66. | Conference abstract |
| 298 | Chesis N. A Quality Improvement Project to Reduce the Incidence of Nonmedically Indicated Elective Deliveries Before 39 Weeks. Journal of Obstetric, Gynecologic, & Neonatal Nursing. 2015 Jun;44(s1):S49-50. | Conference abstract |
| 299 | Cheyne H, Abhyankar P, McCourt C. Empowering change: realist evaluation of a Scottish Government programme to support normal birth. Midwifery. 2013 Oct 1;29(10):1110-21. | Study not about effectiveness |
| 300 | Chibber KS, Krishnan S, Minkler M. Physician practices in response to intimate partner violence in southern India: insights from a qualitative study. Women & health. 2011 Feb 28;51(2):168-85. | Not about knowledge translation/evidence-based practice |
| 301 | Chiffelle R, Cabrera-Behler M, del Castillo CY. Evidence Based Practice: Cryotherapy for Patients Receiving High Dose Melphalan. Biology of Blood and Marrow Transplantation. 2014 Feb 1;20(2):S298. | Conference abstract |
| 302 | Childress SB, Buckley T, Badke A, Horyna A, Howell J, Gren L, Beck AC. Hardwiring advance directives into an electronic medical record. | Conference abstract |
| 303 | Chin J, Evans A, McGowan T, McKnight L, Hunter A, McLeod R, Irish J, Fleshner N, Srigley J. 188 IMPROVING MARGIN STATUS IN RADICAL PROSTATECTOMIES THROUGH PERFORMANCE MEASUREMENT AND MULTIDISCIPLINARY KNOWLEDGE TRANSFER ACTIVITIES. The Journal of Urology. 2012 Apr;187(4S):e79-. | Conference abstract |
| 304 | Chisholm L, Zimmerman S, Rosemond C, McConnell E, Weiner BJ, Lin FC, Hanson L. Nursing home staff perspectives on adoption of an innovation in goals of care communication. Geriatric Nursing. 2018 Mar 1;39(2):157-61. | Study not about effectiveness |
| 305 | Chiu H, Murphy-Burke D, Werb R, Jung B, Chan-Yan C, Duncan J, Forzley B, Lowry R, Hargrove G, Carson R, Levin A. ADVANCING QUALITY IN PROVINCIAL END-OF-LIFE CARE FOR PATIENTS WITH KIDNEY DISEASE IN CANADA. InNEPHROLOGY DIALYSIS TRANSPLANTATION 2014 May 1 (Vol. 29, pp. 401-401). GREAT CLARENDON ST, OXFORD OX2 6DP, ENGLAND: OXFORD UNIV PRESS. | Conference abstract |
| 306 | Chiwera L, Wigglesworth N, McCoskery C, Lucchese G, Newsholme W. Reducing adult cardiac surgical site infections and the economic impact of using multidisciplinary collaboration. Journal of Hospital Infection. 2018 Dec 1;100(4):428-36. | Study not about effectiveness |
| 307 | Chmielewski NA, Faulkner LD, Drone M. Using Floor Champions to Improve Sepsis Quality Outcomes in Community Hospitals. Nurse Leader. 2019 Apr 1;17(2):151-4. | Study not about effectiveness |
| 308 | Chodhari R, Greenberg M, Geraets A. From Theory to Practice: Asthma Discharge Planning–A quality improvement project. Paediatric Respiratory Reviews. 2013(14):S58. | Conference abstract |
| 309 | Choe HM, Lin AT, Kobernik K, Cohen M, Wesolowicz L, Qureshi N, Leyden T, Share DA, Darland R, Spahlinger DA. Michigan pharmacists transforming care and quality: developing a statewide collaborative of physician organizations and pharmacists to improve quality of care and reduce costs. Journal of managed care & specialty pharmacy. 2018 Apr;24(4):373-8. | Study not about effectiveness |
| 310 | Chong TW, Chiu E. Researchers, please take due care of the formal caregivers in nursing homes. International psychogeriatrics. 2020 Mar;32(3):311-3. | Study not about effectiveness |
| 311 | Choy-Brown M. Examining Supervision as an Implementation Strategy to Improve Provider Adoption of Evidence-Based Practice: A Mixed Methods Study (Doctoral dissertation, New York University). | Study not about effectiveness |
| 312 | Chrupcala KA, Edwards TM, Spatz DL. A continuous quality improvement project to implement infant-driven feeding as a standard of practice in the newborn/infant intensive care unit. Journal of Obstetric, Gynecologic & Neonatal Nursing. 2015 Sep 1;44(5):654-64. | Study not about effectiveness |
| 313 | Chua C.G., Yeo S.L., Ho Y.Y., Cheng L., Tang A., Ong G.N., Tang Y.M., Loong S.W., Teo M.C., Seet B.N., Goh H. Improving patient safety through increased hand hygiene compliance in the dialysis centrer. American Journal of Infection Control 2009;37(5):E127- | Conference abstract |
| 314 | Chua C.G., Yeo S.L., Ho Y.Y., Cheng L., Tang A., Ong G.N., Tang Y.M., Loong S.W., Teo M.C., Seet B.N., Lim P.S., Chua P.T., Goh H. Are we wasting or saving dollars-in the dialysis centres? American Journal of Infection Control 2009;37(5):E34-E35 | Conference abstract |
| 315 | Chuo J, Grayson S, Hufnal C, Carroll J, Jani G. Results from Implementing Medical Checklist ‘Rounding Tool’into Care Practices in a Large Neonatal Intensive Care Network. | Conference abstract |
| 316 | Chyten-Brennan J, Ginsburg Z, Nisen MB, Lipshie-Williams M, Goodenough E, Viraj PV, Beil R. TRANSFORMING A LARGE URBAN HEALTH SYSTEM TO PROVIDE COMPREHENSIVE TRANSGENDER CARE. InJOURNAL OF GENERAL INTERNAL MEDICINE 2017 Apr 1 (Vol. 32, pp. S804-S805). 233 SPRING ST, NEW YORK, NY 10013 USA: SPRINGER. | Conference abstract |
| 317 | Cifra CL, Houston M, Otto A, Kamath SS. Prompting rounding teams to address a daily best practice checklist in a pediatric intensive care unit. The Joint Commission Journal on Quality and Patient Safety. 2019 Aug 1;45(8):543-51. | Study not about effectiveness |
| 318 | Cilliers M, Stokes G, Hunt N. Champions of dementia care. Interview by Jenny Knight. Nursing standard (Royal College of Nursing (Great Britain): 1987). 2010 Aug 1;24(49):18-9. | Not about knowledge translation/evidence-based practice |
| 319 | Circo, K.Addressing and maintaining an infection free environment with foley catheters. Journal of Burn Care and Research 2016;37(SUPPL. 1):S258- | Conference abstract |
| 320 | Clapp, J C. A Multidisciplinary Team Approach to Management of Postpartum Hemorrhage Journal of Obstetric, Gynecologic, and Neonatal Nursing : JOGNN 2015;44(s1):S22 | Conference abstract |
| 321 | Clark A.,​ Wolgast K.A.,​ Mazur N.,​ Mekis A. (2020). Leading Change in Nurse Bedside Shift Report. The Nursing clinics of North America 2020;55(1):21-28 | Study not about effectiveness |
| 322 | Clark B, Kai M, Dix R, White J, Rozenfeld Y, Levy S, Engstrom K. Association of a multimodal educational intervention for primary care physicians with prescriptions of buprenorphine for opioid use disorders. JAMA network open. 2019 Oct 2;2(10):e1913818-. | Study not about effectiveness |
| 323 | Clark M, Pledge A. G591 Using multilingual patient education videos to support a prompt and safe discharge from the emergency department. | Conference abstract |
| 324 | Clark TA, Bak L. Reducing Hypoglycemia through Increased Knowledge of an Evidence-Based Guideline. 2018 | Conference abstract |
| 325 | Clarke R, Hackbarth AS, Saigal C, Skootsky SA. Building the infrastructure for value at UCLA: engaging clinicians and developing patient-centric measurement. Academic Medicine. 2015 Oct 1;90(10):1368-72. | Study not about effectiveness |
| 326 | Clarkson J.E.,​ Bonetti D. (2009). Why Be an Evidence-Based Dentistry Champion? Journal of Evidence-Based Dental Practice 2009;9(3):145-150 | Study not about effectiveness |
| 327 | Clay-Williams R, Nosrati H, Cunningham FC, Hillman K, Braithwaite J. Do large-scale hospital-and system-wide interventions improve patient outcomes: a systematic review. BMC health services research. 2014 Dec;14(1):1-3. | Letters to the editor/ Review studies |
| 328 | Clement CM, Stiell IG, Lowe MA, Brehaut JC, Calder LA, Vaillancourt C, Perry JJ. Facilitators and barriers to application of the Canadian C-spine rule by emergency department triage nurses. International emergency nursing. 2016 Jul 1;27:24-30. | Study not about effectiveness |
| 329 | Cohen AN, Chinman MJ, Hamilton AB, Whelan F, Young AS. Using patient-facing kiosks to support quality improvement at mental health clinics. Medical care. 2013 Mar;51(3 0 1):S13. | Study not about effectiveness |
| 330 | Cohen E. Building Capacity Through Experiential Learning. InCANCER NURSING 2016 Nov 1 (Vol. 39, pp. S29-S30). TWO COMMERCE SQ, 2001 MARKET ST, PHILADELPHIA, PA 19103 USA: LIPPINCOTT WILLIAMS & WILKINS. | Conference abstract |
| 331 | Cohen R, Gooberman-Hill R. Staff experiences of enhanced recovery after surgery: systematic review of qualitative studies. BMJ open. 2019 Feb 1;9(2):e022259. | Letters to the editor/ Review studies |
| 332 | Cole A, Keppel GA, Linares A, Alto W, Kriegsman W, Reed A, Holmes J, Mohanachandran M, Baldwin LM. Evaluating the development, implementation and dissemination of a multisite card study in the WWAMI Region Practice and Research Network. Clinical and translational science. 2015 Dec;8(6):764-9. | Study not about effectiveness |
| 333 | Cole J, Rengel A, Ruiz M. HealthPartners Institute, Minneapolis, MN Equitable Care Educational Strategy. Ochsner Journal. 2018 Mar 20;18(S1):24-5. | Conference abstract |
| 334 | Coleman C, Formenti S, Williams T, Petereit D, Pistenmaa D, Grover S, Vikram B. The International Cancer Expert Corps (ICEC): a unique global mentoring model for building sustainable expertise in low-and lower-middle income countries and geographically remote areas in resource-rich countries. Annals of Global Health. 2015 Mar 12;81(1). | Conference abstract |
| 335 | Coleman CN, Daphtary M, Vikram B. 46: A critical challenge for 21st century radiation oncology: reaching the underserved in Low-Middle Income Countries. Radiotherapy and Oncology. 2014(110):S23-4. | Conference abstract |
| 336 | Coleman PL, Shelton C, Mott B, Horgan K, Demmel KM, Flesch L. Effective Use of a Chemotherapy/Stem Cell Champion on a Complex Bone Marrow Transplantation Unit. Biology of Blood and Marrow Transplantation. 2016 Mar 1;22(3):S445. | Conference abstract |
| 337 | Colgan J, Petersen A. Development of a smoking cessation ward nurse champion programme. InEuroHeartCare 2018 2018 (Vol. 70, p. 92). | Conference abstract |
| 338 | Collins K, Moore A, Winter C, Lenguerrand E, Siassakos D, Draycott T. Understanding the implementation of obstetric emergency training: The THISTLE-Plus study. InBJOG-AN INTERNATIONAL JOURNAL OF OBSTETRICS AND GYNAECOLOGY 2017 Mar 1 (Vol. 124, pp. 43-44). 111 RIVER ST, HOBOKEN 07030-5774, NJ USA: WILEY. | Conference abstract |
| 339 | Collins KF, Muthusamy SK, Carr A. Toyota production system for healthcare organisations: prospects and implementation challenges. Total Quality Management & Business Excellence. 2015 Aug 3;26(7-8):905-18. | Study not about effectiveness |
| 340 | Collinsworth AW. A bundled care process for the prevention of delirium in patients in the intensive care unit: implementation strategies and uptake, impact on patient outcomes, and cost-effectiveness (Doctoral dissertation, Tulane University, School of Public Health and Tropical Medicine). | Study not about effectiveness |
| 341 | Colton D. Implementing industrial models of quality improvement in human service organizations: The health care perspective. University of Virginia; 1997. | Letters to the editor/ Review studies |
| 342 | Combes G, Allen K, Sein K, Girling A, Lilford R. Taking hospital treatments home: a mixed methods case study looking at the barriers and success factors for home dialysis treatment and the influence of a target on uptake rates. Implementation Science. 2015 Dec;10(1):1-3. | Not within a health care setting |
| 343 | Communications N. Navigating today's CDI landscape: 10 strategies for success. Healthcare financial management: journal of the Healthcare Financial Management Association. 2014 Jan;68(1):1-6. | Study not about effectiveness |
| 344 | Compas C, Hopkins KA, Townsley E. Best practices in implementing and sustaining quality of care: a review of the quality improvement literature. Research in Gerontological Nursing. 2008 Jul 1;1(3):209-16. | Letters to the editor/ Review studies |
| 345 | Conklin J, Stolee P, Luesby D, Sharratt MT, Chambers LW. Enhancing service delivery capacity through knowledge exchange: the seniors health research transfer network. InHealthcare Management Forum 2007 Dec (Vol. 20, No. 4, pp. 20-26). Sage CA: Los Angeles, CA: SAGE Publications. | Study not about effectiveness |
| 346 | Conkol KJ, Martinez-Strengel A, Coller RJ, Bergman DA, Whelan EM. Pediatric hospitalists’ lessons learned from an innovation award to improve care for children with medical complexity. Hospital Pediatrics. 2020 Aug 1;10(8):694-701. | Conference abstract |
| 347 | Connelly B, Ujano-De Motta LL, Leonard C, Mayberry A, Kelley L, Gaskin D, Gilmartin HM. Mapping the reach of a rural Transitions Nurse Program for veterans with geographic information systems. Implementation science communications. 2020 Dec;1(1):1-7. | Study not about effectiveness |
| 348 | Conoscenti E., Campanella O., Pensato L.. Fazzina M.L., Spina C.; Caruso S., Barone M., Lombardo R., Gioe S.M., Arena G. Martucci G.; Mularoni A. Carvalho Laborne Valle M. Massaud Ribeiro L., Goncalves Panisset A. A quality improvement strategy implementation for sternal wound infections after cardiac surgery. The infection control nurse perpective. Intensive Care Medicine Experimental. 2019;7(Supplement 3) | Conference abstract |
| 349 | Contreras K, Kinderman A, Ferrell B. Cultivating Grass Roots in Drought Condition: Enhancing Palliative Care in Safety Net Hospitals Through a Statewide Initiative (FR408): Safety Net SIG. Journal of Pain and Symptom Management. 2013 Feb 1;45(2):369. | Conference abstract |
| 350 | Conway PH, Coyle S, Sonnenfeld N. Partnership for patients: Innovation and leadership for safer healthcare. Journal of Healthcare Management. 2017 May 1;62(3):166-70. | Study not about effectiveness |
| 351 | Cook M., Burger K., Drach L., Hansen K., McEachern M., Shields S.Implementation of a pediatric PC champions program. Journal of Palliative Medicine / 2017;20(4):A17-A18 | Conference abstract |
| 352 | Cooke D., Gustafsson L., Hinchilffe F., Eagles R. Translating evidence into practice-How does this happen? International Journal of Stroke / 2014;9(SUPPL. 2):33 | Conference abstract |
| 353 | Coombs T, Walter G, Brann P. Overview of the national mental health benchmarking project. Australasian Psychiatry. 2011 Feb;19(1):37-44. | Study not about effectiveness |
| 354 | Coope SA. Designing and implementing a trust-wide quality assurance programme. British journal of community nursing. 2018 Apr 2;23(4):190-6. | Study not about effectiveness |
| 355 | Cooper D, McFarland M, Petrilli F, Shells C. Reducing inappropriate antibiotics for urinary tract infections in long-term care: A replication study. Journal of nursing care quality. 2019 Jan 1;34(1):16-21. | Study not about effectiveness |
| 356 | Cooper G., Deeks L.Dementia, medication and care transitions: The current Australian experience. International Journal of Pharmacy Practice. 2012;20(SUPPL. 2):9-10 | Study not about effectiveness |
| 357 | Cooper GS, Armitage KB, Ashar B, Costantini O, Creighton FA, Raiz P, Wong RC, Carlson MD. Design and implementation of an inpatient disease management program. Am J Manag Care. 2000 Jul 1;6(7):793-801. | Study not about effectiveness |
| 358 | Cooper M, Cameron S. Successful implementation of immediate postpartum intrauterine contraception services in Edinburgh and framework for wider dissemination. International Journal of Gynecology & Obstetrics. 2018 Sep;143:56-61. | Study not about effectiveness |
| 359 | Cosper P, Morelock V, Provine B. Please release me: restraint reduction initiative in a health care system. Journal of nursing care quality. 2015 Jan 1;30(1):16-23. | Study not about effectiveness |
| 360 | Cotta MO, Robertson MS, Marshall C, Thursky KA, Liew D, Buising KL. Implementing antimicrobial stewardship in the Australian private hospital system: a qualitative study. Australian Health Review. 2015 Jan 5;39(3):315-22. | Study not about effectiveness |
| 361 | Cotton K. NICE CG83–rehabilitation after critical illness: implementation across a network. Nursing in critical care. 2013 Jan;18(1):32-42. | Study not about effectiveness |
| 362 | Courtney J., Lavery G. Survey of paediatric handover practices in Northern Ireland. Ulster Medical Journal. 2018;87(2):148 | Conference abstract |
| 363 | Courtwright SE, Mastro KA, Preuster C, Dardashti N, McGill S, Madelon M, Johnson D. Reducing hospital‐acquired pressure ulcers using bundle methodology in pediatric and neonatal patients receiving extracorporeal membrane oxygenation therapy: An integrative review and call to action. Journal for Specialists in Pediatric Nursing. 2017 Oct;22(4):e12188. | Letters to the editor/ Review studies |
| 364 | Cowan S, Pease A, Bennett S. Usage and impact of an online education tool for preventing sudden unexpected death in infancy. Journal of paediatrics and child health. 2013 Mar;49(3):228-32. | Study not about effectiveness |
| 365 | Cowell F, Gillespie S, Cheung G, Brown D. Complex regional pain syndrome in distal radius fractures: how to implement changes to reduce incidence and facilitate early management. Journal of Hand Therapy. 2018 Apr 1;31(2):201-5. | Study not about effectiveness |
| 366 | Cowie J, Calveley E, Bowers G, Bowers J. Evaluation of a digital consultation and self-care advice tool in primary care: a multi-methods study. International journal of environmental research and public health. 2018 May;15(5):896. | Study not about effectiveness |
| 367 | Cowie J, Nicoll A, Dimova ED, Campbell P, Duncan EA. The barriers and facilitators influencing the sustainability of hospital-based interventions: a systematic review. BMC health services research. 2020 Dec;20(1):1-27. | Letters to the editor/ Review studies |
| 368 | Cox T, DeMeyer ES, Brougher LI, Roach C, Billups R. Developing High-Quality, Interactive, Repeatable Web-Based Education Modules for Blood Cancer/Hematopoietic Cell Transplantation Nurse Professional Development. Biology of Blood and Marrow Transplantation. 2017 Mar 1;23(3):S381. | Conference abstract |
| 369 | Coyle SK, Mills ME. Nurse executives champion change in integrated health systems. Nursing management. 2000 Feb 1;31(2):32. | Study not about effectiveness |
| 370 | Crabtree BF, Miller WL, Tallia AF, Cohen DJ, DiCicco-Bloom B, McIlvain HE, Aita VA, Scott JG, Gregory PB, Stange KC, McDaniel RR. Delivery of clinical preventive services in family medicine offices. The Annals of Family Medicine. 2005 Sep 1;3(5):430-5. | Study not about effectiveness |
| 371 | Crabtree J, Mack J. Developing champions to enhance the care of people with dementia in general hospitals. Nursing times. 2010 Dec 1;106(48):13-4. | Not about knowledge translation/evidence-based practice |
| 372 | Crabtree J, Mack J. Developing champions to enhance the care of people with dementia in general hospitals. Nursing times. 2010 Dec 1;106(48):13-4. | Study not about effectiveness |
| 373 | Craig TJ, Perlin JB, Fleming BB. Self-reported performance improvement strategies of highly successful Veterans Health Administration facilities. American Journal of Medical Quality. 2007 Nov;22(6):438-44. | Study not about effectiveness |
| 374 | Cranley LA, Cummings GG, Profetto-McGrath J, Toth F, Estabrooks CA. Facilitation roles and characteristics associated with research use by healthcare professionals: a scoping review. BMJ open. 2017 Aug 1;7(8):e014384. | Letters to the editor/ Review studies |
| 375 | Craven DE. Preventing ventilator-associated pneumonia in adults: sowing seeds of change. Chest. 2006 Jul 1;130(1):251-60. | Study not about effectiveness |
| 376 | Creber RM, Dayan PS, Kuppermann N, Ballard DW, Tzimenatos L, Alessandrini E, Mistry RD, Hoffman J, Vinson DR, Bakken S, Pediatric Emergency Care Applied Research Network. Applying the RE-AIM framework for the evaluation of a clinical decision support tool for pediatric head trauma: a mixed-methods study. Applied clinical informatics. 2018 Jul;9(03):693-703. | Study not about effectiveness |
| 377 | Crilly J, Greenslade JH, Berndt S, Hawkins T, Cullen L. Facilitators and barriers for emergency department clinicians using a rapid chest pain assessment protocol: qualitative interview research. BMC health services research. 2020 Dec;20(1):1-1. | Study not about effectiveness |
| 378 | Crilly JL, Boyle J, Jessup M, Wallis M, Lind J, Green D, FitzGerald G. The implementation and evaluation of the patient admission prediction tool: assessing its impact on decision-making strategies and patient flow outcomes in 2 Australian hospitals. Quality management in health care. 2015 Oct 1;24(4):169-76. | Study not about effectiveness |
| 379 | Crockett LK, Leggett C, Curran J, Knisley L, Ripstein J, Brockman G, Scott S, Hartling L, Jabbour M, Johnson D, Klassen T. P024: Sharing evidence, experiences and expertise: the value of networking to standardize emergency care for kids in Canada. Canadian Journal of Emergency Medicine. 2018 May;20(S1):S65-. | Conference abstract |
| 380 | Crosby J, Gibas M, Ethiraj S, Heflin G, Clancy C. Utilizing Data Combined with Specialty Champions as Drivers for Accelerating and Maximizing Patient Blood Management Initiatives. InANESTHESIA AND ANALGESIA 2018 Sep 1 (Vol. 127, No. 3, pp. 47-48). TWO COMMERCE SQ, 2001 MARKET ST, PHILADELPHIA, PA 19103 USA: LIPPINCOTT WILLIAMS & WILKINS. | Conference abstract |
| 381 | Crosson JC, Etz RS, Wu S, Straus SG, Eisenman D, Bell DS. Meaningful use of electronic prescribing in 5 exemplar primary care practices. The Annals of Family Medicine. 2011 Sep 1;9(5):392-7. | Study not about effectiveness |
| 382 | Cuenca, E., Urden, L.D. Implementation of pain resource nurse & pain champion n program in a neurosurgical unit.Communicating Nursing Research 01. 2013;46():414-414 | Conference abstract |
| 383 | Cullen L, Hanrahan K, Farrington M, Anderson R, Dimmer E, Miner R, Suchan T, Rod E. Evidence-based practice change champion program improves quality care. JONA: The Journal of Nursing Administration. 2020 Mar 1;50(3):128-34. | Study not about effectiveness |
| 384 | Cummings J, Stadler D. Nutrition in Transition: Building Clinical Nutrition Capacity to Train Dietitians in Lao PDR. The FASEB Journal. 2017 Apr;31:786-46. | Conference abstract |
| 385 | Cunningham K, Whalen U, Semler M, Polancich S, Kripalani S. Morbidity, Mortality, and Improvement Conferences Engage Residents in Systems-Based Practice: 161. Journal Of Hospital Medicine. 2010 Mar;5:87-8. | Conference abstract |
| 386 | Currow, Senior Associate Editor DC. We need champions, passionate champions. Journal of palliative medicine. 2012 Aug 1;15(8):842-3. | Letters to the editor/ Review studies |
| 387 | Curtis JR, Nielsen EL, Treece PD, Downey L, Dotolo D, Shannon SE, Back AL, Rubenfeld GD, Engelberg RA. Integrating palliative and critical care: results of a cluster randomized trial. InD103. ETHICS AND END-OF-LIFE IN THE ICU 2010 May (pp. A6860-A6860). American Thoracic Society. | Conference abstract |
| 388 | Curtis JR, Treece PD, Nielsen EL, Downey L, Shannon SE, Braungardt T, Owens D, Steinberg KP, Engelberg RA. Integrating palliative and critical care: evaluation of a quality-improvement intervention. American journal of respiratory and critical care medicine. 2008 Aug 1;178(3):269-75. | Study not about effectiveness |
| 389 | da Silva AA, Marques AF, di Biase CB, Zingg W, Dramowski A, Sharland M. Interventions to prevent urinary catheter–associated infections in children and neonates: a systematic review. Journal of pediatric urology. 2018 Dec 1;14(6):556-e1. | Letters to the editor/ Review studies |
| 390 | Dachs R. Interventions to improve antibiotic prescribing practices for hospital inpatients. American family physician. 2008 Mar 1;77(5):618. | Study not about effectiveness |
| 391 | Daher, M., Doumit, M. Palliative care in Lebanon: Current practices, and perspectives for the future. Palliative Care: Perspectives, Practices and Impact on Quality of Life. A Global View, Volume 1 2017;():307-316 | Not about knowledge translation/evidence-based practice |
| 392 | Dale S, Levi C, Ward J, Grimshaw JM, Jammali‐Blasi A, D'Este C, Griffiths R, Quinn C, Evans M, Cadilhac D, Cheung NW. Barriers and enablers to implementing clinical treatment protocols for fever, hyperglycaemia, and swallowing dysfunction in the Quality in Acute Stroke Care (QASC) project—a mixed methods study. Worldviews on Evidence‐Based Nursing. 2015 Feb;12(1):41-50. | Study not about effectiveness |
| 393 | Dale S., Middleton S., Lydtin A., Comerford D., Hill K., Longworth M., McElduff P., D'Este C., Cadilhac D. The quality in acute stroke care (QASC) implementation project: State-wide evidence to practice translation. International Journal of Stroke / 2015;10(SUPPL. 2):198 | Conference abstract |
| 394 | Dalkin, S. The Realist Evaluation of a Palliative Integrated Care Pathway in Primary Care: What Works, For Whom and in What Circumstances?2014. Doctoral thesis, Northumbria University. | Not a champion |
| 395 | Dambisya YM, Matinhure S. Policy and programmatic implications of task shifting in Uganda: a case study. BMC health services research. 2012 Dec;12(1):1-0. | Study not about effectiveness |
| 396 | Damschroder LJ, Banaszak-Holl J, Kowalski CP, Forman J, Saint S, Krein SL. The role of the “champion” in infection prevention: results from a multisite qualitative study. BMJ Quality & Safety. 2009 Dec 1;18(6):434-40. | Study not about effectiveness |
| 397 | Damschroder LJ, Goodrich DE, Robinson CH, Fletcher CE, Lowery JC. A systematic exploration of differences in contextual factors related to implementing the MOVE! weight management program in VA: a mixed methods study. BMC health services research. 2011 Dec;11(1):1-3. | Study not about effectiveness |
| 398 | Damush TM, Miller KK, Plue L, Schmid AA, Myers L, Graham G, Williams LS. National implementation of acute stroke care centers in the Veterans Health Administration (VHA): formative evaluation of the field response. Journal of general internal medicine. 2014 Dec 1;29(4):845-52. | Study not about effectiveness |
| 399 | Danesh V, Gisi B, Narayan M, Yoder L, Zad O. Fecal Occult Blood Tests: Valuable for Screening, Wasteful for Diagnostics. Clinical Nurse Specialist. 2019 Jul 1;33(4):191-4. | Study not about effectiveness |
| 400 | Dao B, Otolorin E, Gomez PP, Carr C, Sanghvi H. Preparing the next generation of maternal and newborn health leaders: The maternal and newborn health champions initiatives. International Journal of Gynecology & Obstetrics. 2015 Jun;130:S40-5. | Study not about effectiveness |
| 401 | Darney BG, VanDerhei D, Weaver MR, Stevens NG, Prager SW. “We have to what?”: lessons learned about engaging support staff in an interprofessional intervention to implement MVA for management of spontaneous abortion. Contraception. 2013 Aug 1;88(2):221-5. | Study not about effectiveness |
| 402 | Darney BG, VanDerhei D, Weaver MR, Stevens NG, Prager SW. “We have to what?”: lessons learned about engaging support staff in an interprofessional intervention to implement MVA for management of spontaneous abortion. Contraception. 2013 Aug 1;88(2):221-5. | Study not about effectiveness |
| 403 | Darney BG, Weaver MR, VanDerhei D, Stevens NG, Prager SW. “One of those areas that people avoid” a qualitative study of implementation in miscarriage management. BMC health services research. 2013 Dec;13(1):1-9. | Study not about effectiveness |
| 404 | Darney BG, Weaver MR, VanDerhei D, Stevens NG, Prager SW. “One of those areas that people avoid” a qualitative study of implementation in miscarriage management. BMC health services research. 2013 Dec;13(1):1-9. | Study not about effectiveness |
| 405 | Darney BG. Program Evaluation in Reproductive Health: Prospective and Retrospective Applications (Doctoral dissertation). | Study not about effectiveness |
| 406 | Dart R., Egan B. Implementation lessons from the formation of a community-based hypertension practice network. Journal of Clinical Hypertension / 2012;14(SUPPL. 1) | Conference abstract |
| 407 | Davidson G, Duffy J, Barry L, Curry P, Darragh E, Lees J. Championing the interface between mental health and child protection: evaluation of a service initiative to improve joint working in Northern Ireland. Child Abuse Review. 2012 May;21(3):157-72. | Study not about effectiveness |
| 408 | Davidson P, Stewart S, Elliott D, Daly J, Sindone A, Cockburn J. Addressing the burden of heart failure in Australia: the scope for home-based interventions. Journal of cardiovascular nursing. 2001 Oct 1;16(1):56-68. | Study not about effectiveness |
| 409 | Davies A, James W, Griffiths L. Implementing a quality improvement programme in a locality mental health service. Nursing Management. 2021 Jun 3;28(3). | Study not about effectiveness |
| 410 | Davies B, Edwards N, Ploeg J, Virani T. Insights about the process and impact of implementing nursing guidelines on delivery of care in hospitals and community settings. BMC Health Services Research. 2008 Dec;8(1):1-5. | Study not about effectiveness |
| 411 | Davies MJ, Kristunas CA, Alshreef A, Dixon S, Eborall H, Glab A, Huddlestone L, Hudson N, Khunti K, Martin G, Northern A. The impact of an intervention to increase uptake to structured self-management education for people with type 2 diabetes mellitus in primary care (the embedding package), compared to usual care, on glycaemic control: study protocol for a mixed methods study incorporating a wait-list cluster randomised controlled trial. BMC family practice. 2019 Dec;20(1):1-5. | Study not about effectiveness |
| 412 | Davila S. Non-ventilator health care-associated pneumonia (NV-HAP): Taking action to improve NV-HAP outcomes. American journal of infection control. 2020 May 1;48(5):A28-35. | Study not about effectiveness |
| 413 | Davis C, Burke L. The effectiveness of clinical supervision for a group of ward managers based in a district general hospital: an evaluative study. Journal of Nursing Management. 2012 Sep;20(6):782-93. | Study not about effectiveness |
| 414 | Davis JM, Janczukowicz J, Stewart J, Quinn B, Feldman CA. Interprofessional education in dental education: An international perspective. InEuropean Journal of Dental Education 2018 Mar (Vol. 22, pp. 10-16). | Study not about effectiveness |
| 415 | Davis KF, Napolitano N, Li S, Buffman H, Rehder K, Pinto M, Nett S, Jarvis JD, Kamat P, Sanders Jr RC, Turner DA. Promoters and barriers to implementation of tracheal intubation airway safety bundle: A mixed–methods analysis. Pediatric critical care medicine: a journal of the Society of Critical Care Medicine and the World Federation of Pediatric Intensive and Critical Care Societies. 2017 Oct;18(10):965. | Study not about effectiveness |
| 416 | Davis PH, Hennig T, Dunkley SK, McCall CM, Long GD. Lab Changes Result in Optimizing Bone Marrow Biopsy Procedure and Processing. Biology of Blood and Marrow Transplantation. 2017 Mar 1;23(3):S381-2. | Conference abstract |
| 417 | Davis, C. We are the champions. Nursing times.2002;98(46):40-1 | Not about knowledge translation/evidence-based practice |
| 418 | Dawson A. A Practical Guide to Performance Improvement: Change Acceleration Process and Techniques to Maintain Improvements. AORN journal. 2020 Jan;111(1):97-102. | Study not about effectiveness |
| 419 | Dawson R, Lemmon K, Trivedi NJ, Hansen S. Improving human papilloma virus vaccination rates throughout military treatment facilities. Vaccine. 2018 Mar 7;36(11):1361-7. | Study not about effectiveness |
| 420 | De Sandre P, Quest T, Lawson R, Lamba S. Integrating Palliative Care in the Emergency Department: How to Jump-Start the Process (FR414). Journal of Pain and Symptom Management. 2014 Feb 1;47(2):423-4. | Conference abstract |
| 421 | Dean NC, Jones BE, Rubin JG, Vines CG, Allen TL, Srivistava R, Webb BJ. Implementation of Electronic Clinical Decision Support for Pneumonia Patients Across 17 Intermountain Emergency Departments. InD36. INNOVATIONS IN RESEARCH METHODS AND EVIDENCE SYNTHESIS 2019 May (pp. A6220-A6220). American Thoracic Society. | Conference abstract |
| 422 | Dean NC, Vines CG, Rubin J, Collingridge DS, Mankivsky M, Srivastava R, Jones BE, Kuttler KG, Walker M, Jenson N, Webb BJ. Implementation of Real-Time Electronic Clinical Decision Support for Emergency Department Patients with Pneumonia Across a Healthcare System. InAMIA Annual Symposium Proceedings 2019 (Vol. 2019, p. 353). American Medical Informatics Association. | Study not about effectiveness |
| 423 | Deasy C, Cronin M, Cahill F, Geary U, Houlihan P, Woodford M, Lecky F, Mealy K, Crowley P, Major Trauma Audit Governance Committee. Implementing major trauma audit in Ireland. Injury. 2016 Jan 1;47(1):166-72. | Study not about effectiveness |
| 424 | DeBourgh GA. Champions for evidence-based practice: a critical role for advanced practice nurses. AACN Advanced Critical Care. 2001 Nov;12(4):491-508. | Study not about effectiveness |
| 425 | Decker CJ, Gialde B, McCartan J, Chhatriwalla A, Spertus JA. Abstract P155: Implementing an Innovative Consent Form: Barriers and Successes. | Conference abstract |
| 426 | Dees J, Schrier L, Meyer M, Pettrey P, Rouillier T, Marthenze A, Munkel M, Nelson P, Thomas J. Changing Perspectives: Clabsi Reduction in a High Risk Patient Population. Biology of Blood and Marrow Transplantation. 2017 Mar 1;23(3):S480-1. | Conference abstract |
| 427 | DeForge CE, Yip NH, Dzierba AL, Ryan PG, Larson EL. Evaluation of an Educational Intervention Utilizing Nurse “Champions” and Nurses' Documentation of Intensive Care Unit Delirium. Dimensions of Critical Care Nursing. 2020 May 1;39(3):155-62. | Study not about effectiveness |
| 428 | DeGennaro R. Implementation of a Robust Intervention to Address Central Line Associated Blood Stream Infection Rates in an Acute Oncology and Stem Cell Patient Population in an Academic Setting. Biology of Blood and Marrow Transplantation. 2014 Feb 1;20(2):S299. | Conference abstract |
| 429 | DeLuca Jr LA, Walsh P, Davidson Jr DD, Stoneking LR, Yang LM, Grall KJ, Gonzaga MJ, Larson WJ, Stolz U, Sabb DM, Denninghoff KR. Impact and feasibility of an emergency department–based ventilator-associated pneumonia bundle for patients intubated in an academic emergency department. American journal of infection control. 2017 Feb 1;45(2):151-7. | Study not about effectiveness |
| 430 | Demes JA, Nickerson N, Farand L, Montekio VB, Torres P, Dube JG, Coq JG, Pomey MP, Champagne F, Jasmin ER. What are the characteristics of the champion that influence the implementation of quality improvement programs?. Evaluation and program planning. 2020 Jun 1;80:101795. | Study not about effectiveness |
| 431 | Denham CR, Angood P, Berwick D, Binder L, Clancy CM, Corrigan JM, Hunt D. Chasing zero: can reality meet the rhetoric?. Journal of patient safety. 2009 Dec 1;5(4):216-22. | Study not about effectiveness |
| 432 | Denham CR, Angood P, Berwick D, Binder L, Clancy CM, Corrigan JM, Hunt D. The chasing zero department: making idealized design a reality. Journal of patient safety. 2009 Dec 1;5(4):210-5. | Study not about effectiveness |
| 433 | Dennis A, Fuentes L, Douglas‐Durham E, Grossman D. Barriers to and facilitators of moving miscarriage management out of the operating room. Perspectives on sexual and reproductive health. 2015 Sep;47(3):141-9. | Study not about effectiveness |
| 434 | Dennison RD. A medication safety education program to reduce the risk of harm caused by medication errors. The Journal of Continuing Education in Nursing. 2007 Jul 1;38(4):176-84. | Study not about effectiveness |
| 435 | Denomme LB, Terry AL, Brown JB, Thind A, Stewart M. Primary health care teams’ experience of electronic medical record use after adoption. Fam Med. 2011 Oct 1;43(9):638-42. | Study not about effectiveness |
| 436 | Deshmukh U, Oliveira CR, Griggs S, Coleman E, Avni-Singer L, Pathy S, Shapiro ED, Sheth SS. Impact of a clinical interventions bundle on uptake of HPV vaccine at an OB/GYN clinic. Vaccine. 2018 Jun 14;36(25):3599-605. | Study not about effectiveness |
| 437 | DeSisto CL, Kroelinger CD, Estrich C, Velonis A, Uesugi K, Goodman DA, Pliska E, Akbarali S, Rankin KM. Application of an implementation science framework to policies on immediate postpartum long-acting reversible contraception. Public Health Reports. 2019 Mar;134(2):189-96. | Study not about effectiveness |
| 438 | DeSouza K, Pit SW, Moehead A. Translating facilitated multimodal online learning into effective person-centred practice for the person living with dementia among health care staff in Australia: an observational study. BMC geriatrics. 2020 Dec;20(1):1-4. | Study not about effectiveness |
| 439 | DeVon HA, Patmon FL, Rosenfeld AG, Fennessy MM, Francis D. Implementing clinical research in the high acuity setting of the emergency department. Journal of Emergency Nursing. 2013 Jan 1;39(1):6-12. | Study not about effectiveness |
| 440 | DeWitt E.M.,Passo M., Kimura Y., Beukelman T., Gottlieb B.S., Margolis P. Pediatric rheumatology improvement network for clinical excellence and safety - PRINCES. Arthritis and Rheumatism / 2009;60(SUPPL. 10):1888 | Conference abstract |
| 441 | Dharmarajan L. Abstract P128: Get With the Guidelines Coronary Artery Disease Program Helps Attain Quality Care in Minority Population: 5 Year Experience in the Inner City Bronx. | Conference abstract |
| 442 | Di Prospero LS, Robson S, Gupta TD, Bristow B, Peacock M, O'Leary B. Leading Practice: Integrating Best Practice Initiatives as Part of the Radiation Therapist Role. Journal of Medical Imaging and Radiation Sciences. 2014 Jun 1;45(2):164-5. | Conference abstract |
| 443 | Di Ruggiero E, Kishchuk N, Viehbeck S, Edwards N, Robinson K, Riley B, Fowler HS. Alliance members’ roles in collective field-building: an assessment of leadership and championship within the Population Health Intervention Research Initiative for Canada. Health research policy and systems. 2017 Dec;15(1):1-1. | Not within a health care setting |
| 444 | Diamond E, French K, Gronkiewicz C, Borkgren M. Electronic medical records: a practitioner's perspective on evaluation and implementation. Chest. 2010 Sep 1;138(3):716-23. | Study not about effectiveness |
| 445 | Dickerman M, O'Brien J, Levy C. Creation of and Experience with the End of Life Plan of Care in the EMR: A Quality Improvement Project in the Pediatric Intensive Care Unit (QI647). Journal of Pain and Symptom Management. 2020 Feb 1;59(2):525. | Conference abstract |
| 446 | Didier D, Pace M, Walker WV. Lessons learned with MS-DRGs getting physicians on board for success. Healthcare Financial Management. 2008 Aug 1;62(8):38-43. | Study not about effectiveness |
| 447 | Diffin J, Ewing G, Grande G. Using Normalisation Process Theory (NPT) to Inform an Implementation Toolkit for a Carer-centred Process of Assessment and Support within Palliative Care. In10th World Research Congress of the EAPC 2018 Feb 2. | Conference abstract |
| 448 | DiLibero J, DeSanto-Madyea S, O’Dongohue S. Improving accuracy of cardiac electrode placement: outcomes of clinical nurse specialist practice. Clinical Nurse Specialist. 2016 Jan 1;30(1):45-50. | Duplicate |
| 449 | DiLibero J, DeSanto-Madyea S, O’Dongohue S. Improving accuracy of cardiac electrode placement: outcomes of clinical nurse specialist practice. Clinical Nurse Specialist. 2016 Jan 1;30(1):45-50. | Study not about effectiveness |
| 450 | Dillon JA, Chiappe M, Martin I, Tinajeros F. CONTROLLING THE SPREAD OF ANTIMICROBIAL RESISTANCE IN NEISSERIA GONORRHOEAE IN LATIN AMERICA AND THE CARIBBEAN-THE GONOCOCCAL ANTIMICROBIAL SUSCEPTIBILITY PROGRAM (GASP). InSEXUALLY TRANSMITTED DISEASES 2014 Jun 1 (Vol. 41, pp. S28-S29). TWO COMMERCE SQ, 2001 MARKET ST, PHILADELPHIA, PA 19103 USA: LIPPINCOTT WILLIAMS & WILKINS. | Conference abstract |
| 451 | Dilworth K, Tao M, Shapiro S, Timmings C. Making health promotion evidenced-informed: an organizational priority. Health promotion practice. 2013 Jan;14(1):139-45. | Study not about effectiveness |
| 452 | Dimopoulos-Bick T, Osten R, Shipway C, Trevena L, Hoffmann T. Shared decision making implementation: a case study analysis to increase uptake in New South Wales. Australian Health Review. 2019 Jan 31;43(5):492-9. | Study not about effectiveness |
| 453 | Dimoska A, Butow PN, Lynch J, Hovey E, Agar M, Beale P, Tattersall MH. Implementing patient question-prompt lists into routine cancer care. Patient education and counseling. 2012 Feb 1;86(2):252-8. | Study not about effectiveness |
| 454 | Diner BM, Carpenter CR, O'Connell T, Pang P, Brown MD, Seupaul RA, Celentano JJ, Mayer D, KT‐CC Theme IIIa Members. Graduate medical education and knowledge translation: role models, information pipelines, and practice change thresholds. Academic Emergency Medicine. 2007 Nov;14(11):1008-14. | Study not about effectiveness |
| 455 | Dobbins M, Greco L, Yost J, Traynor R, Decorby-Watson K, Yousefi-Nooraie R. A description of a tailored knowledge translation intervention delivered by knowledge brokers within public health departments in Canada. Health research policy and systems. 2019 Dec;17(1):1-8. | Study not about effectiveness |
| 456 | Dobish R., Chambers C., Shultz J. Development and implementation of embedded checklists into chemotherapy preparation worksheets. Journal of Oncology Pharmacy Practice / 2014;20(3 SUPPL. 1):25 | Conference abstract |
| 457 | Dobra SE, Baker N, Miller L, Stokes L. Programmatic implementation of hepatocellular carcinoma prevention through hepatitis C testing, secondary prevention, and treatment for a medically and minority underserved population. InCANCER EPIDEMIOLOGY BIOMARKERS & PREVENTION 2016 Mar 1 (Vol. 25, No. 3). 615 CHESTNUT ST, 17TH FLOOR, PHILADELPHIA, PA 19106-4404 USA: AMER ASSOC CANCER RESEARCH. | Conference abstract |
| 458 | Dolansky MA, Hitch JA, Piña IL, Boxer RS. Improving heart failure disease management in skilled nursing facilities: lessons learned. Clinical nursing research. 2013 Nov;22(4):432-47. | Study not about effectiveness |
| 459 | Dolgowicz C, Maynard M. CREATING A REGIONALIZED LUNG HEALTH PROGRAM. Canadian Journal of Respiratory Therapy. 2017 Mar 1;53(2). | Conference abstract |
| 460 | Donaldson SR, Harding AM, Taylor SE, Vally H, Greene SL. Evaluation of a targeted prescriber education intervention on emergency department discharge oxycodone prescribing. Emergency Medicine Australasia. 2017 Aug;29(4):400-6. | Study not about effectiveness |
| 461 | Dong K, Vandenberghe C, Kirkland S, Cummings GG, McKim R, Taylor M, Wild CT. Effect of a knowledge translation intervention on physician screening, brief intervention, and referral to treatment behaviour in a socioeconomically disadvantaged setting. Canadian Journal of Addiction. 2015 Apr 1;6(1):7-14. | Study not about effectiveness |
| 462 | Doolittle T. Avoiding hospital-acquired conditions: A qualitative analysis of early top performers. University of San Diego; 2016. | Study not about effectiveness |
| 463 | Dorsey S, Berliner L, Lyon AR, Pullmann MD, Murray LK. A statewide common elements initiative for children’s mental health. The Journal of Behavioral Health Services & Research. 2016 Apr 1;43(2):246-61. | Study not about effectiveness |
| 464 | Dowrick C, Chew-Graham C, Lovell K, Lamb J, Aseem S, Beatty S, Bower P, Burroughs H, Clarke P, Edwards S, Gabbay M. Increasing equity of access to high-quality mental health services in primary care: a mixed-methods study. | Conference abstract |
| 465 | Doyle, M.,Coughlan, T.The Fall Champion Toolbox. Age & Ageing Supplement 2019;48():iii17-iii65 | Conference abstract |
| 466 | Drake C, Kirk JK, Buse JB, Edelman D, Shea CM, Spratt S, Young LA, Kahkoska AR. Characteristics and Delivery of Diabetes Shared Medical Appointments in North Carolina. North Carolina medical journal. 2019 Sep 1;80(5):261-8. | Study not about effectiveness |
| 467 | Drake J, Redfern WS, Sherburne E, Nugent ML, Simpson P. Pediatric skin care: What do nurses really know?. Journal for Specialists in Pediatric Nursing. 2012 Oct;17(4):329-38. | Study not about effectiveness |
| 468 | Draper DA, Felland LE, Liebhaber A, Melichar L. The role of nurses in hospital quality improvement. Research brief. 2008 Mar 1(3):1-8. | Study not about effectiveness |
| 469 | Drew S, Gooberman-Hill R, Farmer A, Graham L, Javaid MK, Cooper C, Judge A. Making the case for a fracture liaison service: a qualitative study of the experiences of clinicians and service managers. BMC musculoskeletal disorders. 2015 Dec;16(1):1-8. | Study not about effectiveness |
| 470 | Drew S, Judge A, Cohen R, Fitzpatrick R, Barker K, Gooberman-Hill R. Enhanced Recovery After Surgery implementation in practice: an ethnographic study of services for hip and knee replacement. BMJ open. 2019 Mar 1;9(3):e024431. | Study not about effectiveness |
| 471 | Dryden-Palmer K, Middaugh K, Parshuram C. ENHANCING IMPLEMENTATION: IDENTIFYING INFLUENTIAL FACTORS IN THE ADOPTION OF COMPLEX CLINICAL INTERVENTIONS. Pediatric Critical Care Medicine. 2014 May 1;15(4_suppl):201-2. | Conference abstract |
| 472 | Dubb R, Nydahl P, Hermes C, Schwabbauer N, Toonstra A, Parker AM, Kaltwasser A, Needham DM. Barriers and strategies for early mobilization of patients in intensive care units. Annals of the American Thoracic Society. 2016 May;13(5):724-30. | Letters to the editor/ Review studies |
| 473 | DuBose-Morris R. An interpretative phenomenological analysis of telehealth champions (Doctoral dissertation, Nova Southeastern University). | Study not about effectiveness |
| 474 | Dudgeon D, King S, Howell D, Green E, Gilbert J, Hughes E, Lalonde B, Angus H, Sawka C. Cancer Care Ontario's experience with implementation of routine physical and psychological symptom distress screening. Psycho‐Oncology. 2012 Apr;21(4):357-64. | Study not about effectiveness |
| 475 | Duggal S, Flics S, Cornell CN. Introduction of clinical pathways in orthopedic surgical care: the experience of the hospital for special surgery. InPerioperative Care of the Orthopedic Patient 2014 (pp. 365-371). Springer, New York, NY. | Study not about effectiveness |
| 476 | Duggan C. How the Society is championing best practice in education and development. Pharmaceutical Journal. 2012;288(7693):208-209 | Letters to the editor/ Review studies |
| 477 | Dukelow S, Reimer EA. The implementation of SCORE recommendations to prevent hemiplegic shoulder pain during inpatient stroke rehabilitation. InSTROKE 2012 Nov 1 (Vol. 43, No. 11, pp. E120-E120). 530 WALNUT ST, PHILADELPHIA, PA 19106-3621 USA: LIPPINCOTT WILLIAMS & WILKINS. | Conference abstract |
| 478 | Dumyati G, Concannon C, Shelly M. The Challenges in Implementing a Central Line Related Bloodstream Infection (CLABSI) Prevention Outside the ICU through a Multihospital Collaborative. American Journal of Infection Control. 2011 Jun 1;39(5):E138. | Conference abstract |
| 479 | Dunbar P. Nursing Care for Terminal Patients in Intensive Care Units (Doctoral dissertation, Walden University). | Study not about effectiveness |
| 480 | Dunkley-Bent J. Maternity safety champions. British Journal of Midwifery. 2018 Nov 2;26(11):698-. | Study not about effectiveness |
| 481 | Dunning, J. 'Let's make dementia care personal'. Community Care 2009;(1790):28 | Study not about effectiveness |
| 482 | Duquaine D, Farley SM, Sacks R, Mandel-Ricci J, Silfen SL, Shih SC. Designing a quality improvement program with electronic health records: New York City’s Health eQuits. American Journal of Medical Quality. 2015 Mar;30(2):141-8. | Study not about effectiveness |
| 483 | Dvaladze A, Duggan C, Gralow JR, Anderson BO. Breast Cancer Initiative 2.5 (BCI2. 5): A global campaign to reduce disparities in breast cancer outcomes. | Conference abstract |
| 484 | Dyer E, Wong S, Cramp S, Whitwell K. G216 (P) Paediatric Pain Scoring in A&E–A Quality Improvement Project. | Conference abstract |
| 485 | Dykes PC, Duckworth M, Cunningham S, Dubois S, Driscoll M, Feliciano Z, Ferrazzi M, Fevrin FE, Lyons S, Lindros ME, Monahan A. Pilot testing fall TIPS (tailoring interventions for patient safety): a patient-centered fall prevention toolkit. The Joint Commission Journal on Quality and Patient Safety. 2017 Aug 1;43(8):403-13. | Study not about effectiveness |
| 486 | Dziedzic K, Bierma-Zeinstra S, Vlieland TV, Roos EM, Skou ST, Hagen KB, Osteras N, Pais S, Cordeiro C, Duffy H, Hughes R. Joint implementation of guidelines for osteoarthritis in Western Europe: JIGSAW-E. Physiotherapy. 2016 Nov 1;102:e138-9. | Conference abstract |
| 487 | Earp M., Simon J., Marc K., Sinnarajah A., Martopullo C. Palliative care, early & systematic (PACES) project: Impact on patient & health systems outcomes. Supportive Care in Cancer / 2018;26(2 Supplement 1):S199 | Conference abstract |
| 488 | Eckstrom E, Lasater K, Cotrell V, Simonson W, Neal M, Harvath T. C79: Interprofessional, Evidence-Based Education to Reduce Falls. Journal of the American Geriatrics Society. 2012 Apr;60. | Conference abstract |
| 489 | Edelson DP, Yuen TC, Mancini ME, Davis DP, Hunt EA, Miller JA, Abella BS. Hospital cardiac arrest resuscitation practice in the United States: a nationally representative survey. Journal of hospital medicine. 2014 Jun;9(6):353-7. | Study not about effectiveness |
| 490 | Edwards HE, Chang AM, Gibb M, Finlayson KJ, Parker C, O'Reilly M, McDowell J, Shuter P. Reduced prevalence and severity of wounds following implementation of the Champions for Skin Integrity model to facilitate uptake of evidence‐based practice in aged care. Journal of clinical nursing. 2017 Dec 1;26(23-24):4276-85. | Study not about effectiveness |
| 491 | Eilbacher J, Branas A, Caviston S, Devirgilio K, Ryninger M, Rheingold S. Improving the patient and family experience: physical therapy within the oncology clinic. Archives of Physical Medicine and Rehabilitation. 2017 Oct 1;98(10):e75. | Conference abstract |
| 492 | Ellard DR, Thorogood M, Underwood M, Seale C, Taylor SJ. Whole home exercise intervention for depression in older care home residents (the OPERA study): a process evaluation. BMC medicine. 2014 Dec;12(1):1-1. | Study not about effectiveness |
| 493 | Ellimoottil C, An L, Moyer M, Sossong S, Hollander JE. Challenges and opportunities faced by large health systems implementing telehealth. Health Affairs. 2018 Dec 1;37(12):1955-9. | Study not about effectiveness |
| 494 | Elliot DL, Kuehl KS, Goldberg L, DeFrancesco CA, Moe EL. Worksite health promotion in six varied US sites: beta testing as a needed translational step. Journal of environmental and public health. 2011 Apr 7;2011. | Study not about effectiveness |
| 495 | Ellis I. The clinical champion role in the development of a successful telehealth wound care project for remote Australia. Journal of telemedicine and telecare. 2005 Dec;11(2_suppl):26-8. | Study not about effectiveness |
| 496 | Ellis JA, McCleary L, Blouin R, Dube K, Rowley B, MacNeil M, Cooke C. Implementing best practice pain management in a pediatric hospital. Journal for Specialists in Pediatric Nursing. 2007 Oct;12(4):264-77. | Study not about effectiveness |
| 497 | Ellis, R.Investigate the extrinsic and intrinsic motivational factors of knowledge transfer in the hospitals. Solid State Technology 2020;63(1):1-8 | No full text available |
| 498 | Emerick M, Standiford H, McQuillan K, Von Rueden K, Hebden J. Reduction of CLABSI in the Trauma Population. American Journal of Infection Control. 2011 Jun 1;39(5):E50. | Conference abstract |
| 499 | Empey PE, Stevenson JM, Tuteja S, Weitzel KW, Angiolillo DJ, Beitelshees AL, Coons JC, Duarte JD, Franchi F, Jeng LJ, Johnson JA. Multisite investigation of strategies for the implementation of CYP2C19 genotype‐guided antiplatelet therapy. Clinical Pharmacology & Therapeutics. 2018 Oct;104(4):664-74. | Study not about effectiveness |
| 500 | Engel HJ, Needham DM, Morris PE, Gropper MA. ICU early mobilization: from recommendation to implementation at three medical centers. Critical care medicine. 2013 Sep 1;41(9):S69-80. | Study not about effectiveness |
| 501 | Engel HJ, Tatebe S, Alonzo PB, Mustille RL, Rivera MJ. Physical therapist–established intensive care unit early mobilization program: quality improvement project for critical care at the University of California San Francisco Medical Center. Physical therapy. 2013 Jul 1;93(7):975-85. | Study not about effectiveness |
| 502 | Ensor T, Clapham S, Prasai DP. What drives health policy formulation: Insights from the Nepal maternity incentive scheme?. Health policy. 2009 May 1;90(2-3):247-53. | Not about knowledge translation/evidence-based practice |
| 503 | Erikson M, Smith PD, Sparks SW. Best practices to advance health literacy in a Wisconsin adult literacy coalition. HLRP: Health Literacy Research and Practice. 2019 Jul 1;3(3):S8-14. | Not about knowledge translation/evidence-based practice |
| 504 | Erin Ferguson RN M, Phyllis Montgomery RN, Sharolyn Mossey RN M, Craig Duncan RN. Non-pharmacological prevention of delirium in older adults living in long-term care homes: A scoping review of nursing interventions. Perspectives. 2018;40(2):19-27. | Letters to the editor/ Review studies |
| 505 | Escoffery C, Riehman K, Watson L, Priess AS, Borne MF, Halpin SN, Rhiness C, Wiggins E, Kegler MC. Facilitators and barriers to the implementation of the HPV VACs (Vaccinate Adolescents Against Cancers) program: a consolidated framework for implementation research analysis. Preventing chronic disease. 2019 Jul 3;16:E85. | Study not about effectiveness |
| 506 | Eskicioglu C, Pearsall E, Victor JC, Aarts MA, Okrainec A, McLeod RS. A multifaceted knowledge translation strategy can increase compliance with guideline recommendations for mechanical bowel preparation. Journal of Gastrointestinal Surgery. 2015 Jan 1;19(1):39-45. | Study not about effectiveness |
| 507 | Esplen M, Green E, Hunter J, Mcleod D, Campbell M, Clarke S, Wong J. Addressing Psychosocial Needs In Cancer Care-A Train The Trainer Workshop For Oncology Nurses: S1861-1. Psycho-oncology. 2010 May;19. | Conference abstract |
| 508 | Estep B, Kaminski B. Optimal Teams and Performance Feedback Drive Improvements in Processing Measures. ED management: the monthly update on emergency department management. 2016 Sep 1;28(9):104-7. | Study not about effectiveness |
| 509 | Etz RS, Keith RE, Maternick AM, Stein KL, Sabo RT, Hayes MS, Sevak P, Holland J, Crosson JC. Supporting Practices to Adopt Registry-Based Care (SPARC): protocol for a randomized controlled trial. Implementation Science. 2015 Dec;10(1):1-9. | Letters to the editor/ Review studies |
| 510 | Evans B.J., Shoemaker M.E2B: Integrating emergency medical services into the chest pain center. Critical Pathways in Cardiology / 2010;9(3):178-179 | Conference abstract |
| 511 | Evans C., Shouls S., Launchbury L., Yorganci E., Carey I.,Hopper A., Murtagh F.E.M., Gao W., Yi D., Pickles A., Barclay S., Higginson I.J., Koffman J.Reducing variability in implementation of 'new' models of care in clinical trials; Developing and testing benchmark criteria to examine if key intervention mechanisms are embedded in practice. Palliative Medicine / 2018;32(1 Supplement 1):219 | Conference abstract |
| 512 | Evans D, Hartung DM, Beasley D, Fagnan LJ. Breaking up is hard to do: lessons learned from a pharma-free practice transformation. The Journal of the American Board of Family Medicine. 2013 May 1;26(3):332-8. | Study not about effectiveness |
| 513 | Evans DC, Nichol WP, Perlin JB. Effect of the implementation of an enterprise-wide Electronic Health Record on productivity in the Veterans Health Administration. Health Economics, Policy and Law. 2006 Apr;1(2):163-9. | Study not about effectiveness |
| 514 | Evans W, Peter A, Truscott R, Cameron E, Schwartz N, Haque M, Bassier-Paltoo M, Khan S, Giuliani M. MA 18.01 Driving Improvements in Cancer Care Ontario's Smoking Cessation Initiative for Cancer Patients in Ontario, Canada. Journal of Thoracic Oncology. 2017 Nov 1;12(11):S1877. | Conference abstract |
| 515 | Evans W, Truscott R, Cameron E, Timmings C, Haque M, Halligan M, Rana S, Keen D, Rabeneck L. ES20. 03 Tobacco Control Integration in Cancer Care: The Canadian Experience. Journal of Thoracic Oncology. 2019 Oct 1;14(10):S62. | Conference abstract |
| 516 | Evans WK, Truscott R, Cameron E, Rana S, Isaranuwatchai W, Haque M, Rabeneck L. Implementing smoking cessation within cancer treatment centres and potential economic impacts. Translational lung cancer research. 2019 May;8(Suppl 1):S11. | Study not about effectiveness |
| 517 | Everall AC. Best Practice MedsCheck Annual Service: A Multi-Case Study (Doctoral dissertation, University of Toronto (Canada)). | Study not about effectiveness |
| 518 | Ewing G, Austin L, Grande G. Challenges to implementing comprehensive carer assessment in palliative home care: Findings from a cluster trial. BMJ Supportive & Palliative Care. 2014 Mar 1;4(1):115-. | Conference abstract |
| 519 | Ewing G., Grande G.How do carers feature in end of life care policy? Scoping and narrative summary of UK national policy/guidelines on implementing person-centred carer assessment and support. Palliative Medicine / 2018;32(1 Supplement 1):182-183 | Conference abstract |
| 520 | Ewing G., Grande G.What structures and processes need to be in place to enable person-centred assessment and support for carers during end of life care? A multi-perspective, mixed methods study. Palliative Medicine .2018;32(1 Supplement 1):117 | Conference abstract |
| 521 | Eyles JP, Bowden JL, Redman S, Redman A, Dawson G, Newell S, Williams M, Ferreira M, Foster K, Wang X, Melo L. Barriers and enablers to the implementation of the Australian Osteoarthritis Chronic Care Program (OACCP). Osteoarthritis and Cartilage. 2020 Apr 1;28:S446. | Conference abstract |
| 522 | Fabre V, Pleiss A, Klein E, Demko Z, Salinas A, Jones G, Gadala A, Hicks LA, Neuhauser MM, Srinivasan A, Cosgrove SE. A pilot study to evaluate the impact of a nurse-driven urine culture diagnostic stewardship intervention on urine cultures in the acute care setting. The Joint Commission Journal on Quality and Patient Safety. 2020 Nov 1;46(11):650-5. | Study not about effectiveness |
| 523 | Fabro M. Stroke Management: Dysphagia Screening Program (Doctoral dissertation, [Honolulu]:[University of Hawaii at Manoa],[December 2016]). | Study not about effectiveness |
| 524 | Factiva.Oncology; New Findings from Samuel Lunenfeld Research Institute in the Area of Rectal Cancer Described Obesity, Fitness & Wellness Week 2014;():4038 | Conference abstract |
| 525 | Fagnan LJ, Dorr DA, Davis M, McGinnis P, Mahler J, King MM, Michaels L. Turning on the care coordination switch in rural primary care: voices from the practices—clinician champions, clinician partners, administrators, and nurse care managers. The Journal of ambulatory care management. 2011 Jul;34(3):304. | Study not about effectiveness |
| 526 | Fairchild E, Roberts L, Zelman K, Michelli S, Hastings-Tolsma M. Implementation of Robert's Coping with Labor Algorithm© in a large tertiary care facility. Midwifery. 2017 Jul 1;50:208-18. | Study not about effectiveness |
| 527 | Fajardo S, Goralski JL, Leandro J, Taylor MF, Berckmans S, Prieur M, Buchanan M, Jones BW, Sangvai A, Rock M, McGibbon L. QUALITY IMPROVEMENT IN TRANSITION: THE EFFECT OF A TRANSITION COORDINATOR. InPEDIATRIC PULMONOLOGY 2017 Sep 1 (Vol. 52, pp. S436-S437). 111 RIVER ST, HOBOKEN 07030-5774, NJ USA: WILEY. | Conference abstract |
| 528 | Fakih MG, Heavens M, Grotemeyer J, Szpunar SM, Groves C, Hendrich A. Avoiding potential harm by improving appropriateness of urinary catheter use in 18 emergency departments. Annals of emergency medicine. 2014 Jun 1;63(6):761-8. | Study not about effectiveness |
| 529 | Fakih MG, Krein SL, Edson B, Watson SR, Battles JB, Saint S. Engaging health care workers to prevent catheter-associated urinary tract infection and avert patient harm. American journal of infection control. 2014 Oct 1;42(10):S223-9. | Study not about effectiveness |
| 530 | Falkman G, Gustafsson M, Jontell M, Torgersson O. SOMWeb: a semantic web-based system for supporting collaboration of distributed medical communities of practice. Journal of medical Internet research. 2008;10(3):e25. | Study not about effectiveness |
| 531 | Fan T, Sharma A, Charkin E. G513 The rhino project, a quality improvement project targeting early detection and referral of neonates with hypoglycaemia. | Conference abstract |
| 532 | Fanucchi L, Logio LS, Siegler E. INNOVATIONS IN MEDICAL EDUCATION (IME) A CLER LOOK AT MORBIDITY AND MORTALITY CONFERENCES. InJOURNAL OF GENERAL INTERNAL MEDICINE 2013 Jun 1 (Vol. 28, pp. S450-S450). 233 SPRING ST, NEW YORK, NY 10013 USA: SPRINGER. | Conference abstract |
| 533 | Farley K, Hanbury A, Thompson C. Gathering opinion leader data for a tailored implementation intervention in secondary healthcare: a randomised trial. BMC medical research methodology. 2014 Dec;14(1):1-7. | Study not about effectiveness |
| 534 | Farrell JJ, Petrik SC. Hydration and nosocomial pneumonia: killing two birds with one stone (a toothbrush). Rehabilitation Nursing. 2009 Mar 4;34(2):47-50. | Study not about effectiveness |
| 535 | Farrington M. Infection control education: how to make an impact–tools for the job. Journal of Hospital Infection. 2007 Jun 1;65:128-32. | Not a champion |
| 536 | Febre Viera LM, Warren J, Faruqi I. Impact Of A Clinical Leader (CL) On Patient Quality Outcomes In A Rapidly Created Stand Alone ICU Within An IMC Unit. InA39. IMPROVING QUALITY IN THE INTENSIVE CARE UNIT 2012 May (pp. A1454-A1454). American Thoracic Society. | Conference abstract |
| 537 | Fee JP, Sonia Trepina MP, Jennifer Boles CP, Sparks J. Focus on Population Health CDI Generates ACO Shared Savings. | Study not about effectiveness |
| 538 | Feinberg J, Flynn L, Woodward M, Pennell C, Higham H, Morgan L, Holman L, Tully P, McCulloch P. Improving emergency surgical care for patients with right iliac fossa pain at a regional scale: A quality improvement study using the Supported Champions implementation strategy. International Journal of Surgery. 2018 Sep 1;57:105-10. | Study not about effectiveness |
| 539 | Feld L, Belfer J, Kabra R, Buchan M, Castiglione J, Sayres S, Barone S. 106. UTILITY OF A QUALITY DASHBOARD ESTABLISHED BY A RESIDENT QUALITY COUNCIL. Academic Pediatrics. 2020 Sep 1;20(7):e50-1. | Conference abstract |
| 540 | Fenge LA, Jones K, Gibson C. Meaningful dissemination produces the “long tail” that engenders community impact. Qualitative Research Journal. 2018 Feb 5. | Not about knowledge translation/evidence-based practice |
| 541 | Ferlie E, Bennett C. Patterns of strategic change in health care: district health authorities respond to aids. British Journal of Management. 1992 Mar;3(1):21-37. | Not about knowledge translation/evidence-based practice |
| 542 | Fernald DH, Jortberg BT, Hessler DM, Wearner R, Dickinson LM, Fisher L, Dickinson WP. Recruiting primary care practices for research: Reflections and reminders. The Journal of the American Board of Family Medicine. 2018 Nov 1;31(6):947-51. | Study not about effectiveness |
| 543 | Fernandez MA. FERNANDEZ’STROKE: HYPERTENSION AND HYPOTENSION AND CARDIOVASCULAR RESOLUTIONS. Journal of Hypertension. 2018 Jun 1;36:e107-8. | Conference abstract |
| 544 | Filardo G, Nicewander D, Herrin J, Edwards J, Galimbertti P, Tietze M, Mcbride S, Gunderson J, Collinsworth A, Haydar Z, Williams J. A hospital-randomized controlled trial of a formal quality improvement educational program in rural and small community Texas hospitals: one year results. International Journal for Quality in Health Care. 2009 Aug 1;21(4):225-32. | Study not about effectiveness |
| 545 | Filly R. Ultrasound needs vocal champions to move research into clinical use. Diagnostic Imaging. 2000;25:32. | Letters to the editor/ Review studies |
| 546 | Fine PG, Bradshaw DH, Cohen MJ, Connor SR, Donaldson G, Gharibo C, Gidal BE, Muir JC, Tselentis HN. Evaluation of the performance improvement CME paradigm for pain management in the long-term care setting. Pain Medicine. 2014 Mar 1;15(3):403-9. | Study not about effectiveness |
| 547 | Fineout-Overholt E, Levin RF, Melnyk BM. Strategies for advancing evidence-based practice in clinical settings. JNY State Nurses Assoc. 2004 Sep 1;35(2):28-32. | Study not about effectiveness |
| 548 | Fineout-Overholt E, Melnyk BM, Schultz A. Transforming health care from the inside out: advancing evidence-based practice in the 21st century. Journal of professional nursing. 2005 Nov 1;21(6):335-44. | Study not about effectiveness |
| 549 | Fink RM, Somes E, Brackett H, Shanbhag P, Anderson AN, Lum HD. Evaluation of quality improvement initiatives to improve and sustain advance care planning completion and documentation. Journal of hospice and palliative nursing: JHPN: the official journal of the Hospice and Palliative Nurses Association. 2019 Feb;21(1):71. | Study not about effectiveness |
| 550 | Finucane AM, Stevenson B, Moyes R, Oxenham D, Murray SA. Improving end-of-life care in nursing homes: implementation and evaluation of an intervention to sustain quality of care. Palliative medicine. 2013 Sep;27(8):772-8. | Study not about effectiveness |
| 551 | Fisher D, Michaels J, Hase R, Zhang J, Kataria S, Sim B, Tsang JK, Pollard J, Chan M, Swaminathan S. Outpatient parenteral antibiotic therapy (OPAT) in Asia: missing an opportunity. Journal of Antimicrobial Chemotherapy. 2017 Apr 1;72(4):1221-6. | Conference abstract |
| 552 | Fitchett G, Hoffmeyer C, Labuschagne D, Lee A, Pierson AL, Pugliese K, Levine S. A Quantifiable Spiritual Assessment Model in Palliative Care: Putting Two and Two Together for Improved Spiritual Care (TH320). Journal of Pain and Symptom Management. 2019 Feb 1;57(2):374. | Conference abstract |
| 553 | Fitzgerald N., Gabehart K.,Roggy D., Sood R.Implementation of a new practices and interventions and their impact on central line associated blood stream infections. Journal of Burn Care and Research / 2016;37(SUPPL. 1):S148 | Conference abstract |
| 554 | Fitzsimons L. The role of champions in promoting family focused practice across adult mental health and children's services. Advances in Mental Health. 2020 Sep 1;18(3):251-60. | Study not about effectiveness |
| 555 | Flanagan ME, Plue L, Miller KK, Schmid AA, Myers L, Graham G, Miech EJ, Williams LS, Damush TM. A qualitative study of clinical champions in context: Clinical champions across three levels of acute care. SAGE open medicine. 2018 Jan;6:2050312118792426. | Study not about effectiveness |
| 556 | Flanagan T, Avalos LA. Perinatal obstetric office depression screening and treatment: implementation in a health care system. Obstetrics and gynecology. 2016 May;127(5):911. | Study not about effectiveness |
| 557 | Flanigan C, Davidson A, Perumalswami PV, Weiss J, Schwartz J. A Survey of the Hepatitis C Infrastructure and Technical Assistance Needs of New York State Community Health Centers. InHEPATOLOGY 2018 Oct 1 (Vol. 68, pp. 286A-287A). 111 RIVER ST, HOBOKEN 07030-5774, NJ USA: WILEY. | Conference abstract |
| 558 | Fleet R, Dupuis G, Fortin JP, Gravel J, Ouimet M, Poitras J, Légaré F. Rural emergency care 360: mobilising healthcare professionals, decision-makers, patients and citizens to improve rural emergency care in the province of Quebec, Canada: a qualitative study protocol. BMJ open. 2017 Aug 1;7(8):e016039. | Conference abstract |
| 559 | Fleming B, Silver A, Ocepek-Welikson K, Keller D. The relationship between organizational systems and clinical quality in diabetes care. Am J Manag Care. 2004 Dec 1;10(12):934-44. | Study not about effectiveness |
| 560 | Flink E, Kilburn Jr H, Morley J, Wang T, Panzer R. Using process measures to improve patient safety practices to prevent pulmonary embolism. Advances in Patient Safety: New Directions and Alternative Approaches (Vol. 3: Performance and Tools). 2008 Aug. | Study not about effectiveness |
| 561 | Floyd L, Bryce F, Ramaswamy R, Olufolabi A, Srofenyoh E, Goodman D, Pearson N, Morgan K, Tetteh C, Ahwireng V, Owen M. The introduction of a midwife-led obstetric triage system into a regional referral hospital in Ghana. Midwifery. 2018 Jun 1;61:45-52. | Study not about effectiveness |
| 562 | Floyd N. Increasing the pace of evidence gathering. British Journal of Occupational Therapy. 2011 Jul 1;74(7):362-3. | Letters to the editor/ Review studies |
| 563 | Flynn ER. We're Not in ICU Anymore: Long-term Care and Dialysis Units Collaborate on Process Improvement to Reduce Central Line-Associated Bloodstream Infections. American Journal of Infection Control. 2016 Jun 2;44(6):S47-8. | Conference abstract |
| 564 | Foley E., Meyer S. Improving detection and management of high blood pressure public health and health care sharing common goals. Journal of Clinical Hypertension. 2012;14(SUPPL. 1): | Conference abstract |
| 565 | Foley KL, Pockey JR, Helme DW, Song EY, Stewart K, Jones C, Spangler JG, Sutfin EL. Integrating evidence-based tobacco cessation interventions in free medical clinics: opportunities and challenges. Health promotion practice. 2012 Sep;13(5):687-95. | Study not about effectiveness |
| 566 | Fontaine P, Whitebird R, Solberg LI, Tillema J, Smithson A, Crabtree BF. Minnesota’s early experience with medical home implementation: viewpoints from the front lines. Journal of general internal medicine. 2015 Jul;30(7):899-906. | Study not about effectiveness |
| 567 | Forbes R. Advance care planning champions, a Canadian approach to navigating end-of-life care. InJOURNAL OF PSYCHOSOCIAL ONCOLOGY 2016 Mar 3 (Vol. 34, No. 1-2, pp. 139-140). 4 PARK SQUARE, MILTON PARK, ABINGDON OX14 4RN, OXFORDSHIRE, ENGLAND: ROUTLEDGE JOURNALS, TAYLOR & FRANCIS LTD. | Conference abstract |
| 568 | Forchuk C, Martin ML, Jensen E, Ouseley S, Sealy P, Beal G, Reynolds W, Sharkey S. Integrating an evidence‐based intervention into clinical practice:‘Transitional relationship model’. Journal of Psychiatric and Mental Health Nursing. 2013 Sep;20(7):584-94. | Study not about effectiveness |
| 569 | Ford, S.Funding for nurse-led innovation in the community. NursingTimes.net 2014 | Study not about effectiveness |
| 570 | Forshee JD, Whalen EB, Hackel R, Butt LT, Smeltzer PA, Martin J, Lavin PT, Buchner DA. The effectiveness of one-on-one nurse education on the outcomes of high-risk adult and pediatric patients with asthma. Managed care interface. 1998 Dec 1;11(12):82-92. | Study not about effectiveness |
| 571 | Fortin J, Skrabka K, Avinoam G, Willems J, Sharp S, Linkewich E. Abstract W P276: Developing Excellence in Stroke Care Through Knowledge Building and Interprofessional Collaborative Processes. Stroke. 2014 Feb;45(suppl_1):AWP276-. | Conference abstract |
| 572 | Fortin JE, Skrabka K, Avinoam G, Linkewich E, Willems J, Sharp S. A Cross-System Approach to Building Expert Stroke Teams. InSTROKE 2013 Dec 1 (Vol. 44, No. 12, pp. E223-E224). 530 WALNUT ST, PHILADELPHIA, PA 19106-3621 USA: LIPPINCOTT WILLIAMS & WILKINS. | Conference abstract |
| 573 | Fortney JC, Pyne JM, Ward-Jones S, Bennett IM, Diehl J, Farris K, Cerimele JM, Curran GM. Implementation of evidence-based practices for complex mood disorders in primary care safety net clinics. Families, Systems, & Health. 2018 Sep;36(3):267. | Study not about effectiveness |
| 574 | Foster D, Crisp R, Kamal N. Vancouver Island Health Authority's (VIHA) Strategic Plan to Ensure All Patients Receive Care in Stroke Units. InSTROKE 2013 Dec 1 (Vol. 44, No. 12, pp. E179-E180). 530 WALNUT ST, PHILADELPHIA, PA 19106-3621 USA: LIPPINCOTT WILLIAMS & WILKINS. | Conference abstract |
| 575 | Foster J, Spence K, Henderson‐Smart D, Harrison D, Gray PH, Bidewell J. Procedural pain in neonates in A ustralian hospitals: A survey update of practices. Journal of paediatrics and child health. 2013 Jan;49(1):E35-9. | Study not about effectiveness |
| 576 | Foster M, Sethares K. Current strategies to implement informatics into the nursing curriculum: an integrative review. On-Line Journal of Nursing Informatics. 2017 Nov 1;21(3). | Letters to the editor/ Review studies |
| 577 | Foster, S.Digital can make a difference.British Journal of Nursing 01/09/ 2020;29(1):75-75 | Letters to the editor/ Review studies |
| 578 | Fox* J, Stromberg I, Sterling T, Grossgold E, Walters R. MP15-16 A SURGICAL CLINIC’S JOURNEY TOWARD A CULTURE OF SAFETY. The Journal of Urology. 2019 Apr;201(Supplement 4):e202-. | Conference abstract |
| 579 | Frampton SB, Charmel PA, Guastello S, editors. The putting patients first field guide: Global lessons in designing and implementing patient-centered care. John Wiley & Sons; 2013 Sep 20. | No full text available |
| 580 | Francis K, Lucente K. A Better Way of Determining Hand Hygiene Compliance by Counting BOTH Entering AND Exiting a Patient Room as One Compliant Episode. American Journal of Infection Control. 2011 Jun 1;39(5):E170-1. | Conference abstract |
| 581 | Frank 2018 | Conference abstract |
| 582 | Frank J., Johnston R.,Cadotte B., Shuemake V., Emerald M. Dam rounds-saving lives 1 death at a time. Journal of Hospital Medicine.2018;13(4 Supplement 1). | Study not about effectiveness |
| 583 | Frantsve-Hawley J, Meyer DM. The evidence-based dentistry champions: a grassroots approach to the implementation of EBD. Journal of Evidence Based Dental Practice. 2008 Jun 1;8(2):64-9. | Study not about effectiveness |
| 584 | Frantsve-Hawley J, Newman MG, Meyer DM, Krishna A. Proceedings of the Evidence-Based Dentistry Champion Conference. Introduction. The journal of evidence-based dental practice. 2009 Sep;9(3):105-6. | Duplicate |
| 585 | Frantsve-Hawley J, Newman MG, Meyer DM, Krishna A. Proceedings of the Evidence-Based Dentistry Champion Conference. Introduction. The journal of evidence-based dental practice. 2009 Sep;9(3):105-6. | Study not about effectiveness |
| 586 | Frantsve-Hawley J, Newman MG, Meyer DM. Proceedings of the Evidence-Based Dentistry Champion Conference. The journal of evidence-based dental practice. 2008;3(8):113-4. | Duplicate |
| 587 | Freedman R, Rooney K, Asiimwe A, Kayima P, Mumbere O, Ocokoni T, Okidi R, Schaffer N, Ssemujju A, Tungotyo M. Relaunching the WHO Surgical Safety Checklist in a Ugandan referral hospital. Is sustainable change possible? A Lifebox Fellowship project. InANAESTHESIA 2017 Jan 1 (Vol. 72, pp. 22-22). 111 RIVER ST, HOBOKEN 07030-5774, NJ USA: WILEY. | Conference abstract |
| 588 | Freeman SD, Price Jr DL, Landry R, Free C, Wright M, Ostra M, Anton V, Small CW. Beating Back the Brain Attack: Improving Stroke Patients' Door to Lytic Time. InSTROKE 2011 Mar 1 (Vol. 42, No. 3, pp. E332-E332). 530 WALNUT ST, PHILADELPHIA, PA 19106-3621 USA: LIPPINCOTT WILLIAMS & WILKINS. | Conference abstract |
| 589 | Freeman, G. (2018). "CAPTURE Focuses on Coordination, Gait Support." Hospital Peer Review 43(6). | Letters to the editor/ Review studies |
| 590 | Freeman, G.CAPTURE Focuses on Coordination, Gait Support Healthcare Risk Management 2018;40(8) | Study not about effectiveness |
| 591 | Freer T, Ward S. Integration of Respiratory Services. International Journal of Integrated Care. 2017 Jul 11;17(3). | Conference abstract |
| 592 | Frew PM, Randall LA, Malik F, Limaye RJ, Wilson A, O'Leary ST, Salmon D, Donnelly M, Ault K, Dudley MZ, Fenimore VL. Clinician perspectives on strategies to improve patient maternal immunization acceptability in obstetrics and gynecology practice settings. Human vaccines & immunotherapeutics. 2018 Jul 3;14(7):1548-57. | Study not about effectiveness |
| 593 | Friedman A.J., Brown J.T.A framework for achieving excellence in cancer patient: A consensus building process to adopt/adapt evidence-based guidelines and making them relevant for a Canadian context. Journal of Cancer Education / 2009;24(SUPPL. 1):54-55 | Conference abstract |
| 594 | Friedman KG, Fulton DR. Reducing cost through standardization. Current Treatment Options in Pediatrics. 2016 Dec;2(4):296-310. | Study not about effectiveness |
| 595 | Friese CR, Mendelsohn‐Victor K, Ginex P, McMahon CM, Fauer AJ, McCullagh MC. Lessons learned from a practice‐based, multisite intervention study with nurse participants. Journal of Nursing Scholarship. 2017 Mar;49(2):194-201. | Study not about effectiveness |
| 596 | Fry M, Chenoweth L, Arendts G. Assessment and management of acute pain in the older person with cognitive impairment: a qualitative study. International emergency nursing. 2016 Jan 1;24:54-60. | Not a champion |
| 597 | Fu,​ Steven S,​ Roth,​ Craig,​ Battaglia,​ Catherine T,​ Nelson,​ David B,​ Farmer,​ Melissa M,​ Do,​ Tam,​ Goldstein,​ Michael G,​ Widome,​ Rachel,​ Hagedorn,​ Hildi,​ Zillich,​ Alan J (2015). Training primary care clinicians in motivational interviewing: a comparison of two models. *#journal#*,​ 98(1),​ 61 | Study not about effectiveness |
| 598 | Fudge H, MacIntosh R, Burgess S, Holland J, Ayer L, Foster J. 1372: IMPROVING PATIENT FLOW FROM PICU TO INPATIENT WARDS: A QUALITY ASSURANCE PROCESS. Critical Care Medicine. 2019 Jan 1;47(1):662. | Conference abstract |
| 599 | Fuentes L, Dennis A, Douglas-Durham E, Grossman D. Exploring best practices for transitioning to outpatient miscarriage management. Contraception. 2014 Sep 1;90(3):305. | Conference abstract |
| 600 | Fulda KG, Hahn KA, Young RA, Marshall JD, Moore BJ, Espinoza AM, Beltran NM, McFadden P, Crim AD, Cardarelli R. Recruiting Practice-based Research Network (PBRN) physicians to be research participants: lessons learned from the North Texas (NorTex) needs assessment study. The Journal of the American Board of Family Medicine. 2011 Sep 1;24(5):610-5. | Study not about effectiveness |
| 601 | Furnell C, Finlay F. G575 (P) Promoting quality in a community paediatric department. | Conference abstract |
| 602 | Gagnon MP, Desmartis M, Labrecque M, L_gar_ F, Lamothe L, Fortin JP, Rancourt JF, Duplantie J. Implementation of an electronic medical record in family practice: a case study. Journal of Innovation in Health Informatics. 2010;18(1):31-40. | Study not about effectiveness |
| 603 | Gaines-Hill, S., Ohanian, S. Reduction of CAUTI rates organization wide begins in the Emergency Department...46th Annual Conference, APIC 2019, Philadelphia, PA. American Journal of Infection Control Supplement 2019;47():S37-S37 | Conference abstract |
| 604 | Gallagher K, Nutting PA, Nease DE, Graham DG, Bonham AJ, Dickinson WP, Main DS. It takes two: using coleaders to champion improvements in small primary care practices. The Journal of the American Board of Family Medicine. 2010 Sep 1;23(5):632-9. | Study not about effectiveness |
| 605 | Gallaher C, Herrmann S, Hunter L, Wilkins A. The St Thomas’ Hospital Emergency Department Homeless Health Initiative: improving the quality. BMJ open quality. 2020 Feb 1;9(1):e000820. | Study not about effectiveness |
| 606 | Garcia M.L.B., Simbulan C., Patel A., El Hassan M.A., Thomas M.S., El Magboul E.I. A journey towards improving physicians hand hygiene compliance. Antimicrobial Resistance and Infection Control / 2019;8(Supplement 1) | Conference abstract |
| 607 | García PJ, Cárcamo CP, Chiappe M, Valderrama M, La Rosa S, Holmes KK, Mabey DC, Peeling RW. Rapid syphilis tests as catalysts for health systems strengthening: a case study from Peru. PloS one. 2013 Jun 26;8(6):e66905. | Study not about effectiveness |
| 608 | Gardetto NJ, Carroll KC. Management strategies to meet the core heart failure measures for acute decompensated heart failure: a nursing perspective. Critical care nursing quarterly. 2007 Oct 1;30(4):307-20. | Study not about effectiveness |
| 609 | Garfield MJ, Watson RT. Four case studies in state-supported telemedicine initiatives. Telemedicine Journal and e-Health. 2003 Jun 1;9(2):197-205. | Study not about effectiveness |
| 610 | Garner D., Triggs N., Himes R. Educational curriculum for primary care providers regarding focused weight management programs. Journal of Pediatric Gastroenterology and Nutrition. 2019;69(Supplement 2) | Conference abstract |
| 611 | Gartrell K, Trinkoff AM, Storr CL, Wilson ML. Electronic Personal Health Record Use Among Nurses in the Nursing Informatics Community. CIN: Computers, Informatics, Nursing. 2015 Jul 1;33(7):306-14. | Study not about effectiveness |
| 612 | Garza J., Raymond T.,Mannan T,, Drescher K., Wurtz E. Saving patient lives in the CICU: Team challenges the status quo. World Journal for Pediatric and Congenital Heart Surgery / 2019;10(2):NP32 | Conference abstract |
| 613 | Gask L, Lever-Green G, Hays R. Dissemination and implementation of suicide prevention training in one Scottish region. BMC Health Services Research. 2008 Dec;8(1):1-3. | Study not about effectiveness |
| 614 | Gavriloff C. A performance improvement plan to increase nurse adherence to use of medication safety software. Journal of pediatric nursing. 2012 Aug 1;27(4):375-82. | Study not about effectiveness |
| 615 | Geer JJ. Increasing Vaccination Rates in a Pediatric Chronic Hemodialysis Unit. Nephrology Nursing Journal. 2016 Jan 1;43(1). | Study not about effectiveness |
| 616 | Gehani M, Balasubramaniam SM, Johnson AR, Goklani G. Maternal and Perinatal Complications from a Hospital-based Intra-partum Complications Registry Established in Remote Referral Hospitals of a Desert District of India. Indian Journal of Community Health. 2019 Jul 1;31(3). | Study not about effectiveness |
| 617 | Gelmon S, Bouranis N, Sandberg B, Petchel S. Strategies for addressing the challenges of patient-centered medical home implementation: Lessons from Oregon. The Journal of the American Board of Family Medicine. 2018 May 1;31(3):334-41. | Study not about effectiveness |
| 618 | Gerard, J. (2019). Introducing learning disability champions in an acute hospital. Nursing Times. 2019;115(4):44-47 | Study not about effectiveness |
| 619 | Gerrish K, Laker S, Taylor C, Kennedy F, McDonnell A. Enhancing the quality of oral nutrition support for hospitalized patients: a mixed methods knowledge translation study (The EQONS study). Journal of advanced nursing. 2016 Dec;72(12):3182-94. | Study not about effectiveness |
| 620 | Gesthalter YB, Koppelman E, Bolton R, Slatore CG, Yoon SH, Cain HC, Tanner NT, Au DH, Clark JA, Wiener RS. Evaluations of implementation at early-adopting lung cancer screening programs: lessons learned. Chest. 2017 Jul 1;152(1):70-80. | Study not about effectiveness |
| 621 | Ghonim ER, Ime M. Reducing Catheter-Associated Urinary Tract Infection in a Major Academic Facility Using a Novel Comprehensive Tool. American Journal of Infection Control. 2020 Aug 1;48(8):S47-8. | Conference abstract |
| 622 | Gifford H, Paton S, Cvitanovic L, McMenamin J, Newton C. Is routine alcohol screening and brief intervention feasible in a New Zealand primary care environment. NZ Med J. 2012 May 11;125(1354):17-25. | Study not about effectiveness |
| 623 | Gilchrist AT, Gabi Avni RN, Gay SE. e-health at The Ottawa Hospital. The Canadian Nurse. 2008 May 1;104(5):34. | Letters to the editor/ Review studies |
| 624 | Gilleland Marchak J, Halpin SN, Escoffery C, Owolabi S, Mertens AC, Wasilewski‐Masker K. Using formative evaluation to plan for electronic psychosocial screening in pediatric oncology. Psycho‐Oncology. 2021 Feb;30(2):202-11. | Study not about effectiveness |
| 625 | Gillespie BM, Hamilton K, Ball D, Lavin J, Gardiner T, Withers TK, Marshall AP. Unlocking the “black box” of practice improvement strategies to implement surgical safety checklists: a process evaluation. Journal of multidisciplinary healthcare. 2017;10:157. | Study not about effectiveness |
| 626 | Gillespie BM, Harbeck E, Lavin J, Gardiner T, Withers TK, Marshall AP. Using normalisation process theory to evaluate the implementation of a complex intervention to embed the surgical safety checklist. BMC health services research. 2018 Dec;18(1):1-1. | Study not about effectiveness |
| 627 | Ginestra J, Klaiman T, Oredeko F, Silvestri J, Srinivasan T, Szymanski S, Tran T, Lane-Fall M, Kerlin MP. Determinants of utilization of prone positioning for severe acute respiratory distress syndrome. InC24. ECMO, PRONE POSITIONING, AND NMB IN ARDS 2020 May (pp. A4598-A4598). American Thoracic Society. | Conference abstract |
| 628 | Girgis A, Delaney G, Arnold A, Miller AA, Carolan M, Della-Fiorentina S, Kaadan N, Avery S, Domburg N, Ng W, Spring K. Translating evidence into practice through the PROMPT-care project: utilising patient reported outcome measures for personalised cancer treatment and care. | Conference abstract |
| 629 | Girvalaki C, Saridaki A, Papadakis S, Vardavas C, Lionis C. Study protocol of the TiTAN Crete project: Development of a tobacco treatment training network in Crete, Greece. | Conference abstract |
| 630 | Givens SE, Skully C, Bromley G. Psychiatric inpatient bedside handoff: implementation of a quality improvement project and nurses' responses. Journal of psychosocial nursing and mental health services. 2016 Jul 1;54(7):33-7. | Study not about effectiveness |
| 631 | Godbee K, Gunn J, Lautenschlager NT, Curran E, Palmer VJ. Implementing dementia risk reduction in primary care: a preliminary conceptual model based on a scoping review of practitioners’ views. Primary health care research & development. 2019;20. | Letters to the editor/ Review studies |
| 632 | Godbee K, Gunn J, Lautenschlager NT, Palmer VJ. Refined conceptual model for implementing dementia risk reduction: incorporating perspectives from Australian general practice. Australian Journal of Primary Health. 2020 Jul 7;26(3):247-55. | Study not about effectiveness |
| 633 | Goedken CC, Livorsi DJ, Sauder M, Vander Weg MW, Chasco EE, Chang NC, Perencevich E, Reisinger HS. “The role as a champion is to not only monitor but to speak out and to educate”: the contradictory roles of hand hygiene champions. Implementation Science. 2019 Dec;14(1):1-1. | Study not about effectiveness |
| 634 | Goett R, Lamba S, Wang D, Aberger K, Schultz R. Joining forces with the emergency department: Successful programs, initiatives, and emerging practices (TH318). Journal of Pain and Symptom Management. 2018 Feb 1;55(2):568. | Conference abstract |
| 635 | Goff SL, Unruh ML, Klingensmith J, Eneanya ND, Garvey C, Germain MJ, Cohen LM. Advance care planning with patients on hemodialysis: an implementation study. BMC palliative care. 2019 Dec;18(1):1-8. | Study not about effectiveness |
| 636 | Goicolea I, Vives-Cases C, Hurtig AK, Marchal B, Briones-Vozmediano E, Otero-García L, García-Quinto M, San Sebastian M. Mechanisms that trigger a good health-care response to intimate partner violence in Spain. Combining realist evaluation and qualitative comparative analysis approaches. PLoS one. 2015 Aug 13;10(8):e0135167. | Study not about effectiveness |
| 637 | Gold HT, Karia RJ, Link A, Lebwohl R, Zuckerman JD, Errico TJ, Slover JD, Buckland AJ, Mann DM, Cantor MN. Implementation and early adaptation of patient-reported outcome measures into an electronic health record: a technical report. Health informatics journal. 2020 Mar;26(1):129-40. | Study not about effectiveness |
| 638 | Gold M, Taylor EF. Moving research into practice: lessons from the US Agency for Healthcare Research and Quality's IDSRN program. Implementation Science. 2007 Dec;2(1):1-1. | Study not about effectiveness |
| 639 | Gold R., Bunce A.,Cohen D., Hollombe C., Nelson C., DeVoe J. Seeing the invisible: An example of how "support mechanisms" affect implementation. Clinical and Translational Science / 2014;7(3):270-271 | Conference abstract |
| 640 | Goldberg JP, Wright CM. Lessons learned from two decades of research in nutrition education and obesity prevention: considerations for alcohol education. Patient education and counseling. 2017 Jan 1;100:S30-6. | Not within a health care setting |
| 641 | Goldlist K, Rhodes-Kropf J. Integrating Memory Support into Primary Care (IMSIP). InJOURNAL OF THE AMERICAN GERIATRICS SOCIETY 2019 Apr 1 (Vol. 67, pp. S42-S42). 111 RIVER ST, HOBOKEN 07030-5774, NJ USA: WILEY. | Conference abstract |
| 642 | Goldman J, Meuser J, Lawrie L, Rogers J, Reeves S. Interprofessional primary care protocols: a strategy to promote an evidence-based approach to teamwork and the delivery of care. Journal of interprofessional care. 2010 Nov 1;24(6):653-65. | Study not about effectiveness |
| 643 | Goldsack J, Bergey M, Mascioli S, Cunningham J. Hourly rounding and patient falls: what factors boost success?. Nursing2020. 2015 Feb 1;45(2):25-30. | Study not about effectiveness |
| 644 | Goldszer RC, Rutherford A, Banks P, Zou KH, Curley M, Rossi PB, Kahlert T, Goulart D, Santos K, Gustafson M. Implementing clinical pathways for patients admitted to a medical service: lessons learned. Critical pathways in cardiology. 2004 Mar 1;3(1):35-41 | Study not about effectiveness |
| 645 | Gonzales R, Auerbach A. Trainees, teams, and timely performance feedback. | Study not about effectiveness |
| 646 | Gonzales R., Anderer T., Stahl M., Yefko M., Maselli J., Molecavage J., Bloom F.,Metlay J. Comparative effectiveness of different point-of-care strategies to improve antibiotic use for acute bronchitis in primary care. Journal of General Internal Medicine / 2011;26(10):1223 | Conference abstract |
| 647 | Goodfriend L, Kennedy S, Hein A, Baker R. Implementation of a Vascular Access Experience Program to Train Unit-Based Vascular Access Champions. Journal of Infusion Nursing. 2020 Jul 1;43(4):193-9. | Study not about effectiveness |
| 648 | Goodhand K. Transfer of learning from simulation to clinical practice in pre-registration healthcare student education (Doctoral dissertation). | Study not about effectiveness |
| 649 | Goodson P, Smith MM, Evans A, Meyer B, Gottlieb NH. Maintaining prevention in practice: survival of PPIP in primary care settings. American journal of preventive medicine. 2001 Apr 1;20(3):184-9. | Study not about effectiveness |
| 650 | Goodyear-Smith F, Petousis-Harris H, Turner N. Immunization champions: characteristics of general practitioners associated with better immunization delivery. Human vaccines. 2009 Jun 1;5(6):403-11. | Not a champion |
| 651 | Gordon PR. The effects of nursing education on decreasing catheter associated urinary tract infection rates (Doctoral dissertation, Walden University). | Study not about effectiveness |
| 652 | Gören JL, Rose AJ, Engle RL, Smith EG, Christopher ML, Rickles NM, Semla TP, McCullough MB. Organizational characteristics of Veterans Affairs clinics with high and low utilization of clozapine. Psychiatric Services. 2016 Nov 1;67(11):1189-96. | Study not about effectiveness |
| 653 | Gosling AS, Westbrook JI, Coiera EW. Variation in the use of online clinical evidence: a qualitative analysis. International journal of medical informatics. 2003 Jan 1;69(1):1-6. | Study not about effectiveness |
| 654 | Gotlib Conn L, McKenzie M, Pearsall EA, McLeod RS. Successful implementation of an enhanced recovery after surgery programme for elective colorectal surgery: a process evaluation of champions’ experiences. | Study not about effectiveness |
| 655 | Gottlieb LD, Roer D, Jega K, D'arc St Pierre J, Dobbins J, Dwyer M, Lewis S, Manus D. Clinical pathway for pneumonia: development, implementation, and initial experience. Best practices and benchmarking in healthcare: a practical journal for clinical and management application. 1996 Sep 1;1(5):262-5. | Study not about effectiveness |
| 656 | Gottlieb O. Anesthesia information management systems in the ambulatory setting: benefits and challenges. Anesthesiology clinics. 2014 Jun 1;32(2):559-76. | Study not about effectiveness |
| 657 | Gotur D, Juang PJ, Faz L, Richardson L, Perry L, Gloria K, Riley T, Baldwin S, Fontenot N, Young J, Zimmerman J. 380: A STEPWISE APPROACH ON QUALITY INITIATIVE FOR MANUAL PRONING IN A MEDICAL ICU. Critical Care Medicine. 2018 Jan 1;46(1):173. | Conference abstract |
| 658 | Gould KA. A Culture of Respect: Champions and Models. | Study not about effectiveness |
| 659 | Goutier JM, Holzmueller CG, Edwards KC, Klompas M, Speck K, Berenholtz SM. Strategies to enhance adoption of ventilator-associated pneumonia prevention interventions: a systematic literature review. Infection Control & Hospital Epidemiology. 2014 Aug;35(8):998-1005. | Letters to the editor/ Review studies |
| 660 | Govindaswamy P., Laing S., Waters D., Walker K., Spence K., Badawi N. Translating research in to practice in a surgical nicu. Journal of Paediatrics and Child Health. 2019;55(Supplement 1):78 | Conference abstract |
| 661 | Gradin S, Wolf N, Yurcan M, Yardley T, Kaizer L, Kukreti V. Evaluating the implementation of computerized prescriber order entry (CPOE) for systemic treatment (ST) in Ontario. | Conference abstract |
| 662 | Graham E, Campbell S. Reducing avoidable emergency department attendances through bespoke education. Nursing Older People (2014+). 2017 Nov 1;29(10):32. | Study not about effectiveness |
| 663 | Graham ID, Alvarez G, Tetroe J, McAuley L, Laupacis A. Factors influencing the adoption of blood alternatives to minimize allogeneic transfusion: the perspective of eight Ontario hospitals. Canadian journal of surgery. 2002 Apr;45(2):132. | Study not about effectiveness |
| 664 | Graham JM, Sabeta ME, Cooke JT, Berg ER, Osten WM. A system's approach to improve organ donation. Progress in Transplantation. 2009 Sep;19(3):216-20. | Study not about effectiveness |
| 665 | Gramlich L, Nelson G, Nelson A, Lagendyk L, Gilmour LE, Wasylak T. Moving enhanced recovery after surgery from implementation to sustainability across a health system: a qualitative assessment of leadership perspectives. BMC health services research. 2020 Dec;20(1):1-1. | Study not about effectiveness |
| 666 | Gramlich LM, Nelson G, Nelson A, Wasylak T. Sustaining enhanced recovery across a health system: leadership perspectives. Clinical Nutrition ESPEN. 2019 Jun 1;31:125-6. | Conference abstract |
| 667 | Grande SW, Durand MA, Fisher ES, Elwyn G. Physicians as part of the solution? Community-based participatory research as a way to get shared decision making into practice. Journal of general internal medicine. 2014 Jan 1;29(1):219-22. | Not a champion |
| 668 | Granger CL, Denehy L, Remedios L, Retica S, Phongpagdi P, Hart N, Parry SM. Barriers to translation of physical activity into the lung cancer model of care. A qualitative study of clinicians’ perspectives. Annals of the American Thoracic Society. 2016 Dec;13(12):2215-22. | Study not about effectiveness |
| 669 | Gray D. The development of gloucestershirés clinical standards for core rehabilitation-an integrated system approach. Physiotherapy. 2020 May 1;107:e131-2. | Conference abstract |
| 670 | Gray J, Razmus I. Improving venous thromboembolism prevention processes and outcomes at a community hospital. The Joint Commission Journal on Quality and Patient Safety. 2012 Feb 1;38(2):61-AP5. | Study not about effectiveness |
| 671 | Gray, D., Struve, S., Huibregtse, C., Chen, Q.New primary palliative care (PC) model for community cancer clinics (CCC). Journal of Clinical Oncology 10/10/ 2016;34(29):159-159 | Conference abstract |
| 672 | Gray-Miceli D, Quigley PA. Fall prevention: assessment, diagnoses, and intervention strategies. Evidence-based geriatric nursing protocols for best practice. 2012;4. | Study not about effectiveness |
| 673 | Greco S, Jackson K, Kritek P, Nasenbeny K, Shushan S, Ross B. 270: ROLE-BASED RESUSCITATION AND INTERDISCIPLINARY CODE BLUE TEAM TRAINING. Critical Care Medicine. 2014 Dec 1;42(12):A1425. | Conference abstract |
| 674 | Green CA, McCarty D, Mertens J, Lynch FL, Hilde A, Firemark A, Weisner CM, Pating D, Anderson BM. A qualitative study of the adoption of buprenorphine for opioid addiction treatment. Journal of substance abuse treatment. 2014 Mar 1;46(3):390-401. | Study not about effectiveness |
| 675 | Green E, de Calvo LE, Truant TL, McCarthy S, MacDonald D, Patel K, Stewart J, Brunelli MV, Cullen C, de Ellis SE, Nunes LM. Oral Chemotherapy: Enhancing Nursing Practice in Four Latin American Countries. InCANCER NURSING 2016 Nov 1 (Vol. 39, pp. S54-S54). TWO COMMERCE SQ, 2001 MARKET ST, PHILADELPHIA, PA 19103 USA: LIPPINCOTT WILLIAMS & WILKINS. | Conference abstract |
| 676 | Greene D, Reifert D, Elleson J, Levitt J. A Journey Toward Blood Management at an Academic Medical Center: Searching for Excellence: AP29. Transfusion. 2011 Sep;51. | Conference abstract |
| 677 | Greene MT, Fakih MG, Watson SR, Ratz D, Saint S. Reducing inappropriate urinary catheter use in the emergency department: comparing two collaborative structures. infection control & hospital epidemiology. 2018 Jan;39(1):77-84. | Study not about effectiveness |
| 678 | Greenhalgh T, Stramer K, Bratan T, Byrne E, Mohammad Y, Russell J. Introduction of shared electronic records: multi-site case study using diffusion of innovation theory. Bmj. 2008 Oct 23;337. | Study not about effectiveness |
| 679 | Greenwood N, Mackenzie A, Habibi R, Atkins C, Jones R. General practitioners and carers: a questionnaire survey of attitudes, awareness of issues, barriers and enablers to provision of services. BMC Family Practice. 2010 Dec;11(1):1-8. | Study not about effectiveness |
| 680 | Gregory A, Ramsay J, Agnew-Davies R, Baird K, Devine A, Dunne D, Eldridge S, Howell A, Johnson M, Rutterford C, Sharp D. Primary care I dentification and R eferral to I mprove S afety of women experiencing domestic violence (IRIS): protocol for a pragmatic cluster randomised controlled trial. BMC public health. 2010 Dec;10(1):1-7. | Study not about effectiveness |
| 681 | Greiver M, Barnsley J, Glazier RH, Moineddin R, Harvey BJ. Implementation of electronic medical records: Theory-informed qualitative study. Canadian Family Physician. 2011 Oct 1;57(10):e390-7. | Study not about effectiveness |
| 682 | Grembowski D, Marcus-Smith M. The 10 conditions that increased Vermont's readiness to implement statewide health system transformation. Population health management. 2018 Jun 1;21(3):180-7. | Study not about effectiveness |
| 683 | Gribko M. Community Education through a Stroke Champion Program (Doctoral dissertation, Walden University). | Study not about effectiveness |
| 684 | Griffin B, Cooper H, Horack C, Klyber M, Schimmelpfenning D. Best-practice protocols: reducing harm from pressure ulcers. Nursing management. 2007 Sep 1;38(9):29-32. | Study not about effectiveness |
| 685 | Griffiths, J.NEW SCHEME TO CREATE RESEARCH CHAMPIONS. Midwives 09// 2018;21():10-11 | Letters to the editor/ Review studies |
| 686 | Grudzen CR, Stone SC, Morrison RS. The palliative care model for emergency department patients with advanced illness. Journal of palliative medicine. 2011 Aug 1;14(8):945-50. | Study not about effectiveness |
| 687 | Guetterman TC, Kellenberg JE, Krein SL, Harrod M, Lehrich JL, Iwashyna TJ, Kronick SL, Girotra S, Chan PS, Nallamothu BK. Nursing roles for in-hospital cardiac arrest response: higher versus lower performing hospitals. BMJ quality & safety. 2019 Nov 1;28(11):916-24. | Study not about effectiveness |
| 688 | Gui X, Chen Y, Zhou X, Reynolds TL, Zheng K, Hanauer DA. Physician champions’ perspectives and practices on electronic health records implementation: challenges and strategies. JAMIA open. 2020 Apr;3(1):53-61. | Study not about effectiveness |
| 689 | Guihan M, Bosshart HT, Nelson A. Lessons learned in implementing SCI clinical practice guidelines. SCI nursing: a publication of the American Association of Spinal Cord Injury Nurses. 2004 Jan 1;21(3):136-42. | No full text available |
| 690 | Gunn R, Ferrara LK, Dickinson C, Stock I, Griffith-Weprin J, Wiser A, Hatch B, Fagnan LJ, Carney PA, Davis MM. Human Papillomavirus Immunization in Rural Primary Care. American journal of preventive medicine. 2020 Sep 1;59(3):377-85. | Study not about effectiveness |
| 691 | Gupta A. Antimicrobial stewardship: think global, act local. International Journal of Infectious Diseases. 2014 Apr 1;21:74-5. | Conference abstract |
| 692 | Gupta R, Berman J, Dervan A, Abhat A, Marcotte L. COMMON DOLLARS, COMMON SENSE: HARMONIZING A HIGH VALUE CARE CURRICULUM ACROSS AMBULATORY AND INPATIENT SETTINGS IN AN ACADEMIC MEDICAL SYSTEM. InJOURNAL OF GENERAL INTERNAL MEDICINE 2014 Apr 1 (Vol. 29, pp. S511-S511). 233 SPRING ST, NEW YORK, NY 10013 USA: SPRINGER. | Conference abstract |
| 693 | Gurzick M, Kesten KS. The impact of clinical nurse specialists on clinical pathways in the application of evidence-based practice. Journal of Professional Nursing. 2010 Jan 1;26(1):42-8. | Study not about effectiveness |
| 694 | Gutierrez F, Smith K. Reducing falls in a definitive observation unit: an evidence-based practice institute consortium project. Critical care nursing quarterly. 2008 Apr 1;31(2):127-39. | Study not about effectiveness |
| 695 | Gyedu A, Bingener J, Dally C, Oppong J, Price R, Reid-Lombardo K. Starting a laparoscopic surgery programme in the second largest teaching hospital in Ghana. East African medical journal. 2014 Nov 20;91(4):133-7. | Study not about effectiveness |
| 696 | Hadjistavropoulos T, Williams J, Kaasalainen S, Hunter PV, Savoie ML, Wickson-Griffiths A. Increasing the frequency and timeliness of pain assessment and management in long-term care: knowledge transfer and sustained implementation. Pain Research and management. 2016 Jan 1;2016. | Study not about effectiveness |
| 697 | Hagedorn HJ, Brown R, Dawes M, Dieperink E, Myrick DH, Oliva EM, Wagner TH, Wisdom JP, Harris AH. Enhancing access to alcohol use disorder pharmacotherapy and treatment in primary care settings: ADaPT-PC. Implementation Science. 2015 Dec;11(1):1-9. | Study not about effectiveness |
| 698 | Hagedorn HJ, Wisdom JP, Gerould H, Pinsker E, Brown R, Dawes M, Dieperink E, Myrick DH, Oliva EM, Wagner TH, Harris AH. Implementing alcohol use disorder pharmacotherapy in primary care settings: a qualitative analysis of provider-identified barriers and impact on implementation outcomes. Addiction science & clinical practice. 2019 Dec;14(1):1-3. | Study not about effectiveness |
| 699 | Hagg R. Multidisciplinary journey to reduce catheter related urinary tract infections. American Journal of Infection Control. 2009;37(5):49-50. | Conference abstract |
| 700 | Hai NT, Tapanainen T, Ishmatova D. Critical success factors in health information technology implementation: The perspective of Finnish IT managers. InE-Health and Telemedicine: Concepts, Methodologies, Tools, and Applications 2016 (pp. 1488-1505). IGI Global. | Study not about effectiveness |
| 701 | Hai NT, Tapanainen T, Ishmatova D. Critical success factors in health information technology implementation: The perspective of Finnish IT managers. InE-Health and Telemedicine: Concepts, Methodologies, Tools, and Applications 2016 (pp. 1488-1505). IGI Global. | Study not about effectiveness |
| 702 | Haizlip J, Plews-Ogan M. Successful Adaptation of Appreciative Inquiry for Academic Medicine. AI Practitioner. 2010 Aug 1;12(3). | Study not about effectiveness |
| 703 | Hale M, Carberry G, Watters J, Sherry S, Murray K. Ventilator-Associated Pneumonia in the Trauma Intensive Care Unit: Getting to Zero and Staying There. American Journal of Infection Control. 2011 Jun 1;39(5):E59-60. | Conference abstract |
| 704 | Hale R, Powell T, Drey NS, Gould DJ. Working practices and success of infection prevention and control teams: a scoping study. Journal of Hospital Infection. 2015 Feb 1;89(2):77-81. | Letters to the editor/ Review studies |
| 705 | Hale-Gallardo JL, Kreider CM, Jia H, Castaneda G, Freytes IM, Ripley DC, Ahonle ZJ, Findley K, Romero S. Telerehabilitation for rural veterans: A qualitative assessment of barriers and facilitators to implementation. Journal of Multidisciplinary Healthcare. 2020;13:559. | Study not about effectiveness |
| 706 | Hall KM, Moreno JR, Dosselman L, Bagley CA. Implementation of an Enhanced Recovery Pathway in Complex Spine Surgery Patients in a Multidisciplinary Center. Neurosurgery. 2019 Sep 1;66(Supplement_1):nyz310_614. | Conference abstract |
| 707 | Hall, C; Engle, C; Flahaven, E. WHAT DO PUPPs AND SKIN HAVE IN COMMON? A MULTIDISCIPLINARY APPROACH TO DOCUMENTATION IMPROVEMENTS...WOCN Society’s 49th Annual Conference, Salt Lake City, Utah, May 19-23, 2017. Journal of Wound, Ostomy & Continence Nursing ;44():S42-S43 | Conference abstract |
| 708 | Ham C. Improving the performance of health services: the role of clinical leadership. The Lancet. 2003 Jun 7;361(9373):1978-80. | Study not about effectiveness |
| 709 | Hanson DS. Nurse educators' consensus opinion on using an academic electronic health record: A Delphi study. The University of North Dakota; 2013. | Study not about effectiveness |
| 710 | Hanson J, Grant M. UNIT-SPECIFIC EVIDENCE-BASED PRACTICE NURSING CHAMPIONS.: 2945. InOncology Nursing Forum 2008 (Vol. 35, No. 3). | Conference abstract |
| 711 | Hardy S, Mushore M, Goddard L. Supporting student mental health nurses in clinical placement through virtual in-practice support (VIPS): Innovation uptake and the ‘VIPS’project. Nurse education today. 2016 Nov 1;46:133-8. | Study not about effectiveness |
| 712 | Hargraves D, White C, Frederick R, Cinibulk M, Peters M, Young A, Elder N. Implementing SBIRT (Screening, Brief Intervention and Referral to Treatment) in primary care: lessons learned from a multi-practice evaluation portfolio. Public health reviews. 2017 Dec;38(1):1-1. | Study not about effectiveness |
| 713 | Hargreaves L. Toolkit for Implementation of Temporal Artery Thermometers for Neonates (Doctoral dissertation, Walden University). | Study not about effectiveness |
| 714 | Harnagea H, Lamothe L, Couturier Y, Esfandiari S, Voyer R, Charbonneau A, Emami E. From theoretical concepts to policies and applied programmes: the landscape of integration of oral health in primary care. BMC oral health. 2018 Dec;18(1):1-2. | Letters to the editor/ Review studies |
| 715 | Harper KD, Loper AC, Louison LM, Morse JE. Stage-based implementation of immediate postpartum long-acting reversible contraception using a reproductive justice framework. American journal of obstetrics and gynecology. 2020 Apr 1;222(4):S893-905. | Study not about effectiveness |
| 716 | Harper PG, Baker NJ, Reif CJ. Implementing community-oriented primary care projects in an urban family practice residency program. Fam Med. 2000 Nov 1;32(10):683-90. | Study not about effectiveness |
| 717 | Harris AH, Brown R, Dawes M, Dieperink E, Myrick DH, Gerould H, Wagner TH, Wisdom JP, Hagedorn HJ. Effects of a multifaceted implementation intervention to increase utilization of pharmacological treatments for alcohol use disorders in the US Veterans Health Administration. Journal of substance abuse treatment. 2017 Nov 1;82:107-12. | Study not about effectiveness |
| 718 | Harris D, Karogiannis D, Balfour N, Gerry M, Mushta J, Cabral J. MP26: An emergency department team-based quality improvement initiative reduces narcotic and benzodiazepine ‘to-go’medication administration. Canadian Journal of Emergency Medicine. 2018 May;20(S1):S50-. | Conference abstract |
| 719 | Harrison MB, Mackey M, Friedberg E. Pressure ulcer monitoring: a process of evidence-based practice, quality, and research. The Joint Commission Journal on Quality and Patient Safety. 2008 Jun 1;34(6):355-9. | Study not about effectiveness |
| 720 | Hart T. Promoting hand hygiene in clinical practice. Nursing times. 2013 Sep 1;109(38):14-5. | Study not about effectiveness |
| 721 | Hauer KE, Landefeld CS. CHAMP trains champions: Hospitalist‐educators develop new ways to teach care for older patients. | Study not about effectiveness |
| 722 | Haugen,​ Maureen,​ Kelly,​ Katherine Patterson,​ Leonard,​ Marcia,​ Mills,​ Denise,​ Sung,​ Lillian,​ Mowbray,​ Catriona,​ Landier,​ Wendy (2016). Nurse-Led Programs to Facilitate Enrollment to Children’s Oncology Group Cancer Control Trials. *#journal#*,​ 33(5),​ 387 | Study not about effectiveness |
| 723 | Haun N, Hofer A, Greene MT, Borlaug G, Pritchett J, Scallon T, Safdar N. Prevention of Clostridium difficile infection in rural hospitals. American journal of infection control. 2014 Mar 1;42(3):311-5. | Study not about effectiveness |
| 724 | Hawk M, Nowalk MP, Moehling KK, Pavlik V, Raviotta JM, Brown AE, Zimmerman RK, Ricci EM. Using a mixed methods approach to examine practice characteristics associated with implementation of an adult immunization intervention using the 4 Pillars™ Practice Transformation Program. Journal for healthcare quality: official publication of the National Association for Healthcare Quality. 2017 May;39(3):153. | Study not about effectiveness |
| 725 | Hawkins CM, Alsip CN, Pryor RM, Leach AD, Larson DB. Quality Improvement and Confirmation Projects: Facilitating Rapid, Measurable Performance Improvement. Radiographics: a review publication of the Radiological Society of North America, Inc. 2013 Aug 26:135058-. | Study not about effectiveness |
| 726 | Hayden H. Hand hygiene: Cleaning up bad practice: G552 (P). Archives of Disease in Childhood. 2016 Apr;101. | Conference abstract |
| 727 | Haynes LF, Korte JE, Holmes BE, Gooden L, Matheson T, Feaster DJ, Leff JA, Wilson L, Metsch LR, Schackman BR. HIV rapid testing in substance abuse treatment: Implementation following a clinical trial. Evaluation and Program Planning. 2011 Nov 1;34(4):399-406. | Study not about effectiveness |
| 728 | Haywood K, Humphrey L, Mikles S, Chan EK, Edwards T. A practical supplement to the user's guide to implementing PRO measures in clinical practice: case study analysis of challenges and opportunities. InQUALITY OF LIFE RESEARCH 2016 Oct 1 (Vol. 25, pp. 41-42). VAN GODEWIJCKSTRAAT 30, 3311 GZ DORDRECHT, NETHERLANDS: SPRINGER. | Conference abstract |
| 729 | Health Forum, Inc.Should your best RNs be at the bedside or leading quality effort? H&HN Hospitals & Health Networks.2008; 82 (4). | Study not about effectiveness |
| 730 | Heavens M, Grotemeyer J, Groves C, Fakih M. Preventing Patient Harm by Reducing Unnecessary Urinary Catheter Use: Successful Implementation at 6 Emergency Departments. American Journal of Infection Control. 2014 Jun 1;42(6):S121. | Conference abstract |
| 731 | Heimall LM, Storey B, Stellar JJ, Davis KF. Beginning at the bottom: evidence-based care of diaper dermatitis. MCN: The American Journal of Maternal/Child Nursing. 2012 Jan 1;37(1):10-6. | Study not about effectiveness |
| 732 | Heishman C, Nolting P. Selection, Maintenance, and Remove Today (SMART): Focus on Device Utilization and Healthcare-associated Infections. American Journal of Infection Control. 2014 Jun 1;42(6):S73-4. | Conference abstract |
| 733 | Held RF, Santos S, Marki M, Helmer D. Dissemination and implementation of an educational tool for veterans on complementary and alternative medicine: a case study. BMC complementary and alternative medicine. 2016 Dec;16(1):1-8. | Study not about effectiveness |
| 734 | Helfrich CD, Li YF, Sharp ND, Sales AE. Organizational readiness to change assessment (ORCA): development of an instrument based on the Promoting Action on Research in Health Services (PARIHS) framework. Implementation science. 2009 Dec;4(1):1-3. | Study not about effectiveness |
| 735 | Helfrich CD, Weiner BJ, McKinney MM, Minasian L. Determinants of implementation effectiveness: adapting a framework for complex innovations. Medical care research and review. 2007 Jun;64(3):279-303. | Study not about effectiveness |
| 736 | Helfrich CD. Exploring a model of innovation implementation: Cancer prevention and control trials in community clinical oncology program research bases. The University of North Carolina at Chapel Hill; 2005. | Study not about effectiveness |
| 737 | Helmer-Smith M, Fung C, Afkham A, Crowe L, Gazarin M, Keely E, Moroz I, Liddy C. The feasibility of using electronic consultation in long-term care homes. Journal of the American Medical Directors Association. 2020 Aug 1;21(8):1166-70. | Study not about effectiveness |
| 738 | Helps C, Barclay L. Aboriginal women in rural Australia; a small study of infant feeding behaviour. Women and Birth. 2015 Jun 1;28(2):129-36. | Study not about effectiveness |
| 739 | Helseth SA, Janssen T, Scott K, Squires DD, Becker SJ. Training community-based treatment providers to implement contingency management for opioid addiction: Time to and frequency of adoption. Journal of substance abuse treatment. 2018 Dec 1;95:26-34. | Study not about effectiveness |
| 740 | Helton BJ. Process Improvement: Facility wide Reduction in Hospital-Associated Infections Utilizing CHG for Oral Care and Preoperative Preparation. American Journal of Infection Control. 2012 Jun 1;40(5):e123. | Conference abstract |
| 741 | Hendricks-Muñoz KD, Prendergast CC. Barriers to provision of developmental care in the neonatal intensive care unit: neonatal nursing perceptions. American journal of perinatology. 2007 Dec;25(02):071-7. | Study not about effectiveness |
| 742 | Hendy J, Barlow J. The role of the organizational champion in achieving health system change. Social science & medicine. 2012 Feb 1;74(3):348-55. | Study not about effectiveness |
| 743 | Hennessy KA, Judy Dynan MS. Improving compliance with personal protective equipment use through the model for improvement and staff champions. Clinical journal of oncology nursing. 2014 Oct 1;18(5):497. | Not a champion |
| 744 | Henry J, Richardson M, Black-Pond C, Sloane M, Atchinson B, Hyter Y. A grassroots prototype for trauma-informed child welfare system change. Child Welfare. 2011 Nov 1;90(6). | Study not about effectiveness |
| 745 | Hensel D. Typologies of Professional Identity Among Graduating Baccalaureate‐Prepared Nurses. Journal of Nursing Scholarship. 2014 Mar;46(2):125-33. | Not about knowledge translation/evidence-based practice |
| 746 | Herrera CN, Brochier A, Pellicer M, Garg A, Drainoni ML. Implementing social determinants of health screening at community health centers: clinician and staff perspectives. Journal of primary care & community health. 2019 Nov;10:2150132719887260. | Study not about effectiveness |
| 747 | Herscher M., Mikhaylov D., Turakhia P., Barazani S., Sastow D., Lysov D.; Cushnie S., Cho H.The sleep hygeine in the hospital project: Shh! Journal of Hospital Medicine. 2018;13(4 Supplement 1) | Conference abstract |
| 748 | Hespe C, Rychetnik L, Peiris D, Harris M. Informing implementation of quality improvement in Australian primary care. BMC health services research. 2018 Dec;18(1):1-9. | Study not about effectiveness |
| 749 | Hess DR. How to initiate a noninvasive ventilation program: bringing the evidence to the bedside. Respiratory care. 2009 Feb 1;54(2):232-45. | Study not about effectiveness |
| 750 | Hewison A, Gale N, Yeats R, Shapiro J. An evaluation of staff engagement programmes in four National Health Service Acute Trusts. Journal of health organization and management. 2013 Mar 15. | Study not about effectiveness |
| 751 | Higuchi KS, Downey A, Davies B, Bajnok I, Waggott M. Using the NHS sustainability framework to understand the activities and resource implications of Canadian nursing guideline early adopters. Journal of Clinical Nursing. 2013 Jun;22(11-12):1707-16. | Study not about effectiveness |
| 752 | Hillman E, Paul J, Neustadt M, Reddy M, Wooldridge D, Dall L, Drees B. Establishing a multi-institutional quality and patient safety consortium: collaboration across affiliates in a community-based medical school. Academic Medicine. 2020 Dec;95(12). | Study not about effectiveness |
| 753 | Hinojosa C, Giardina J, Radtke K, Vournazos C. Heart Failure Core Measures-A Multidisciplinary Approach. Heart & Lung: The Journal of Cardiopulmonary and Acute Care. 2009 May 1;38(3):277. | Conference abstract |
| 754 | Hinton R, China Z, Brammar L, Burrows A, Collins P. Championing venous thromboembolism (VTE): Empowering junior doctors to improve VTE prophylaxis. InBRITISH JOURNAL OF HAEMATOLOGY 2018 Apr 1 (Vol. 181, pp. 55-55). 111 RIVER ST, HOBOKEN 07030-5774, NJ USA: WILEY. | Conference abstract |
| 755 | Ho M, Battaglia C, Haverhals LM, Fagan KM, Szarka JG, Alexander R, Sayre G, Helfrich C, Kirsh S, Au D, Aron D. A MIXED-METHODS EVALUATION OF A VA PATIENT-CENTERED MEDICAL HOME FOR SPECIALTY CARE. InJOURNAL OF GENERAL INTERNAL MEDICINE 2014 Apr 1 (Vol. 29, pp. S9-S10). 233 SPRING ST, NEW YORK, NY 10013 USA: SPRINGER. | Conference abstract |
| 756 | Hochman KA, Adler N, Gumbrecht L, Bosworth B. AN INTERDISCIPLINARY STRATEGY FOR IMPROVING HAND HYGIENE ON AN INPATIENT MEDICINE UNIT. InJOURNAL OF GENERAL INTERNAL MEDICINE 2017 Apr 1 (Vol. 32, pp. S115-S115). 233 SPRING ST, NEW YORK, NY 10013 USA: SPRINGER. | Conference abstract |
| 757 | Hockman-McDowell N. Reducing falls in the frail elderly. Journal of the American Medical Directors Association. 2018;19(3):B21 | Conference abstract |
| 758 | Hodgson L, Fairhurst A, Thorburn P, Frew AF, Doffman SR. P127 Improving smoking cessation advice through the implementation of a quality improvement intervention. Thorax. 2011 Dec 1;66(Suppl 4):A118-9. | Conference abstract |
| 759 | Hoesel L, Mouawad N, Silverman L, Brandt MM, Purtill MA, Bander J, Posa P. JUMPING THE HURDLES-BARRIERS DURING IMPLEMENTATION OF A SEPSIS PROGRAM. InCRITICAL CARE MEDICINE 2010 Dec 1 (Vol. 38, No. 12, pp. U110-U110). 530 WALNUT ST, PHILADELPHIA, PA 19106-3621 USA: LIPPINCOTT WILLIAMS & WILKINS. | Conference abstract |
| 760 | Hoffmann B., Moyer A. Leading quality change one beat at a time: Telemetry reduction in a university hospital. Journal of Hospital Medicine. 2011;6(4 SUPPL. 2):S114 | Conference abstract |
| 761 | Hofler L, Cordes S, Cwiak C, Goedken P, Jamieson D, Kottke M. Implementation of immediate postpartum LARC in Georgia. Contraception. 2016 Oct 1;94(4):425-6. | Conference abstract |
| 762 | Hofler LG, Cordes S, Cwiak CA, Goedken P, Jamieson DJ, Kottke M. Implementing immediate postpartum long-acting reversible contraception programs. Obstetrics & Gynecology. 2017 Jan 1;129(1):3-9. | Study not about effectiveness |
| 763 | Hofman JJ, Mohammed H. Experiences with facility-based maternal death reviews in northern Nigeria. International Journal of Gynecology & Obstetrics. 2014 Aug 1;126(2):111-4. | Study not about effectiveness |
| 764 | Hohlfelder B, Kubiak DW, Degrado JR, Reardon DP, Szumita PM. Implementation of a prolonged infusion guideline for time-dependent antimicrobial agents at a tertiary academic medical center. American journal of therapeutics. 2016 Nov 1;23(6):e1768-73. | Study not about effectiveness |
| 765 | Holder C. PRESSURE ULCER REDUCTION IN MEDICAL INTENSIVE CARE UNIT: SUCCESS OF MICU SKIN BREAKDOWN PREVENTION PROTOCOL. InCRITICAL CARE MEDICINE 2010 Dec 1 (Vol. 38, No. 12, pp. U214-U214). 530 WALNUT ST, PHILADELPHIA, PA 19106-3621 USA: LIPPINCOTT WILLIAMS & WILKINS. | Conference abstract |
| 766 | Holland R, Meyers D, Hildebrand C, Bridges AJ, Roach MA, Vogelman B. Creating champions for health care quality and safety. American Journal of Medical Quality. 2010 Mar;25(2):102-8. | Study not about effectiveness |
| 767 | Holmboe ES, Meehan TP, Radford MJ, Wang Y, Krumholz HM. What's Happening in Quality Improvement at the Local Hospital: A State Wide Study From the Cooperative Cardiovascular Project. American Journal of Medical Quality. 2000 May;15(3):106-13. | Study not about effectiveness |
| 768 | Holmboe ES, Meehan TP, Radford MJ, Wang Y, Marciniak TA, Krumholz HM. Use of critical pathways to improve the care of patients with acute myocardial infarction. The American journal of medicine. 1999 Oct 1;107(4):324-31. | Study not about effectiveness |
| 769 | Holmes CA. Postdisciplinarity in mental health‐care: an Australian viewpoint. Nursing Inquiry. 2001 Dec;8(4):230-9. | Study not about effectiveness |
| 770 | Holmes RD, Steele JG, Exley C, Vernazza CR, Donaldson C. Use of programme budgeting and marginal analysis to set priorities for local NHS dental services: learning from the north east of England. Journal of Public Health. 2018 Dec 1;40(4):e578-85. | Study not about effectiveness |
| 771 | Holmquist J. Zero Catheter Associated Urinary Tract Infections (CAUTIs): One Veteran's Affairs Hospital Experience in Medical-Surgical Areas. American Journal of Infection Control. 2011 Jun 1;39(5):E49. | Conference abstract |
| 772 | Hom LA, Martin GR. Newborn critical congenital heart disease screening using pulse oximetry: nursing aspects. American journal of perinatology. 2016 Sep;33(11):1072-5. | Study not about effectiveness |
| 773 | Hopkins JM, Glenn BA, Cole BL, McCarthy W, Yancey A. Implementing organizational physical activity and healthy eating strategies on paid time: process evaluation of the UCLA WORKING pilot study. Health Education Research. 2012 Jun 1;27(3):385-98. | Study not about effectiveness |
| 774 | Horst HM, Rubinfeld I, Mlynarek M, Brandt MM, Boleski G, Jordan J, Gnam G, Conway W. A tight glycemic control initiative in a surgical intensive care unit and hospitalwide. The Joint Commission Journal on Quality and Patient Safety. 2010 Jul 1;36(7):291-AP1. | Study not about effectiveness |
| 775 | Horvath S, Bumpus M, Luchowski A. From uptake to access: a decade of learning from the ACOG LARC program. American journal of obstetrics and gynecology. 2020 Apr 1;222(4):S866-8. | Study not about effectiveness |
| 776 | Houle-Burns R. Reduction of Ventilator Associated Pneumonia (VAP) in a Level II Adult Neuro/Trauma Unit Through Improved Use of Ventilator Bundles and Focused Nursing-Driven Ianterventions. American Journal of Infection Control. 2011 Jun 1;39(5):E194-5. | Conference abstract |
| 777 | Houzé-Cerfon CH, Boet S, Marhar F, Saint-Jean M, Geeraerts T. Simulation-based interprofessional education for critical care teams: Concept, implementation and assessment. Presse medicale (Paris, France: 1983). 2019 Jul 1;48(7-8 Pt 1):780-7. | Study not about effectiveness |
| 778 | Howell D, Rosberger Z, Mayer C, Faria R, Hamel M, Snider A, Lukosius DB, Montgomery N, Mozuraitis M, Li M. Personalized symptom management: a quality improvement collaborative for implementation of patient reported outcomes (PROs) in ‘real-world’oncology multisite practices. Journal of patient-reported outcomes. 2020 Dec;4(1):1-3. | Study not about effectiveness |
| 779 | Howell JM, Higgins CA. Champions of change: Identifying, understanding, and supporting champions of technological innovations. Organizational dynamics. 1990 Jun 1;19(1):40-55. | Not within a health care setting |
| 780 | Howell JM, Shea CM, Higgins CA. Champions of product innovations: defining, developing, and validating a measure of champion behavior. Journal of business venturing. 2005 Sep 1;20(5):641-61. | Not within a health care setting |
| 781 | Howell K, Karish C, Bigham M. 1416: WASTE NOT, WANT NOT: SAVING MEDICATION COSTS IN THE PEDIATRIC INTENSIVE CARE UNIT. Critical Care Medicine. 2019 Jan 1;47(1):684. | Conference abstract |
| 782 | Hoy AR, Patrick H, Campbell B, Lyratzopoulos G. Measuring the influence of colleagues on a consultant team's use of breast conserving surgery. International journal of technology assessment in health care. 2010 Apr;26(2):156-62. | Study not about effectiveness |
| 783 | Hoyte VE, Berger J, Pozzuoli L, Woods B. Decrease in VAP Rates Due to Interdisciplinary Collaboration. American Journal of Infection Control. 2013 Jun 1;41(6):S104-5. | Conference abstract |
| 784 | Hshieh TT, Yang T, Gartaganis SL, Yue J, Inouye SK. Hospital elder life program: systematic review and meta-analysis of effectiveness. The American Journal of Geriatric Psychiatry. 2018 Oct 1;26(10):1015-33. | Study not about effectiveness |
| 785 | Hsu A., Fouche S., Forman J., Hunt N., Shields T., Kronick S., Domeier R., Nelson C.D., Mendel P., Fetters M.D., Neumar R.W., Nallamothu B.K., Abir M. The role of champions for out-of-hospital cardiac arrest survival. Academic Emergency Medicine / 2020;27(Supplement 1):S253 | Conference abstract |
| 786 | Htun H, Ioannides S, Fishman T, Lawrenson R, Elwood M. INTERNATIONAL STUDY OF CANCER MANAGEMENT IN GENERAL PRACTICE (NEW ZEALAND): PRELIMINARY FINDINGS: 528. Asia-pacific Journal of Clinical Oncology. 2014 Dec;10. | Conference abstract |
| 787 | Hu S. PI21 collaborative quality improvement initiative drives pressure injuries to zero. Journal of wound ostomy and continence nursing 2020 may 1 (vol. 47, pp. S27-s28). | Conference abstract |
| 788 | Hudak ML, Cumbler E. Positive Deviance of Acute Ischemic Stroke Care: Time Truly is Brain at Best Practice Hospitals. | Conference abstract |
| 789 | Hudson P, Kupa A. ANAPHYLAXIS DISCHARGE MANAGEMENT “REDUCING THE RISK”: 36. Internal Medicine Journal. 2010 Sep;40. | Conference abstract |
| 790 | Hudson P, Kupa A. Optimising anaphylaxis discharge management: 95. Allergy: European Journal of Allergy and Clinical Immunology. 2010 Jun;65. | Conference abstract |
| 791 | Hudson SA. Systematic Literature Review on Fall Prevention in an Acute Care Hospital Setting (Doctoral dissertation, Walden University). | Letters to the editor/ Review studies |
| 792 | Huffstetler AN, Kuzel AJ, Sabo RT, Richards A, Brooks EM, Lail Kashiri P, Villalobos G, Arias AJ, Svikis D, Bortz BA, Edwards A. Practice facilitation to promote evidence-based screening and management of unhealthy alcohol use in primary care: a practice-level randomized controlled trial. BMC family practice. 2020 Dec;21:1-1. | Study not about effectiveness |
| 793 | Hughes ML, Weiss M. Adverse drug reaction reporting by community pharmacists—the barriers and facilitators. Pharmacoepidemiology and drug safety. 2019 Dec;28(12):1552-9. | Study not about effectiveness |
| 794 | Huijg JM, Crone MR, Verheijden MW, van der Zouwe N, Middelkoop BJ, Gebhardt WA. Factors influencing the adoption, implementation, and continuation of physical activity interventions in primary health care: a Delphi study. BMC family practice. 2013 Dec;14(1):1-9. | Study not about effectiveness |
| 795 | Hujcs M, Eckhardt D, Danielle M. Clinical Nurse Champions Improve Patient Outcome: Sustaining Catheter-Related BSI Reduction in Neurocritical Care: CS64. Critical Care Nurse. 2009 Apr;29(2). | Conference abstract |
| 796 | Hullick C, Rosen T, Stern M, Lachs M. Understanding Innovative Emergency Department Interventions for Older Adults.: B25. Journal of the American Geriatrics Society. 2010 Apr;58. | Conference abstract |
| 797 | Humphrey, M. The islington care home speech and langauge therapy dysphagia pilot project-2014-2016. Dysphagia / 2017;32(1):201 | Conference abstract |
| 798 | Hunt SC, Burgo-Black L. HEALTH IMPACTS OF WAR: AN EDUCATIONAL CAMPAIGN FOR VA PROVIDERS. InJOURNAL OF GENERAL INTERNAL MEDICINE 2011 May 1 (Vol. 26, pp. S589-S589). 233 SPRING ST, NEW YORK, NY 10013 USA: SPRINGER. | Conference abstract |
| 799 | Hurtado DA, Greenspan SA, Dumet LM, Heinonen GA. Use of Champions Identified by Social Network Analysis to Reduce Health Care Worker Patient-Assist Injuries. The Joint Commission Journal on Quality and Patient Safety. 2020 Nov 1;46(11):608-16. | Study not about effectiveness |
| 800 | Huxtable S, Palmer M. The efficacy of protected mealtimes in reducing mealtime interruptions and improving mealtime assistance in adult inpatients in an Australian hospital. European journal of clinical nutrition. 2013 Sep;67(9):904-10. | Study not about effectiveness |
| 801 | Huynh T, Kouz S, Afilalo M, Rinfret S, Schampaert E, Mansour S, Montigny M, Eisenberg M, Lauzon C, Dery JP, Nguyen M. Knowledge translation to improve prescription of evidence-based medical therapy for patients admitted with acute coronary syndromes: Insights from the AMI-OPTIMA study. Journal of the American College of Cardiology. 2015 Mar 17;65(10S):A5-. | Conference abstract |
| 802 | Huynh T, Tardif JC, Segal E, L’Allier P, Nguyen M, Dery JP, Afilalo M, Mansour S, Montigny M, Eisenberg M, Ross D. KNOWLEDGE TRANSLATION TO REDUCE DELAYS OF PRIMARY PERCUTANEOUS CORONARY INTERVENTION IN PATIENTS WITH MYOCARDIAL INFARCTION WITH ST-SEGMENT ELEVATION: INSIGHTS FROM THE AMI-ON TIME STUDY. Journal of the American College of Cardiology. 2015 Mar 17;65(10S):A134-. | Conference abstract |
| 803 | Huynh, A., Taylor, S. L., Dvorin, K., Bolton, R.,Elwy, A. R., Bokhour, B. G.; Whitehead, A., Kligler, B. What Should Health Care Systems Consider When Implementing Complementary and Integrative Health: Lessons from Veterans Health Administration. Journal of Alternative & Complementary Medicine. 2019;25():S52-S60 | Study not about effectiveness |
| 804 | Hyman S, Bernstein P, Shim SH, Messina M, Villamor J, Angevine P, Connolly S, Furuya Y, Katz M, Saiman L, Scully B. Reducing Surgical Post-operative Infections Through a Multidisciplinary Team Approach: Presentation Number 12-136. Ajic (american Journal of Infection Control). 2010 Jun;38(5). | Conference abstract |
| 805 | Hyman S, Kertesz L, Nelson P, Compton A, Almarez-Fox V, Evanko JC. Engaging staff to be responsible for surgical site infection prevention in a large academic tertiary hospital. American Journal of Infection Control. 2012 Jun 1;40(5):e157. | Conference abstract |
| 806 | Ibrahim K, May CR, Patel HP, Baxter M, Sayer AA, Roberts HC. Implementation of grip strength measurement in medicine for older people wards as part of routine admission assessment: identifying facilitators and barriers using a theory-led intervention. BMC geriatrics. 2018 Dec;18(1):1-4. | Study not about effectiveness |
| 807 | Iloabuchi T, Vannerson J, Butler D, Cottingham A, Alder C, McCabe H, Reed C, James F, Counsell SR. Integration of Geriatrics and Primary Care in Community Health Centers through Professional Development and Practice Improvement Strategies. InJOURNAL OF THE AMERICAN GERIATRICS SOCIETY 2017 May 1 (Vol. 65, pp. S3-S3). 111 RIVER ST, HOBOKEN 07030-5774, NJ USA: WILEY. | Conference abstract |
| 808 | Ilonze C, Idada F, Kraft M, Karns T, Webb K, Hanlon K, Journeycake J, Sinha A. IMPLEMENTATION OF PAIN ALGORITHM AND PROVIDER EDUCATION IMPROVES OUTCOMES IN SICKLE CELL DISEASE. InPEDIATRIC BLOOD & CANCER 2019 Jun 1 (Vol. 66). 111 RIVER ST, HOBOKEN 07030-5774, NJ USA: WILEY. | Conference abstract |
| 809 | Innes K, Jackson D, Plummer V, Elliott D. Emergency department waiting room nurse role: A key informant perspective. Australasian Emergency Nursing Journal. 2017 Feb 1;20(1):6-11. | Study not about effectiveness |
| 810 | Innis J, Dryden-Palmer K, Perreira T, Berta W. How do health care organizations take on best practices? A scoping literature review. International journal of evidence-based healthcare. 2015 Dec 1;13(4):254-72. | Letters to the editor/ Review studies |
| 811 | Insigne AG, Akin J, Thorsen R, Martinez J, Buchanan L, Nambiar A, Moayeri M, Bakhtary S, Nedelcu E. Blood Bank Ambassadors Bridge Communication Gap and Sustainably Improve Workflow Via Project Connect. In2018 Annual Meeting 2018 Oct 14. AABB. | Conference abstract |
| 812 | Irena A, Patel K, Thompson DB, Gesese A, Schleis GJ, Battiola RJ. Diabetes—Improved Service Efficiency Improves Racial Disparity. The Ochsner Journal. 2018 Mar 20;18(Spec AIAMC Iss):11-. | Conference abstract |
| 813 | Irimu GW, Greene A, Gathara D, Kihara H, Maina C, Mbori-Ngacha D, Zurovac D, Migiro S, English M. Factors influencing performance of health workers in the management of seriously sick children at a Kenyan tertiary hospital-participatory action research. BMC health services research. 2014 Dec;14(1):1-7. | Study not about effectiveness |
| 814 | Irving K, Piasek P, Coen A, Kilcullen S, Manning M. Irish National Dementia Educational Needs Analysis 2014. InIRISH JOURNAL OF MEDICAL SCIENCE 2014 Sep 1 (Vol. 183, pp. S301-S302). 236 GRAYS INN RD, 6TH FLOOR, LONDON WC1X 8HL, ENGLAND: SPRINGER LONDON LTD. | Conference abstract |
| 815 | Isaacksz S, Chang M. Delivering the digital backbone for integrated care in Ontario, Canada. International Journal of Integrated Care (IJIC). 2019 Aug 8;19. | Conference abstract |
| 816 | Iverson KM, Adjognon O, Grillo AR, Dichter ME, Gutner CA, Hamilton AB, Stirman SW, Gerber MR. Intimate partner violence screening programs in the Veterans Health Administration: informing scale-up of successful practices. Journal of general internal medicine. 2019 Nov;34(11):2435-42. | Not about knowledge translation/evidence-based practice |
| 817 | Iwata AJ, Olden HA, Kippen KE, Swegal WC, Johnson CC, Chang SS. Flexible model for patient engagement: achieving quality outcomes and building a research agenda for head and neck cancer. Head & neck. 2019 Apr;41(4):1087-93. | Study not about effectiveness |
| 818 | Jabbour M, Newton AS, Johnson D, Curran JA. Defining barriers and enablers for clinical pathway implementation in complex clinical settings. Implementation Science. 2018 Dec;13(1):1-3. | Study not about effectiveness |
| 819 | Jack S, Dobbins M, Tonmyr L, Dudding P, Brooks S, Kennedy B. Research evidence utilization in policy development by child welfare administrators. Child Welfare. 2010 Jul 1;89(4):83. | Not within a health care setting |
| 820 | Jack-Waugh A, Ritchie L, MacRae R. Assessing the educational impact of the dementia champions programme in Scotland: Implications for evaluating professional dementia education. Nurse education today. 2018 Dec 1;71:205-10. | Study not about effectiveness |
| 821 | Jacobs DM, Kuper K, Septimus E, Arafat R, Garey KW. Assessment of antimicrobial stewardship activities in a large metropolitan area. Journal of pharmacy practice. 2016 Jun;29(3):188-93. | Study not about effectiveness |
| 822 | Jacobson, J.Physician champions key to successful quality improvement projects. AHRQ Research Activities 09// 2010;(361):4-4 | Letters to the editor/ Review studies |
| 823 | Jain S, Walsh I, Shastri N, Conners G. Financial Stewardship through Appropriate Critical Care Procedure Documentation. | Conference abstract |
| 824 | Jan S, Steinway C, Greenberg A, Szalda D, Wu K, Kim R, Trachtenberg SW. A TIERED APPROACH TO TRANSITIONING YOUNG ADULTS WITH MEDICAL COMPLEXITY OR INTELLECTUAL DISABILITY TO ADULT CARE. InJOURNAL OF GENERAL INTERNAL MEDICINE 2017 Apr 1 (Vol. 32, pp. S731-S731). 233 SPRING ST, NEW YORK, NY 10013 USA: SPRINGER. | Conference abstract |
| 825 | Janamian T, Crossland LJ, Jackson C, Morcom J. Triggering change in diabetes care delivery in general practice: a qualitative evaluation approach using the clinical microsystem framework. BMC family practice. 2014 Dec;15(1):1-6. | Study not about effectiveness |
| 826 | Janaway D, O'Riordan S, Marshall J. NO ROOM FOR CLINICAL NIHILISM IN PREVENTING FALLS AND FRACTURES IN IN-PATIENTS. InOSTEOPOROSIS INTERNATIONAL 2010 Nov 1 (Vol. 21, pp. S495-S495). 236 GRAYS INN RD, 6TH FLOOR, LONDON WC1X 8HL, ENGLAND: SPRINGER LONDON LTD. | Conference abstract |
| 827 | Jardien-Baboo S, van Rooyen D, Ricks E, Jordan P, ten Ham-Baloyi W. Best practice guideline for patient-centred care in South African public hospitals. Africa Journal of Nursing and Midwifery. 2019 Dec 1;21(2):1-20. | Not about knowledge translation/evidence-based practice |
| 828 | Jeffs L, McIsaac W, Zahradnik M, Senthinathan A, Dresser L, McIntyre M, Tannenbaum D, Bell C, Morris A. Barriers and facilitators to the uptake of an antimicrobial stewardship program in primary care: A qualitative study. Plos one. 2020 Mar 5;15(3):e0223822. | Study not about effectiveness |
| 829 | Jegasothy R, Subramaniam T, Tukiman R, Jeganathan R. The A to J of Curricular Change in Midwifery Training in Malaysia: 43. Medical Education. 2014 Oct;48. | Conference abstract |
| 830 | Jenkins J, Calzone KA, Caskey S, Culp S, Weiner M, Badzek L. Methods of genomic competency integration in practice. Journal of Nursing Scholarship. 2015 May;47(3):200-10. | Study not about effectiveness |
| 831 | Jenkins T, Latchford T. A Successful Approach to Developing Primary Palliative Care Nurse Champions on a Blood and Marrow Transplant Unit. Biology of Blood and Marrow Transplantation. 2018 Mar 1;24(3):S474. | Conference abstract |
| 832 | Jenssen JI, Jørgensen G. How do corporate champions promote innovations?. International Journal of Innovation Management. 2004 Mar;8(01):63-86. | Not within a health care setting |
| 833 | Jeronimo J., Holme F. Strategies for updating national cervical cancer prevention guidelines: Experience from central America. International Journal of Gynecology and Obstetrics. 2015. 131(SUPPL. 5):E153 | Conference abstract |
| 834 | Jiwa M, Deas K, Ross J, Shaw T, Wilcox H, Spilsbury K. An inclusive approach to raising standards in general practice: working with a'community of practice'in Western Australia. BMC medical research methodology. 2009 Dec;9(1):1-8. | Study not about effectiveness |
| 835 | Joag K, Shields-Zeeman L, Kapadia-Kundu N, Kawade R, Balaji M, Pathare S. Feasibility and acceptability of a novel community-based mental health intervention delivered by community volunteers in Maharashtra, India: the Atmiyata programme. BMC psychiatry. 2020 Dec;20(1):1-4. | Not within a health care setting |
| 836 | John J.S., Burke D., Baumgarten K., Schmucker D., Rucker E., Chapman J., Smith A., Bailey B., Pennington C., Neal T. Reducing catheter-associated urinary tract infection and indwelling urinary catheter utilization through implementation of a nurse-driven foley removal protocol. Ochsner Journal. 2016; 16(3):389 | Conference abstract |
| 837 | John P, Chin M, Campbell A, Wilkes A, Vable A, Burnet D, Quinn M, Schaefer C, Heuer L. COACH: A COLLABORATIVE APPROACH TO QUALITY IMPROVEMENT FOR WEIGHT MANAGEMENT PROGRAMS IN FIVE MIDWESTERN HEALTH CENTERS. InJOURNAL OF GENERAL INTERNAL MEDICINE 2010 Jun 1 (Vol. 25, pp. 240-241). 233 SPRING ST, NEW YORK, NY 10013 USA: SPRINGER. | Conference abstract |
| 838 | Johnson AM, Kuperstein J, Howell D, Dupont-Versteegden EE. Physical therapists know function: an opinion on mobility and level of activity during hospitalization for adult inpatients. Hospital topics. 2018 Apr 3;96(2):61-8. | Study not about effectiveness |
| 839 | Johnson C. A literature review examining the barriers to the implementation of family witnessed resuscitation in the Emergency Department. International emergency nursing. 2017 Jan 1;30:31-5. | Study not about effectiveness |
| 840 | Johnson CV, Mimiaga MJ, Reisner SL, VanDerwarker R, Mayer KH. Barriers and facilitators to routine HIV testing: perceptions from Massachusetts Community Health Center personnel. AIDS patient care and STDs. 2011 Nov 1;25(11):647-55. | Study not about effectiveness |
| 841 | Johnson EE, Simpson AN, Harvey JB, Simpson KN. Bariatric surgery implementation trends in the USA from 2002 to 2012. Implementation Science. 2015 Dec;11(1):1-9. | Study not about effectiveness |
| 842 | Johnson EE, Sterba KR, Goodwin AJ, Warr EH, Beeks R, Zapka JM, Ford DW. Implementation of an academic-to-community hospital intensive care unit quality improvement program. Qualitative analysis of multilevel facilitators and barriers. Annals of the American Thoracic Society. 2019 Jul;16(7):877-85. | Study not about effectiveness |
| 843 | Johnson I, Donovan D, Parboosingh J. Steps to improve the teaching of public health to undergraduate medical students in Canada. Academic medicine. 2008 Apr 1;83(4):414-8. | Not within a health care setting |
| 844 | Johnson JE, Veneziano T, Green J, Howarth E, Malast T, Mastro K, Moran A, Mulligan L, Smith A. Breaking the fall. JONA: The Journal of Nursing Administration. 2011 Dec 1;41(12):538-45. | Study not about effectiveness |
| 845 | Johnson K, Johnstone H, McGougan T. The role of technology-enabled care in high-quality patient care. Practice Nursing. 2018 Aug 2;29(8):397-9. | Study not about effectiveness |
| 846 | Johnson K, Kennedy SB, Harris AO, Lincoln A, Neace W, Collins D. Strengthening the HIV/AIDS service delivery system in Liberia: an international research capacity‐building strategy. Journal of Evaluation in Clinical Practice. 2005 Jun;11(3):257-73. | Not about knowledge translation/evidence-based practice |
| 847 | Johnson M, Cross L, Sandison N, Stevenson J, Monks T, Moore M. Funding and policy incentives to encourage implementation of point-of-care C-reactive protein testing for lower respiratory tract infection in NHS primary care: a mixed-methods evaluation. BMJ open. 2018 Oct 1;8(10):e024558. | Study not about effectiveness |
| 848 | Johnson MJ, Leaf AA, Pearson F, Clark HW, Dimitrov BD, Pope C, May CR. Successfully implementing and embedding guidelines to improve the nutrition and growth of preterm infants in neonatal intensive care: a prospective interventional study. Bmj Open. 2017 Dec 1;7(12):e017727. | Study not about effectiveness |
| 849 | Johnson S, Hartigan S, Holt E, Sop D, McHenry C, Lipato T, Elliott T, Ferlis M, Mcmanus C, Smith WR. Using Lean Six Sigma to Develop a Patient Centered Medical Home for Adults with Sickle Cell Disease. | Conference abstract |
| 850 | Johnston B., Ahmed T., Al-Moasseb Z., Habgood M., Clarke S., Krige A.Improving the delivery of daily calorific targets via the enteral route in a critically ill patient population: A quality improvement cycle in a mixed surgical and medical intensive care unit in the United Kingdom | Conference abstract |
| 851 | Johnston C., Sen S., Greenhalgh D.G., Palmieri T.L. Do ventilator associated pneumonia prevention bundles work in burn intensive care units? Journal of Burn Care and Research / 2013;34(2 SUPPL. 1):S133 | Conference abstract |
| 852 | Johnston S. An analysis of the adoption and implementation of breastfeeding policies in Washington state clinics (Doctoral dissertation). | Study not about effectiveness |
| 853 | Johnston T, Atkinson R, Brackenridge A, Camfield E, Carroll J, Domingo J, Griffiths R, Gulati A, Wandel J, Sen Gupta P. The causes of hypoglycaemia in the inpatient setting: The views of health care professionals. InDIABETIC MEDICINE 2019 Mar 1 (Vol. 36, pp. 133-133). 111 RIVER ST, HOBOKEN 07030-5774, NJ USA: WILEY. | Conference abstract |
| 854 | Jones C, Gupta A. WHAT MATTERS MOST? PROMOTING PATIENT CENTRED HIP FRACTURE CARE. InOSTEOPOROSIS INTERNATIONAL 2018 Apr 1 (Vol. 29, pp. S282-S282). 236 GRAYS INN RD, 6TH FLOOR, LONDON WC1X 8HL, ENGLAND: SPRINGER LONDON LTD. | Conference abstract |
| 855 | Jones K, Griffiths L. Back to the floor Friday: evaluation of the impact on the patient experience. Journal of Nursing Management. 2011 Mar;19(2):170-6. | Study not about effectiveness |
| 856 | Jones, M. R., Hooper, T. J., Cuomo, C., Crouch, G., Hickam, T., Lestishock, L., Mennito, S., White, P.H. Evaluation of a Health Care Transition Improvement Process in Seven Large Health Care Systems. Journal of Pediatric Nursing. 2019;47():44-50 | Study not about effectiveness |
| 857 | Jordan L, Etienne L, Brownlee J, Venkataramani M, Pierre-Louis RL, Whiteman L, McFarland SA. Sickle Cell Day Hospital at Memorial Regional Hospital-A Solution That Works: 029. American Journal Of Hematology. 2011 Oct;86(10). | Conference abstract |
| 858 | Jordan P, Mpasa F, ten Ham-Baloyi W, Bowers C. Implementation strategies for guidelines at ICUs: a systematic review. International journal of health care quality assurance. 2017 May 8. | Letters to the editor/ Review studies |
| 859 | Jornsay DL, Garnett ED. Diabetes champions: culture change through education. Diabetes Spectrum. 2014 Aug 1;27(3):188-92. | Study not about effectiveness |
| 860 | Jortberg BT, Fernald DH, Hessler DM, Dickinson LM, Wearner R, Connelly L, Holtrop JS, Fisher L, Dickinson WP. Practice characteristics associated with better implementation of patient self-management support. The Journal of the American Board of Family Medicine. 2019 May 1;32(3):329-40. | Study not about effectiveness |
| 861 | Joshi M, Sahoo T, Thukral A, Joshi P, Sethi A, Agarwal R. Improving duration of kangaroo mother care in a tertiary-care neonatal unit: A quality improvement Initiative. Indian pediatrics. 2018 Sep;55(9):744-7. | Study not about effectiveness |
| 862 | Joshi RD, Zervos M, Kaljee LM, Shrestha B, Maki G, Prentiss T, Bajracharya D, Karki K, Joshi N, Rai SM. Evaluation of a Hospital-Based Post-Prescription Review and Feedback Pilot in Kathmandu, Nepal. The American journal of tropical medicine and hygiene. 2019 Oct;101(4):923. | Study not about effectiveness |
| 863 | Joyner K, Mash B. A comprehensive model for intimate partner violence in South African primary care: action research. BMC Health Services Research. 2012 Dec;12(1):1-0. | Not about knowledge translation/evidence-based practice |
| 864 | Joyner K, Mash B. Quality of care for intimate partner violence in South African primary care: A qualitative study. Violence and victims. 2014 Jan 1;29(4):652-69. | Not about knowledge translation/evidence-based practice |
| 865 | Judy LR, Boineau J, Brown A. Secondary Fracture Prevention Programs: Experiences From the Field. Journal of Clinical Densitometry. 2018 Jan 1;21(1):27. | Conference abstract |
| 866 | Kaasalainen S, Brazil K, Akhtar-Danesh N, Coker E, Ploeg J, Donald F, Martin-Misener R, DiCenso A, Hadjistavropoulos T, Dolovich L, Papaioannou A. The evaluation of an interdisciplinary pain protocol in long term care. Journal of the American Medical Directors Association. 2012 Sep 1;13(7):664-e1. | Study not about effectiveness |
| 867 | Kaasalainen S, Ploeg J, Donald F, Coker E, Brazil K, Martin-Misener R, Dicenso A, Hadjistavropoulos T. Positioning clinical nurse specialists and nurse practitioners as change champions to implement a pain protocol in long-term care. Pain Management Nursing. 2015 Apr 1;16(2):78-88. | Study not about effectiveness |
| 868 | Kabululu EK, Ray-Barruel G, Alexandrou E, Rickard CM. One million global catheters PIVC worldwide prevalence study: building alliances to put vascular access device management and infection prevention on the world stage from Australia to the Democratic Republic of Congo. Antimicrobial Resistance and Infection Control. 2015 Dec;4(1):1-2. | Conference abstract |
| 869 | Kaelin K, Okland K. Buildings, Barriers, and Breakthroughs. Nursing administration quarterly. 2018 Jan 1;42(1):15-25. | Study not about effectiveness |
| 870 | Kahwati LC, Lewis MA, Kane H, Williams PA, Nerz P, Jones KR, Lance TX, Vaisey S, Kinsinger LS. Best practices in the Veterans Health Administration's MOVE! Weight management program. American journal of preventive medicine. 2011 Nov 1;41(5):457-64. | Study not about effectiveness |
| 871 | Kairy D, Messier F, Zidarov D, Ahmed S, Poissant L, Rushton PW, Vincent C, Fillion B, Lavoie V. Evaluating the implementation process of a new telerehabilitation modality in three rehabilitation settings using the normalization process theory: study protocol. International Journal of Healthcare Management. 2017 May 8. | Study not about effectiveness |
| 872 | Kairy D, Poissant L, Rushton P, Messier F, Vincent C, Zidarov D, Ahmed S, Fillion B, Lavoie V. Telerehabilitation implementation and routine clinical use: Preliminary findings from a case study across three rehabilitation centers. In2017 International Conference on Virtual Rehabilitation (ICVR) 2017 Jun 19 (pp. 1-2). IEEE. | Study not about effectiveness |
| 873 | Kaiser K, McGuire D, Shanholtz C, Haisfield-Wolfe ME, Deeley M. Implementing an Evidence-Based Tool for Assessing Pain in Non-Communicative Palliative Care Patients: Challenges And Solutions (S752). Journal of Pain and Symptom Management. 2016 Feb 1;51(2):435-6. | Conference abstract |
| 874 | Kaiser SV, Jennings B, Rodean J, Cabana MD, Garber MD, Ralston SL, Fassl B, Quinonez R, Mendoza JC, McCulloch CE, Parikh K. Pathways for improving inpatient pediatric asthma care (PIPA): a multicenter, national study. Pediatrics. 2020 Jun 1;145(6). | Study not about effectiveness |
| 875 | Kaiser SV, Johnson MD, Walls TA, Teach SJ, Sampayo EM, Dudley NC, Zorc JJ. Pathways to improve pediatric asthma care: a multisite, national study of emergency department asthma pathway implementation. The Journal of pediatrics. 2020 Aug 1;223:100-7. | Study not about effectiveness |
| 876 | Kalfon P, Alessandrini M, Boucekine M, Renoult S, Geantot MA, Deparis-Dusautois S, Berric A, Collange O, Floccard B, Mimoz O, Julien A. Tailored multicomponent program for discomfort reduction in critically ill patients may decrease post-traumatic stress disorder in general ICU survivors at 1 year. Intensive care medicine. 2019 Feb;45(2):223-35. | Conference abstract |
| 877 | Kalluri D, Davila J, Rinke M, Shoberu B, Edusei G, Rosario J, Manwani D, Morrone K. Pediatric Sickle Cell Action Plan: Decreasing Acute Care Utilization for Vaso Occlusive Crisis. InPEDIATRIC BLOOD & CANCER 2017 Jun 1 (Vol. 64, pp. S22-S22). 111 RIVER ST, HOBOKEN 07030-5774, NJ USA: WILEY. | Conference abstract |
| 878 | Kamal AH, Bowman B, Ritchie CS. Identifying palliative care champions to promote high‐quality care to those with serious illness. Journal of the American Geriatrics Society. 2019 May;67(S2):S461-7. | Study not about effectiveness |
| 879 | Kamal AH, Quinn D, Gilligan TD, Davis BC, Dalby CK, Bretsch J, McNiff KK, Jacobson JO, Kamal AH, Quinn D, Gilligan TD. ReCAP: Feasibility and effectiveness of a pilot program to facilitate quality improvement learning in oncology: Experience of the American Society of Clinical Oncology Quality Training Program. Journal of oncology practice. 2016 Feb;12(2):177-. | Study not about effectiveness |
| 880 | Kamal AH, Quinn D, Gilligan TD, Davis BC, Dalby CK, Bretsch J, McNiff KK, Jacobson JO, Kamal AH, Quinn D, Gilligan TD. ReCAP: Feasibility and effectiveness of a pilot program to facilitate quality improvement learning in oncology: Experience of the American Society of Clinical Oncology Quality Training Program. Journal of oncology practice. 2016 Feb;12(2):177-. | Study not about effectiveness |
| 881 | Karmali K, Grobovsky L, Levy J, Keatings M. Enhancing cultural competence for improved access to quality care. Healthcare Quarterly (Toronto, Ont.). 2011 Jan 1;14:52-7. | Study not about effectiveness |
| 882 | Kartha A, Restuccia JD, Burgess Jr JF, Benzer J, Glasgow J, Hockenberry J, Mohr DC, Kaboli PJ. Nurse practitioner and physician assistant scope of practice in 118 acute care hospitals. Journal of Hospital Medicine. 2014 Oct;9(10):615-20. | Conference abstract |
| 883 | Karwoski, J. E. Identifying informal advisors among neuromuscular specialists. Dissertation Abstracts International: Section B: The Sciences and Engineering .2007;67(12-B):7399 | Study not about effectiveness |
| 884 | Kathol RG, Butler M, McAlpine DD, Kane RL. Barriers to physical and mental condition integrated service delivery. Psychosomatic medicine. 2010 Jul 1;72(6):511-8. | Study not about effectiveness |
| 885 | Katzman J.G.,Comerci G., Duhigg D., Boyle J.G., Olivas C. Unm echo-pain and headache program. Headache . 2012;52(5):887-888 | Conference abstract |
| 886 | Kaufman J, Attwell K, Hauck Y, Leask J, Omer SB, Regan A, Danchin M. Designing a multi-component intervention (P3-MumBubVax) to promote vaccination in antenatal care in Australia. Health Promotion Journal of Australia: Official Journal of Australian Association of Health Promotion Professionals. 2021 Jul;32(3):391-8. | Study not about effectiveness |
| 887 | Kawuwa MB, Mairiga AG, Usman HA. Community perspective of maternal mortality: experience from Konduga local government area, Borno state, Nigeria. Annals of African medicine. 2007 Sep 1;6(3):109. | Study not about effectiveness |
| 888 | Kay R, Cox M, McKean BI, Gunning J, Hucker N, Meyer K, Briand ME. Preventing Healthcare Associated Blood Stream Infections Among Patients Suspected of Line Manipulation. American Journal of Infection Control. 2016 Jun 2;44(6):S30-1. | Conference abstract |
| 889 | Kay R, Wadsworth T. Utilizing physician leadership to reduce length of stay. Medical group management journal. 1995 Sep 1;42(5):66-8. | Study not about effectiveness |
| 890 | Kay Wagner DH. Health care reform and leadership: switching from volume to value. Physician leadership journal. 2014 Sep 1;1(1):22. | Study not about effectiveness |
| 891 | Kayyali A, Joy SD. Preventing Central Line Infections with a ‘Quality Nurse’. AJN The American Journal of Nursing. 2014 Jul 1;114(7):55. | Study not about effectiveness |
| 892 | Kearns M, Collier D, Curran M, McCarron P. From local organisational harmonisation to Global change leader: a story of operational success. International Journal of Integrated Care. 2017 Oct 17;17(5). | Conference abstract |
| 893 | Keating N, Tuakli ER, Fornasari L, Townsend M, Conneally K. Physiotherapists can ́make every contact count́ to promote smoking cessation; a quality improvement project on a vascular ward. Physiotherapy. 2020 May 1;107:e179-80. | Conference abstract |
| 894 | Keboa M, Beaudin A, Cyr J, Decoste J, Power F, Hovey R, LaFrance L, Ouellet D, Wiseman M, Macdonald ME. Dentistry and nursing working together to improve oral health care in a long-term care facility. Geriatric Nursing. 2019 Mar 1;40(2):197-204. | Study not about effectiveness |
| 895 | Keijser WA, Penterman L, van Montfort AP, Smits JG, Wilderom CP. The 7 Habits of Highly Effective Implementation of eHealth Enabled Integrated Care. International journal of integrated care. 2017;17(5):A486. | Conference abstract |
| 896 | Kelleher AD, Moorer A, Makic MF. Peer-to-peer nursing rounds and hospital-acquired pressure ulcer prevalence in a surgical intensive care unit: a quality improvement project. Journal of Wound Ostomy & Continence Nursing. 2012 Mar 1;39(2):152-7. | Study not about effectiveness |
| 897 | Keller C, Edenius M, Lindblad S. Adopting proactive knowledge use as an innovation: The case of a knowledge management system in rheumatology.Conference on Information Systems.2009 | Study not about effectiveness |
| 898 | Keller, C. A third person in the room: A case study of the swedish rheumatoid register.Conference on Information Systems.2017 | Study not about effectiveness |
| 899 | Kelley A, Aston L. An evaluation of using champions to enhance inter-professional learning in the practice setting. Nurse education in practice. 2011 Jan 1;11(1):36-40. | Study not about effectiveness |
| 900 | Kelley,​ T.,​ Jansen,​ D.,​ Richards,​ B.,​ Radice,​ N. How Orlando Health stemmed healthcare costs by eliminating unnecessary clinical process variation. Healthcare Financial Management 2020;74(3):32-36 | Study not about effectiveness |
| 901 | Kelloway LJ, Ireland S, Avinoam G, Bursey S, Edwards E, Fleck R, Gryfe P, Lumley-Leger K, Morrison K, Saulnier S, Skrabka K. Laying the foundation for knowledge translation in the Ontario stroke system. InSTROKE 2011 Nov 1 (Vol. 42, No. 11, pp. E624-E625). 530 WALNUT ST, PHILADELPHIA, PA 19106-3621 USA: LIPPINCOTT WILLIAMS & WILKINS. | Conference abstract |
| 902 | Kelly AM, Pannifex J. Improving management of atrial fibrillation across a health system using a clinical network. Emergency Medicine Australasia. 2015 Jun;27:13-4. | Conference abstract |
| 903 | Kelly D., Chaudhry S. An assessment and examination of the implementation, and barriers to implementation, of National Patient Safety Agency (NPSA) alerts. International Journal of Pharmacy Practice. 2013;21(SUPPL. 2):136-137 | Conference abstract |
| 904 | Kelly K, Christians J. Best practices in implementing a telehealth program. Caring: National Association for Home Care magazine. 2008 Jul 1;27(7):44-7. | Study not about effectiveness |
| 905 | Kelly T, Blankinship M. 374: Overcoming barriers to respiratory therapist engagement in implementation of the ABCDE bundle. Critical Care Medicine. 2013 Dec 1;41(12):A88-9. | Conference abstract |
| 906 | Kelman, GB.; Jadlos, MA. FACILITY-ACQUIRED PRESSURE INJURIES (FAPIs) IN ACUTE CARE 2009-2019...Scientific and Clinical Abstracts From WOCNext 2020 Reimagined, June 5-7, 2020. Journal of Wound, Ostomy & Continence Nursing Supplement 2020;47():S39-S39 | Conference abstract |
| 907 | Kemp K, Zelle H, Bonnie RJ. Embedding advance directives in routine care for persons with serious mental illness: implementation challenges. Psychiatric Services. 2015 Jan 1;66(1):10-4. | Study not about effectiveness |
| 908 | Kendall E, Ehrlich C, Young M, Muenchberger H, Wilkie K, Rushton C. Health partnerships: perspectives of medical practitioners in general practice, health systems and hospital settings. Australian Journal of Primary Health. 2009 Dec 17;15(4):319-25. | Study not about effectiveness |
| 909 | Kendra MS, Dang J, Artandi M, Vemuri M. Connecting tobacco users in the primary care setting to comprehensive tobacco treatment: a quality improvement initiative. Journal of Public Health. 2020 Oct 21:1-6. | Study not about effectiveness |
| 910 | Kennedy CC, Ioannidis G, Giangregorio LM, Adachi JD, Thabane L, Morin SN, Crilly RG, Marr S, Josse RG, Lohfeld L, Pickard LE. An interdisciplinary knowledge translation intervention in long-term care: Study protocol for the vitamin D and osteoporosis study (ViD OS) pilot cluster randomized controlled trial. Implementation Science. 2012 Dec;7(1):1-2. | Study not about effectiveness |
| 911 | Kennedy ED, Milot L, Fruitman M, Al-Sukhni E, Heine G, Schmocker S, Brown G, McLeod RS. Development and implementation of a synoptic MRI report for preoperative staging of rectal cancer on a population-based level. Diseases of the colon & rectum. 2014 Jun 1;57(6):700-8. | Study not about effectiveness |
| 912 | Kennedy L, Pinkney S, Suleman S, Mâsse LC, Naylor PJ, Amed S. Propagating change: using RE-FRAME to scale and sustain a community-based childhood obesity prevention initiative. International journal of environmental research and public health. 2019 Jan;16(5):736. | Not within a health care setting |
| 913 | Kennedy R, Binns F, Brammer A, Grant J, Bowen J, Morgan R. Continuous service quality improvement and change management for children and young people with autism and their families: A model for change. Comprehensive child and adolescent nursing. 2016 Jul 2;39(3):192-214. | Study not about effectiveness |
| 914 | Kennedy R, Binns F. Communicating and managing children and young people with autism and extensive burn injury. Wounds UK. 2014 Sep 16;10(3):60-5. | Study not about effectiveness |
| 915 | Kennedy V. Implementation of Computerized Distress Screening: Challenges and Best Practices With Cancer Center and Community Organization Providers: 17–3. Pscyho-oncology. 2014 Feb;23. | Conference abstract |
| 916 | Kenny DJ, Goodman P. Care of the patient with enteral tube feeding: an evidence-based practice protocol. Nursing research. 2010 Jan 1;59(1):S22-31. | Not a champion |
| 917 | Kenyon S, Dann S, Hope L, Clarke P, Hogan A, Jenkinson D, Hemming K. Evaluation of a bespoke training to increase uptake by midwifery teams of NICE Guidance for membrane sweeping to reduce induction of labour: a stepped wedge cluster randomised design. Trials. 2017 Dec;18(1):1-0. | Study not about effectiveness |
| 918 | Kerber KJ, Mathai M, Lewis G, Flenady V, Erwich JJ, Segun T, Aliganyira P, Abdelmegeid A, Allanson E, Roos N, Rhoda N. Counting every stillbirth and neonatal death through mortality audit to improve quality of care for every pregnant woman and her baby. BMC pregnancy and childbirth. 2015 Dec 1;15(S2):S9. | Study not about effectiveness |
| 919 | Keshvani N, Berger K, Gupta A, DePaola S, Nguyen OK, Makam AN. Improving Respiratory Rate Accuracy in the Hospital: A Quality Improvement Initiative. Journal of hospital medicine. 2019 Nov;14(11):673. | Conference abstract |
| 920 | Kester-Greene N, Cocco C, DeSousa S, Thomas-Boaz W, Nathens A, Burgess R, Ramagnano S, Filipowska C, Mazurik L. P078: If you build it they will come: use of live actor patients during a hospital-wide mass casualty simulation exercise to garner institutional commitment to long term drills. Canadian Journal of Emergency Medicine. 2018 May;20(S1):S84-. | Conference abstract |
| 921 | Kester-Greene N, Cocco C, DeSousa S, Thomas-Boaz W, Nathens A, Burgess R, Ramagnano S, Filipowska C, Mazurik L. P078: If you build it they will come: use of live actor patients during a hospital-wide mass casualty simulation exercise to garner institutional commitment to long term drills. Canadian Journal of Emergency Medicine. 2018 May;20(S1):S84- | Conference abstract |
| 922 | Ketelaar M, Harmer-Bosgoed M, Willems M, ROSENBAUM P. Moving research evidence into clinical practice: the role of knowledge brokers: IC 12. Developmental Medicine & Child Neurology. 2010 May;52:66-7. | Conference abstract |
| 923 | Ketelaar M, Harmer-Bosgoed M, Willems M, VERSCHUREN O, VERHOEF M. The role of knowledge brokers in the implementation of evidence-based measures in multidisciplinary teams: an example of the GMFCS and MACS: I10. Developmental Medicine & Child Neurology. 2010 Sep;52. | Conference abstract |
| 924 | Khan H, Sadeghi C, Tiemeier A, Bakhai S. Improving blood pressure control by optimization of guideline directed therapy for hypertension in a primary care setting: quality improvement project. Journal of Hypertension. 2019 Jul 1;37:e188. | Conference abstract |
| 925 | Khanna N, Klyushnenkova E, Montgomery R. Hypertension and Diabetes Quality Improvement in a Practice Transformation Network. American Journal of Medical Quality. 2020 Dec;35(6):486-90. | Study not about effectiveness |
| 926 | Khasnabish S, Duckworth M, Adelman J, Alfieri L, Kurian S, Lindros ME, Ryan V, Scanlan M, Spivack LB, Yu SP, Dykes P. The use of an audit tool to evaluate implementation of an evidence-based fall prevention program. Journal of General Internal Medicine. 2018 Apr 1 (Vol. 33, pp. S368-S368). 233 | Conference abstract |
| 927 | Khera N, Mau LW, Denzen EM, Houg K, Lee SJ, Horowitz MM, Burns L. Understanding Physicians' Perspectives About Translating Research Into Clinical Practice: Example of Blood and Marrow Transplant Clinical Trials Network (BMT CTN) 0201 Results. Biology of Blood and Marrow Transplantation. 2017 Mar 1;23(3):S71-2. | Conference abstract |
| 928 | Kibidi T., Muiruri K., Kiptoo O., Mutegi J., Muthee V., Walumbe S., Njeru M. Kenyaemr on-site mentorship in nyandarua county enhances more ownership and sustainability of the system. International Journal of Gynecology and Obstetrics . 2015;131(SUPPL. 5):E247-E248 | Conference abstract |
| 929 | Kiel JM. Using Organizational Development for Electronic Medical Record Transformation. The health care manager. 2016 Oct 1;35(4):305-11. | Study not about effectiveness |
| 930 | Kielly-Carroll C, Shaw T, White K, McGregor D, Avery S, Manley S, Webb G, Delaney L, Rankin N. A framework and toolkit for mapping and localizing cancer referral and diagnosis pathways. Asia-pacific journal of clinical oncology 2016 Nov 1 (Vol. 12, pp. 153-153). | Conference abstract |
| 931 | Kim CS, Spahlinger DA, Kin JM, Coffey RJ, Billi JE. Implementation of lean thinking: one health system’s journey. The Joint Commission Journal on Quality and Patient Safety. 2009 Aug 1;35(8):406-AP4. | Not a champion |
| 932 | Kim P, Daly JM, Berkowitz S, Levy BT. Use of the Fluoride Varnish Billing Code in a Tertiary Care Center Setting. Journal of primary care & community health. 2020 Mar;11. | Study not about effectiveness |
| 933 | Kim SC, Ecoff L, Brown CE, Gallo AM, Stichler JF, Davidson JE. Benefits of a regional evidence‐based practice fellowship program: A test of the ARCC model. Worldviews on Evidence‐Based Nursing. 2017 Apr;14(2):90-8. | Study not about effectiveness |
| 934 | Kim YM, Lee SJ, Jo SJ, Park KN. Implementation of the guidelines for targeted temperature management after cardiac arrest: a longitudinal qualitative study of barriers and facilitators perceived by hospital resuscitation champions. BMJ open. 2016 Jan 1;6(1):e009261. | Study not about effectiveness |
| 935 | Kimber C, Grimmer-Somers K. Preventing osteoporosis-related fractures from happening (again). International Journal of Orthopaedic and Trauma Nursing. 2011 Aug 1;15(3):121-35. | Study not about effectiveness |
| 936 | King C, Iuliano A, Burgess RA, Agwai I, Ahmar S, Aranda Z, Bahiru S, Bakare AA, Colbourn T, Shittu F, Graham H. A mixed‐methods evaluation of stakeholder perspectives on pediatric pneumonia in Nigeria—priorities, challenges, and champions. Pediatric pulmonology. 2020 Jun;55:S25-33. | Not a champion |
| 937 | Kinsinger LS, Jones KR, Kahwati L, Harvey R, Burdick M, Zele V, Yevich SJ. Peer reviewed: design and dissemination of the MOVE! Weight-Management Program for veterans. Preventing chronic disease. 2009 Jul;6(3). | Study not about effectiveness |
| 938 | Kirchner JE, Parker LE, Bonner LM, Fickel JJ, Yano EM, Ritchie MJ. Roles of managers, frontline staff and local champions, in implementing quality improvement: stakeholders' perspectives. Journal of Evaluation in Clinical Practice. 2012 Feb;18(1):63-9. | Study not about effectiveness |
| 939 | Kirk AP, McGlinsey A, Beckett A, Rudd P, Arbour R. Restraint reduction, restraint elimination, and best practice: role of the clinical nurse specialist in patient safety. Clinical Nurse Specialist. 2015 Nov 1;29(6):321-8. | Duplicate |
| 940 | Kirk AP, McGlinsey A, Beckett A, Rudd P, Arbour R. Restraint reduction, restraint elimination, and best practice: role of the clinical nurse specialist in patient safety. Clinical Nurse Specialist. 2015 Nov 1;29(6):321-8. | Duplicate |
| 941 | Kirk AP, McGlinsey A, Beckett A, Rudd P, Arbour R. Restraint reduction, restraint elimination, and best practice: role of the clinical nurse specialist in patient safety. Clinical Nurse Specialist. 2015 Nov 1;29(6):321-8. | Study not about effectiveness |
| 942 | Kirk AP, McGlinsey A, Beckett A, Rudd P, Arbour R. Restraint reduction, restraint elimination, and best practice: role of the clinical nurse specialist in patient safety. Clinical Nurse Specialist. 2015 Nov 1;29(6):321-8. | Study not about effectiveness |
| 943 | Kitchen-Clark T. Implementation of oral care best practices. International Journal of Stroke. 2015;10(SUPPL. 4):66 | Conference abstract |
| 944 | Kitson AL. Approaches used to implement research findings into nursing practice: report of a study tour to Australia and New Zealand. International Journal of Nursing Practice. 2001 Dec;7(6):392-405. | Study not about effectiveness |
| 945 | Klaassen LA, Friesen-Storms JH, Bours GJ, Dirksen CD, Boersma LJ, Hoving C. Perceived facilitating and limiting factors for healthcare professionals to adopting a patient decision aid for breast cancer aftercare: A cross-sectional study. Patient education and counseling. 2020 Jan 1;103(1):145-51. | Study not about effectiveness |
| 946 | Kleidon TM, Cattanach P, Mihala G, Ullman AJ. Implementation of a paediatric peripheral intravenous catheter care bundle: a quality improvement initiative. Journal of paediatrics and child health. 2019 Oct;55(10):1214-23. | Study not about effectiveness |
| 947 | Klinga C, Hasson H, Sachs MA, Hansson J. Dynamics of sustainable integration of health and social care: A 20-year case study. International Journal of Integrated Care (IJIC). 2018 Oct 2;18. | Conference abstract |
| 948 | Knapp H, Hagedorn H, Anaya HD. HIV rapid testing in a Veterans Affairs hospital ED setting: a 5-year sustainability evaluation. The American journal of emergency medicine. 2014 Aug 1;32(8):878-83. | Study not about effectiveness |
| 949 | Knerr S, West KM, Angelo FA. Organizational readiness to implement population‐based screening and genetic service delivery for hereditary cancer prevention and control. Journal of genetic counseling. 2020 Oct;29(5):867-76. | Study not about effectiveness |
| 950 | Knight AW, Szucs C, Dhillon M, Lembke T, Mitchell C. The eCollaborative: using a quality improvement collaborative to implement the National eHealth Record System in Australian primary care practices. International journal for quality in health care. 2014 Aug 1;26(4):411-7. | Study not about effectiveness |
| 951 | Knott A, Pathak S, McGrath JS, Kennedy R, Horgan A, Mythen M, Carter F, Francis NK. Consensus views on implementation and measurement of enhanced recovery after surgery in England: Delphi study. BMJ open. 2012 Jan 1;2(6):e001878. | Study not about effectiveness |
| 952 | Knox M, Murphy EJ, Leslie T, Wick R, Tuot DS. e-Consult implementation success: lessons from 5 county-based delivery systems. Am J Manag Care. 2020 Jan 1;26(1):e21-7. | Study not about effectiveness |
| 953 | Kocher KE, Pribble JM, Uren BJ, Macy ML, Ham JJ, Proudlock AL, Didyk JS, White EN, Nypaver MM. 333 Creating a Regional Quality Collaborative in Emergency Medicine: The Michigan Emergency Department Improvement Collaborative. Annals of Emergency Medicine. 2017 Oct 1;70(4):S132. | Conference abstract |
| 954 | Koczwara B, Barton M, Blinman PL, Crossing S, Grimison PS, Walpole ET, Wong N, Francis K. The shortage of medical oncologists and low chemotherapy utilization in Australia. Journal of Clinical Oncology. 2010 May 20;28(15_suppl):6104-. | Conference abstract |
| 955 | Koebner IJ, Fishman SM, Paterniti D, Sommer D, Ward D, Joseph JG. Curating care: The design and feasibility of a partnership between an art museum and an academic pain center. Curator: The Museum Journal. 2018 Jul;61(3):415-29. | Study not about effectiveness |
| 956 | Kogan JN, Schuster J, Nikolajski C, Schake P, Carney T, Morton SC, Kang C, Reynolds III CF. Challenges encountered in the conduct of optimal health: a patient-centered comparative effectiveness study of interventions for adults with serious mental illness. Clinical Trials. 2017 Feb;14(1):5-16. | Study not about effectiveness |
| 957 | Kokotis,​ K.A. (2014). The PICC team Peripherally Inserted Central Venous Catheters. 2014:165-186 | Study not about effectiveness |
| 958 | Kolb W, Harper B. Using the RE-AIM framework to evaluate and guide a quality improvement program: Implementation of low back pain clinical practice guidelines. Manual Therapy. 2016;100(25):e129-30. | Conference abstract |
| 959 | Kollef M. SMART approaches for reducing nosocomial infections in the ICU. Chest. 2008 Aug 1;134(2):447-56. | Study not about effectiveness |
| 960 | Konrad, S.C. Random acts of innovation: Infiltrating silos and creating an interprofessional culture for quality care. Journal of Prevention, Assessment & Rehabilitation / 2012;41(3):229-231 | Study not about effectiveness |
| 961 | Korenvain C, MacKeigan L, Dainty K, Guilcher SJ, McCarthy L. Exploring deprescribing opportunities for community pharmacists: Protocol for a qualitative study. Canadian Pharmacists Journal/Revue Des Pharmaciens Du Canada. 2018 Jul;151(4):228-32. | Study not about effectiveness |
| 962 | Kothari A, Boyko JA, Conklin J, Stolee P, Sibbald SL. Communities of practice for supporting health systems change: a missed opportunity. Health Research Policy and Systems. 2015 Dec;13(1):1-9. | Study not about effectiveness |
| 963 | Kourouche S, Buckley T, Van C, Munroe B, Curtis K. Designing strategies to implement a blunt chest injury care bundle using the behaviour change wheel: a multi-site mixed methods study. BMC health services research. 2019 Dec;19(1):1-7. | Study not about effectiveness |
| 964 | Kowalski CP, Veeser M, Heisler M. Formative evaluation and adaptation of pre-and early implementation of diabetes shared medical appointments to maximize sustainability and adoption. BMC family practice. 2018 Dec;19(1):1-23. | Study not about effectiveness |
| 965 | Kraut A, Graff L, McLean D. Behavioral change with influenza vaccination: factors influencing increased uptake of the pandemic H1N1 versus seasonal influenza vaccine in health care personnel. Vaccine. 2011 Oct 26;29(46):8357-63. | Study not about effectiveness |
| 966 | Krist AH, Woolf SH, Bello GA, Sabo RT, Longo DR, Kashiri P, Etz RS, Loomis J, Rothemich SF, Peele JE, Cohn J. Engaging primary care patients to use a patient-centered personal health record. The Annals of Family Medicine. 2014 Sep 1;12(5):418-26. | Study not about effectiveness |
| 967 | Krivoy M, Elledge C, Downey C. Implementation of Chemotherapy Order and Administration Checklists Ensures Adherence to National Chemotherapy Guidelines. Biology of Blood and Marrow Transplantation. 2015 Feb 1;21(2):S254. | Conference abstract |
| 968 | Krupp A, Steege L, King B. A systematic review evaluating the role of nurses and processes for delivering early mobility interventions in the intensive care unit. Intensive and Critical Care Nursing. 2018 Aug 1;47:30-8. | Letters to the editor/ Review studies |
| 969 | Kuhn JM. Using Action Research to Support an On-going Organizational Culture Transformation in a Health Care Organization (Doctoral dissertation, University of Minnesota). | Study not about effectiveness |
| 970 | Kumar A, Nesbitt KM, Bakkum-Gamez JN. Quality improvement in gynecologic oncology: Current successes and future promise. Gynecologic oncology. 2019 Mar 1;152(3):486-91. | Study not about effectiveness |
| 971 | Kumari, R.; Keleekai-Brapoh, N.; Flood, R.; Audett, J.; Anghel, S.; Gundavarapu, R. Physician Champions: Key to Malnutrition Diagnosis Identification and Documentation...2019 Food & Nutrition Conference & Expo, 26-29 October 2019, Philadelphia, PA. Journal of the Academy of Nutrition & Dietetics ;119(9):S66-S66 | Conference abstract |
| 972 | Kunnuji MO, Robinson RS, Shawar YR, Shiffman J. Variable implementation of sexuality education in three Nigerian states. Studies in family planning. 2017 Dec;48(4):359-76. | Not within a health care setting |
| 973 | Kuo CC, Robb WJ. Critical roles of orthopaedic surgeon leadership in healthcare systems to improve orthopaedic surgical patient safety. Clinical Orthopaedics and Related Research. 2013 Jun;471(6):1792-800. | Study not about effectiveness |
| 974 | Kuo KM, Liu CF, Ma CC. An investigation of the effect of nurses’ technology readiness on the acceptance of mobile electronic medical record systems. BMC medical informatics and decision making. 2013 Dec;13(1):1-4. | Study not about effectiveness |
| 975 | Kuper M, Gold SJ, Callow C, Quraishi T, King S, Mulreany A, Bianchi M, Conway DH. Intraoperative fluid management guided by oesophageal Doppler monitoring. Bmj. 2011 May 24;342. | Study not about effectiveness |
| 976 | Kwakwa, J., Ashforth, H., Pritchard, A.,Trout, R. Training to promote dementia support: NT. Nursing Times 2016;112(25):20 | Study not about effectiveness |
| 977 | Kwan BM, Fernald D, Ferrarone P, Loskutova N, Holtrop JS, Staton EW, Westfall JM. Implementation and evaluation of a laboratory safety process improvement toolkit. The Journal of the American Board of Family Medicine. 2019 Mar 1;32(2):136-45. | Study not about effectiveness |
| 978 | LaBresh KA, Ellrodt AG, Gliklich R, Liljestrand J, Peto R. Get with the guidelines for cardiovascular secondary prevention: pilot results. Archives of Internal Medicine. 2004 Jan 26;164(2):203-9. | Study not about effectiveness |
| 979 | Laibhen-Parkes N. Increasing the practice of questioning among pediatric nurses:“The Growing Culture of Clinical Inquiry” project. Journal of pediatric nursing. 2014 Mar 1;29(2):132-42. | Study not about effectiveness |
| 980 | Laing G, Djebah R, Hoff J, Shaffer R, Harding SR, Moga C, Kletke S, Scott A. OP35 Integrated Knowledge Translation In Policy Development. International Journal of Technology Assessment in Health Care. 2018;34(S1):14-. | Conference abstract |
| 981 | Lalani M, Hall K, Skrypak M, Laing C, Welch J, Toohey P, Seaholme S, Weijburg T, Eyre L, Marshall M. Building motivation to participate in a quality improvement collaborative in NHS hospital trusts in Southeast England: a qualitative participatory evaluation. BMJ open. 2018 Apr 1;8(4):e020930. | Study not about effectiveness |
| 982 | Lam A., Knowles S., Lai S.SNF 2.0: Interact-ing with the unengaged. Journal of the American Geriatrics Society. 2014;62(SUPPL. 1):S133 | Conference abstract |
| 983 | Landry D, Dowd J, Greco B, Sweet S. Effectiveness of a Protocol-Driven Continuous Quality Improvement Initiative on Dialysis-Related Outcomes in Eight Outpatient Hemodialysis Units. Hemodialysis International. 2010 Jan;14(1). | Conference abstract |
| 984 | Lane AJ, Mitchell CG. Using a train-the-trainer model to prepare educators for simulation instruction. The journal of continuing education in nursing. 2013 Jul 1;44(7):313-7. | Study not about effectiveness |
| 985 | Lange JW, Mager D, Greiner PA, Saracino K. The ELDER project: educational model and three-year outcomes of a community-based geriatric education initiative. Gerontology & geriatrics education. 2011 Apr 1;32(2):164-81. | Study not about effectiveness |
| 986 | Langford BJ, Quirk J, Carey S, Daneman N, Garber GE. Influencing duration of antibiotic therapy: A behavior change analysis in long-term care. American journal of infection control. 2019 Dec 1;47(12):1409-14. | Study not about effectiveness |
| 987 | Lanier WL. A three-decade perspective on anesthesia safety. The American Surgeon. 2006 Nov;72(11):985-9. | Letters to the editor/ Review studies |
| 988 | Larson,​ L. (2006). Keeping the relationship alive. Trustee : the journal for hospital governing boards / 2006;59(7):22-1 | Study not about effectiveness |
| 989 | Lau CY, Greysen SR, Mistry RI, Han SJ, Mummaneni PV, Berger MS. Creating a culture of safety within operative neurosurgery: the design and implementation of a perioperative safety video. Neurosurgical focus. 2012 Nov 1;33(5):E3. | Study not about effectiveness |
| 990 | Lau F, Doze S, Vincent D, Wilson D, Noseworthy T, Hayward R, Penn A. Patterns of improvisation for evidence‐based practice in clinical settings. Information Technology & People. 1999 Sep 1. | Study not about effectiveness |
| 991 | Lau F, Penn A, Wilson D, Noseworthy T, Vincent D, Doze S. The diffusion of an evidence-based disease guidance system for managing stroke. International journal of medical informatics. 1998 Aug 1;51(2-3):107-16. | Study not about effectiveness |
| 992 | Lau LS. Leadership and management in quality radiology. Biomedical imaging and intervention journal. 2007 Jul;3(3). | Study not about effectiveness |
| 993 | Laukka E, Huhtakangas M, Heponiemi T, Kanste O. Identifying the roles of healthcare leaders in hit implementation: a scoping review of the quantitative and qualitative evidence. International journal of environmental research and public health. 2020 Jan;17(8):2865. | Letters to the editor/ Review studies |
| 994 | Laur C, Bell J, Valaitis R, Ray S, Keller H. The Sustain and Spread Framework: strategies for sustaining and spreading nutrition care improvements in acute care based on thematic analysis from the More-2-Eat study. BMC health services research. 2018 Dec;18(1):1-1. | Duplicate |
| 995 | Laur C, Bell J, Valaitis R, Ray S, Keller H. The Sustain and Spread Framework: strategies for sustaining and spreading nutrition care improvements in acute care based on thematic analysis from the More-2-Eat study. BMC health services research. 2018 Dec;18(1):1-1. | Study not about effectiveness |
| 996 | Laur C, Butterworth D, Nasser R, Bell J, Marcell C, Murphy J, Valaitis R, Bernier P, Ray S, Keller H. Impact of Facilitated Behavior Change Strategies on Food Intake Monitoring and Body Weight Measurements in Acute Care: Case Examples From the More‐2‐Eat Study. Nutrition in Clinical Practice. 2019 Jun;34(3):459-74. | Study not about effectiveness |
| 997 | Laurenzano RS. Dental consultants champion evidence-based dentistry. AHIP Coverage. 2008 Sep 1;49(5):62-3. | Not about knowledge translation/evidence-based practice |
| 998 | Lavigne-Sims S, Iheagwara K, Mathews R, Walker G, McMahon P. IMPROVING SEDATION LEVELS: A QUALITY IMPROVEMENT PROJECT. Journal of Investigative Medicine. 2019 feb 1 (vol. 67, no. 2, pp. 626-626). | Conference abstract |
| 999 | Lawrence P. Preparing a cultural strategy for PACS. Radiology management. 2005 Jan 1;27(1):21-6. | Study not about effectiveness |
| 1000 | Lawrence, T. P. and D. Thompson (2004). "Physician buy-in helps PI team reduce LOS: data credibility, physician champion key elements." Hospital Case Management 12(6): 89-90. | Study not about effectiveness |
| 1001 | Laytin AD, Azazh A, Girma B, Debebe F, Beza L, Seid H, Landes M, Wytsma J, Reynolds TA. Mixed methods process evaluation of pilot implementation of the African Federation for Emergency Medicine trauma data project protocol in Ethiopia. African journal of emergency medicine. 2019 Jan 1;9:S28-31. | Study not about effectiveness |
| 1002 | Leach M, Etheridge L, Truesdale P. G217 (P) Quality Improvement Project:‘Safe Prescribing’in St Georges Hospital paediatric department. Archives of Disease in Childhood. 2014 Apr 1;99(Suppl 1):A203-. | Conference abstract |
| 1003 | Leach ME, Pasha N, McKinnon K, Etheridge L. Quality improvement project to reduce paediatric prescribing errors in a teaching hospital. Archives of Disease in Childhood-Education and Practice. 2016 Dec 1;101(6):311-5. | Study not about effectiveness |
| 1004 | Leader A, Cadet C, Lazala D, Roa W, Arroyo O, Jensen L. Collaborative implementation strategy for newborn resuscitation and essential care training in the Dominican Republic. Frontiers in public health. 2017 Mar 31;5:61. | Study not about effectiveness |
| 1005 | Leake E, Koopmans E, Sanders C. Primary care providers involvement in caring for young adults with complex chronic conditions exiting pediatric care: An integrative literature review. Comprehensive child and adolescent nursing. 2020 Mar 19:1-22. | Letters to the editor/ Review studies |
| 1006 | Leblanc CM, Chui N, Ambler K, Adamo KB, Spence G, Orrbine E, Cummings E. Practicing what We Preach Part 2: a Follow-Up Look At Healthy Active Living Policy and Practice in Canadian Academic Paediatric Health Centres (Caphc)—Has Anything Changed?. Paediatrics & Child Health. 2010 May 1;15(suppl_A):73A-4A. | Conference abstract |
| 1007 | Lebrun LA, Shi L, Chowdhury J, Sripipatana A, Zhu J, Sharma R, Hayashi AS, Daly CA, Tomoyasu N, Nair S, Ngo-Metzger Q. Primary care and public health activities in select US health centers: documenting successes, barriers, and lessons learned. American journal of preventive medicine. 2012 Jun 1;42(6):S191-202. | Study not about effectiveness |
| 1008 | Lee A, McKee C, Biddle A, Harrison S, Hoffman T, Marcum G, Teske D, Brilli R. A preventable harm index is an effective tool to measure patient safety events in a pediatric car-diothoracic intensive care unit. Critical Care Medicine. 2011 Dec 1;39(12):177. | Conference abstract |
| 1009 | Lee DB, Dell RM. Implementation of a fracture liaison service model of post-fracture osteoporosis care in the us Medicare beneficiary population. Osteoporosis International 2012 Mar 1 (Vol. 23, pp. S264-S265). | Conference abstract |
| 1010 | Lee J, Schack E, Hattensen D, Phongtankuel V, Kozlov E, Adelman RD. The Palliative Care Champions Program: A Survey Study of a Palliative Care Training Curriculum for Social Workers and Nurses. Journal of the American Geriatrics Society. 2019 Apr 1 (Vol. 67, pp. S151-S151). | Conference abstract |
| 1011 | Lee JJ. Improving Smoking Cessation Education Program for Patients with Mental Illness. State University of New York at Binghamton, Decker School of Nursing; 2015. | Study not about effectiveness |
| 1012 | Lee MJ, Berman MR, Flores C, Baron E, Wang J, Matheson D, Lipshutz R, Stein JL. 575: Successful adoption and acceptance of a full-time laborist model in a community-based hospital. American Journal of Obstetrics & Gynecology. 2019 Jan 1;220(1):S382. | Conference abstract |
| 1013 | Lee NK, Cameron J, Harney A, Roeg S. Dual diagnosis capability after an AOD workforce initiative. Advances in Dual Diagnosis. 2011 Nov 17. | Study not about effectiveness |
| 1014 | Lee SJ, Higashi RT, Inrig SJ, Sanders JM, Zhu H, Argenbright KE, Tiro JA. County-level outcomes of a rural breast cancer screening outreach strategy: a decentralized hub-and-spoke model (BSPAN2). Translational behavioral medicine. 2017 Jun 1;7(2):349-57. | Study not about effectiveness |
| 1015 | Lee Y.C., Tay Y.C., Cheong F.W.F. Occupational therapy department's fall prevention initiatives to reduce patient fall incidents. Annals of the Academy of Medicine Singapore. 2014;43(9 SUPPL. 1):S41 | Conference abstract |
| 1016 | Leece P, Timmings C, Moore J, Shantharam Y, Furlan a. Development of an intervention to improve opioid guideline adherence using a behaviour change framework: WIP16–0378. Pain Practice. 2016 May;16. | Conference abstract |
| 1017 | Leeman J, Askelson N, Ko LK, Rohweder CL, Avelis J, Best A, Friedman D, Glanz K, Seegmiller L, Stradtman L, Vanderpool RC. Understanding the processes that Federally Qualified Health Centers use to select and implement colorectal cancer screening interventions: a qualitative study. Translational behavioral medicine. 2020 Apr;10(2):394-403. | Study not about effectiveness |
| 1018 | Lees L, McAuliffe M. Venous thromboembolism risk assessments in acute care. Nursing Standard. 2010 Feb 3;24(22). | Study not about effectiveness |
| 1019 | Lehtonen L, Axelin A. Medical champions can make a difference in initiating culture change. | Study not about effectiveness |
| 1020 | Leigh JA, Long PW, Barraclough BH. The Clinical Support Systems Program: supporting system‐wide improvement. Medical journal of Australia. 2004 May;180:S101-3. | Study not about effectiveness |
| 1021 | LeMaster CH, Hoffart N, Chafe T, Benzer T, Schuur JD. Implementing the central venous catheter infection prevention bundle in the emergency department: experiences among early adopters. Annals of emergency medicine. 2014 Mar 1;63(3):340-50. | Study not about effectiveness |
| 1022 | Lennon MR, Bouamrane MM, Devlin AM, O'connor S, O'donnell C, Chetty U, Agbakoba R, Bikker A, Grieve E, Finch T, Watson N. Readiness for delivering digital health at scale: lessons from a longitudinal qualitative evaluation of a national digital health innovation program in the United Kingdom. Journal of medical Internet research. 2017 Feb 16;19(2):e6900. | Study not about effectiveness |
| 1023 | Leon V, Erbafina MP. Get in the red zone for patient safety. Pediatric Critical Care Medicine. 2014 May 1;15(4_suppl):208. | Conference abstract |
| 1024 | Leong L.A., Mendelsohn M., Saavedra C.J., Morgan R. An interdisciplinary model of collaboration between medical staff (MS), hospital quality improvement (QI), and continuing medical education (CME) departments to promote education and practice changes in the prevention of venous thromboembolism (VTE) in ca. Journal of Clinical Oncology. 2014;32(30 SUPPL. 1). | Conference abstract |
| 1025 | Leong T, Roome K, Miller T, Gorbatkin O, Singleton L, Agarwal M, Lazarus SG. Expansion of a multi-pronged safe sleep quality improvement initiative to three children’s hospital campuses. Injury epidemiology. 2020 Jun;7(1):1-8. | Study not about effectiveness |
| 1026 | Leung V., Lloyd-Smith E., Romney M. Physician hand hygiene champions. Canadian Journal of Infectious Diseases and Medical Microbiology / 2012;23(SUPPL. SB):35B | Conference abstract |
| 1027 | Lewis CC, Boyd M, Puspitasari A, Navarro E, Howard J, Kassab H, Hoffman M, Scott K, Lyon A, Douglas S, Simon G. Implementing measurement-based care in behavioral health: a review. JAMA psychiatry. 2019 Mar 1;76(3):324-35. | Study not about effectiveness |
| 1028 | Lewis M.J., Simmons E. Preserving dignity and self esteem while preventing catheter-associated urinary tract infections. Journal of Spinal Cord Medicine / 2013;36(5 SPEC. ISSUE):555-556 | Conference abstract |
| 1029 | Lewis T, Edwards C. How clinical champions can improve quality. Nursing management. 2008 Mar 1;14(10). | Study not about effectiveness |
| 1030 | Lewis, L.,Hauck, Y. L., PhD, Ashton, E., Engelbrecht, D., Nicolaou, L., Crichton, C., Spence, M. Maternity patients' perceptions of staff compliance with hand hygiene and skin-to-skin contact with a newborn. Evidence Based Midwifery 2016;14(4):125-130 | Study not about effectiveness |
| 1031 | Leyenaar JK, Andrews CB, Tyksinski ER, Biondi E, Parikh K, Ralston S. Facilitators of interdepartmental quality improvement: a mixed-methods analysis of a collaborative to improve pediatric community-acquired pneumonia management. BMJ quality & safety. 2019 Mar 1;28(3):215-22. | Study not about effectiveness |
| 1032 | Li PT. A population-based study on the association of standardized protocols in the emergency department for childhood asthma with outcomes in Ontario, Canada. University of Toronto (Canada); 2010. | Study not about effectiveness |
| 1033 | Li SA, Jeffs L, Barwick M, Stevens B. Organizational contextual features that influence the implementation of evidence-based practices across healthcare settings: a systematic integrative review. Systematic reviews. 2018 Dec;7(1):1-9. | Letters to the editor/ Review studies |
| 1034 | Li T, Chen C, Zhang SS, Dankwa-Mullan I, Chen A, Preininger A, Jackson GP, Liang J. Deployment and integration of a cognitive technology in China: Experiences and lessons learned. | Conference abstract |
| 1035 | Liaw KR. Integrated Family Stress Screening and Response in Pediatric Intensive Care. In64th Annual Meeting 2017 Oct 24. AACAP. | Conference abstract |
| 1036 | Liaw RL. Family Emotional Well-Being and Resiliency in Pediatric Diabetes Care: Early Learning From the Roadmap Pilot Collaborative. In2020 Virtual Meeting 2020 Oct 24. AACAP. | Conference abstract |
| 1037 | Liaw YQ, Goh ML. Improving the accuracy of fluid intake charting through patient involvement in an adult surgical ward: a best practice implementation project. JBI Evidence Synthesis. 2018 Aug 1;16(8):1709-19. | Study not about effectiveness |
| 1038 | Lichtman JH, Roumanis SA, Radford MJ, Riedinger MS, Weingarten S, Krumholz HM. Can practice guidelines be transported effectively to different settings? Results from a multicenter interventional study. The Joint Commission journal on quality improvement. 2001 Jan 1;27(1):42-53. | Study not about effectiveness |
| 1039 | Liddy C, Hogg W, Russell G. Implementation and utilisation of disease registries in primary care. International Journal of Healthcare Technology and Management. 2014 Jan 1;14(4):239-53. | Study not about effectiveness |
| 1040 | Liebhaber A, Draper DA, Cohen GR. Hospital strategies to engage physicians in quality improvement. Issue Brief Cent Stud Health Syst Change. 2009 Oct 1;127:1-4. | Study not about effectiveness |
| 1041 | Liersch S, Tobias T, Brady L. Strengthening the provision of supportive care for adolescents and young adults (Aya's) with cancer in Southern Melbourne: 274. Asia-pacific Journal of Clinical Oncology. 2011 Nov;7. | Conference abstract |
| 1042 | Lieu TA, Herrinton LJ, Buzkov DE, Liu L, Lyons D, Neugebauer R, Needham T, Ng D, Prausnitz S, Stewart K, Van Den Eeden SK. Developing a Prognostic Information System for Personalized Care in Real Time. eGEMs. 2019;7(1). | Study not about effectiveness |
| 1043 | Lim K, Burns M, Hardie J, Rae MC, Pillay K, Ghooloo MF, Roberts P, Rayment M, Finlay S, Sullivan A. HIV testing in the ED is effective and sustainable. HIV Med. 2014 Apr 1;15(Suppl 3):101. | Conference abstract |
| 1044 | Lim ZJ, Nagle D, McAllan F, Ramanan R, Dendle C, Stuart RL, Egerton-Warburton D. Evaluating the sustained effectiveness of a multimodal intervention aimed at influencing PIVC insertion practices in the emergency department. Emergency Medicine Journal. 2020 Jul 1;37(7):444-9. | Not a champion |
| 1045 | Lin F, Marshall AP, Gillespie B, Li Y, O’Callaghan F, Morrissey S, Whitelock K, Morley N, Chaboyer W. Evaluating the Implementation of a Multi‐Component Intervention to Prevent Surgical Site Infection and Promote Evidence‐Based Practice. Worldviews on Evidence‐Based Nursing. 2020 Jun;17(3):193-201. | Study not about effectiveness |
| 1046 | Lin L., Wade C. Comprehensive prevention and management of pressure ulcers in an acute inpatient rehabilitation facility: An evidence ebased assessment. PM and R. 2016;8(9 Supplement):S182-S183 | Conference abstract |
| 1047 | Lin M, Heisler S, Fahey L, McGinnis J, Whiffen TL. Nurse knowledge exchangeplus: Human-centered implementation for spread and sustainability. The Joint Commission Journal on Quality and Patient Safety. 2015 Jul 1;41(7):303-AP5. | Duplicate |
| 1048 | Lin M, Heisler S, Fahey L, McGinnis J, Whiffen TL. Nurse knowledge exchangeplus: Human-centered implementation for spread and sustainability. The Joint Commission Journal on Quality and Patient Safety. 2015 Jul 1;41(7):303-AP5. | Study not about effectiveness |
| 1049 | Lin SY, Lewis FM. Dementia friendly, dementia capable, and dementia positive: concepts to prepare for the future. The Gerontologist. 2015 Apr 1;55(2):237-44. | Not a champion |
| 1050 | Lindberg A. Linking Departmental Priorities to Knowledge Management: The Experiences of Santa Cruz County's Human Services Department. Journal of evidence-based social work. 2012 Feb 29;9(1-2):57-67. | Study not about effectiveness |
| 1051 | Lindenauer PK. 'Modest' benefits seen with use of hospitalists: no significant improvement in patient outcomes. Healthcare Benchmarks & Quality Improvement. 2008;15(3):34-35 | Conference abstract |
| 1052 | Lindley LC, Herr KA, Norton SA. The role of hospice and palliative care nurses in quality improvement. Journal of Hospice & Palliative Nursing. 2017 Apr 1;19(2):160-5. | Study not about effectiveness |
| 1053 | Lindsay HC, Gallaher J, Wright C, Korchinski L, Sing CK. MP23: A collaborative quality improvement initiative to improve the time to electrocardiogram in patients with chest pain presenting to the emergency department. Canadian Journal of Emergency Medicine. 2018 May;20(S1):S48-9. | Conference abstract |
| 1054 | Lindsey G., Hoverman J.R. Development of a quality infrastructure for a 135-site statewide network of community oncologists. Journal of Clinical Oncology / 2012;30(34 SUPPL. 1). | Conference abstract |
| 1055 | Lingren D, Callaway T, Lagrimas A, Owen T, Sweet D, Timmons R, Vermeltfoort J, Daly MA. 106: Successful implementation of icu liberation using the interprofessional team model. Critical Care Medicine. 2013 Dec 1;41(12):A20. | Conference abstract |
| 1056 | Linke CA, Chapman LB, Berger LJ, Kelly TL, Korpela CA, Petty MG. Early mobilization in the ICU: a collaborative, integrated approach. Critical care explorations. 2020 Apr;2(4). | Study not about effectiveness |
| 1057 | Linnan L, Fisher EB, Hood S. The power and potential of peer support in workplace interventions. American journal of health promotion: AJHP. 2013 Sep 1;28(1):TAHP2-10. | Study not about effectiveness |
| 1058 | Lipshutz AK, Fee C, Schell H, Campbell L, Taylor J, Sharpe BA, Nguyen J, Gropper MA. Strategies for success: A PDSA analysis of three QI initiatives in critical care. The Joint Commission Journal on Quality and Patient Safety. 2008 Aug 1;34(8):435-44. | Study not about effectiveness |
| 1059 | Littlewood R, Canfell OJ, Tracey F. Building a Children’s Health Service and System Research Strategy: development and integration in an Australian paediatric healthcare setting. BMC health services research. 2020 Dec;20(1):1-1. | Study not about effectiveness |
| 1060 | Liu H, Laba TL, Massi L, Jan S, Usherwood T, Patel A, Hayman NE, Cass A, Eades AM, Lawrence C, Peiris DP. Facilitators and barriers to implementation of a pragmatic clinical trial in Aboriginal health services. Medical Journal of Australia. 2015 Jul;203(1):24-7. | Study not about effectiveness |
| 1061 | Liu J.-F., Shanmugavadivel D., Wilne S., Walker D.A. Reducing time to diagnosis of paediatric brain tumours in the uk-headsmart awareness campaign. Neuro-Oncology / 2017;19(Supplement 4). | Conference abstract |
| 1062 | Liu SS, Meyerson B, King J, Yih Y, Ostovari M. Drivers and barriers for adopting accreditation at local health departments for their performance improvement effort. Journal of public health management and practice: JPHMP. 2017 Nov;23(6):e25. | Study not about effectiveness |
| 1063 | Liversedge H. An analysis of hospital-acquired skin damage in neonatal units:“You sometimes feel like they haven’t really got any skin” (Doctoral dissertation, University of Southampton). | Study not about effectiveness |
| 1064 | Lloyd-Smith E, Gilbart W, Romney M. P09. 17 Implementation of an infection control champions project in a North American setting: feedback from focus groups. Journal of Hospital Infection. 2010(76):S32. | Conference abstract |
| 1065 | Lloyd-Smith E., Curtin J., Romney M. Infection control champions: A randomized clinical trial. Journal of Hospital Infection / 2010;76(SUPPL. 1):S31-S32 | Conference abstract |
| 1066 | Locatelli, S. M., Sohn, M.-W., Spring, B., Hadi, S.,Weaver, F. M. Participant Retention in the Veterans Health Administration's MOVE! Weight management program, 2010. Preventing chronic disease / 2012;9(101205018):E129 | Study not about effectiveness |
| 1067 | Locock L, Dopson S, Chambers D, Gabbay J. Understanding the role of opinion leaders in improving clinical effectiveness. Social science & medicine. 2001 Sep 1;53(6):745-57. | Letters to the editor/ Review studies |
| 1068 | Loehr J. Creating a Sustainable Vaccine Delivery Practice. Primary Care: Clinics in Office Practice. 2020 Sep 1;47(3):407-18. | Letters to the editor/ Review studies |
| 1069 | Loeppke R, Howell JW. Integrating clinical performance improvement across physician organizations: the PhyCor experience. The Joint Commission journal on quality improvement. 1999 Feb 1;25(2):55-67. | Study not about effectiveness |
| 1070 | Loffredo A, Torbati S, Nuckols T, Robertson V, Geiderman J. 216 A Quality Improvement Intervention That Promotes Goals of Care Discussions Between Emergency Physicians and Patients Near the End of Life. Annals of Emergency Medicine. 2017 Oct 1;70(4):S86-7. | Conference abstract |
| 1071 | Löfgren S, Hansson J, Øvretveit J, Brommels M. Context challenges the champion: improving hip fracture care in a Swedish university hospital. International journal of health care quality assurance. 2012 Feb 3. | Study not about effectiveness |
| 1072 | Logan AY, Williamson JE, Reinke EK, Jarrett SW, Boger MS, Davidson LE. Establishing an antimicrobial stewardship collaborative across a large, diverse health care system. The Joint Commission Journal on Quality and Patient Safety. 2019 Sep 1;45(9):591-9. | Study not about effectiveness |
| 1073 | Loh KP, Kaushik R, Mohile SG, Ogie S, Kadambi S, Zittel J, Yousefi-Nooraie R, Moorthi K, Patil A, Sanapala C, Yang S. Speeding the dissemination and implementation of geriatric assessment: What we can learn from the business world. Journal of geriatric oncology. 2020 Sep 1;11(7):1170-4. | Conference abstract |
| 1074 | Lollier A, Rodriguez EM, Saad-Harfouche FG, Widman CA, Mahoney MC. HPV vaccination: pilot study assessing characteristics of high and low performing primary care offices. Preventive medicine reports. 2018 Jun 1;10:157-61. | Study not about effectiveness |
| 1075 | Lomas J. Evidence-based practice in Steeltown: a good start on needed cultural change. Healthcarepapers. 2003 Jan 1;3(3):24-8. | Letters to the editor/ Review studies |
| 1076 | Lomas J. Retailing research: increasing the role of evidence in clinical services for childbirth. The Milbank Quarterly. 1993 Jan 1:439-75. | Study not about effectiveness |
| 1077 | Long, L., Lucki, M. M., Polonia, D., Fada, R., Hartwell, J. L., Gascon, G.M. Reducing Rates of Perioperative Deep Vein Thrombosis and Pulmonary Emboli in Hip and Knee Arthroplasty Patients: A Quality Improvement Project. Journal of Clinical Outcomes Management.2019;26(1):19-25 | Study not about effectiveness |
| 1078 | Longman JM, Adams CM, Johnston JJ, Passey ME. Improving implementation of the smoking cessation guidelines with pregnant women: how to support clinicians?. Midwifery. 2018 Mar 1;58:137-44. | Not a champion |
| 1079 | Looney JA, Shaw EK, Crabtree BF. Passing the Baton: Sustaining Organizational Change After the Facilitator Leaves. Group Facilitation: A Research & Applications Journal. 2011 Sep 1;11. | Study not about effectiveness |
| 1080 | Lorenzi NM, Kouroubali A, Detmer DE, Bloomrosen M. How to successfully select and implement electronic health records (EHR) in small ambulatory practice settings. BMC medical informatics and decision making. 2009 Dec;9(1):1-3. | Study not about effectiveness |
| 1081 | Louie E, Giannopoulos V, Baillie A, Uribe G, Byrne S, Deady M, Teesson M, Baker A, Haber PS, Morley KC. Translating evidence-based practice for managing comorbid substance use and mental illness using a multimodal training package. Journal of dual diagnosis. 2018 Apr 3;14(2):111-9. | Study not about effectiveness |
| 1082 | Louise C, Gregus A. Accreditation canada stroke distinction program: a global program for improving quality of stroke services. International Journal of Stroke. 2018.13: 95-95. | Conference abstract |
| 1083 | Lovell M., Phillips J., Agar M., Luckett T., Carrow D., Boyle F., Davidson P., Lam L., McCaffrey N., Shaw T., Read A. Strategies to improve routine pain screening in Australian outpatient oncology and palliative care clinics: process data from the stop cancer pain trial. Supportive Care in Cancer / 2018;26(2 Supplement 1):S101 | Conference abstract |
| 1084 | Lövquist E, Shorten G, Aboulafia A. Virtual reality-based medical training and assessment: The multidisciplinary relationship between clinicians, educators and developers. Medical Teacher. 2012 Jan 1;34(1):59-64. | Study not about effectiveness |
| 1085 | Low LF, Baker JR, Jeon YH, Camp C, Haertsch M, Skropeta M. Study protocol: translating and implementing psychosocial interventions in aged home care the lifestyle engagement activity program (LEAP) for life. BMC geriatrics. 2013 Dec;13(1):1-2. | Study not about effectiveness |
| 1086 | Low LF, Fletcher J, Goodenough B, Jeon YH, Etherton-Beer C, MacAndrew M, Beattie E. A systematic review of interventions to change staff care practices in order to improve resident outcomes in nursing homes. PloS one. 2015 Nov 11;10(11):e0140711. | Letters to the editor/ Review studies |
| 1087 | Lowenhoff C, Davison‐Fischer J, Pike N, Appleton JV. Using the TIDieR checklist to describe health visitor support for mothers with mental health problems: Analysis of a cross‐sectional survey. Health & social care in the community. 2019 Sep;27(5):e824-36. | Study not about effectiveness |
| 1088 | Lowenthal G, Dkyes PC, Lipsitz SR, Yoon C. Promoting proper utilization of early detection technology in the acute care setting. Journal of General Internal Medicine. 2015.30: S236-S237. | Conference abstract |
| 1089 | Lu T, Pink J, Whitten L, Hill C, Adams R, Gibb C. A champion-driven pathway towards quality improvement in the medical management of osteoporotic fractures. | Study not about effectiveness |
| 1090 | Lucas E, Bevan A. Nurses’ underuse of non-pharmacological pain relief interventions in preterm infants. Nursing children and young people. 2020 Jan 9;32(1). | Study not about effectiveness |
| 1091 | Lucas E, Bevan A. Nurses’ underuse of non-pharmacological pain relief interventions in preterm infants. Nursing children and young people. 2020 Jan 9;32(1). | Study not about effectiveness |
| 1092 | Luckett T, Phillips J, Agar M, Lam L, Davidson PM, McCaffrey N, Boyle F, Shaw T, Currow DC, Read A, Hosie A. Protocol for a phase III pragmatic stepped wedge cluster randomised controlled trial comparing the effectiveness and cost-effectiveness of screening and guidelines with, versus without, implementation strategies for improving pain in adults with cancer attending outpatient oncology and palliative care services: the Stop Cancer PAIN trial. BMC health services research. 2018 Dec;18(1):1-3. | Protocol |
| 1093 | Lum H, Perez A, Saxinger C, Parnes B. Improving advance care planning through team-based quality improvement initiative in a geriatrics clinic: B154. Journal of the American Geriatrics Society. 2016 May;64. | Conference abstract |
| 1094 | Lustig A, Ogden M, Brenner RW, Penso J, Westrich KD, Dubois RW. The central role of physician leadership for driving change in value-based care environments. Journal of managed care & specialty pharmacy. 2016 Oct;22(10):1116-22. | Study not about effectiveness |
| 1095 | Luther V, Hammersley D, Chekairi A. Improving patient handover between teams using a business improvement model: PDSA cycle. British Journal of Hospital Medicine. 2014 Jan;75(1):44-7. | Study not about effectiveness |
| 1096 | Luton MN,Stewart MS, Steward-Scott MS, Mullen MS, CCRN-K J, Jones MN, Hagan ScD J. Evidence-based skin champion program reduces pressure injuries in a pediatric hospital. Journal of Nursing & Interprofessional Leadership in Quality & Safety. 2018;2(1):2. | Study not about effectiveness |
| 1097 | Luz S, Shadmi E, Admi H, Peterfreund I, Drach‐Zahavy A. Characteristics and behaviours of formal versus informal nurse champions and their relationship to innovation success. Journal of advanced nursing. 2019 Jan;75(1):85-95. | Study not about effectiveness |
| 1098 | Luz S, Shadmi E, Drach-Zahavy A. Nursing innovation: The joint effects of championship behaviors, project types, and initiation levels. Nursing outlook. 2019 Jul 1;67(4):404-18. | Study not about effectiveness |
| 1099 | Lydtin A, Dale S, Longworth M, Hill K, Katalinic E, Tinsley M, Comerford D, Ryan F, Quinn C, Patterson S, Grimshaw J. The QASC Implementation Project: Upscale and spread of evidence based results across NSW. International Journal of Stroke. 2014 Aug;9:9-10. | Conference abstract |
| 1100 | Lyssa Friedman RN, Constance Engelking RN, Catherine Harvey RN, Martha Read RN. The EDUCATE study: a continuing education exemplar for clinical practice guideline implementation. Clinical Journal of Oncology Nursing. 2009 Apr 1;13(2):219. | Study not about effectiveness |
| 1101 | Mabolo M, Brown D, Futrell J, Sherer A, Morrison T. Caution! CAUTI-Free Zone: Improving Outcomes in Heart Failure Patients. Heart & Lung: The Journal of Cardiopulmonary and Acute Care. 2014 Jul 1;43(4):380. | Conference abstract |
| 1102 | MacDonell R, Koe S, Okafor I, Adam J, Ahern C, Scanlan T. P255 Building resuscitation capability in low resource settings; development of the PAIRS course (Outlining the development of a tailored paediatric resuscitation programme for low resource healthcare settings). Archives of Disease in Childhood. 2019 Jun 1;104(Suppl 3):A259. | Conference abstract |
| 1103 | Macdonell R, Lambert V, Fitzsimons J, Horkan S. Isqua17-3305 developing and implementing a national paediatric early warning system for managing child clinical deterioration. International Journal for Quality in Health Care. 2017 Sep 1;29(suppl_1):36-7. | Conference abstract |
| 1104 | MacDonell R, Scanlan T, Koe S, Okafor I, Ahern C, James A. OC37 Challenges and successes of implementing a paediatric early warning system in a limited resource paediatric oncology setting. | Conference abstract |
| 1105 | MacEachin SR, Lopez CM, Powell KJ, Corbett NL. The fetal heart rate collaborative practice project: situational awareness in electronic fetal monitoring—a Kaiser Permanente perinatal patient safety program initiative. The Journal of perinatal & neonatal nursing. 2009 Oct 1;23(4):314-23. | Study not about effectiveness |
| 1106 | MacIntosh‐Murray A, Choo CW. Information behavior in the context of improving patient safety. Journal of the American Society for Information Science and Technology. 2005 Oct;56(12):1332-45 | Study not about effectiveness |
| 1107 | MacIntosh-Murray A. Information behaviour of health care providers for improving patient safety. University of Toronto; 2003. | Study not about effectiveness |
| 1108 | MacKay D, Kirkham R, Freeman N, Murtha K, Van Dokkum P, Boyle J, Campbell S, Barzi F, Connors C, O’Dea K, Oats J. Improving systems of care during and after a pregnancy complicated by hyperglycaemia: A protocol for a complex health systems intervention. BMC health services research. 2020 Dec;20(1):1-7. | Protocol |
| 1109 | MacKean G, Noseworthy T, Elshaug AG, Leggett L, Littlejohns P, Berezanski J, Clement F. Health technology reassessment: the art of the possible. International journal of technology assessment in health care. 2013 Oct;29(4):418-23. | Study not about effectiveness |
| 1110 | Mackereth PA, Stringer J. CAM and cancer care: champions for integration. Complementary therapies in clinical practice. 2005 Feb 1;11(1):45-7. | Study not about effectiveness |
| 1111 | Mackridge AJ, Krska J, Stokes EC, Heim D. Towards improving service delivery in screening and intervention services in community pharmacies: a case study of an alcohol IBA service. Journal of Public Health. 2016 Mar 1;38(1):92-8. | Study not about effectiveness |
| 1112 | Maddox J, Roberts A. Reduction in hospital acquired VTE by improved compliance with VTE risk assessment: P-MO-440. Journal of Thrombosis and Haemostasis. 2011 Jul;9. | Conference abstract |
| 1113 | Mafi J, Godoy-Travieso P, Wei E, Berry J, Amaya R, Wong B, Carillo C, Sarff L, Daskivich L, Vangala S, Keeler E. Evaluation of a Choosing Wisely™ Intervention to Reduce Low Value Preoperative Care for Patients Undergoing Cataract Surgery at a Safety Net Health System. In2017 Annual Research Meeting 2017 Jun 26. AcademyHealth. | Conference abstract |
| 1114 | Mahabee-Gittens EM, Dixon CA, Vaughn LM, Duma EM, Gordon JS. Parental tobacco screening and counseling in the pediatric emergency department: practitioners’ attitudes, perceived barriers, and suggestions for implementation and maintenance. Journal of Emergency Nursing. 2014 Jul 1;40(4):336-45. | Study not about effectiveness |
| 1115 | Mair A, McIntosh J, Codina C, Molist N, Alonso A. Polypharmacy and Adherence: Key Components of Integrated Care Findings from the SIMPATHY Project. International Journal of Integrated Care (IJIC). 2016 Dec 2;16(6). | Conference abstract |
| 1116 | Maletis GB, Funahashi TT, Love R, Wyatt R, Prentice HA, Burfeind W, Paxton L. Demonstrating the Value of an ACL Registry. Orthopaedic Journal of Sports Medicine. 2018 Jul 30;6(7_suppl4):2325967118S00103. | Conference abstract |
| 1117 | Malone K, Clark S, Palmer JA, Lopez S, Pradhan M, Furth S, Kim J, Fisher B, Laskin B. A quality improvement initiative to increase pneumococcal vaccination coverage among children after kidney transplant. Pediatric transplantation. 2016 Sep;20(6):783-9. | Study not about effectiveness |
| 1118 | Malone M.L. Want to Champion Better Care for Vulnerable Older Americans? Start Here. Journal of the American Geriatrics Society. 2018;66(5):847-849 | Letters to the editor/ Review studies |
| 1119 | Mancini MA, Miner CS. Learning and change in a community mental health setting. Journal of Evidence-Based Social Work. 2013 Oct 1;10(5):494-504. | Study not about effectiveness |
| 1120 | Mandapakala C, Levine DL. Walking the talk to improve error reporting. Journal of general internal medicine 2014 Apr 1 (Vol. 29, pp. S249-S249). | Conference abstract |
| 1121 | Mandavia R, Hannink G, Ahmed MN, Premakumar Y, Chu TS, Blackshaw H, Ferdous T, Mehta N, Manjaly J, Khan M, Schilder AG. Prognostic factors for outcomes of idiopathic sudden sensorineural hearing loss: protocol for the SeaSHeL national prospective cohort study. BMJ open. 2020 Sep 1;10(9):e038552. | Study not about effectiveness |
| 1122 | Manheim S, Li QH, Ekpo E. Improving Advance Directive Completion Rates for Patients Followed in the Melanoma Clinic Using the Advancing Research and Clinical Practice Through Close Collaboration Model (ARCC). Nursing Research. 2016.65(2): E32-E33. | Conference abstract |
| 1123 | Manias E, Williams A. Communication between patients with chronic kidney disease and nurses about managing pain in the acute hospital setting. Journal of clinical nursing. 2007 Nov;16(11c):358-67. | Study not about effectiveness |
| 1124 | Manning MW, Bean EW, Miller AC, Templer SJ, Mackenzie RS, Richardson DM, Bresnan KA, Greenberg MR. Using medical student quality improvement projects to promote evidence-based care in the emergency department. Western Journal of Emergency Medicine. 2018 Jan;19(1):148. | Study not about effectiveness |
| 1125 | Marciel KK, Kimberg C, Riekert KA, Swenson A, Quittner AL. Participation of multidisciplinary team members in a behavioral intervention to improve adherence in adolescents with CF. Pediatric Pulmonology.2010. 437-437. | Conference abstract |
| 1126 | Marealle S.B., Jorgensen J. Technology innovation in mentoring and supervision of community health workers in Zanzibar. BMC Proceedings / 2017;11(6 Supplement 1). | Conference abstract |
| 1127 | Maria-Alexandra P, Malherbe S. Environmentally sustainable perioperative medicine: simple strategies for anesthetic practice. Canadian Journal of Anesthesia. 2020 Aug 1;67(8):1044-63. | Study not about effectiveness |
| 1128 | Marino J, Bucher D, Beach M, Yegneswaran B, Cooper B. Implementation of an intensive care unit delirium protocol: an interdisciplinary quality improvement project. Dimensions of Critical Care Nursing. 2015 Sep 1;34(5):273-84. | Study not about effectiveness |
| 1129 | Markham SK, Aiman-Smith L. Product champions: Truths, myths and management. Research-Technology Management. 2001 May 1;44(3):44-50. | Not within a health care setting |
| 1130 | Markham SK, Green SG, Basu R. Champions and antagonists: Relationships with R&D project characteristics and management. Journal of Engineering and Technology Management. 1991 Dec 1;8(3-4):217-42. | Not within a health care setting |
| 1131 | Marlowe F. Dementia care in radiography and the role of the dementia champion. Imaging & Therapy Practice. 2014 Aug 1:13. | Study not about effectiveness |
| 1132 | Marsden E, Taylor A, Wallis M, Craswell A, Broadbent M, Barnett A, Crilly J. Effect of the Geriatric Emergency Department Intervention on outcomes of care for residents of aged care facilities: A non‐randomised trial. Emergency Medicine Australasia. 2020 Jun 1;32(3):422-9. | Study not about effectiveness |
| 1133 | Marsh D, Åkesson K, Beaton DE, Bogoch ER, Boonen S, Brandi ML, McLellan AR, Mitchell PJ, Sale JE, Wahl DA. Coordinator-based systems for secondary prevention in fragility fracture patients. Osteoporosis international. 2011 Jul;22(7):2051-65. | Study not about effectiveness |
| 1134 | Marshall AP, Cahill NE, Gramlich L, MacDonald G, Alberda C, Heyland DK. Optimizing nutrition in intensive care units: empowering critical care nurses to be effective agents of change. American journal of critical care. 2012 May;21(3):186-94. | Study not about effectiveness |
| 1135 | Marshall S, Spencer L, Swindells L, Wainwright J, Power M, Tyrrell P. A quality improvement project to maintain quality stroke care using a stroke champion approach: 3 Acute stroke: clinical patterns and practice including nursing. Cerebrovascular Diseases. 2009;27. | Conference abstract |
| 1136 | Marsteller JA, Woodward P, Underwood WS, Hsiao CJ, Barr MS. Design of a quality and performance improvement project for small primary care practices: reflections on the Center for Practice Innovation. Quality in primary care. 2011 Jan 1;19(1):49-57. | Study not about effectiveness |
| 1137 | Martin A, Darwin L, Thomas N, Owen H, Conway D, Atkinson D, Sharman M, Moore J. ERAS+ reduces pulmonary morbidity after major surgery: 117. Anaesthesia. 2016 Jan;71. | Conference abstract |
| 1138 | Martin GR, Bradshaw EA, Becker J, Cuzzi S, Kiernan S, Nagel N. Implementing pulse oximetry screening for critical congenital heart disease in a community nursery. Journal of the American College of Cardiology. 2010 Mar 9;55(10S):A45-E431. | Conference abstract |
| 1139 | Martindale T., Mathieu S. A quality improvement intervention to reduce noise on the ICU. Journal of the Intensive Care Society / 2016;17(4 Supplement 1):69-70 | Conference abstract |
| 1140 | Martinez C, Bacigalupe G, Cortada JM, Grandes G, Sanchez A, Pombo H, Bully P. The implementation of health promotion in primary and community care: a qualitative analysis of the ‘Prescribe Vida Saludable’strategy. BMC family practice. 2017 Dec;18(1):1-3. | Study not about effectiveness |
| 1141 | Martinez C, Bacigalupe G, Cortada JM, Grandes G, Sanchez A, Pombo H, Bully P. The implementation of health promotion in primary and community care: a qualitative analysis of the ‘Prescribe Vida Saludable’strategy. BMC family practice. 2017 Dec;18(1):1-3. | Study not about effectiveness |
| 1142 | Martinsons MG. Cultivating the champions for strategic information systems. Journal of Systems Management. 1993 Aug 1;44(8):31. | Not within a health care setting |
| 1143 | Masica A, Collinsworth A, Kouznetsova M, Berryman C, Smith S, Lopes S. 810: Evaluating the effectiveness of an abcde bundle implementation progam. Critical Care Medicine. 2014 Dec 1;42(12):A1555. | Conference abstract |
| 1144 | Mathews SC, Gent P, Palmer A, Outten K, Pronovost PJ, Shin EJ, Lee LA. 743 Implementing a Comprehensive Unit-Based Safety Program (CUSP) for Endoscopy Units: A Model for Improving Quality and Safety. Gastroenterology. 2016 Apr 1;150(4):S152. | Conference abstract |
| 1145 | Mathieson AE. A qualitative case study of the implementation and adoption of an evidence-based intervention used by informal carers and community-based care providers. The University of Manchester (United Kingdom); 2019. | Study not about effectiveness |
| 1146 | Mattheos N, Stefanovic N, Apse P, Attstrom R, Buchanan J, Brown P, Camilleri A, Care R, Fabrikant E, Gundersen S, Honkala S. Potential of information technology in dental education. European Journal of Dental Education. 2008 Feb;12:85-92. | Study not about effectiveness |
| 1147 | Mauermann WJ, Nemergut EC, Warltier DC. The anesthesiologist's role in the prevention of surgical site infections. The Journal of the American Society of Anesthesiologists. 2006 Aug 1;105(2):413-21. | Study not about effectiveness |
| 1148 | Maunder K, Walton K, Williams P, Ferguson M, Beck E. Strategic leadership will be essential for dietitian eHealth readiness: A qualitative study exploring dietitian perspectives of eHealth readiness. Nutrition & Dietetics. 2019 Sep;76(4):373-81. | Study not about effectiveness |
| 1149 | Maureen, B. Reduction of Healthcare-Associated Infections (HAIs) by 83% in a long-term acute care setting (LTAC). American Journal of Infection Control. 2019;47: S52. | Conference abstract |
| 1150 | May J, Cooper R, Magin P, Critchley A. Integrated models or mayhem? Lessons learnt from three integrated primary health care entities in regional New South Wales. Australian Health Review. 2008;32(4):595-604. | Study not about effectiveness |
| 1151 | May, R. ‘Matrons undoubtedly improve the quality of patient care’.Nursing Times 2020;116(2):13 | Study not about effectiveness |
| 1152 | Mayer CM, Cluff L, Lin WT, Willis TS, Stafford RE, Williams C, Saunders R, Short KA, Lenfestey N, Kane HL, Amoozegar JB. Evaluating efforts to optimize TeamSTEPPS implementation in surgical and pediatric intensive care units. The Joint Commission journal on quality and patient safety. 2011 Aug 1;37(8):365-AP3. | Study not about effectiveness |
| 1153 | Mayrhofer A, Goodman C, Holman C. Establishing a community of practice for dementia champions (innovative practice). Dementia. 2015 Mar;14(2):259-66. | Study not about effectiveness |
| 1154 | Mazzoni SE, Brewer SE, Pyrzanowski JL, Durfee MJ, Dickinson LM, Barnard JG, Dempsey AF, O’Leary ST. Effect of a multi-modal intervention on immunization rates in obstetrics and gynecology clinics. American journal of obstetrics and gynecology. 2016 May 1;214(5):617-e1. | Study not about effectiveness |
| 1155 | McAlearney AS, Hefner J, Robbins J, Garman AN. The role of leadership in eliminating health care-associated infections: a qualitative study of eight hospitals. InLeading in health care organizations: Improving safety, satisfaction and financial performance 2013 Jul 31. Emerald Group Publishing Limited. | Study not about effectiveness |
| 1156 | McAlearney AS, Reiter KL, Weiner BJ, Minasian L, Song PH. Challenges and facilitators of community clinical oncology program participation: a qualitative study. Journal of Healthcare Management/American College of Healthcare Executives. 2013 Jan;58(1):29. | Study not about effectiveness |
| 1157 | McAlearney, AS, Reiter, KL, Weiner, BJ, Minasian, L, Song, PH. Challenges and facilitators of community clinical oncology program participation: a qualitative study. Journal of healthcare management. 2013;58(1):29-6 | Duplicate |
| 1158 | McBeth C, Curry M, McGlynn G, Shepard J, Hoze M, Nguyen H, Gross E. Improving Severe Sepsis Order Set Compliance in the Emergency Department: 511. Academic Emergency Medicine. 2016 May;23. | Conference abstract |
| 1159 | McBride D, Dohan D, Handley MA, Powe NR, Tuot DS. Developing a CKD registry in primary care: provider attitudes and input. American journal of kidney diseases. 2014 Apr 1;63(4):577-83. | Study not about effectiveness |
| 1160 | McCabe MP, Karantzas GC, Mrkic D, Mellor D, Davison TE. A randomized control trial to evaluate the beyondblue depression training program: does it lead to better recognition of depression?. International journal of geriatric psychiatry. 2013 Mar;28(3):221-6. | Study not about effectiveness |
| 1161 | McCarthy P, Bethune C, Fitzgerald S, Graham W, Asghari S, Heeley T, Godwin M. Curriculum development of 6for6: Longitudinal research skills program for rural and remote family physicians. Canadian Family Physician. 2016 Feb 1;62(2):e89-95. | Study not about effectiveness |
| 1162 | McCaul M, Hendricks L, Naidoo R. Prehospital providers’ perspectives for clinical practice guideline implementation and dissemination: strengthening guideline uptake in South Africa. PloS one. 2019 Jul 22;14(7):e0219761. | Study not about effectiveness |
| 1163 | McCommons R, Wheeler M, Houston S. Colonoscopy Comfort. Gastroenterology Nursing. 2016 May 1;39(3):212-5. | Study not about effectiveness |
| 1164 | McCormick T, Cavalier S, Padua E, Olson L, Ely M, Schmul P, Telford R, Haukoos J, Gausche-Hill M. 191 Evaluation of Interventions to Improve Pediatric Readiness in Community Emergency Departments: A Mixed Methods Study. Annals of Emergency Medicine. 2017 Oct 1;70(4):S77. | Conference abstract |
| 1165 | McCready RA. A Surgeon’s Perspective on the Role of Surgical Champions in the American College of Surgeons National Surgical Quality Improvement Program. Journal of Surgical Research. 2011 Dec 1;171(2):459-60. | Letters to the editor/ Review studies |
| 1166 | McCrum C, Dick K. NICE guidance and quality standard assurance evaluation: a process redesign to improve effectiveness and efficiency. Physiotherapy. 2020 May 1;107:e214-5. | Conference abstract |
| 1167 | McCuistion MH, Stults CD, Dohan D, Frosch DL, Hung DY, Tai-Seale M. Overcoming challenges to adoption of shared medical appointments. Population health management. 2014 Apr 1;17(2):100-5. | Study not about effectiveness |
| 1168 | McCullough MB, Gillespie C, Petrakis BA, Jones EA, Park AM, Lukas CV, Rose AJ. Forming and activating an internal facilitation group for successful implementation: A qualitative study. Research in Social and Administrative Pharmacy. 2017 Sep 1;13(5):1014-27. | Not a champion |
| 1169 | McDaniel CE, Jeske M, Sampayo EM, Liu P, Walls TA, Kaiser SV. Implementing Pediatric Asthma Pathways in Community Hospitals: A National Qualitative Study. Journal of hospital medicine. 2019 Sep 18;15(1):35-41. | Study not about effectiveness |
| 1170 | McDougall T. Child and adolescent mental health services in the UK: nurse consultants. Journal of Child and Adolescent Psychiatric Nursing. 2005 Apr;18(2):79-83. | Study not about effectiveness |
| 1171 | McErlean M. All healthcare staff should become exercise champions. BMJ. 2019 Oct 16;367. | Letters to the editor/ Review studies |
| 1172 | McEvoy R, Tierney E, MacFarlane A. ‘Participation is integral’: understanding the levers and barriers to the implementation of community participation in primary healthcare: a qualitative study using normalisation process theory. BMC health services research. 2019 Dec;19(1):1-4. | Not a champion |
| 1173 | McGivern G, Nzinga J, English M. ‘Pastoral practices’ for quality improvement in a Kenyan clinical network. Social science & medicine. 2017 Dec 1;195:115-22. | Study not about effectiveness |
| 1174 | McGrath BA, Lynch SJ, Bovento B,; Sharpe G, Grainger E, Pieri-Davies S, Wallace S. Improvements in the safety and quality of care in four UK NHS hospitals participating in the global tracheostomy collaborative. Intensive Care Medicine Experimental .2016;4(Supplement 1). | Conference abstract |
| 1175 | McGrath D. How to motivate physicians and develop a physician champion. The Journal of medical practice management: MPM. 2006 Jan 1:13-6. | Study not about effectiveness |
| 1176 | McGraw K, Gelso B, Barry D, Bechowski MS, Tate K, Houston J. The DoD practice-based implementation (PBI) network: Estimating return on investment. Theoretical Issues in Ergonomics Science. 2019 Jan 2;20(1):51-65. | Study not about effectiveness |
| 1177 | McGuire AB, Salyers MP, White DA, Gilbride DJ, White LM, Kean J, Kukla M. Factors affecting implementation of an evidence-based practice in the Veterans Health Administration: Illness management and recovery. Psychiatric Rehabilitation Journal. 2015 Dec;38(4):300. | Study not about effectiveness |
| 1178 | McHugh F, Robertson S, Pryde K, Williams S. Engaging junior paediatric trainees in patient safety improvement projects. Archives of Disease in Childhood. 2012 May 1;97(Suppl 1):A174-. | Conference abstract |
| 1179 | McInnes E, Phillips R, Middleton S, Gould D. A qualitative study of senior hospital managers’ views on current and innovative strategies to improve hand hygiene. BMC infectious diseases. 2014 Dec;14(1):1-2. | Study not about effectiveness |
| 1180 | McIntosh J, Tolson D. Leadership as part of the nurse consultant role: banging the drum for patient care. Journal of Clinical Nursing. 2009 Jan;18(2):219-27. | Study not about effectiveness |
| 1181 | Mckew, M.General practice nurses to train as digital champions.Primary Health Care. 2018;28(6):6-6 | Letters to the editor/ Review studies |
| 1182 | Mckew, M.General practice nurses to train as digital champions.Primary Health Care. 2018;28(6):6-6 | Letters to the editor/ Review studies |
| 1183 | McKinley L, Phillips L, Stampfli C, Matteson K, Crnich C. 463. Healthcare Workers Perceptions Regarding the Use of an Electronic Hand Hygiene Monitoring System at a VA Hospital. InOpen Forum Infectious Diseases 2018 Nov (Vol. 5, No. Suppl 1, p. S174). Oxford University Press. | Conference abstract |
| 1184 | McKinney MM, Warnecke RB, Kaluzny AD. Strategic approaches to cancer control research in NCI-funded research bases. Cancer detection and prevention. 1992 Jan 1;16(5-6):329-35. | Study not about effectiveness |
| 1185 | McLane P, Scott K, Suleman Z, Deol J, Fanaeian J, Olmstead A, Ross M, Hair H, Holroyd B, Lang E, Biggs C. MP30: implementing buprenorphine/naloxone in emergency departments for opioid agonist treatment: a quality improvement initiative. Canadian Journal of Emergency Medicine. 2019 May;21(S1):S53-. | Conference abstract |
| 1186 | McLean HS, Carriker C, Bordley WC. Good to great: quality-improvement initiative increases and sustains pediatric health care worker hand hygiene compliance. Hospital pediatrics. 2017 Apr 1;7(4):189-96. | Study not about effectiveness |
| 1187 | McLellan L, Grin S. Harnessing Nursing Expertise and LEAN Methodology to Champion Change to Arterial Blood Pressure Monitoring. Canadian Journal of Critical Care Nursing. 2016 Jun 1;27(2). | Conference abstract |
| 1188 | McLeod RS, Aarts MA, Chung F, Eskicioglu C, Forbes SS, Conn LG, McCluskey S, McKenzie M, Morningstar B, Nadler A, Okrainec A. Development of an enhanced recovery after surgery guideline and implementation strategy based on the knowledge-to-action cycle. Annals of surgery. 2015 Dec 1;262(6):1016-25. | Study not about effectiveness |
| 1189 | McLeod RS, Aarts MA, Chung F, Eskicioglu C, Forbes SS, Conn LG, McCluskey S, McKenzie M, Morningstar B, Nadler A, Okrainec A. Development of an enhanced recovery after surgery guideline and implementation strategy based on the knowledge-to-action cycle. Annals of surgery. 2015 Dec 1;262(6):1016-25. | Duplicate |
| 1190 | McMahon D. Improving clinical care with unit based education champions. Journal of Cancer Education / 2009;24(SUPPL. 1):57 | Conference abstract |
| 1191 | McManus J, Salinas J, Morton M, Lappan C, Poropatich R. Teleconsultation program for deployed soldiers and healthcare professionals in remote and austere environments. Prehospital and disaster medicine. 2008 Jun;23(3):210-6. | Study not about effectiveness |
| 1192 | McNab D, McKay J, Bowie P. A before and after study of warfarin monitoring in a single region as part of the Scottish patient safety programme in primary care. Scottish medical journal. 2015 Nov;60(4):196-201. | Study not about effectiveness |
| 1193 | McNally M, Martin-Misener R, McNeil K, Brillant M, Moorhouse P, Crowell S, Matthews D, Clovis J. Implementing oral care practices and policy into long-term care: the brushing up on mouth care project. Journal of the American Medical Directors Association. 2015 Mar 1;16(3):200-7. | Study not about effectiveness |
| 1194 | McNees P, Kueven JA. The bottom line on wound care standardization: at North Mississippi Medical Center, standardization of wound care processes and products led to improved healing rates and a $300,000 annual reduction in supply expenses. Healthcare Financial Management. 2011 Mar 1;65(3):70-6. | Study not about effectiveness |
| 1195 | McNeill MM, Archer S, Remsburg D, Storer J, Rudman H. Rapid Response Team-Quality Champion Registered Nurse: Observations and Perceptions. Journal of nursing care quality. 2019 Oct 1;34(4):325-9. | Not about knowledge translation/evidence-based practice |
| 1196 | McNulty CA, Freeman E, Oliver I, Ford-Young W, Randall S. Strategies used to increase chlamydia screening in general practice: a qualitative study. Public Health. 2008 Sep 1;122(9):845-56. | Study not about effectiveness |
| 1197 | McPherson J. Healthcare waste management for hospitals in resource-constrained settings: What determines effective implementation?. | Study not about effectiveness |
| 1198 | McVeigh J, Davidson P. Nurse Practitioners in Cardiac Care: State of the Nation. Heart, Lung and Circulation. 2011 Jan 1;20:S231-2. | Conference abstract |
| 1199 | Mead C, Gilbert P, Husbands S, O'Brien J, Matthews R,Mead C, Gilbert P, Husbands S, O'Brien J, Matthews R. Diabetes Improvement through Mentoring and Peer-led Education (DIMPLE). Diabetic Medicine. 2012;29(SUPPL. 1):114 | Conference abstract |
| 1200 | Mechanick JI, Camacho PM, Garber AJ, Garber JR, Pessah-Pollack R, Petak SM, Tangpricha V, Trence DL. American association of clinical endocrinologists and american college of endocrinology protocol for standardized production of clinical practice guidelines, algorithms, and checklists-2014 update and the AACe G4G program. Endocrine Practice. 2014 Jul 1;20(7):692-702. | Study not about effectiveness |
| 1201 | Megalaa R, Bevilacqua R, Garcha S, Downey A, Chadha A, Schellhase DE. Increasing influenza vaccination rates in cystic fibrosis utilizing a conversation starter: a quality improvement project. Pediatric pulmonology. 2019; 54: S466-S466. | Conference abstract |
| 1202 | Mehdi N, Butler DJ. Implementing CF rise pediatric to adult CF health care transition. Pediatric pulmonology.2018;53:440-440. | Conference abstract |
| 1203 | Mehtar S, Wanyoro A, Ogunsola F, Ameh EA, Nthumba P, Kilpatrick C, Revathi G, Antoniadou A, Giamarelou H, Apisarnthanarak A, Ramatowski JW. Implementation of surgical site infection surveillance in low-and middle-income countries a position statement for the international society for infectious diseases: a position statement for the international society for infectious diseases. International Journal of Infectious Diseases. 2020 Nov 1;100:123-31. | Study not about effectiveness |
| 1204 | Mello MM, Boothman RC, McDonald T, Driver J, Lembitz A, Bouwmeester D, Dunlap B, Gallagher T. Communication-and-resolution programs: the challenges and lessons learned from six early adopters. Health affairs. 2014 Jan 1;33(1):20-9. | Study not about effectiveness |
| 1205 | Meresman JF, Hunkeler EM, Hargreaves WA, Kirsch AJ, Robinson P, Green A, Mann EZ, Getzell M, Feigenbaum P. A case report: implementing a nurse telecare program for treating depression in primary care. Psychiatric Quarterly. 2003 Mar;74(1):61-73. | Study not about effectiveness |
| 1206 | Merry AF, Gargiulo DA, Bissett I, Cumin D, English K, Frampton C, Hamblin R, Hannam J, Moore M, Reid P, Roberts S. The effect of implementing an aseptic practice bundle for anaesthetists to reduce postoperative infections, the Anaesthetists Be Cleaner (ABC) study: protocol for a stepped wedge, cluster randomised, multi-site trial. Trials. 2019 Dec;20(1):1-4. | Protocol |
| 1207 | Merwin SL, Fornari A, Lane LB. A preliminary report on the initiation of a clinical research program in an orthopaedic surgery department: roadmaps and tool kits. Journal of surgical education. 2014 Jan 1;71(1):43-51. | Study not about effectiveness |
| 1208 | Meyer E, Nartowicz E, Burrow C, Townsend S, Nicol K. Blood Utilization Review: A Vital Initial Step in a Pediatric Blood Management Program. Transfusion. 2016;56:114A-114A. | Conference abstract |
| 1209 | Meyer H. At UPMC, improving care processes to serve patients better and cut costs. Health Affairs. 2011 Mar 1;30(3):400-3. | Study not about effectiveness |
| 1210 | Miani C, Marjanovic S, Jones MM, Marshall M, Meikle S, Nolte E. Barking, Havering and Redbridge university hospitals NHS trust fellowships in clinical leadership programme: an evaluation. Rand health quarterly. 2013;3(3). | Study not about effectiveness |
| 1211 | Micallef R, Grewal JS, Khan S, Wells J, Kayyali R. Health champions in South London: evaluation of training, and impact on public health. International Journal of Pharmacy Practice. 2019 Feb;27(1):71-9. | Study not about effectiveness |
| 1212 | Michtalik H, Bates B, Hody R, Phan P, Kantsiper M, Rennert-Ariev J, Winner L, Brotman D, Wachter P, Park E, Howell E. Increasing Value Across Four Hospitals through a Hospitalist-Led Quality Improvement Program: Top-Down Support for Bottom-up Change to Decrease Length of Stay and Cost of Care. In2015 Annual Research Meeting 2015 Jun 15. AcademyHealth. | Conference abstract |
| 1213 | Middleton DB, Fox DE, Nowalk MP, Skledar SJ, Sokos DR, Zimmerman RK, Ervin KA, Lin CJ. Overcoming barriers to establishing an inpatient vaccination program for pneumococcus using standing orders. Infection Control & Hospital Epidemiology. 2005 Nov;26(11):874-81. | Study not about effectiveness |
| 1214 | Middleton S, Comerford D, Lydtin A, Dale S, Cadilhac D, D'Este C, McElduff P, Hill K, Cheung NW, Levi C, Longworth M. Abstract NS23: Changing State-wide Stroke Practice: The QASC Implementation Project. Stroke. 2015 Feb;46(suppl_1):ANS23-. | Conference abstract |
| 1215 | Middleton S, Comerford D, Lydtin A, Dale S, Hill K, Dunne J, D'Este C, Long-worth M, Katalinic E, Tinsley M, Quinn C. The QASC Implementation Project: Implementing evidence based care in stroke services throughout NSW, Australia. Cerebrovascular diseases. 2014; 37:325-325. | Conference abstract |
| 1216 | Middleton S, Levi C, D'Este C, Grimshaw J, Cadilhac D, Considine JA, Cheung W, McInnes L, Dale S, Gerraty R, Fitzgerald M. T-3 stroke trial protocol: Triage, treatment and transfer of patients with stroke emergency departments. International Journal of Stroke. 2013 Aug 1;8:18-. | Conference abstract |
| 1217 | Middleton S, Lydtin A, Comerford D, Cadilhac DA, McElduff P, Dale S, Hill K, Longworth M, Ward J, Cheung NW, D'Este C. From QASC to QASCIP: successful Australian translational scale-up and spread of a proven intervention in acute stroke using a prospective pre-test/post-test study design. BMJ open. 2016 May 1;6(5):e011568. | Study not about effectiveness |
| 1218 | Middleton S, McInnes E, Dale S, Craig L, Schadewaldt V, Cadilhac D, Grimshaw J, Considine J, D'Este C, Cheung N.W, Levi C, Gerraty R. Process evaluation of the t3 stroke trial-clinicians views on factors for successful implementation of evidence-based stroke protocols in the emergency department. European Stroke Journal. 2018;3(1 Supplement 1):271 | Conference abstract |
| 1219 | Miech EJ, Rattray NA, Flanagan ME, Damschroder L, Schmid AA, Damush TM. Inside help: an integrative review of champions in healthcare-related implementation. SAGE open medicine. 2018 May 1;6:2050312118773261. | Letters to the editor/ Review studies |
| 1220 | Miller A, Chaboyer W. Captain and champion: nurses’ role in patient safety. | Letters to the editor/ Review studies |
| 1221 | Miller J, MacLean LB, Coward P, Broemeling AM. Developing strategies to enhance health services research capacity in a predominantly rural Canadian health authority.2009 | Study not about effectiveness |
| 1222 | Miller ME, Patel A, Schindler N, Hirsch K, Ming M, Weber S, Turner P, Howell MD, Arora VM, Oyler JL. Bridging the gap: interdepartmental quality improvement and patient safety curriculum created by hospital leaders, faculty, and trainees. Journal of graduate medical education. 2018 Oct;10(5):566-72. | Study not about effectiveness |
| 1223 | Miller R, Quinn BM, Smith L, Jenkins D. Leading The Patient Safety Charge-Keeping Our Patients Wound-Free. Journal of the American Medical Directors Association. 2020 Mar 1;21(3):B22-3. | Conference abstract |
| 1224 | Miller RH, Sim I, Newman J. Electronic medical records in solo/small groups: a qualitative study of physician user types. InMEDINFO 2004 2004 (pp. 658-662). Ios Press. | Study not about effectiveness |
| 1225 | Miller S, Dukelow S, Lam K, Gollega A, Kennedy K. FES'Train the Trainer': a Process of Moving Evidence-Based Care Into the Clinical Realm. Stroke. 2014;45: E280-E280. | Conference abstract |
| 1226 | Miller S, Gollega A, Dukelow S. 'Training the Trainers'-the Process of Effectively Moving Evidence-Based Functional Electrical Stimulation (FES) Into the Clinical Realm. Stroke.2013;44:E197-E197. | Conference abstract |
| 1227 | Miller SA. Efficiently Accessing the Evidence for Dental Practitioners. Journal of Evidence Based Dental Practice. 2009 Sep 1;9(3):113-21. | Not a champion |
| 1228 | Miller TA, Lisanti AJ, Witte MK, Elhoff JJ, Mahle WT, Uzark KC, Alexander N, Butler SC. A collaborative learning assessment of developmental care practices for infants in the cardiac intensive care unit. The Journal of pediatrics. 2020 May 1;220:93-100. | Study not about effectiveness |
| 1229 | Miller, L. Hanson, K, Jansen, G, Fankhanel, BY, Martois, E, Field, J, Amornvut, P. UNIT-BASED SKIN CARE CHAMPION PROGRAM PRACTICE INNOVATION...WOCN Society’s 49th Annual Conference, Salt Lake City, Utah, May 19-23, 2017. Journal of Wound, Ostomy & Continence Nursing ;44():S44-S44 | Conference abstract |
| 1230 | Miller-Petrie MK, Mazia G, Serpa M, Pooley B, Marshall M, Meléndez C, Vicuña M. Building alliances for improving newborn health in Latin America and the Caribbean. Revista Panamericana de Salud Pública. 2014;36:44-9. | Not a champion |
| 1231 | Miller-Willis KL, Joe V, Thomas M. 499 Shifting to 1% Chlorhexidine Gluconate Burn Wound Bathing: And Evidence-Informed Change Project. Journal of Burn Care & Research. 2019 Mar 9;40(Supplement_1):S226-. | Conference abstract |
| 1232 | Millery M, Kukafka R. Health information technology and quality of health care: strategies for reducing disparities in underresourced settings. Medical Care Research and Review. 2010 Oct;67(5_suppl):268S-98S. | Study not about effectiveness |
| 1233 | Minear S, Wachman EM. Management of newborns with prenatal opioid exposure: one institution's journey. Clinical therapeutics. 2019 Sep 1;41(9):1663-8. | Study not about effectiveness |
| 1234 | Mirigo Gichuhi M, Gomersall JC. Implementation of best practice for dyspepsia management in an outpatient hospital setting in K enya. International Journal of Evidence‐Based Healthcare. 2013 Sep;11(3):187-93. | Study not about effectiveness |
| 1235 | Miske L, Hickey E, Stellar J, Kramer B. Hospital acquired skin injury related to the use of noninvasive positive pressure ventilation. Chest. 2012 Oct 1;142(4):772A. | Conference abstract |
| 1236 | Mitchell C, Van Son C, Santovito-Carducci G. Infection Prevention in the Outpatient Physician Clinic. American Journal of Infection Control. 2015 Jun 2;43(6):S33-4. | Conference abstract |
| 1237 | Mitchell E, Lawes H. Falls education for practitioners: Auditing a three-tier learning approach. Nursing older people. 2008 Feb 1;20(1). | Not a champion |
| 1238 | Mitton C, Adair CE, McKenzie E, Patten S, Waye-Perry B, Smith N. Designing a knowledge transfer and exchange strategy for the Alberta Depression Initiative: contributions of qualitative research with key stakeholders. International Journal of Mental Health Systems. 2009 Dec;3(1):1-0. | Study not about effectiveness |
| 1239 | Mitton C, Donaldson C. Twenty-five years of programme budgeting and marginal analysis in the health sector, 1974-1999. Journal of Health Services Research & Policy. 2001 Oct 1;6(4):239-48. | Study not about effectiveness |
| 1240 | Miyawaki CE, Belza B, Kohn MJ, Petrescu-Prahova M. Champions of an older adult exercise program: Believers, promoters, and recruiters. Journal of Applied Gerontology. 2018 Jun;37(6):728-44. | Study not about effectiveness |
| 1241 | Mladenovic J, Shea JA, Duffy FD, Lynn LA, Holmboe ES, Lipner RS. Variation in internal medicine residency clinic practices: assessing practice environments and quality of care. Journal of general internal medicine. 2008 Jul 1;23(7):914-20. | Study not about effectiveness |
| 1242 | Modes M, Davis A, Farnan J, Arora V. Ordering Wisely: Engaging Faculty to Champion High-Value Care Initiatives. American Journal of Medical Quality. 2016 Jul 1;31(4):380-1. | Letters to the editor/ Review studies |
| 1243 | Modica R, Raja S, Quinones M, Diongon H, Figueredo J, Coyle C. Reaching our goal of zero catheter associated urinary tract infections (CAUTI). American Journal of Infection Control. 2014 Jun 1;42(6):S102-3. | Conference abstract |
| 1244 | Moffat MA, Sheikh A, Price D, Peel A, Williams S, Cleland J, Pinnock H. Can a GP be a generalist and a specialist? Stakeholders views on a respiratory General Practitioner with a special interest service in the UK. BMC Health Services Research. 2006 Dec;6(1):1-8. | Study not about effectiveness |
| 1245 | Mohammadi JJ, Franks K, Hines S. Effectiveness of professional oral health care intervention on the oral health of residents with dementia in residential aged care facilities: a systematic review protocol. JBI Evidence Synthesis. 2015 Oct 1;13(10):110-22. | Letters to the editor/ Review studies |
| 1246 | Mohd Yusof M, Matsumura Y, Takeda T, Mihara N, Abdul Rahman R. Managing health information systems-induced medication error: Comparison between hospitals in Japan and Malaysia. European Journal of Epidemiology. 2016;31(Supplement 1):S136-S137 | Conference abstract |
| 1247 | Monahan JL, Scheirer MA. The role of linking agents in the diffusion of health promotion programs. Health Education Quarterly. 1988 Dec;15(4):417-33. | Study not about effectiveness |
| 1248 | Money AG, Barnett J, Kuljis J, Craven MP, Martin JL, Young T. The role of the user within the medical device design and development process: medical device manufacturers' perspectives. BMC medical informatics and decision making. 2011 Dec;11(1):1-2. | Study not about effectiveness |
| 1249 | Moore A. Investment in quality saves NHS trust millions. Nursing Standard (2014+). 2017 Nov 15;32(12):22. | Study not about effectiveness |
| 1250 | Moore B, Dorflinger L, Goulet J, Lee A, Kerns R. (449) Opioid prescribing and the Stepped Care Model of Pain Management in the Veteran's Health Administration. The Journal of Pain. 2014 Apr 1;15(4):S88. | Conference abstract |
| 1251 | Moore JE, Grouchy M, Graham ID, Shandling M, Doyle W, Straus SE. The Council of academic hospitals of Ontario (CAHO) adopting research to improve care (ARTIC) program: reach, sustainability, spread and lessons learned from an implementation funding model. Healthcare Policy. 2016 May;11(4):27. | Study not about effectiveness |
| 1252 | Moore JE, Marquez C, Dufresne K, Harris C, Park J, Sayal R, Kastner M, Kelloway L, Munce SE, Bayley M, Meyer M. Supporting the implementation of stroke quality-based procedures (QBPs): a mixed methods evaluation to identify knowledge translation activities, knowledge translation interventions, and determinants of implementation across Ontario. BMC health services research. 2018 Dec;18(1):1-3. | Study not about effectiveness |
| 1253 | Moore JE, Mascarenhas A, Marquez C, Almaawiy U, Chan WH, D’Souza J, Liu B, Straus SE. Mapping barriers and intervention activities to behaviour change theory for Mobilization of Vulnerable Elders in Ontario (MOVE ON), a multi-site implementation intervention in acute care hospitals. Implementation Science. 2014 Dec;9(1):1-9. | Study not about effectiveness |
| 1254 | Moore K, Johnson G, Fortner BV, Houts AC. The AIM Higher Initiative: new procedures implemented for assessment, information, and management of chemotherapy toxicities in community oncology clinics. Clinical journal of oncology nursing. 2008 Apr 1;12(2). assessment,​ information,​ and management of chemotherapy toxicities in community oncology clinics. *#journal#*,​ 12(2),​ 229 | Study not about effectiveness |
| 1255 | Moore R, Wilkinson J, Masterson A. The Department of Health: championing investment and capacity building in nursing research. Nursing Management (through 2013). 2005 Nov 1;12(7):15. | Letters to the editor/ Review studies |
| 1256 | Morcuende JA, Cook TM. The Ponseti Method in Low and Middle Income Countries: Challenges and Lessons Learned. Foot and ankle clinics. 2015 Dec 1;20(4):547-54. | Study not about effectiveness |
| 1257 | Moreau MA. Changing Breastfeeding Culture through Staff Training. Journal of Obstetric, Gynecologic, & Neonatal Nursing. 2015 Jun;44(s1):S31-. | Duplicate |
| 1258 | Moreau, M.A. Changing Breastfeeding Culture through Staff Training...Proceedings of the 2015 AWHONN Convention. JOGNN: Journal of Obstetric, Gynecologic & Neonatal Nursing ;44():S31-S31 | Conference abstract |
| 1259 | Morel MN, Luu C, Vachon B, Maillet L, Gaboury I. How can we make community reintegration following stroke better? Perspectives of primary healthcare professionals and community organization workers. International Journal of Stroke; 14(Supplementary 3):40-40. | Conference abstract |
| 1260 | Morgan D, Kosteniuk J, O’Connell ME, Kirk A, Stewart NJ, Seitz D, Bayly M, Chow AF, Elliot V, Daku J, Hack T. Barriers and facilitators to development and implementation of a rural primary health care intervention for dementia: a process evaluation. BMC health services research. 2019 Dec;19(1):1-8. | Study not about effectiveness |
| 1261 | Morgan TO. Blood conservation: the CEO perspective. Journal of cardiothoracic and vascular anesthesia. 2004 Aug 1;18(4):S15-7. | Study not about effectiveness |
| 1262 | Morgan TO. Cost, quality, and risk: Measuring and stopping the hidden costs of coronary artery bypass graft surgery. American journal of health-system pharmacy. 2005 Sep 15;62(18_Supplement_4):S2-5. | Study not about effectiveness |
| 1263 | Morgenstern S, Puett L, Ziegfeld S, Stewart D. 221 Implementation of a Burn Champion Workgroup in a Mixed Medical-Surgical Pediatric Intensive Care Unit. Journal of Burn Care & Research. 2019 Mar 9;40(Supplement_1):S90-1. | Conference abstract |
| 1264 | Morgenstern S.; Puett L.; Ziegfeld S.; Stewart D.Implementation of a burn champion workgroup in a mixed medical-surgical pediatric intensive care unit. Journal of Burn Care and Research / 2019;40(Supplement 1):S90-S91 | Conference abstract |
| 1265 | Mørk A, Krupp A, Hankwitz J, Malec A. Using Kotter's change framework to implement and sustain multiple complementary ICU initiatives. Journal of nursing care quality. 2018 Jan 1;33(1):38-45. | Study not about effectiveness |
| 1266 | Morris A, Naeem MA, Bal A, Murray T, Thomas T, DeGregory K, Lewis J, Keng M. Prospective Study of an Antifungal Program to Reduce Invasive Fungal Infections in Patients with Acute Myeloid Leukemia Receiving Induction and Re-Induction Chemotherapy. Blood. 2017 Dec 7;130(Supplement 1):345-. | Conference abstract |
| 1267 | Morrison JL, Swartz J, Raum D, Conigliaro R. A patient-centered medical home (PCMH) resident rotation: teaching internal medicine residents the principles of PCMH through community-based primary care. Journal of General Internal Medicine. 2014;29:S503-S504. | Conference abstract |
| 1268 | Morrison LJ, Brooks SC, Dainty KN, Dorian P, Needham DM, Ferguson ND, Rubenfeld GD, Slutsky AS, Wax RS, Zwarenstein M, Thorpe K. Improving use of targeted temperature management after out-of-hospital cardiac arrest: a stepped wedge cluster randomized controlled trial. Critical care medicine. 2015 May 1;43(5):954-64. | Study not about effectiveness |
| 1269 | Morrison LJ, Brooks SC, Dainty KN, Dorian P, Needham DM, Ferguson ND, Rubenfeld GD, Slutsky AS, Wax RS, Zwarenstein M, Thorpe K. Improving use of targeted temperature management after out-of-hospital cardiac arrest: a stepped wedge cluster randomized controlled trial. Critical care medicine. 2015 May 1;43(5):954-64. | Study not about effectiveness |
| 1270 | Morrone KA, Kalluri D, Davila JG, Edusei G, Shoberu B, Rosario J, Figueiredo LM, Carullo VP, Weiss M, Rinke M, Manwani D. Decreased Hospital Readmissions for Vaso Occlusive Crisis with Implementation of a Sickle Cell Pain Action Plan (SPAP). | Conference abstract |
| 1271 | Mosoiu, D, Eniu, A. Barriers towards establishing palliative care in eastern Europe and prospects for improvements in the future: Romania as an example. Palliative Care: Perspectives, Practices and Impact on Quality of Life.2017;(1):211-224 | Not a champion |
| 1272 | Mott B, Horgan K, Flesch L, Hayward M, Demmel K, Morrison C. Moving a Bone Marrow Transplant Unit Towards a High Reliability Unit. Biology of Blood and Marrow Transplantation. 2014 Feb 1;20(2):S305. | Conference abstract |
| 1273 | Moussa L, Benrimoj SI, Garcia-Cardenas V.The Most frequently used facilitation strategies during the implementation of innovations in healthcare practice: A systematic reviewPharmacy Practice / 2018;16(Supplement 1). | Conference abstract |
| 1274 | Mouzoon ME, Munoz FM, Greisinger AJ, Brehm BJ, Wehmanen OA, Smith FA, Markee JA, Glezen WP. Improving influenza immunization in pregnant women and healthcare workers. The American journal of managed care. 2010 Mar 1;16(3):209-16. | Study not about effectiveness |
| 1275 | Moynihan KM, Snaman JM, Kaye EC, Morrison WE, DeWitt AG, Sacks LD, Thompson JL, Hwang JM, Bailey V, Lafond DA, Wolfe J. Integration of pediatric palliative care into cardiac intensive care: a champion-based model. Pediatrics. 2019 Aug 1;144(2). | Letters to the editor/ Review studies |
| 1276 | Mudge AM, McRae P, Cruickshank M. Eat walk engage: an interdisciplinary collaborative model to improve care of hospitalized elders. American Journal of Medical Quality. 2015 Jan;30(1):5-13. | Study not about effectiveness |
| 1277 | Mueller CJ. Implementing a Program to Standardize Central Line Maintenance. American Journal of Infection Control. 2020 Aug 1;48(8):S5-6. | Conference abstract |
| 1278 | Mukaba T, Binanga A, Fohl S, Bertrand JT. Family planning policy environment in the Democratic Republic of the Congo: levers of positive change and prospects for sustainability. Global Health: Science and Practice. 2015 Jun 1;3(2):163-73. | Study not about effectiveness |
| 1279 | Mulgrew H. An audit on the use and documentation of Intra-operative Fluid Management Technology (IOFMT) in the anaesthetic department. Anaesthesia. 2014;69:130-130. | Conference abstract |
| 1280 | Mullaney K. Decreasing Catheter Associated Urinary Tract Infections (CAUTI) using the BREAKTHROUGH (LEAN) Method. American Journal of Infection Control. 2012 Jun 1;40(5):e130-1. | Conference abstract |
| 1281 | Mullen C, Gavin‐Daley AN, Kilgannon H, Swift J. Nurse COnsultants 10 years on: an insight to the role for nurse managers. Journal of Nursing Management. 2011 Sep;19(6):820-31. | Not a champion |
| 1282 | Muller AC, Hujcs M, Dubendorf P, Harrington PT. Sustaining excellence: clinical nurse specialist practice and magnet designation. Clinical Nurse Specialist. 2010 Sep 1;24(5):252-9. | Study not about effectiveness |
| 1283 | Muller M, Siddiqui N. P14. 02 A multi-disciplinary team's multi-modal strategy to increase hand hygiene compliance at a Toronto teaching hospital. Journal of Hospital Infection. 2010(76):S45-6. | Conference abstract |
| 1284 | Mullins ME, Kozlowski SW, Schmitt N, Howell AW. The role of the idea champion in innovation: The case of the Internet in the mid-1990s. Computers in Human Behavior. 2008 Mar 1;24(2):451-67. | Not within a health care setting |
| 1285 | Mulvale G, Embrett M, Razavi SD. ‘Gearing Up’to improve interprofessional collaboration in primary care: a systematic review and conceptual framework. BMC family practice. 2016 Dec;17(1):1-3. | Letters to the editor/ Review studies |
| 1286 | Munro S, Manski R, Donnelly KZ, Agusti D, Stevens G, Banach M, Boardman MB, Brady P, Bradt CC, Foster T, Johnson DJ. Investigation of factors influencing the implementation of two shared decision-making interventions in contraceptive care: a qualitative interview study among clinical and administrative staff. Implementation Science. 2019 Dec;14(1):1-6. | Not a champion |
| 1287 | Murphree J, Englert J, Koch K, Davis KM, Heer J. North Mississippi Medical Center: A Focus on Quality, Safety, and Financial Critical Success Factors. The Joint Commission Journal on Quality and Patient Safety. 2005 Oct 1;31(10):545-53. | Not a champion |
| 1288 | Murphy C, Anderson S, Bhatt J, Gerritsen J, Gibson E, Huffman S, Keddy A, Kellam M, Lynch J, MacNeil J, Martin C. Taking Action: A Community Hospital Applies Stroke Best Practices. Stroke. 2014 ;45 (12): E277-E277. | Conference abstract |
| 1289 | Murray J, Barker A, Underdown K, Lynch E. Why is oral care not a priority on acute stroke units?. InSTROKE 2018: Bridging the continuum 2018 Aug 1. | Conference abstract |
| 1290 | Musselman KT, Moczygemba LR, Pierce AL, Plum MB, Brokaw DK, Kelly DL. Development and implementation of clinical pharmacist services within an integrated medical group. Journal of pharmacy practice. 2017 Feb;30(1):75-81. | Study not about effectiveness |
| 1291 | Myburgh H, Murphy JP, van Huyssteen M, Foster N, Grobbelaar CJ, Struthers HE, McIntyre JA, Hurter T, Peters RP. Implementation of an electronic monitoring and evaluation system for the antiretroviral treatment programme in the Cape Winelands district, South Africa: a qualitative evaluation. PloS one. 2015 May 12;10(5):e0127223. | Study not about effectiveness |
| 1292 | Myers G, Côté‐Arsenault D, Worral P, Rolland R, Deppoliti D, Duxbury E, Stoecker M, Sellers K. A cross‐hospital exploration of nurses’ experiences with horizontal violence. Journal of nursing management. 2016 Jul;24(5):624-33. | Study not about effectiveness |
| 1293 | Myers KM, Vander Stoep A, McCarty CA, Klein JB, Palmer NB, Geyer JR, Melzer SM. Child and adolescent telepsychiatry: variations in utilization, referral patterns and practice trends. Journal of Telemedicine and Telecare. 2010 Apr;16(3):128-33. | Study not about effectiveness |
| 1294 | Myers SS, Clark MD, Russell JA, Graham CC, Stultz MB, Reidy KM. Focusing measures for performance-based privileging of physicians on improvement. The Joint Commission Journal on Quality and Patient Safety. 2008 Dec 1;34(12):724-33. | Not a champion |
| 1295 | Myrick KM. Improving follow-up care for fragility fractures: An evidence-based practice initiative. University of Connecticut; 2010. | Study not about effectiveness |
| 1296 | Na S, Kuan WS, Mahadevan M, Li CH, Shrikhande P, Ray S, Batech M, Nguyen HB, ATLAS investigators. Implementation of early goal-directed therapy and the surviving sepsis campaign resuscitation bundle in Asia. International journal for quality in health care. 2012 Oct 1;24(5):452-62. | Study not about effectiveness |
| 1297 | Nabukeera J, Katarikawe E, Conecker GA. Delivering High-Impact, Evidence-based Interventions to Save the Lives of Women and Babies in Insingiro District. Annals of Global Health. 2017 Apr 7;83(1). | Conference abstract |
| 1298 | Najjar PA, Whang EE, Urman RD, McGrath CT, Beloff JR, Bleday R. Institution-wide implementation strategies, finance, and administration for enhanced recovery after surgery programs. International anesthesiology clinics. 2017 Oct 1;55(4):90-100. | Study not about effectiveness |
| 1299 | Nanji KC, Cina J, Patel N, Churchill W, Gandhi TK, Poon EG. Overcoming barriers to the implementation of a pharmacy bar code scanning system for medication dispensing: a case study. Journal of the American Medical Informatics Association. 2009 Sep 1;16(5):645-50. | Study not about effectiveness |
| 1300 | Naranjo A, Ojeda S, Giner M, Balcells-Oliver M, Canals L, Cancio JM, Duaso E, Mora-Fernández J, Pablos C, González A, Lladó B. Best Practice Framework of Fracture Liaison Services in Spain and their coordination with Primary Care. Archives of osteoporosis. 2020 Dec;15(1):1-7. | Study not about effectiveness |
| 1301 | Nathan JK, Foley J, Hoang T, Hiner J, Brooks S, Gendreau JL, Meurer WJ, Pandey AS, Adelman EE. The stroke navigator: meaningful use of the electronic health record to efficiently report inpatient stroke care quality. Journal of the American Medical Informatics Association. 2018 Nov;25(11):1534-9. | Study not about effectiveness |
| 1302 | Naunton R. 129 How health and care professionals use goal setting after attending me first training to apply child and young person (CYP) centered communication. | Conference abstract |
| 1303 | Nauton R, McCulloch J. 131 Me first: helping children and young people to reach an agreement with professionals and parents in health and social care using children and young people centred communication training. | Conference abstract |
| 1304 | Naylor CD. Better care and better outcomes: the continuing challenge. JAMA. 1998 May 6;279(17):1392-4. | Study not about effectiveness |
| 1305 | Nease DE, Nutting PA, Graham DG, Dickinson WP, Gallagher KM, Jeffcott-Pera M. Sustainability of depression care improvements: success of a practice change improvement collaborative. The Journal of the American Board of Family Medicine. 2010 Sep 1;23(5):598-605. | Study not about effectiveness |
| 1306 | Nease Jr DE, Nutting PA, Dickinson WP, Bonham AJ, Graham DG, Gallagher KM, Main DS. Inducing sustainable improvement in depression care in primary care practices. The Joint Commission Journal on Quality and Patient Safety. 2008 May 1;34(5):247-55. | Study not about effectiveness |
| 1307 | Nelson C, Roy LA, Wallace HJ. Radiation Oncology Incident Learning System (RO-ILS): Increasing stakeholder participation for safety and quality improvement. | Conference abstract |
| 1308 | Nelson DE, Reynolds JH, Luke DA, Mueller NB, Eischen MH, Jordan J, Lancaster RB, Marcus SE, Vallone D. Successfully maintaining program funding during trying times: lessons from tobacco control programs in five states. Journal of Public Health Management and Practice. 2007 Nov 1;13(6):612-20. | Not within a health care setting |
| 1309 | Nembhard IM, Savage S, Labao I. Overcoming Implementation Challenges in Health Care: A Qualitative Study of What Project Leaders Do. InAcademy of Management Proceedings 2014 (Vol. 2014, No. 1, p. 16598). Briarcliff Manor, NY 10510: Academy of Management. | Conference abstract |
| 1310 | Neuman MD, Kennelly AM, Tosi LL. Breakout session: sex/gender and racial/ethnic disparities in the care of osteoporosis and fragility fractures. Clinical Orthopaedics and Related Research®. 2011 Jul;469(7):1936-40. | Study not about effectiveness |
| 1311 | Ng AW, Sage S. Igniting the potential of wound care champions. Australian Nursing and Midwifery Journal. 2017 Mar;24(8):38-9. | Study not about effectiveness |
| 1312 | Nga E, Macleod-Collins E, Taylor T, Watson B, Keidan A. From 4% to over 90%: venous thromboembolism risk assessment (VTE RA) in a district general hospital: 200. British Journal of Haematology. 2011 Apr;153. | Conference abstract |
| 1313 | Ngai EW, Law CC, Wat FK. Examining the critical success factors in the adoption of enterprise resource planning. Computers in industry. 2008 Aug 1;59(6):548-64. | Not within a health care setting |
| 1314 | Ngune I, Jiwa M, Dadich A, Lotriet J, Sriram D. Effective recruitment strategies in primary care research: a systematic review. Quality in primary care. 2012 May 1;20(2):115-23. | Letters to the editor/ Review studies |
| 1315 | Nguyen HB, Oh J, Otero RM, Burroughs K, Wittlake WA, Corbett SW. Standardization of severe sepsis management: a survey of methodologies in academic and community settings. The Journal of emergency medicine. 2010 Feb 1;38(2):122-32. | Study not about effectiveness |
| 1316 | Nguyen HQ, Moy ML, Fan VS, Gould MK, Xiang A, Bailey A, Desai S, Coleman KJ. Applying the pragmatic-explanatory continuum indicator summary to the implementation of a physical activity coaching trial in chronic obstructive pulmonary disease. Nursing outlook. 2018 Sep 1;66(5):455-63. | Study not about effectiveness |
| 1317 | Nguyen OK, Kruger J, Greysen SR, Lyndon A, Goldman LE. The silo next door: primary care leaders'perspectives on collaborating with hospitals during hospital-to-clinic care transitions in the safety net. Journal of general internal medicine 2013 Jun 1 (Vol. 28, pp. S212-S213). | Conference abstract |
| 1318 | Niazi SK, Spaulding A, Vargas E, Chauhan M, Nordan L, Vizzini M, Puspitasari AJ, Uitti RJ, Rummans T. Feasibility Study of Three-Phase Implementation of International Consortium for Health Outcomes Measurement Depression and Anxiety Standard Set in an Outpatient Consultation-Liaison Psychiatry Practice. Psychosomatics. 2020 Jan 1;61(1):8-18. | Study not about effectiveness |
| 1319 | Nickel NC, Taylor EC, Labbok MH, Weiner BJ, Williamson NE. Applying organisation theory to understand barriers and facilitators to the implementation of baby-friendly: a multisite qualitative study. Midwifery. 2013 Aug 1;29(8):956-64. | Study not about effectiveness |
| 1320 | Niederhauser A, Lukas CV, Parker V, Ayello EA, Zulkowski K, Berlowitz D. Comprehensive programs for preventing pressure ulcers: a review of the literature. Advances in skin & wound care. 2012 Apr 1;25(4):167-88. | Letters to the editor/ Review studies |
| 1321 | Nils P. Minimizing social isolation in low-income communities: practice, and implementation strategies for dementia care (Doctoral dissertation, University of Southern California). | Study not about effectiveness |
| 1322 | Nitsch KP, Stipp K, Gracz K, Ehrlich-Jones L, Graham ID, Heinemann AW. Integrating Spinal Cord Injury–Quality of Life instruments into rehabilitation: Implementation science to guide adoption of patient-reported outcome measures. The journal of spinal cord medicine. 2020 Jan 24:1-9. | Study not about effectiveness |
| 1323 | Njeuhmeli E, Schnure M, Vazzano A, Gold E, Stegman P, Kripke K, Tchuenche M, Bollinger L, Forsythe S, Hankins C. Using mathematical modeling to inform health policy: a case study from voluntary medical male circumcision scale-up in eastern and southern Africa and proposed framework for success. PloS one. 2019 Mar 18;14(3):e0213605. | Study not about effectiveness |
| 1324 | Noor I, Alevi R, Theriot J, Gupta R, Davis-Lorton MA, Aquino M, Fonacier LS, Stukus DR, Gubernick R. Improved Management For Children With Asthma And Anaphylaxis Via A Web-based Quality Improvement Project. Journal of Allergy and Clinical Immunology. 2017 Feb 1;139(2):AB56. | Conference abstract |
| 1325 | Norris CM, Tannenbaum C, Pilote L, Wong G, Cantor WJ, McMurtry MS. Systematic incorporation of sex‐specific information into clinical practice guidelines for the management of ST‐Segment–Elevation Myocardial Infarction: Feasibility and outcomes. Journal of the American Heart Association. 2019 Apr 2;8(7):e011597. | Study not about effectiveness |
| 1326 | North CM, Attia EF, Rudd KE, Siddharthan T, Papali A, Çoruh B, Carter EJ, Christiani DC, Richards JB, Huang L, Engelberg R. Global Health–related Training Opportunities. A National Survey of Pulmonary and Critical Care Medicine Fellowship Programs. Annals of the American Thoracic Society. 2019 Sep;16(9):1171-8. | Study not about effectiveness |
| 1327 | Northridge ME, Kavathe R, Zanowiak J, Wyatt L, Singh H, Islam N. Implementation and dissemination of the Sikh American families oral health promotion program. Translational behavioral medicine. 2017 Sep 1;7(3):435-43. | Study not about effectiveness |
| 1328 | Northridge ME, Kum SS, Chakraborty B, Greenblatt AP, Marshall SE, Wang H, Kunzel C, Metcalf SS. Third places for health promotion with older adults: using the consolidated framework for implementation research to enhance program implementation and evaluation. Journal of Urban Health. 2016 Oct;93(5):851-70. | Study not about effectiveness |
| 1329 | Northway T, Mawdsley C. The Canadian ICU Collaborative: on being a nurse champion. influencing support and change. Dynamics (Pembroke, Ont.). 2007 Jan 1;18(3):25-7. | Study not about effectiveness |
| 1330 | Norton E. Implementing the Universal Protocol Hospital‐Wide. AORN journal. 2007 Jun;85(6):1187-97. | Study not about effectiveness |
| 1331 | Novack S. A Multi-Disciplinary Approach for VAP Prevention: Building a Culture of Safety for ICU Patients. Ajic (american Journal of Infection Control). 2009 Jun;37(5). | Conference abstract |
| 1332 | Novick G, Womack JA, Lewis J, Stasko EC, Rising SS, Sadler LS, Cunningham SC, Tobin JN, Ickovics JR. Perceptions of barriers and facilitators during implementation of a complex model of group prenatal care in six urban sites. Research in nursing & health. 2015 Dec;38(6):462-74. | Study not about effectiveness |
| 1333 | Nowalk MP, Lin CJ, Pavlik VN, Brown AE, Zhang S, Moehling KK, Raviotta JM, South-Paul JE, Hawk M, Ricci EM, Middleton DB. Using the 4 pillars™ practice transformation program to increase adult Tdap immunization in a randomized controlled cluster trial. Vaccine. 2016 Sep 22;34(41):5026-33. | Study not about effectiveness |
| 1334 | Nowalk MP, Moehling KK, Zhang S, Raviotta JM, Zimmerman RK, Lin CJ. Using the 4 Pillars™ to Increase Vaccination among High-risk Adults: Who Benefits?. The American journal of managed care. 2017 Nov;23(11):651. | Study not about effectiveness |
| 1335 | Ntizimira CR, Nkurikiyimfura JL, Mukeshimana O, Ngizwenayo S, Mukasahaha D, Clancy C. Palliative care in Africa: a global challenge. ecancermedicalscience. 2014;8. | Study not about effectiveness |
| 1336 | Nugent J, Hutton T, Zambrano M, De Ocampo SK. The Age of Unicorns: Successful Implementation and Highlights of a Sustainable Unit Based Infection Control Champion Program. American Journal of Infection Control. 2016 Jun 2;44(6):S64. | Conference abstract |
| 1337 | Nyblade LC, Addo N, KA GE, CS JS. Reducing health worker stigma and discrimination is critical to reaching 90-90-90 targets and is possible: evaluation results of a whole-facility approach in Ghana. In22nd International AIDS Conference, Amsterdam, Netherlands 2018 Jul 1 (pp. 23-27). | Conference abstract |
| 1338 | Nyombi T., Rahimzai M., Aloyo J., Karamagi E. Getting from 22 to 125: Scaling up interventions to improve outcomes of hiv-positive mother-baby pairs in Northern Uganda. International Journal for Quality in Health Care / 2017;29(Supplement 1):50 | Conference abstract |
| 1339 | Nypaver M, Macy M, Pribble J, Uren B, Kocher K, Levine G. The Michigan Emergency Department Improvement Collaborative: A novel model for implementing large scale practice change in pediatric emergency care. | Conference abstract |
| 1340 | O Reilly A., Langan C. Whose cares about mouth care? An inter-disciplinary quality improvement initiative in a large acute teaching hospital. Dysphagia. 2019;34(5):777-778 | Conference abstract |
| 1341 | O’Connor S, Bigelow C, Langlois M, Hutchison J, Tyrrell A. Advance Care Planning Innovation-Listen, Plan, Act, Learn, Change. InJournal of Pain and Symptom Management 2016 Dec 1 (Vol. 52, No. 6, pp. E106-E106). 360 PARK AVE SOUTH, NEW YORK, NY 10010-1710 USA: ELSEVIER SCIENCE INC. | Conference abstract |
| 1342 | O’Reilly GM, Gabbe B, Braaf S, Cameron PA. An interview of trauma registry custodians to determine lessons learnt. Injury. 2016 Jan 1;47(1):116-24. | Study not about effectiveness |
| 1343 | O’Rourke N, O’Toole E. 22 Building capability, leadership and a home for evidence based medicine in ireland. | Conference abstract |
| 1344 | O'boyle AM, Graham E, Ellis M. In reach into Nursing Homes Education, Training and Development Programme. International Journal of Integrated Care. 2017 Oct 17;17(5). | Conference abstract |
| 1345 | Obringer E, Bartlett A. An Integrated Approach to Influenza Vaccination for Pediatric Subspecialty Patients. | Conference abstract |
| 1346 | O'Connor L, Oliver J, Mulchay C, Hargis J, Leitner R. ADMISSION 2 DISCHARGE TOGETHER (A2D)-improving the hospital journey for people with an intellectual disability. International Journal of Integrated Care. 2017 Jul 11;17(3). | Conference abstract |
| 1347 | O'Connor O. The MediStori. A personal health record and standardised self-management toolkit which can improve integrated care systems. International Journal of Integrated Care. 2017 Oct 17;17(5). | Conference abstract |
| 1348 | O'Connor P, Creager J, Mooney S, Laizner AM, Ritchie JA. Taking aim at fall injury adverse events: best practices and organizational change. Healthcare quarterly (Toronto, Ont.). 2006 Jan 1;9:43-9. | Study not about effectiveness |
| 1349 | Oduola S, Wykes T, Robotham D, Craig TK. What is the impact of research champions on integrating research in mental health clinical practice? A quasiexperimental study in South London, UK. BMJ open. 2017 Sep 1;7(9):e016107. | Study not about effectiveness |
| 1350 | Ofman JJ, Segal R, Russell WL, Cook DJ, Sandhu M, Maue SK, Lowenstein EH, Pourfarzib R, Blanchette E, Ellrodt G, Weingarten SR. A randomized trial of an acid-peptic disease management program in a managed care environment. American Journal of Managed Care Jun 2003;9(6):425-433 | Study not about effectiveness |
| 1351 | Ohanian S, Gaines-Hill S. Reduction of CAUTI rates organization wide begins in the Emergency Department. American Journal of Infection Control. 2019 Jun 1;47(6):S37. | Conference abstract |
| 1352 | Ojiako U, Maguire S, Koh L, Grainger T, Wainwright D. Softer perspectives on enhancing the patient experience using IS/IT. International journal of health care quality assurance. 2010 Feb 9. | Study not about effectiveness |
| 1353 | Okafor N, Payne VL, Chathampally Y, Miller S, Doshi P, Singh H. Using voluntary reports from physicians to learn from diagnostic errors in emergency medicine. Emergency Medicine Journal. 2016 Apr 1;33(4):245-52. | Study not about effectiveness |
| 1354 | Okafor N.; Payne V.; Chathampally Y.; Miller S.; Doshi P.; Singh H. Using voluntary physician reporting to learn from diagnostic errors in emergency medicine. Diagnosis. 2015;2(1):eA13 | Conference abstract |
| 1355 | O'Keefe‐McCarthy S, Santiago C, Lau G. Ventilator‐associated pneumonia bundled strategies: an evidence‐based practice. Worldviews on Evidence‐Based Nursing. 2008 Dec;5(4):193-204. | Study not about effectiveness |
| 1356 | Okoroh EM, Kane DJ, Gee RE, Kieltyka L, Frederiksen BN, Baca KM, Rankin KM, Goodman DA, Kroelinger CD, Barfield WD. Policy change is not enough: engaging provider champions on immediate postpartum contraception. American journal of obstetrics and gynecology. 2018 Jun 1;218(6):590-e1.82 | Study not about effectiveness |
| 1357 | Olan I, Gunn K. Blood is a gift-why use two when one will do. InBritish Journal of Anaesthesia 2012 Mar 1 (Vol. 108, pp. 10-11). THE BOULEVARD, LANGFORD LANE, KIDLINGTON, OXFORD OX5 1GB, OXON, ENGLAND: ELSEVIER SCI LTD. | Conference abstract |
| 1358 | O'Leary ST, Pyrzanowski J, Brewer SE, Sevick C, Dickinson LM, Dempsey AF. Effectiveness of a multimodal intervention to increase vaccination in obstetrics/gynecology settings. Vaccine. 2019 Jun 6;37(26):3409-18. | Study not about effectiveness |
| 1359 | Oliffe JL, Halpin M, Bottorff JL, Hislop TG, McKenzie M, Mroz L. How prostate cancer support groups do and do not survive: British Columbian perspectives. American Journal of Men's Health. 2008 Jun;2(2):143-55. | Study not about effectiveness |
| 1360 | Olowo G.; Teressa G.; Abbasi S.; Goolsarran N. The resident is in charge! a novel approach to improve early hospital discharge rates. Journal of Hospital Medicine. 2018;13(4 Supplement 1). | Conference abstract |
| 1361 | Olsen-Scribner RJ, Hayes C, Pottinger P. Sustaining reduction of catheter-associated urinary tract infection (CAUTI)-outcomes after two educational methods in a regional university-affiliated medical center. American Journal of Infection Control. 2014 Jun 1;42(6):S22. | Conference abstract |
| 1362 | Olson CA, Tooman TR, Alvarado CJ. Knowledge systems, health care teams, and clinical practice: a study of successful change. Advances in Health Sciences Education. 2010 Oct;15(4):491-516. | Study not about effectiveness |
| 1363 | Olson M, Ammon A, Page C, Larkin L. Building integrated teams to address mental and behavioral health needs in rural primary care: The Western Colorado COEARTH project. International Journal of Integrated Care. 2019 Aug 8;19(4). | Conference abstract |
| 1364 | Olson R, Garite TJ, Fishman A, Andress IF. Obstetrician/gynecologist hospitalists: can we improve safety and outcomes for patients and hospitals and improve lifestyle for physicians?. American journal of obstetrics and gynecology. 2012 Aug 1;207(2):81-6. | Study not about effectiveness |
| 1365 | Omari R, Zotor F, Tagwireyi J, Lokosang L. Advocacy for scaling up biofortified crops for improved micronutrient status in Africa: Approaches, achievements, challenges and lessons. Proceedings of the Nutrition Society. 2019 Nov;78(4):567-75. | Not within a health care setting |
| 1366 | OMeeghan R. Change the Hospital Culture-Make Donation Routine. Transplantation. 2018 Jul 1;102:S807. | Conference abstract |
| 1367 | Ooi CY, Ng CJ, Sales AE, Lim HM. Implementation Strategies for Web-Based Apps for Screening: Scoping Review. Journal of medical Internet research. 2020;22(7):e15591. | Letters to the editor/ Review studies |
| 1368 | Opipari-Arrigan L, Kouril M, Connor J, Griffin N, Stark LJ. Co-producing improved nutrition and growth in children with cf: using continuous improvement to learn how to implement a personalized digital health intervention in clinical care. Pediatric pulmonology.2019; (54):S93-S95. | Conference abstract |
| 1369 | Orchard J, Li J, Gallagher R, Freedman B, Lowres N, Neubeck L. Uptake of a primary care atrial fibrillation screening program (AF-SMART): a realist evaluation of implementation in metropolitan and rural general practice. BMC family practice. 2019 Dec;20(1):1-3. | Conference abstract |
| 1370 | Orchard J, Li J, Gallagher R, Freedman B, Lowres N, Neubeck L. Uptake of a primary care atrial fibrillation screening program (AF-SMART): a realist evaluation of implementation in metropolitan and rural general practice. BMC family practice. 2019 Dec;20(1):1-3. | Study not about effectiveness |
| 1371 | Orchard J, Lowres N, Freedman SB, Ladak L, Lee W, Zwar N, Peiris D, Kamaladasa Y, Li J, Neubeck L. Screening for atrial fibrillation during influenza vaccinations by primary care nurses using a smartphone electrocardiograph (iECG): a feasibility study. European journal of preventive cardiology. 2016 Oct 1;23(2_suppl):13-20. | Study not about effectiveness |
| 1372 | OReilly OJ. Virtual Heart Failure Clinic-An Integrated Care Programme Support for General Practice. International Journal of Integrated Care (IJIC). 2018 Oct 2;18. | Conference abstract |
| 1373 | Orgill M, Gilson L, Chitha W, Michel J, Erasmus E, Marchal B, Harris B. A qualitative study of the dissemination and diffusion of innovations: bottom up experiences of senior managers in three health districts in South Africa. International journal for equity in health. 2019 Dec;18(1):1-5. | Study not about effectiveness |
| 1374 | Orto V, Hendrix CC, Griffith B, Shaikewitz ST. Implementation of a smart pump champions program to decrease potential patient harm. Journal of nursing care quality. 2015 Apr 1;30(2):138-43. | Study not about effectiveness |
| 1375 | Oseji M, Ogu R, Onwumah U. P312 Community‐based interventions for the reduction of maternal mortality–the role of professional health associations, non‐governmental organisations and community‐based organisations in Delta State, Nigeria. International Journal of Gynecology & Obstetrics. 2009 Oct;107:S502-. | Conference abstract |
| 1376 | O'Shaughnessy J. CNE SERIES. Early Sepsis Identification. MedSurg Nursing. 2017 Jul 1;26(4). | Study not about effectiveness |
| 1377 | Osman A.; Panjwani D.; Robinson S. Creating a medication safety culture in picu. Archives of Disease in Childhood. 2015;100(SUPPL. 3):A227-A228 | Conference abstract |
| 1378 | Osmond MH, Gazarian M, Henry RL, Clifford TJ, Tetzlaff J, PERC Spacer Study Group. Barriers to metered‐dose inhaler/spacer use in Canadian pediatric emergency departments: a national survey. Academic Emergency Medicine. 2007 Nov;14(11):1106-13. | Study not about effectiveness |
| 1379 | O'Sullivan PS, Yuan P, Satre DD, Wamsley M, Satterfield J. A sequential implementation model for workforce development: A case study of medical residency training for substance use concerns. Teaching and learning in medicine. 2018 Jan 2;30(1):84-94. | Study not about effectiveness |
| 1380 | O'Toole JK, Starmer AJ, Calaman S, Campos ML, Hepps J, Lopreiato JO, Patel SJ, Rosenbluth G, Schnipper JL, Sectish TC, Srivastava R. I-PASS mentored implementation handoff curriculum: champion training materials. MedEdPORTAL. 2019 Jan 10;15. | Study not about effectiveness |
| 1381 | Ouslander JG. Quality improvement initiatives for urinary incontinence in nursing homes. Journal of the American Medical Directors Association ;8(SUPP):S6-S11 | Study not about effectiveness |
| 1382 | Overbeck G, Davidsen AS, Kousgaard MB. Enablers and barriers to implementing collaborative care for anxiety and depression: a systematic qualitative review. Implementation Science. 2016 Dec;11(1):1-6. | Letters to the editor/ Review studies |
| 1383 | Owens MG, Coogle C, Gentili A, Marrs S, Slattum P, Parsons P, Waters L, Ansello E. Evidence-Based Falls Prevention Training at Hunter Holmes McGuire Veterans Medical Center. InJOURNAL OF THE AMERICAN GERIATRICS SOCIETY 2019 Apr 1; 67:S5-S6. | Conference abstract |
| 1384 | Pace L, Krier C. Shifting us global health policy to provide international development assistance for cancer control in low-and middle-income countries: 591. Asia-pacific Journal of Clinical Oncology. 2014 Dec;10. | Conference abstract |
| 1385 | Padala K, Padala P, Nabholz L, Jackson B, Taylor T, Sullivan D. Home-Based Activity Promotion for Rural Older Veterans via Telehealth: Geriatric Tele-Walking Clinic: B166. Journal of the American Geriatrics Society. 2015 May;63. | Conference abstract |
| 1386 | Pagan M, Harvey P. Implementing a pilot skin and wound care programme in two residential aged care facilities. Wound Practice & Research: Journal of the Australian Wound Management Association. 2019 Dec;27(4):184-92. | Study not about effectiveness |
| 1387 | Page CP, Reid A, Coe CL, Carlough M, Rosenbaum D, Beste J, Fagan B, Steinbacher E, Jones G, Newton WP. Learnings from the pilot implementation of mobile medical milestones application. Journal of graduate medical education. 2016 Oct;8(4):569-75. | Study not about effectiveness |
| 1388 | Page E, Allen R, Wensley F, Rayman G. Improving the peri‐operative pathway of people with diabetes undergoing elective surgery: the IP3D project. Diabetic Medicine. 2020 Dec;37(12):2019-26. | Study not about effectiveness |
| 1389 | Page K, Punch M, Willis J, Rolley J, Daws K, Posenelli S, Winter M, MacIssac A. Strengthening Aboriginal Cardiac Care at St Vincent's Hospital Melbourne—An Integrated Approach. Heart, Lung and Circulation. 2011 Jan 1;20:S8. | Conference abstract |
| 1390 | Pai M, Lloyd NS, Cheng J, Thabane L, Spencer FA, Cook DJ, Haynes RB, Schünemann HJ, Douketis JD. Strategies to enhance venous thromboprophylaxis in hospitalized medical patients (SENTRY): a pilot cluster randomized trial. Implementation Science. 2013 Dec;8(1):1-1. | Study not about effectiveness |
| 1391 | Paladino J, Kavanagh J, Sanders J, Fromme E. Driving Organizational Change in Serious Illness Communication: Successes and Challenges of Implementing the Serious Illness Care Program (QI729). Journal of Pain and Symptom Management. 2019 Feb 1;57(2):471-2. | Conference abstract |
| 1392 | Palka D, Portelli D, Gaudreau M, Levy M, Boss R, Carino G, Palmisciano A. Improving Survival In Patients With Severe Sepsis And Septic Shock: Using Nurse-Physician Dyads, Evidence Based Education And Positive Feedback To Impact Compliance With Early Antibiotics. InC94. FROM BAY TO BREAKING INTENSIVE CARE UNIT CAPACITY: STAFFING, STRAINS AND STEMMING THE TIDE 2012 May (pp. A5078-A5078). American Thoracic Society. | Conference abstract |
| 1393 | Pallas J. The Acute Incident Response program: a framework guiding multidisciplinary responses to acutely traumatic or stress-inducing incidents in the ED setting. Journal of Emergency Nursing. 2020 Sep 1;46(5):579-89. | Study not about effectiveness |
| 1394 | Palm HC, Degnan JH, Biefeld SD, Reese AL, Espey E, Hofler LG. An initiative to implement immediate postpartum long-acting reversible contraception in rural New Mexico. American journal of obstetrics and gynecology. 2020 Apr 1;222(4):S911-e1. | Study not about effectiveness |
| 1395 | Palmer JA, Parker VA, Mor V, Volandes AE, Barre LR, Belanger E, Carter P, Loomer L, McCreedy E, Mitchell SL. Barriers and facilitators to implementing a pragmatic trial to improve advance care planning in the nursing home setting. BMC health services research. 2019 Dec;19(1):1-2. | Study not about effectiveness |
| 1396 | Palubiak S.; Lloyd-Puryear M.; Therrell B.; Kak L. Survive and Thrive: A global alliance Orvosi Hetilap. 2012;153(SUPPL. 3):26 | Conference abstract |
| 1397 | Palumbo R. Reforming penitentiary health. The transition from ‘cure’to ‘care’in Italian prisons. International Journal of Healthcare Management. 2015 Nov 1;8(4):232-43. | Not within a health care setting |
| 1398 | Pannucci CJ, Jaber RM, Zumsteg JM, Golgotiu V, Spratke LM, Wilkins EG. Changing practice: implementation of a venous thromboembolism prophylaxis protocol at an academic medical center. Plastic and reconstructive surgery. 2011 Nov;128(5):1085. | Study not about effectiveness |
| 1399 | Paone D. Implementation of evidence-based practice in 2 states: Lessons from the Chronic Disease Self-Management Program. | Study not about effectiveness |
| 1400 | Paone D. Implementation of evidence-based practice in 2 states: Lessons from the Chronic Disease Self-Management Program. | Duplicate |
| 1401 | Papadakis S, Gharib M, Hambleton J, Reid RD, Assi R, Pipe AL. Delivering evidence-based smoking cessation treatment in primary care practice: experience of Ontario family health teams. Canadian Family Physician. 2014 Jul 1;60(7):e362-71. | Duplicate |
| 1402 | Papadopoulou C, Sime C, Rooney K, Kotronoulas G. Sexual health care provision in cancer nursing care: A systematic review on the state of evidence and deriving international competencies chart for cancer nurses. International journal of nursing studies. 2019 Dec 1;100:103405. | Letters to the editor/ Review studies |
| 1403 | Pappas JM, Flaherty KE, Wooldridge B. Tapping into hospital champions-strategic middle managers. Health care management review. 2004 Jan 1;29(1):8-16. | Not a champion |
| 1404 | Paradise RK, Hatch M, Quessa A, Gargano F, Khaliif M, Costa V. Reducing the use of ad hoc interpreters at a safety-net health care system. The Joint Commission Journal on Quality and Patient Safety. 2019 Jun 1;45(6):397-405. | Study not about effectiveness |
| 1405 | Parand A, Burnett S, Benn J, Iskander S, Pinto A, Vincent C. Medical engagement in organisation-wide safety and quality-improvement programmes: experience in the UK Safer Patients Initiative. Quality and Safety in Health care. 2010 Oct 1;19(5):e44-. | Study not about effectiveness |
| 1406 | Parish E, Runnacles J, Davey N, Roueché A. 102 Developing effective leaders: creating change champions in children’s healthcare. | Conference abstract |
| 1407 | Park HW. Improving quality of healthcare in Korea. Journal of the Korean Medical Association. 2012 Oct 1;55(10):969-77. | Not English |
| 1408 | Parker CN, Shuter P, Maresco‐Pennisi D, Sargent J, Collins L, Edwards HE, Finlayson KJ. Implementation of the Champions for Skin Integrity model to improve leg and foot ulcer care in the primary healthcare setting. Journal of clinical nursing. 2019 Jul;28(13-14):2517-25. | Study not about effectiveness |
| 1409 | Parker V, Giles M, Graham L, Suthers B, Watts W, O’Brien T, Searles A. Avoiding inappropriate urinary catheter use and catheter-associated urinary tract infection (CAUTI): a pre-post control intervention study. BMC health services research. 2017 Dec;17(1):1-9. | Protocol |
| 1410 | Parkerton PH, Needleman J, Pearson ML, Upenieks VV, Soban LM, Yee T. Lessons from nursing leaders on implementing TCAB. AJN The American Journal of Nursing. 2009 Nov 1;109(11):71-6. | Study not about effectiveness |
| 1411 | Parkosewich J, Funk M, Bradley EH. Applying five key success factors to optimize the quality of care for patients hospitalized with coronary artery disease. Progress in cardiovascular nursing. 2005 Jun;20(3):111-6. | Study not about effectiveness |
| 1412 | Parris S, Cochrane G, Marjanovic S, Ling T, Chataway J. Galvanising the nhs to adopt innovation: the feasibility and practicality of recommendations from the interim report of the accelerated access review. Rand health quarterly. 2016 Jun 20;6(1). | Study not about effectiveness |
| 1413 | Pasio KS, Mash R, Naledi T. Development of a family physician impact assessment tool in the district health system of the Western Cape Province, South Africa. BMC family practice. 2014 Dec;15(1):1-9. | Not about knowledge translation/evidence-based practice |
| 1414 | Patel B, Usherwood T, Harris M, Panaretto K, Redfern J, Jansen J, McKinn S, Lyford M, Patel A, Peiris D. PT253 Understanding the impact of a multifaceted quality improvement intervention to improve cardiovascular disease risk management in Australian Primary Health Care: The TORPEDO study process evaluation. Global Heart. 2014;1(9):e214-5. | Conference abstract |
| 1415 | Pathania S, Slater LZ, Vose C, Navarra AM. Music therapy and pain management in patients with end-stage liver disease: An evidence-based practice quality improvement project. Pain Management Nursing. 2019 Feb 1;20(1):10-6. | Study not about effectiveness |
| 1416 | Patterson J.A.; Olson B.H.; Keuler N.S. Improving the culture of breastfeeding support in primary care practices: A pilot of the outpatient breastfeeding champion program. Breastfeeding Medicine. 2018;13(7):A-31 | Conference abstract |
| 1417 | Patterson JA, Keuler NS, Eglash AR, Olson BH. Outpatient breastfeeding champion program: Breastfeeding support in primary care. Breastfeeding Medicine. 2020 Jan 1;15(1):44-8. | Study not about effectiveness |
| 1418 | Patterson PD, Anderson MS, Zionts ND, Paris PM. The emergency medical services safety champions. American journal of medical quality. 2013 Jul;28(4):286-91. | Not about knowledge translation/evidence-based practice |
| 1419 | Patwardhan MB, Matchar DB, Samsa GP, Haley WE. Utility of the advanced chronic kidney disease patient management tools: case studies. American Journal of Medical Quality. 2008 Mar;23(2):105-14. | Study not about effectiveness |
| 1420 | Paulsen MM, Varsi C, Paur I, Tangvik RJ, Andersen LF. Barriers and facilitators for implementing a decision support system to prevent and treat disease-related malnutrition in a hospital setting: qualitative study. JMIR formative research. 2019 May 9;3(2):e11890. | Study not about effectiveness |
| 1421 | Paxton EW, Kiley ML, Love R, Barber TC, Funahashi TT, Inacio MC. Kaiser Permanente implant registries benefit patient safety, quality improvement, cost-effectiveness. The Joint Commission Journal on Quality and Patient Safety. 2013 Jun 1;39(6):246-AP4. | Study not about effectiveness |
| 1422 | Payne JI, Dunbar MJ, Talbot P, Tan MH. Diabetes Care Program of Nova Scotia: Celebrating 25 Years of Improving Diabetes Care in Nova Scotia. Canadian journal of diabetes. 2018 Jun 1;42(3):317-24. | Study not about effectiveness |
| 1423 | Peace D. A Multi-disciplinary approach to improving environmental hygiene in a pediatric system American Journal of Infection Control. 2010;38(5):E100-E10 | Conference abstract |
| 1424 | Peace D. Pediatric Environmental Infection Prevention-Infancy to Adolescence. American Journal of Infection Control. 2011 Jun 1;39(5):E24-5. | Conference abstract |
| 1425 | Pearson D, Heffner A, Gabbard E, Garvey L, Wares C, Karvetski C, Runyon M. Impact of a Standardized Post-Arrest Clinical Pathway and Quality Improvement Tool on Three Receiving Cardiac Resuscitation Centers Within a Single Healthcare System: 604. Academic Emergency Medicine. 2016 May;23. | Conference abstract |
| 1426 | Pease S. integrating care to improve surgical outcomes and reduce costs. Healthcare Financial Management. 2018 Aug 1;72(8):32-7. | Study not about effectiveness |
| 1427 | Pek JH, Kang HM, Wong E. Improving apnoeic oxygenation use for rapid sequence intubation in an emergency department. Trends in Anaesthesia and Critical Care. 2017 Apr 1;13:25-31. | Study not about effectiveness |
| 1428 | Pekkala J, Cross‐Barnet C, Kirkegaard M, Silow‐Carroll S, Courtot B, Hill I. Key considerations for implementing group prenatal care: lessons from 60 practices. Journal of midwifery & women's health. 2020 Mar;65(2):208-15. | Study not about effectiveness |
| 1429 | Peleshok J.; Yamada J.; Krancevic A.-M.; McNair C.; Nanji M.; Leblanc A.; Hanley J.; Stevens B.; Campbell F. Linking customized knowledge translation initiatives to pediatric pain outcomes. Pain Research and Management / 2014;19(3):e90-e91 | Conference abstract |
| 1430 | Peritore NR. Communicating social support: Understanding complexities of breastfeeding communication among African American mothers. | Not about knowledge translation/evidence-based practice |
| 1431 | Perkins A, Nicholls K, Shaw T, Liu G, Molokhia E. Attitudes toward colorectal cancer screening in the digital age: a survey of practices and attitudes among screening-eligible Alabamians. Southern medical journal. 2013 Aug 1;106(8):462-7. | Study not about effectiveness |
| 1432 | Perlin JB, Hickok JD, Septimus EJ, Moody JA, Englebright JD, Bracken RM. A bundled approach to reduce methicillin‐resistant Staphylococcus aureus infections in a system of community hospitals. Journal for Healthcare Quality. 2013 May;35(3):57-69. | Study not about effectiveness |
| 1433 | Pernigotti D, Rosenblum D, Belliveau T, Zazula J, Houlihan BV, Seetharama S, Jette A. Development of the New England Spinal Cord Injury Toolkit for Peer-to-Peer Clinical Education. Archives of Physical Medicine and Rehabilitation. 2015 Oct 1;96(10):e41-2. | Conference abstract |
| 1434 | Pesut B, Duggleby W, Warner G, Kervin E, Bruce P, Antifeau E, Hooper B. Implementing volunteer-navigation for older persons with advanced chronic illness (Nav-CARE): a knowledge to action study. BMC Palliative Care. 2020 Dec;19:1-6. | Study not about effectiveness |
| 1435 | Peterson JC, Rogers EM, Cunningham-Sabo L, Davis SM. A framework for research utilization applied to seven case studies. American journal of preventive medicine. 2007 Jul 1;33(1):S21-34. | Not within a health care setting |
| 1436 | Peterson KA, Radosevich DM, O'Connor PJ, Nyman JA, Prineas RJ, Smith SA, Arneson TJ, Corbett VA, Weinhandl JC, Lange CJ, Hannan PJ. Improving diabetes care in practice: findings from the TRANSLATE trial. Diabetes care. 2008 Dec 1;31(12):2238-43. | Study not about effectiveness |
| 1437 | Peterson KA, Radosevich DM, O'Connor PJ, Nyman JA, Prineas RJ, Smith SA, Arneson TJ, Corbett VA, Weinhandl JC, Lange CJ, Hannan PJ. Improving diabetes care in practice: findings from the TRANSLATE trial. Diabetes care. 2008 Dec 1;31(12):2238-43. | Study not about effectiveness |
| 1438 | Petrescu-Prahova M, Belza B, Kohn M, Miyawaki C. Implementation and maintenance of a community-based older adult physical activity program. The Gerontologist. 2016 Aug 1;56(4):677-86. | Not within a health care setting |
| 1439 | Petrides AK, Tanasijevic MJ, Goonan EM, Landman AB, Kantartjis M, Bates DW, Melanson SE. Top ten challenges when interfacing a laboratory information system to an electronic health record: Experience at a large academic medical center. International journal of medical informatics. 2017 Oct 1;106:9-16. | Study not about effectiveness |
| 1440 | Phillips BO, Duffrin C. Ohio Osteopathic Network of Excellence: establishing a statewide telehealth consortium. Journal of Osteopathic Medicine. 2001 Dec 1;101(12):720-2. | Study not about effectiveness |
| 1441 | Phillips G, Ker J. Champion students! Experience with a standardized infection control training package in medical students. Journal of Hospital Infection. 2006 Apr 1;62(4):518-9. | Letters to the editor/ Review studies |
| 1442 | Phillips SJ, Stevens A, Cao H, Simpkin W, Payne J, Gill N. Improving stroke care in Nova Scotia, Canada: a population-based project spanning 14 years. BMJ Open Quality. 2021 Sep 1;10(3):e001368. | Conference abstract |
| 1443 | Phillips‐Angeles E, Song L, Hannon PA, Celedonia M, Stearns S, Edwards K, Feest S, Shumann A. Fostering partnerships and program success. Cancer. 2013 Aug 1;119:2884-93. | Study not about effectiveness |
| 1444 | Pignatiello A, Teshima J, Boydell KM, Minden D, Volpe T, Braunberger PG. Child and youth telepsychiatry in rural and remote primary care. Child and Adolescent Psychiatric Clinics. 2011 Jan 1;20(1):13-28. | Study not about effectiveness |
| 1445 | Pigot M, Miller CE, Brockman R, Grenyer BF. Barriers and facilitators to the implementation of a stepped care intervention for personality disorder in mental health services. Personality and mental health. 2019 Nov;13(4):230-8. | Study not about effectiveness |
| 1446 | Pilling LB, Bogen J, Simmons R. Building Interprofessional Global Health Infrastructure at a University and Health System: Navigating Challenges and Scaling Successes. Annals of Global Health. 2017 Apr 7;83(1). | Conference abstract |
| 1447 | Pinderup P. Improving the knowledge, attitudes, and practices of mental health professionals regarding dual diagnosis treatment–a mixed methods study of an intervention. Issues in mental health nursing. 2018 Apr 3;39(4):292-303. | Study not about effectiveness |
| 1448 | Pinto C, Bristowe K, Witt J, Davies JM, de Wolf-Linder S, Dawkins M, Guo P, Higginson IJ, Daveson B, Murtagh FE. Perspectives of patients, family caregivers and health professionals on the use of outcome measures in palliative care and lessons for implementation: a multi-method qualitative study. Annals of palliative medicine. 2018 Oct 1;7:S137-50. | Study not about effectiveness |
| 1449 | Piscotty RJ, Tzeng HM. Exploring the clinical information system implementation readiness activities to support nursing in hospital settings. CIN: Computers, Informatics, Nursing. 2011 Nov 1;29(11):648-56. | Study not about effectiveness |
| 1450 | Piver-Renna JM. The adoption and implementation of alcohol screening, brief intervention, and referral to treatment programs in mid-Atlantic Level I trauma centers. The Johns Hopkins University; 2009. | Study not about effectiveness |
| 1451 | Planas LG, Desselle SP, Cao K. Valuable Lessons for Pharmacist PBRNs: Insights and Experiences from Physician PBRN Members. Pharmacy. 2019 Sep;7(3):123. | Study not about effectiveness |
| 1452 | Ploeg J, Davies B, Edwards N, Gifford W, Miller PE. Factors influencing best‐practice guideline implementation: Lessons learned from administrators, nursing staff, and project leaders. Worldviews on Evidence‐Based Nursing. 2007 Dec;4(4):210-9. | Study not about effectiveness |
| 1453 | Ploeg J, Markle-Reid M, Davies B, Higuchi K, Gifford W, Bajnok I, McConnell H, Plenderleith J, Foster S, Bookey-Bassett S. Spreading and sustaining best practices for home care of older adults: a grounded theory study. Implementation Science. 2014 Dec;9(1):1-7. | Study not about effectiveness |
| 1454 | Ploeg J, Skelly J, Rowan M, Edwards N, Davies B, Grinspun D, Bajnok I, Downey A. The role of nursing best practice champions in diffusing practice guidelines: a mixed methods study. Worldviews on Evidence‐Based Nursing. 2010 Dec;7(4):238-51. | Study not about effectiveness |
| 1455 | Plunkett A, Kelly N, Scott D. G549 (P) Learning from excellence: a new paradigm of safety reporting. 2015 | Conference abstract |
| 1456 | Polaha J, Smith JD, Sunderji N. A recipe for assessing fidelity in family and health systems. 2019 | Letters to the editor/ Review studies |
| 1457 | Poland B, Graham H, Walsh E, Williams P, Fell L, Lum JM, Polzer J, Syed S, Tobin S, Kim G, Yardy G. ‘Working at the margins’ or ‘leading from behind’?: a Canadian study of hospital− community collaboration. Health & social care in the community. 2005 Mar;13(2):125-35. | Study not about effectiveness |
| 1458 | Polihronis C, Cloutier PF, Cappelli M, Gray C, Zemek R, Kennedy A, Reid S, Gardner W, Pajer KA, Jabbour M. Barriers and Enablers of Implementing an Emergency Department Mental Health Service Pathway. In66th Annual Meeting 2019 Oct 16. AACAP. | Conference abstract |
| 1459 | Pollard J, Oliver-McNeil S, Patel S, Baker H, Mason L, Melia A, Hummel SL. Regional hospital collaboration is associated with reduced 30-day readmission in medicare heart failure patients. Journal of Cardiac Failure. 2014 Aug 1;20(8):S55. | Conference abstract |
| 1460 | Pomare C, Churruca K, Long JC, Ellis LA, Braithwaite J. Organisational change in hospitals: a qualitative case-study of staff perspectives. BMC health services research. 2019 Dec;19(1):1-9. | Study not about effectiveness |
| 1461 | Ponte AH. Paths to Tier 1 Genomics Implementation: A Survey of Chronic Disease Directors (Doctoral dissertation, Walden University). | Study not about effectiveness |
| 1462 | Pop H, Lamb K, Livesay S, Altman P, Sanchez A, Nora ME. Tailoring a comprehensive bundled intervention for ED fall prevention. Journal of emergency nursing. 2020 Mar 1;46(2):225-32. | Study not about effectiveness |
| 1463 | Poremski D.; Kahan D.; Pauly D.; Stergiopoulos V. Challenges in continuity of care: The experience of frequent users of emergency departments in a large metropolitan center in Canada. Annals of the Academy of Medicine Singapore. 2015;44(10 SUPPL. 1):S227 | Conference abstract |
| 1464 | Porter SC, Johnston P, Parry G, Damian F, Hoppa EC, Stack AM. Improving parent-provider communication in the pediatric emergency department: results from the clear and concise communication campaign. Pediatric emergency care. 2011 Feb 1;27(2):75-80. | Study not about effectiveness |
| 1465 | Portnoy B, Lee SJ, Kincheloe J, Breen N, Olson JL, McCormally J, Brown ER. Independent state health surveys: responding to the need for local population health data. Journal of public health management and practice: JPHMP. 2014 Sep;20(5):E21. | Study not about effectiveness |
| 1466 | Postema TR, Peeters JM, Friele RD. Key factors influencing the implementation success of a home telecare application. International journal of medical informatics. 2012 Jun 1;81(6):415-23. | Study not about effectiveness |
| 1467 | Poulos RG, Lord SR, Zwi AB. Towards enhancing national capacity for evidence informed policy and practice in falls management: a role for a" Translation Task Group"?. Australia and New Zealand Health Policy. 2007;4(1). | Study not about effectiveness |
| 1468 | Powers MA, Cuddihy RM, Bergenstal RM, Tompos P, Pearson J, Morgan B. Improving blood pressure control in individuals with diabetes: a quality improvement collaborative. The Joint Commission Journal on Quality and Patient Safety. 2011 Mar 1;37(3):110-AP1. | Protocol |
| 1469 | Powers, M. How to create the best appeal strategy for coding denials. Healthcare Financial Management 2020;74(3):30-31 | Not about knowledge translation/evidence-based practice |
| 1470 | Pradarelli JC, Yule S, Panda N, Craig M, Lowery KW, Ashley SW, Gee DW, Waters PM, Knight J, Smink DS. Optimizing the implementation of surgical coaching through feedback from practicing surgeons. JAMA surgery. 2021 Jan 1;156(1):42-9. | Study not about effectiveness |
| 1471 | Prasad S, Cohen H, Adelson K, Abdelghany O. Developing a pharmacoeconomics program in an academic medical center. Value in health. 2017;20 (5):132-A132. | Conference abstract |
| 1472 | Prata N, Gessessew A, Abraha AK, Holston M, Potts M. Prevention of postpartum hemorrhage: options for home births in rural Ethiopia. African Journal of Reproductive Health. 2009;13(2). | Study not about effectiveness |
| 1473 | Preas MA, Custer M, Rew C, Hebden J, Thom K, Cafeo C. Economic Impact of a Dedicated Nurse Champion in Reducing Catheter Associated Bloodstream Infections (CLABSIs). American Journal of Infection Control. 2011 Jun 1;39(5):E207. | Conference abstract |
| 1474 | Preas MA, Emerick M, Harris-Williams M, Filippell M, Hebden J, Thom K, Harris A, Leekha S. Culture Change and CLABSI Reduction: Achieving Success in a Medical Center with 10 Distinctively Different Intensive Care Units. American Journal of Infection Control. 2012 Jun 1;40(5):e194-5. | Conference abstract |
| 1475 | Prebble K, Kidd J, O'Brien A, Carlyle D, McKenna B, Crowe M, Deering D, Gooder C. Implementing and maintaining nurse-led healthy living programs in forensic inpatient settings: an illustrative case study. Journal of the American Psychiatric Nurses Association. 2011 Mar;17(2):127-38. | Study not about effectiveness |
| 1476 | Prendergast M, Honey M. The barriers and facilitators for nurse educators using telehealth for education. InMEDINFO 2019: Health and Wellbeing e-Networks for All 2019 (pp. 1323-1326). IOS Press. | Study not about effectiveness |
| 1477 | Prescribing Technology to Increase Uptake of Depression Treatment in Primary Care: A Pre-implementation Focus Group Study of SOVA (Supporting Our Valued Adolescents) | Study not about effectiveness |
| 1478 | Preshaw J, Evans K, Attilia B. Revolutionising sepsis management within maternity at Musgrove Park Hospital, Taunton. BJOG: An International Journal of Obstetrics and Gynaecology / 2016;123(Supplement 4):5 | Conference abstract |
| 1479 | Preslaski CR, Lat I, MacLaren R, Poston J. Pharmacist contributions as members of the multidisciplinary ICU team. Chest. 2013 Nov 1;144(5):1687-95. | Study not about effectiveness |
| 1480 | Price K, Kennedy KJ, Rando TL, Dyer AR, Boylan J. Education and process change to improve skin health in a residential aged care facility. International wound journal. 2017 Dec;14(6):1140-7. | Study not about effectiveness |
| 1481 | Priest KC, Englander H, McCarty D. “Now hospital leaders are paying attention”: a qualitative study of internal and external factors influencing addiction consult services. Journal of substance abuse treatment. 2020 Mar 1;110:59-65. | Study not about effectiveness |
| 1482 | Proehl JA, Hoyt KS. Clinical inertia and champions for change. Advanced emergency nursing journal. 2014 Jul 1;36(3):207-8. | Letters to the editor/ Review studies |
| 1483 | Pronovost P, Weast B, Rosenstein B, Sexton JB, Holzmueller CG, Paine L, Davis R, Rubin HR. Implementing and validating a comprehensive unit-based safety program. Journal of Patient Safety. 2005 Mar 1;1(1):33-40. | Study not about effectiveness |
| 1484 | Provaznik J, Ahmad M. Early identification of delirium in elderly hospitalized patients. Journal of general internal medicine. 2018;33: S173-S173. | Conference abstract |
| 1485 | Pruden J. PICU's Mission to Eliminate CA-BSI by Joining a National Collaborative. American Journal of Infection Control. 2011 Jun 1;39(5):E156. | Conference abstract |
| 1486 | Puleo E, Zapka J, White MJ, Mouchawar J, Somkin C, Taplin S. Caffeine, cajoling, and other strategies to maximize clinician survey response rates. Evaluation & the health professions. 2002 Jun;25(2):169-84. | Study not about effectiveness |
| 1487 | Pulia M, Redwood R, May L. Antimicrobial stewardship in the emergency department. Emergency Medicine Clinics. 2018 Nov 1;36(4):853-72. | Study not about effectiveness |
| 1488 | Purvis T, Marion V, Andrew NE, Kilkenny MF, Breen S, Cadilhac DA. Reducing clinical variation: process evaluation of the Shared Team Efforts Leading to Adherence Results (STELAR) project. International Journal of Stroke 2019 Aug 1;14: 8-8. | Conference abstract |
| 1489 | Qamruddin A, Duran G, Stefanescu A, Boehm E, Parashette V, Stefanescu B. NEWBORN INTERVENTION PROMOTING PRETERM INFANT EATING READINESS: 415. Journal of Investigative Medicine. 2019 Jan;67(1). | No full text available |
| 1490 | Qu H, Hu X, Singh JA. Factors influencing implementation of a computerized, individualized, culturally tailored lupus decision aid in lupus clinics: a qualitative semi-structured interview study. Clinical rheumatology. 2019 Oct;38(10):2793-801. | Study not about effectiveness |
| 1491 | Quicke JG, Cottrell E, Duffy H, Somerville S, Oxtoby J, Wallbanks A, Campbell L, Blackburn S, Stevenson K, Cooper V, Finney A. Implementing and evaluating a pilot physiotherapist-led osteoarthritis clinic in general practice. Physiotherapy. 2019 Jan 1;105:e33-4. | Conference abstract |
| 1492 | Quinn KG, Christenson E, Spector A, Amirkhanian Y, Kelly JA. The influence of peers on PrEP perceptions and use among young black gay, bisexual, and other men who have sex with men: a qualitative examination. Archives of sexual behavior. 2020 Feb 3:1-5. | Study not about effectiveness |
| 1493 | Quraishi F, Cheung V, Sharp S, Avinoam G. Collaboratively moving knowledge into practice: AP. 99. International Journal of Stroke. 2015 Sep;10. | Conference abstract |
| 1494 | Raab S, Currens H, Woodman D. IMPLEMENTATION OF LEAN STRATEGIES IN CYTOPATHOLOGY: S3-019. Cytopathology. 2011 Oct;22. | Conference abstract |
| 1495 | Raff E, Campbell C, Wei E. Added value: Quality improvement in radiology Emergency Radiology. 2016;23(6):571 | Conference abstract |
| 1496 | Rafferty MR, MacDonald J, Byskosh A, Sloan L, Toledo S, Marciniak C, Simuni T. Using implementation frameworks to provide proactive physical therapy for people with Parkinson disease: case report. Physical therapy. 2019 Dec 16;99(12):1644-55. | Study not about effectiveness |
| 1497 | Rafie C, Ayers A, Cadet D, Quillin J, Hackney MH. Reaching hard to reach populations with hard to communicate messages: efficacy of a breast health research champion training program. Journal of Cancer Education. 2015 Sep;30(3):599-606. | Study not about effectiveness |
| 1498 | Ragazzi,​ H.,​ Keller,​ A.,​ Ehrensberger,​ R.,​ Irani,​ A.-M. (2011). Evaluation of a practice-based intervention to Ragazzi H, Keller A, Ehrensberger R, Irani AM. Evaluation of a practice-based intervention to improve the management of pediatric asthma. Journal of Urban Health. 2011 Feb 1;88(1):38-48. | Study not about effectiveness |
| 1499 | Rajaraman M, Urquhart R, Kendell C, Geldenhuys L, Ross A, Folkes A, Sullivan V, Rayson D, Porter GA. 218 How Scientific Evidence is Used to Adopt Complex Innovations in Cancer Care: A Multiple-Case Study from Nova Scotia, Canada. Radiotherapy and Oncology. 2019 Oct 1;139:S91. | Conference abstract |
| 1500 | Rana AJ. Building a Patient-Reported Outcome Metric Database: One Hospital's Experience. The Journal of arthroplasty. 2016 Jun 1;31(6):1151-4. | Study not about effectiveness |
| 1501 | Rand CM, Concannon C, Wallace-Brodeur R, Davis W, Albertin CS, Humiston SG, Szilagyi PG. Identifying Strategies to Reduce Missed Opportunities for HPV Vaccination in Primary Care: A Qualitative Study of Positive Deviants. Clinical pediatrics. 2020 Oct;59(12):1058-68. | Study not about effectiveness |
| 1502 | Rand CM, Concannon C, Wallace-Brodeur R, Davis W, Albertin CS, Humiston SG, Szilagyi PG. Identifying Strategies to Reduce Missed Opportunities for HPV Vaccination in Primary Care: A Qualitative Study of Positive Deviants. Clinical pediatrics. 2020 Oct;59(12):1058-68. | Study not about effectiveness |
| 1503 | Randall, A, Lieberthal, B. One size does not fit all. Health Management Technology 2012;33(10):26-7 | Letters to the editor/ Review studies |
| 1504 | Randell TV, Madeira T, Pavlovic NV, Abshire M. Mechanical Circulatory Support Driveline Infection Reduction through Nursing Quality Improvement. Journal of Cardiac Failure. 2019 Aug 1;25(8):S123. | Conference abstract |
| 1505 | Rangachari P, Dellsperger KC, Rethemeyer RK. Network analysis of the structure of inter-professional knowledge exchange related to Electronic Health Record Medication Reconciliation within a Social Knowledge Networking system. Journal of healthcare leadership. 2019;11:87. | Study not about effectiveness |
| 1506 | Rangachari P, Madaio M, Rethemeyer RK, Wagner P, Hall L, Roy S, Rissing P. The evolution of knowledge exchanges enabling successful practice change in two intensive care units. Health Care Management Review. 2015 Jan 1;40(1):65-78. | Study not about effectiveness |
| 1507 | Rangachari P, Rethemeyer K. Structure of Inter-Professional Knowledge Exchange Related to"" EHR MedRec"" within an SKN System. InAcademy of Management Proceedings 2019 Jul 18 (Vol. 2019, No. 1, p. 10517). Briarcliff Manor, NY 10510: Academy of Management. | Conference abstract |
| 1508 | Rangachari P, Rethemeyer RK. Impact of Periodic Top-down Communications on Infection Prevention Practices & Outcomes in Two Units. InAcademy of Management Proceedings 2014 (Vol. 2014, No. 1, p. 11039). Briarcliff Manor, NY 10510: Academy of Management. | Conference abstract |
| 1509 | Rangan K, Bava L, Bailan C, Carcich S, Green S, Vingelen MB, O’Connell J, Thomas S. Nurs-18. Development of a multidisciplinary care service for adolescent and young adult oncology patients at an urban pediatric academic center. Neuro-oncology. 2018; 20(Suppl 2):i154. | Conference abstract |
| 1510 | Rankin NM, Shaw T, York S, Mcgregor D, Butow P, Young J, White K, Barnes D, Zielinski R, Stone E. Improving Pathways to Early Lung Cancer Diagnosis: Process Mapping and Qualitative Analysis. Journal of thoracic oncology.2015;10 (9):S398-S398. | Conference abstract |
| 1511 | Rantz MJ, Grando V, Conn V, Zwygart-Staffacher M, Hicks L, Flesner M, Scott J, Manion P, Minner D, Porter R, Maas M. Getting the basics right: Care delivery in nursing homes. | Not a champion |
| 1512 | Rantz MJ, Grando V, Conn V, Zwygart-Staffacher M, Hicks L, Flesner M, Scott J, Manion P, Minner D, Porter R, Maas M. Getting the basics right: Care delivery in nursing homes. | Duplicate |
| 1513 | Rantz MJ, Zwygart-Stauffacher M, Flesner M, Hicks L, Mehr D, Russell T, Minner D. Challenges of using quality improvement methods in nursing homes that “need improvement”. Journal of the American Medical Directors Association. 2012 Oct 1;13(8):732-8. | Study not about effectiveness |
| 1514 | Rao SK, Turner SM, Ferris TG. Reducing the burden of quality improvement through the abms maintenance of certification portfolio sponsor program. Journal of general internal medicine. 2017;32: S792-S792. | Conference abstract |
| 1515 | Rasmusson J, Almonte R, Brown A. Educating the Stroke Patient Thus Educating the Community. Stroke 2010;41 (4): E205-E205. | Conference abstract |
| 1516 | Ratnam A, Rudra A, Chatterjee K, Das RC. Psychiatric Advance Directives in India: What will the future hold?. Asian journal of psychiatry. 2015 Aug 1;16:36-40. | Not a champion |
| 1517 | Rattray NA, Damush TM, Miech EJ, Homoya B, Myers LJ, Penney LS, Ferguson J, Giacherio B, Kumar M, Bravata DM. Empowering implementation teams with a learning health system approach: leveraging data to improve quality of care for transient ischemic attack. Journal of general internal medicine. 2020 Nov;35(2):823-31. | Study not about effectiveness |
| 1518 | Rautemaa-Richardson R, Rautemaa V, Al-Wathiqi F, Moore CB, Craig L, Felton TW, Muldoon EG. Impact of a diagnostics-driven antifungal stewardship programme in a UK tertiary referral teaching hospital. Journal of Antimicrobial Chemotherapy. 2018 Dec 1;73(12):3488-95. | Study not about effectiveness |
| 1519 | Raval MV, Bentrem DJ, Eskandari MK, Ingraham AM, Hall BL, Randolph B, Ko CY, Morton JM. The role of surgical champions in the American College of Surgeons National Surgical Quality Improvement Program–a national survey. Journal of Surgical Research. 2011 Mar 1;166(1):e15-25. | Study not about effectiveness |
| 1520 | Rawal R, Adeyemo O, Kunnath P, Saad H, Vartanyan A, Schmidt J. The barrier to mindful lab ordering: The attending. Journal of Hospital Medicine. 2018;13(4 Supplement 1) | Conference abstract |
| 1521 | Ray S, Laur C, Douglas P, Rajput-Ray M, van der Es M, Redmond J, Eden T, Sayegh M, Minns L, Griffin K, McMillan C. Nutrition education and leadership for improved clinical outcomes: training and supporting junior doctors to run ‘Nutrition Awareness Weeks’ in three NHS hospitals across England. BMC medical education. 2014 Dec;14(1):1-1. | Study not about effectiveness |
| 1522 | Rayment M, Rae C, Sullivan A, Skene H, Davies G. Routine HIV testing on an Acute Admissions Unit (AAU) is feasible and affordable, but a challenge to sustain. InHIV MEDICINE 2015 Apr 1 (Vol. 16, pp. 48-48). | Conference abstract |
| 1523 | Rea K, Le-Jenkins U, Rutledge C. A technology intervention for nurses engaged in preventing catheter-associated urinary tract infections. CIN: Computers, Informatics, Nursing. 2018 Jun 1;36(6):305-13. | Study not about effectiveness |
| 1524 | Reddy KK, Samuel A, Smiley KA, Weber S, Hon H. Reducing Central line–associated bloodstream infections in Three ICUs at a tertiary care hospital in the United Arab Emirates. The Joint Commission Journal on Quality and Patient Safety. 2014 Dec 1;40(12):559-AP1. | Study not about effectiveness |
| 1525 | Reddy SM, Rose DE, Burgess Jr JF, Charns MP, Yano EM. The role of organizational factors in the provision of comprehensive women's health in the Veterans Health Administration. Women's Health Issues. 2016 Nov 1;26(6):648-55. | Not a champion |
| 1526 | Reddy,​ Kalpana K.,​ Samuel,​ Asha,​ Smiley,​ Kathleen Ann,​ Weber,​ Stefan,​ Hon,​ Hubert (2014). Reducing Central Line--Associated Bloodstream Infections in Three ICUs at a Tertiary Care Hospital in the United Arab Emirates. *#journal#*,​ 40(12),​ 559 | Study not about effectiveness |
| 1527 | Redman R. A multifaceted, evidence-based program to reduce inappropriate antibiotic treatment of suspected urinary tract infections. Annals of Long-Term Care. 2017 Mar. | Study not about effectiveness |
| 1528 | Reed SM, Brock AJ, Anderson TJ. CE: champions for central line care. AJN The American Journal of Nursing. 2014 Sep 1;114(9):40-8. | Study not about effectiveness |
| 1529 | Reeths A, Krueger R, Lips K, Cohen T, Madden J, Fleming-Hill M, Cash E, Baker K, Konkle D, Miller M, Will J. Our Journey to Eliminate Central Line Associated Blood Stream Infections in our NICU. American Journal of Infection Control. 2012 Jun 1;40(5):e58. | Conference abstract |
| 1530 | Reeves T. Reducing central line infections...Reaching beyond critical care. A quality improvement initiative. Journal of the Association for Vascular Access. 2011;16(4):210 | Conference abstract |
| 1531 | Regan L, Peterson S, Bright L, Omron R, Neira P, Patch M. Partners in Training, Partners in Care: Integrating Nurses in Emergency Medicine Residency Training. Western Journal of Emergency Medicine: Integrating Emergency Care with Population Health. 2016;17(4.1). | Conference abstract |
| 1532 | Regenstein M, Huang J, West C, Trott J, Mead H, Andres E. Improving the quality of language services delivery: findings from a hospital quality improvement initiative. Journal for Healthcare Quality. 2012 Mar;34(2):53-63. | Study not about effectiveness |
| 1533 | Reicherter EA, Gordes KL, Glickman LB, Hakim EW. Creating disseminator champions for evidence-based practice in health professions education: an educational case report. Nurse education today. 2013 Jul 1;33(7):751-6. | Not within a health care setting |
| 1534 | Reidy J, Halvorson J, Makowski S, Katz D, Weinstein B, McCluskey C, Doering A, DeCarli K, Tjia J. Health system advance care planning culture change for high-risk patients: the promise and challenges of engaging providers, patients, and families in systematic advance care planning. Journal of palliative medicine. 2017 Apr 1;20(4):388-94. | Study not about effectiveness |
| 1535 | Reinecke JD, Kelvin JF, Arvey SR, Quinn GP, Levine J, Beck LN, Miller A. Implementing a systematic approach to meeting patients' cancer and fertility needs: a review of the Fertile Hope Centers of Excellence program. Journal of oncology practice. 2012 Sep;8(5):303-8. | Study not about effectiveness |
| 1536 | Reinholdt F, Murray J. Distinction as a Quality Improvement Partnership in a Community Hospital. InSTROKE 2013 Dec 1 (Vol. 44, No. 12, pp. E225-E225). | Conference abstract |
| 1537 | Reisinger JD, Wojcik A, Jenkins I, Edson B, Pegues DA, Greene L. The Project Protect Infection Prevention Fellowship: A model for advancing infection prevention competency, quality improvement, and patient safety. American journal of infection control. 2017 Aug 1;45(8):876-82. | Study not about effectiveness |
| 1538 | Relias Media (2008). "What if physicians don't believe your quality data? Your reaction will determine if you obtain buy-in." Healthcare Benchmarks & Quality Improvement 15(7): 67-69. | Study not about effectiveness |
| 1539 | Reliasmedia. Grooming the next QI physician champion. Hospital Peer Review 11// 2013;38(11):126-127 | Letters to the editor/ Review studies |
| 1540 | ReliasMedia. Nurses become quality, safety investigators. Healthcare Risk Management 2010;(): | Conference abstract |
| 1541 | ReliasMedia.ICP: find a champion for infection prevention: innovative prevention possible in all settings. Hospital Infection Control Jan 2007;34(1):6-6 | Letters to the editor/ Review studies |
| 1542 | Remedios D, Brkljacic B, Ebdon-Jackson S, Hierath M, Sinitsyn V, Vassileva J. Collaboration, campaigns and champions for appropriate imaging: feedback from the Zagreb workshop. Insights into imaging. 2018 Apr;9(2):211-4. | Study not about effectiveness |
| 1543 | Remenyi B, Carapetis J, Wyber R, Taubert K, Mayosi BM. Position statement of the World Heart Federation on the prevention and control of rheumatic heart disease. Nature Reviews Cardiology. 2013 May;10(5):284-92. | Not about knowledge translation/evidence-based practice |
| 1544 | Resnick B, Carrico R, Gravenstein S, Hogue MD, Middleton DB, Rehm S, Schaffner W, Tan L. Dissemination and implementation of the ICAMP. Translational behavioral medicine. 2018 Dec;8(6):867-75. | Study not about effectiveness |
| 1545 | Resnick B, Galik E, Boltz M. Function focused care approaches: literature review of progress and future possibilities. Journal of the American Medical Directors Association. 2013 May 1;14(5):313-8. | Letters to the editor/ Review studies |
| 1546 | Resnick B, Galik E, Gruber‐Baldini A, Zimmerman S. Testing the effect of function‐focused care in assisted living. Journal of the American Geriatrics Society. 2011 Dec;59(12):2233-40. | Study not about effectiveness |
| 1547 | Resnick B, Galik E, Vigne E, Carew AP. Dissemination and implementation of function focused care for assisted living. Health Education & Behavior. 2016 Jun;43(3):296-304. | Study not about effectiveness |
| 1548 | Resnick B, Kolanowski A, Van Haitsma K, Boltz M, Galik E, Bonner A, Vigne E, Holtzman L, Mulhall PM. Pilot testing of the EIT-4-BPSD intervention. American Journal of Alzheimer's Disease & Other Dementias®. 2016 Nov;31(7):570-9. | Not a champion |
| 1549 | Reymond L, Israel FJ, Charles MA. A residential aged care end-of-life care pathway (RAC EoLCP) for Australian aged care facilities. Australian Health Review. 2011 Aug 25;35(3):350-6. | Study not about effectiveness |
| 1550 | Rhoton B, Biller JM, Prause P, Cochran JB, Ball N, Augustus R, Formby L. Preventing Central Line Associated Bloodstream Infections in the PICU: Changing What's Possible in Infection Prevention. American Journal of Infection Control. 2012 Jun 1;40(5):e60-1. | Conference abstract |
| 1551 | Riccioni N, Berlanga R, Hagan J, Schier R, Gordon M. Interrater reliability of the Braden and Braden Q by Skin Champion nurses. Journal of pediatric nursing. 2019 Jan 1;44:9-15. | Study not about effectiveness |
| 1552 | Richardson D, Fortin J, Avinoam G, Skrabka K, Willems J, Sharp S, Linkewich B. Evidence-Informed Knowledge to Practice: Implementation of Stroke Best Practices. InSTROKE 2013 Dec 1 (Vol. 44, No. 12, pp. E203-E203). | Conference abstract |
| 1553 | Richardson NC, Behringer B, Warren MD. Barriers to the integration of tobacco cessation programs in primary care practices in Tennessee. | Conference abstract |
| 1554 | Riedel A. Standardized skin rounds at small independent children's hospital to decrease skin integrity-related injuries. Journal of wound ostomy and continence nursing 2020 May 1 (Vol. 47, pp. S18-S19). | Conference abstract |
| 1555 | Rieger KL, Hack TF, Beaver K, Schofield P. Should consultation recording use be a practice standard? A systematic review of the effectiveness and implementation of consultation recordings. Psycho‐oncology. 2018 Apr;27(4):1121-8. | Letters to the editor/ Review studies |
| 1556 | Riley BL. Dissemination of heart health promotion in the Ontario Public Health System: 1989–1999. Health Education Research. 2003 Feb 1;18(1):15-31. | Study not about effectiveness |
| 1557 | Riley M, Patterson V, Lane JC, Won KM, Ranalli L. The adolescent champion model: primary care becomes Adolescent-Centered via targeted quality improvement. The Journal of pediatrics. 2018 Feb 1;193:229-36. | Study not about effectiveness |
| 1558 | Roberts JH, Perrow F. 226.‘GP Champions' in England; Family Physicians and Youth Workers Collaborate in a New Model of Youth-friendly Primary Care Practice to Address Unmet Need. Journal of Adolescent Health. 2015 Feb 1;56(2):S115-6. | Conference abstract |
| 1559 | Roberts, J., Ozer, E.; Haller, D.; Gray, N., Sanci, L."Spreading the word": Creating a Critical Mass of Youth-Friendly Practitioners in Primary Care and Developing Their Role in Preventive Interventions. Turkish Archives of Pediatrics / 2013;48(Suppl 2):25 | Conference abstract |
| 1560 | Robinson C, Hoze M, Hevener S, Nichols AA. Development of an RN champion model to improve the outcomes of ventilator-associated pneumonia patients in the intensive care unit. JONA: The Journal of Nursing Administration. 2018 Feb 1;48(2):79-84. | Study not about effectiveness |
| 1561 | Robinson E, Lagu T. TRANSFORM-ing Patient Safety Culture: A Universal Imperative. | Study not about effectiveness |
| 1562 | Robinson E. A Changing World Demands Care That Takes a Consumer-Centric Approach. Frontiers of health services management. 2019 Dec 1;36(2):3-14. | Not about knowledge translation/evidence-based practice |
| 1563 | Robinson JM, Renfro CP, Shockley SJ, Blalock SJ, Watkins AK, Ferreri SP. Training and toolkit resources to support implementation of a community pharmacy fall prevention service. Pharmacy. 2019 Sep;7(3):113. | Study not about effectiveness |
| 1564 | Robinson S, Brownett T. Educating public health champions. Health Education Journal. 2018 Dec;77(8):978-94. | Not within a health care setting |
[truncated: 125,198 more chars]
