# Supplementary material for: The effectiveness of champions in implementing innovations in health care: a systematic review
Source: Implement Sci Commun. 2022 Jul 22;3:80. doi: 10.1186/s43058-022-00315-0 (PMC9308185; doi:10.1186/s43058-022-00315-0)
Supplement: Supplementary file 5 — Additional file 5. Quality Appraisal Assessments. [file 43058_2022_315_MOESM5_ESM.docx]

**Additional File 5: Quality Appraisal Assessments**

**Supplemental Table 1**: Quality Appraisal of Included Cross-sectional Studies (*n* = 24)

| **Author, Year** | **Q1** | **Q2** | **Q3** | **Q4** | **Q5** | **Q6** | **Q7** | **Q8** | **Total Score** | **Quality Score** | **Quality** |
| --- | --- | --- | --- | --- | --- | --- | --- | --- | --- | --- | --- |
| Albert, 2012 | Yes | Yes | No | Yes | Yes | Yes | No | Yes | 12 | .75 | Strong |
| Alidina, 2018 | Yes | Yes | No | Yes | Yes | Yes | No | Yes | 12 | .75 | Strong |
| Ash, 1997 | Yes | No | No | Yes | Yes | Yes | No | Yes | 10 | .63 | Moderate |
| Ben-David, 2019 | Yes | No | No | Yes | No | No | Yes | No | 6 | .38 | Weak |
| Bradley, 2012 | Yes | Yes | No | Yes | Yes | Yes | Yes | Yes | 7 | .44 | Weak |
| Chang, 2012 | Yes | No | No | Yes | Yes | Yes | No | Yes | 10 | .63 | Moderate |
| Ellerbeck, 2006 | No | No | No | Yes | Yes | No | No | No | 4 | .25 | Weak |
| Goff, 2019 | Yes | Yes | No | Yes | Yes | No | No | No | 8 | .5 | Moderate |
| Granade, 2020 | Yes | Yes | No | Yes | Yes | Yes | No | Yes | 12 | .75 | Strong |
| Hsia, 2019 | No | Yes | Yes | Yes | Yes | Yes | Yes | Yes | 14 | .88 | Strong |
| Hung, 2008 | No | Yes | No | Yes | Yes | Yes | No | Yes | 10 | .63 | Strong |
| Kabukye, 2020 | Yes | Yes | Yes | Yes | Yes | Yes | Yes | Yes | 16 | 1 | Strong |
| Kenny,2005 | Yes | Yes | No | Yes | Yes | Yes | Yes | Yes | 14 | .88 | Strong |
| Khera, 2018 | Yes | No | No | Yes | Yes | Yes | No | Yes | 10 | .63 | Strong |
| Korall, 2017, 2018^1^ | Yes | Yes | No | Yes | Yes | Yes | Yes | Yes | 14 | .88 | Strong |
| Lago, 2013 | Yes | Yes | No | Yes | No | No | No | No | 6 | .38 | Weak |
| Papadakis, 2014 | Yes | Yes | No | Yes | Yes | Yes | No | Yes | 12 | .75 | Strong |
| Pare, 2011 | No | Yes | Yes | Yes | Yes | Yes | Yes | Yes | 14 | .88 | Strong |
| Patton, 2013 | Yes | No | No | Yes | No | No | No | Yes | 6 | .38 | Weak |
| Shea, 2016 | No | No | No | Yes | Yes | Yes | Yes | Yes | 10 | .63 | Moderate |
| Strasser, 2003 | Yes | Yes | No | Yes | Yes | Yes | No | Yes | 12 | .75 | Strong |
| Tierney, 2003 | Yes | Yes | No | Yes | Yes | Yes | No | Yes | 12 | .75 | Strong |
| Ward, 2004 | Yes | Yes | No | Yes | Yes | Yes | No | Yes | 12 | .75 | Strong |
| Westrick, 2009 | Yes | Yes | Yes | Yes | Yes | Yes | Yes | Yes | 16 | 1 | Strong |
| % of Yes | 80 | 68 | 16 | 100 | 88 | 80 | 36 | 88 |  |  |  |

1 - Korall, 2017 and Korall, 2018 are from one study but had two reports.

**Note:** Scoring system: Yes = 2 points, Unclear = 1 point, and No = 0 point

Q1- Were the criteria for inclusion in the sample clearly defined?

Q2- Were the study subjects and the setting described in detail?

Q3- Was the exposure measured in a valid and reliable way?

Q4 - Were objective, standard criteria used for measurement of the condition?

Q5- Were confounding factors identified?

Q6 - Were strategies to deal with confounding factors stated?

Q7 - Were the outcomes measured in a valid and reliable way?

Q8- Was appropriate statistical analysis used?;

**Supplemental Table 2**: Quality Appraisal of Included Cross-sectional Studies without Weak Studies (*n* = 20)

| **Author, Year** | **Q1** | **Q2** | **Q3** | **Q4** | **Q5** | **Q6** | **Q7** | **Q8** | **Total Score** | **Quality Score** | **Quality** |
| --- | --- | --- | --- | --- | --- | --- | --- | --- | --- | --- | --- |
| Albert, 2012 | Yes | Yes | No | Yes | Yes | Yes | No | Yes | 12 | .75 | Strong |
| Alidina, 2018 | Yes | Yes | No | Yes | Yes | Yes | No | Yes | 12 | .75 | Strong |
| Ash, 1997 | Yes | No | No | Yes | Yes | Yes | No | Yes | 10 | .63 | Moderate |
| Chang, 2012 | Yes | No | No | Yes | Yes | Yes | No | Yes | 10 | .63 | Moderate |
| Goff, 2019 | Yes | Yes | No | Yes | Yes | No | No | No | 8 | .5 | Moderate |
| Granade, 2020 | Yes | Yes | No | Yes | Yes | Yes | No | Yes | 12 | .75 | Strong |
| Hsia, 2019 | No | Yes | Yes | Yes | Yes | Yes | Yes | Yes | 14 | .88 | Strong |
| Hung, 2008 | No | Yes | No | Yes | Yes | Yes | No | Yes | 10 | .63 | Strong |
| Kabukye, 2020 | Yes | Yes | Yes | Yes | Yes | Yes | Yes | Yes | 16 | 1 | Strong |
| Kenny,2005 | Yes | Yes | No | Yes | Yes | Yes | Yes | Yes | 14 | .88 | Strong |
| Khera, 2018 | Yes | No | No | Yes | Yes | Yes | No | Yes | 10 | .63 | Strong |
| Korall, 2017, 2018^1^ | Yes | Yes | No | Yes | Yes | Yes | Yes | Yes | 14 | .88 | Strong |
| Papadakis, 2014 | Yes | Yes | No | Yes | Yes | Yes | No | Yes | 12 | .75 | Strong |
| Pare, 2011 | No | Yes | Yes | Yes | Yes | Yes | Yes | Yes | 14 | .88 | Strong |
| Shea, 2016 | No | No | No | Yes | Yes | Yes | Yes | Yes | 10 | .63 | Moderate |
| Strasser, 2003 | Yes | Yes | No | Yes | Yes | Yes | No | Yes | 12 | .75 | Strong |
| Tierney, 2003 | Yes | Yes | No | Yes | Yes | Yes | No | Yes | 12 | .75 | Strong |
| Ward, 2004 | Yes | Yes | No | Yes | Yes | Yes | No | Yes | 12 | .75 | Strong |
| Westrick, 2009 | Yes | Yes | Yes | Yes | Yes | Yes | Yes | Yes | 16 | 1 | Strong |
| % of Yes | 80 | 75 | 20 | 100 | 100 | 95 | 35 | 95 |  |  |  |

1 - Korall, 2017 and Korall, 2018 are from one study but had two reports.

**Note:** Scoring system: Yes = 2 points, Unclear = 1 point, and No = 0 point

Q1- Were the criteria for inclusion in the sample clearly defined?

Q2- Were the study subjects and the setting described in detail?

Q3- Was the exposure measured in a valid and reliable way?

Q4 - Were objective, standard criteria used for measurement of the condition?

Q5- Were confounding factors identified?

Q6 - Were strategies to deal with confounding factors stated?

Q7 - Were the outcomes measured in a valid and reliable way?

Q8- Was appropriate statistical analysis used?

**Supplemental Table 3**: Quality Appraisal of Included Non-controlled Before and After and Interrupted Time Series Studies (*n* = 6)

| **Author, Year** | **Q1** | **Q2** | **Q3** | **Q4** | **Q5** | **Q6** | **Q7** | **Q8** | **Q9** | **Total Score** | **Quality Score** | **Quality** |
| --- | --- | --- | --- | --- | --- | --- | --- | --- | --- | --- | --- | --- |
| Campbell, 2008 | Yes | Yes | Yes | No | No | Yes | Yes | No | Yes | 12 | .67 | Moderate |
| Foster, 2017 | Yes | Yes | Yes | No | No | Yes | Yes | No | Yes | 12 | .67 | Moderate |
| Sharkey, 2013 | Yes | Yes | Yes | No | No | Yes | Yes | No | Yes | 12 | .67 | Moderate |
| Soni, 2016 | Yes | Yes | Yes | No | Yes | Yes | Yes | No | Yes | 14 | .78 | Strong |
| Weiler, 2012, 2013 ^1^ | Yes | Yes | Yes | No | Yes | Yes | Yes | No | Yes | 14 | .78 | Strong |
| Zavalkoff, 2015 | Yes | Yes | Yes | No | Yes | Yes | Yes | No | Yes | 14 | .78 | Strong |
| % of Yes | 100 | 100 | 100 | 0 | 50 | 100 | 100 | 0 | 100 |  |  |  |

1 - Weiler, 2012 and Weiler, 2013 are one study with two reports.

**Note:** Scoring system: Yes = 2 points, Unclear = 1 point, and No = 0 point

Q1: Is it clear in the study what is the ‘cause’ and what is the ‘effect’ (i.e., there is no confusion about which variable comes first)?

Q2: Were the participants included in any comparisons similar?

Q3: Were the participants included in any comparisons receiving similar treatment/care, other than the exposure or intervention of interest?

Q4: Was there a control group?

Q5: Were there multiple measurements of the outcome both pre and post the intervention/exposure?

Q6: Was follow up complete and if not, were differences between groups in terms of their follow up adequately described and analyzed?

Q7: Were the outcomes of participants included in any comparisons measured in the same way?

Q8: Were outcomes measured in a reliable way?

Q9: Was appropriate statistical analysis used?

**Supplemental Table 4**: Quality Appraisal of Included Cohort Studies (*n* =3)

| **Author, Year** | **Q1** | **Q2** | **Q3** | **Q4** | **Q5** | **Q6** | **Q7** | **Q8** | **Q9** | **Q10** | **Q11** | **Total Score** | **Quality Score** | **Quality** |
| --- | --- | --- | --- | --- | --- | --- | --- | --- | --- | --- | --- | --- | --- | --- |
| Anand, 2017 | Yes | Yes | No | Yes | Yes | Unclear | Yes | Yes | Yes | N/A | Yes | 17 | .77 | Moderate |
| Sisodia, 2020 | Yes | Yes | No | Yes | Yes | Yes | No | Yes | Yes | N/A | Yes | 16 | .72 | Moderate |
| Whitebird, 2014 | Unclear | Yes | No | Yes | No | Yes | No | Yes | Yes | N/A | No | 11 | .5 | Moderate |
| % of Yes | 66.7 | 100 | 0 | 100 | 66.7 | 66.7 | 33.3 | 100 | 100 | N/A | 66.7 |  |  |  |

**Note:** Scoring system: Yes = 2 points, Unclear = 1 point, and No = 0 point

Q1: Were the two groups similar and recruited from the same population?

Q2: Were the exposures measured similarly to assign people to both exposed and unexposed groups?

Q3: Was the exposure measured in a valid and reliable way?

Q4: Were confounding factors identified?

Q5: Were strategies to deal with confounding factors stated?

Q6: Were the groups/participants free of the outcome at the start of the study (or at the moment of exposure)?

Q7: Were the outcomes measured in a valid and reliable way?

Q8: Was the follow up time reported and sufficient to be long enough for outcomes to occur?

Q9: Was follow up complete, and if not, were the reasons to loss to follow up described and explored?

Q10: Were strategies to address incomplete follow up utilized?

Q11: Was appropriate statistical analysis used?

**Supplemental Table 5**: Quality Appraisal of Included Case Control Studies (*n* =1)

| **Author, Year** | **Q1** | **Q2** | **Q3** | **Q4** | **Q5** | **Q6** | **Q7** | **Q8** | **Q9** | **Q10** | **Total Score** | **Quality Score** | **Quality** |
| --- | --- | --- | --- | --- | --- | --- | --- | --- | --- | --- | --- | --- | --- |
| Slaunwhite, 2009 | Yes | No | No | No | Yes | No | Yes | No | Yes | Yes | 10 | .5 | Moderate |
| % of Yes | 100 | 0 | 0 | 0 | 100 | 0 | 100 | 0 | 100 | 100 |  |  |  |

**Note:** Scoring system: Yes = 2 points, Unclear = 1 point, and No = 0 point

Q1: Were the groups comparable other than presence of the exposure of interest in cases or absence of disease in controls?

Q2: Were the study subjects and the setting described in detail?

Q3: Was the exposure measured in a valid and reliable way?

Q4: Was exposure measured in a standard, valid and reliable way?

Q5: Was exposure measured in the same way for cases and controls?

Q6: Were confounding factors identified?

Q7: Were strategies to deal with confounding factors stated?

Q8: Were outcomes assessed in a standard, valid and reliable way for cases and controls?

Q9: Was the exposure period of interest long enough to be meaningful?

Q10: Was the exposure period of interest long enough to be meaningful?

**Supplemental Table 6**: Quality Appraisal of Included Randomized Controlled Trials (*n* =1)

| **Author, Year** | **Q1** | **Q2** | **Q3** | **Q4** | **Q5** | **Q6** | **Q7** | **Q8** | **Q9** | **Q10** | **Q11** | **Q12** | **Q13** | **Total Score*** | **Quality Score** | **Quality** |
| --- | --- | --- | --- | --- | --- | --- | --- | --- | --- | --- | --- | --- | --- | --- | --- | --- |
| Bentz, 2007 | Unclear | Unclear | Yes | Unclear | Yes | Unclear | Yes | Yes | Yes | Yes | No | Yes | Yes | 20 | .87 | Strong |
| % of Yes | 0 | 0 | 100 | 0 | 100 | 0 | 100 | 100 | 100 | 100 | 0 | 100 | 100 |  |  |  |

**Note**: Scoring system: Yes = 2 points, Unclear = 1 point, and No = 0 point

Q1: Was true randomization used for assignment of participants to treatment groups?

Q2: Was allocation to groups concealed?

Q3: Were treatment groups similar at the baseline?

Q4: Were participants blind to treatment assignment?

Q5: Were those delivering treatment blind to treatment assignment?

Q6: Were outcomes assessors blind to treatment assignment?

Q7: Were treatment groups treated identically other than the intervention of interest?

Q8: Was follow up complete and if not, were differences between groups in terms of their follow up adequately described and analyzed?

Q9: Were participants analyzed in the groups to which they were randomized?

Q10: Were outcomes measured in the same way for treatment groups?

Q11: Were outcomes measured in a reliable way?

Q12: Was appropriate statistical analysis used?

Q13: Was the trial design appropriate for the topic, and any deviations from the standard RCT design accounted for in the conduct and analysis?
