# Supplementary material for: The effectiveness of champions in implementing innovations in health care: a systematic review
Source: Implement Sci Commun. 2022 Jul 22;3:80. doi: 10.1186/s43058-022-00315-0 (PMC9308185; doi:10.1186/s43058-022-00315-0)
Supplement: Supplementary file 2 — Additional file 2. Synthesis Without Meta-analysis (SWIM) in Systematic Reviews Reporting Guideline Checklist. [file 43058_2022_315_MOESM2_ESM.docx]

**Additional File 2: Synthesis Without Meta-analysis (SWIM) in Systematic Reviews Reporting Guideline Checklist**

Table 1. Synthesis Without Meta-analysis (SWIM) items

| **SWiM reporting item** | **Item description** | **Page in manuscript where item is reported** | **Other*** |
| --- | --- | --- | --- |
| **Methods** | | | |
| 1. Grouping studies  for synthesis | 1a) Provide a description of, and rationale for, the groups used in the synthesis (eg, groupings of populations, interventions, outcomes, study design) | p.4 |  |
|  | 1b) Detail and provide rationale for any changes made subsequent to the protocol in the groups used in the synthesis | Not applicable |  |
| 2. Describe the standardised metric and  transformation methods used | Describe the standardised metric for each outcome. Explain why the metric(s) was chosen and describe any methods used to transform the intervention effects, as reported in the study, to the standardised metric, citing any methodological guidance consulted | p.4-5 |  |
| 3. Describe the synthesis methods | Describe and justify the methods used to synthesise the effects for each outcome when it was not possible to undertake a meta-analysis of effect estimates | Table 1 on p.5 |  |
| 4. Criteria used to prioritise results for  summary and synthesis | Where applicable, provide the criteria used, with supporting justification, to select the particular studies, or a particular study, for the main synthesis or to draw conclusions from the synthesis (e.g., based on study design, risk of bias assessments, directness in relation to the review question) | p.4 explains categorization of studies.  Table 1, point 4 on p.5 details prioritization of multivariate statistics. |  |
| 5. Investigation of heterogeneity  in reported effects | State the method(s) used to examine heterogeneity in reported effects when it was not possible to undertake a meta-analysis of effect estimates and its extensions to investigate heterogeneity | In p.4 states that the heterogeneity is apparent from the varying outcomes. Table 5 and 6 demonstrates the variability of innovation types across the studies, and categories of innovation use and outcomes. |  |
| 6. Certainty of evidence | Describe the methods used to assess the certainty of the synthesis findings | p.4-5 |  |
| 7. Data presentation methods | Describe the graphical and tabular methods used to present the effects (e.g., tables, forest plots, harvest plots)  Specify key study characteristics (e.g., study design, risk of bias) used to order the studies, in the text and any tables or graphs, clearly referencing the studies included | page 4.  Also see Table 3 for detailed descriptions of each study, and tables 5 and 6 where we reported key study characteristics.  For methodological quality, please see p.5. |  |
| **Results** | | | |
| 8. Reporting results | For each comparison and outcome, provide a description of the synthesised findings, and the certainty of the findings. Describe the result in language that is consistent with the question the synthesis addresses, and indicate which studies contribute to the synthesis | For methodological quality, please see p.5 and additional file 5.  See p.8-41 for the text relating to the results. Also please see table 5 and 6 - for each of the main category, we summarized the conclusion/trends we made using the applied vote counting rules. |  |
| **Discussion** | | | |
| 9. Limitations of the synthesis | Report the limitations of the synthesis methods used and/or the groupings used in the synthesis, and how these affect the conclusions that can be drawn in relation to the original review question | p.46 |  |

Note: *If the information is not provided in the systematic review, give details of where this information is available (eg, protocol, other published papers (provide citation details), or website (provide the URL)). This table is from Campbell M, McKenzie JE, Sowden A, Katikireddi SV, Brennan SE, Ellis S, Hartmann-Boyce J, Ryan R, Shepperd S, Thomas J, Welch V. Synthesis without meta-analysis (SWiM) in systematic reviews: reporting guideline. bmj. 2020 Jan 16;368.
