# Supplementary material for: The effectiveness of champions in implementing innovations in health care: a systematic review
Source: Implement Sci Commun. 2022 Jul 22;3:80. doi: 10.1186/s43058-022-00315-0 (PMC9308185; doi:10.1186/s43058-022-00315-0)
Supplement: Supplementary file 3 — Additional file 3. All Accessed Databases and Peer Review Assessment. [file 43058_2022_315_MOESM3_ESM.docx]

**Additional File 3: All Accessed Databases and Peer Review Assessment**

**1. Database:** Medline

**Platform:** OVID

**Date of Search:** October 26, 2020

**Limits: None; Filters:** None

| **PCC (Population, Concept, Context)** | **Search terms** | **Results** |
| --- | --- | --- |
| Population: Champions | 1. (champion*).mp. 2. Peer Influence/ 3. Leadership/ 4. peer group/ 5. 3 and 4 6. 1 OR 2 or 5 | 7192  612  41564  20832  341  8141 |
| Concept: Knowledge translation/evidence base practice | 7. (ebp or ebm or ebn or cpg* or best practice* or (evidence adj2 practice*)).mp.  8. (guideline* adj2 (implement* or adher*)).mp.  9. (quality adj1 (improv* or manag*)).mp.  10. (research adj2 (‘use’ or utili* or adopt* or implement* or disseminat* or uptake or transfer* or translat* or support)).mp.  11. (knowledge adj2 (‘use’ or utili* or adopt* or implement* or disseminat* or uptake or transfer* or translat* or support)).mp.  12. (evidence adj2 (‘use’ or utili* or adopt* or implement* or disseminat* or uptake or transfer* or translat* or support)).mp.  13. ((innovation adj2 (adopt* or diffus*)) or (implementation adj2 (science* or research)) or (innovation or diffusion)).mp  14. exp Evidence-Based Practice/ or Information Dissemination/  15. guideline adherence/ or quality assurance, health care/ or quality improvement/ or Outcome Assessment, Health Care/ or Process Assessment, Health Care/ or "Outcome and Process Assessment, Health Care"/ or Safety Management/  16. "diffusion of innovation"/ or implementation science/ or technology transfer/ or Organizational Innovation/  17. OR/7-16 | 97979  38927  98951  9249429  17452  50289  298347  104811  225565  43244  9781948 |
| Context: Healthcare | 18. (health adj2 care or healthcare).mp.  19. ((health care adj2 (professional* or personnel* or provider* or occupation*)) or (healthcare adj2 (professional* or personnel* or provider* or occupation*)) or nurse* or physician* or dentist* or (allied adj2 profession*)).mp.  20. Patient care/ or "Delivery of Health Care"/ or Health Services Administration/ or Quality of Health Care/ or health resources/ or "health services needs and demand"/ or Health Services/ or Health Services Research/ or Hospitals/ or Ambulatory Care Facilities/  21. Health Personnel/ or Health Occupations/ or practice patterns, dentists'/ or practice patterns, nurses'/ or practice patterns, physicians'/ or professional practice gaps/ or "attitude of health personnel"/ or Interprofessional Relations/ or Health Knowledge, Attitudes, Practice/  22. OR/18-21 | 990507  1104944  365331  431637  2099889 |
| Combinations | 25. 6 AND 17 AND 22 | 1498 |

**2. Database:** Psych Info

**Platform:** OVID

**Date of Search:** October 26, 2020

**Limits: None; Filters:** None

1	champion*.mp. (3795)

2	interpersonal influences/ (6032)

3	Leadership/ (34935)

4	exp peers/ (13051)

5	3 and 4	(171)

6	1 or 2 or 5 (9983)

7	(ebp or ebm or ebn or cpg* or best practice* or (evidence adj2 practice*)).mp. (42383)

8	(guideline* adj2 (implement* or adher*)).mp. (1681)

9	(quality adj1 (improv* or manag*)).mp. (12329)

10	(research adj2 ('use' or utili* or adopt* or implement* or disseminat* or uptake or transfer* or translat* or support)).mp. (24271)

11	(knowledge adj2 ('use' or utili* or adopt* or implement* or disseminat* or uptake or transfer* or translat* or support)).mp. (13587)

12	(evidence adj2 ('use' or utili* or adopt* or implement* or disseminat* or uptake or transfer* or translat* or support)).mp. (18320)

13	((innovation adj2 (adopt* or diffus*)) or (implementation adj2 (science* or research)) or (innovation or diffusion)).mp.	(47221)

14	Evidence Based Practice/ or Information Dissemination/ (19788)

15	treatment guidelines/ or quality control/ or "treatment process and outcome measures"/ or patient safety/ or Program Evaluation/ (23436)

16	innovation/ (14003)

17	or/7-16	(166248)

18	((health adj2 care) or healthcare).mp. (212797)

19	((health care adj2 (professional* or personnel* or provider* or occupation*)) or (healthcare adj2 (professional* or personnel* or provider* or occupation*)) or nurse* or physician* or dentist* or (allied adj2 profession*)).mp. (175468)

20	Health Care Delivery/ or Health Care Services/ or Health Care Administration/ or Health Care Administration/ or Hospitals/ or Outpatient Treatment/ (82534)

21	Health Personnel Attitudes/ or Health Personnel/ (35993)

22	or/18-21 (344095)

23	6 and 17 and 22 (199)

**3. Database:** Embase

**Platform:** OVID

**Date of Search:** October 26, 2020

**Limits: None; Filters:** None

1	champion*.mp. (9734)

2	peer pressure/	(2584)

3	leadership/ (69681)

4	peer group/ (24180)

5	3 and 4	(553)

6	1 or 2 or 5 (12849)

7	(ebp or ebm or ebn or cpg* or best practice* or (evidence adj2 practice*)).mp. (191909)

8	(guideline* adj2 (implement* or adher*)).mp. (15599)

9	(quality adj1 (improv* or manag*)).mp. (144271)

10	(research adj2 ('use' or utili* or adopt* or implement* or disseminat* or uptake or transfer* or translat* or support)).mp. (63323)

11	(knowledge adj2 ('use' or utili* or adopt* or implement* or disseminat* or uptake or transfer* or translat* or support)).mp. (23055)

12	(evidence adj2 ('use' or utili* or adopt* or implement* or disseminat* or uptake or transfer* or translat* or support)).mp. (63915)

13	((innovation adj2 (adopt* or diffus*)) or (implementation adj2 (science* or research)) or (innovation or diffusion)).mp.	(353964)

14	evidence based practice/ or information dissemination/ (87660)

15	protocol compliance/ or quality control/ or total quality management/ or outcome assessment/ or safety/	(1054843)

16	implementation science/ (1418)

17	or/7-16	(1767408)

18	((health adj2 care) or healthcare).mp.	(1840718)

19	((health care adj2 (professional* or personnel* or provider* or occupation*)) or (healthcare adj2 (professional* or personnel* or provider* or occupation*)) or nurse* or physician* or dentist* or (allied adj2 profession*)).mp. (1385992)

20	patient care/ or health care/ or health service/ or health care quality/ or health care planning/ or hospital/ or ambulatory care/ or health services research/ (1209612)

21	health care personnel/ or medical profession/ or practice gap/ or health personnel attitude/ or clinical practice/ or public relations/ or attitude to health/	(692041)

22	or/18-21 (3501254)

23	6 and 17 and 22 (1946)

**4. Database:** CINAHL

**Platform:** EBSCO

**Date of Search:** October 26, 2020

**Limits: None; Filters:** None

| **#** | **Query** | **Results** |
| --- | --- | --- |
| S23 | S6 AND S17 AND S22 | 851 |
| S22 | S18 OR S19 OR S20 OR S21 | 1,404,487 |
| S21 | (MH "Health Personnel") OR (MH "Health Occupations") OR (MH "Attitude of Health Personnel") OR (MH "Practice Patterns") OR (MH "Interprofessional Relations") | 118,133 |
| S20 | (MH "Patient Care") OR (MH "Health Care Delivery") OR (MH "Health Care Delivery, Integrated") OR (MH "Health Services Administration") OR (MH "Health Care Supervision (Omaha)") OR (MH "Health Services Needs and Demand") OR (MH "Health Services") OR (MH "Quality of Health Care") OR (MH "Health Services Research") OR (MH "Hospitals") OR (MH "Ambulatory Care") | 275,787 |
| S19 | (((health care n2 (professional* or personnel* or provider* or occupation*)) OR ((healthcare n2 (professional* or personnel* or provider* or occupation*)) OR ( nurse* or physician* or dentist* or (allied n2 profession*)) | 813,843 |
| S18 | (healthcare OR health n2 care) | 666,707 |
| S17 | S7 OR S8 OR S9 OR S10 OR S11 OR S12 OR S13 OR S14 OR S15 OR S16 | 410,447 |
| S16 | (MH "Diffusion of Innovation") OR (MH "Implementation Science") | 15,458 |
| S15 | ( (MH "Quality Assurance") OR (MH "Quality Assessment") OR (MH "Quality Improvement") ) OR ( (MH "Guideline Adherence") OR (MH "Process Assessment (Health Care)") OR (MH "Outcome Assessment") ) | 140,803 |
| S14 | (MH "Evidence-Based Dental Practice") OR (MH "Medical Practice, Evidence-Based") OR (MH "Nursing Practice, Evidence-Based") OR (MH "Professional Practice, Evidence-Based") OR (MH "Occupational Therapy Practice, Evidence-Based") OR (MH "Physical Therapy Practice, Evidence-Based") OR (MH "Professional Practice, Evidence-Based") OR (MH "Professional Practice, Research-Based") OR (MH "Information Management") | 84,351 |
| S13 | ((innovation n2 (adopt* or diffus*)) or (implementation n2 (science* or research)) or (innovation or diffusion) | 59,670 |
| S12 | (evidence n2 (use or utili* or adopt* or implement* or disseminat* or uptake or transfer* or translat* or support)) | 36,420 |
| S11 | (knowledge n2 (use or utili* or adopt* or implement* or disseminat* or uptake or transfer* or translat* or support)) | 12,831 |
| S10 | (research n2 (use or utili* or adopt* or implement* or disseminat* or uptake or transfer* or translat* or support)) | 41,622 |
| S9 | (quality n1 (improv* or manag*)) | 102,809 |
| S8 | (guideline* n2 (implement* or adher*)) | 21,077 |
| S7 | (ebp or ebm or ebn or cpg* or best practice* or (evidence n2 practice*)) | 109,076 |
| S6 | S1 OR S2 OR S5 | 6,662 |
| S5 | S3 AND S4 | 239 |
| S4 | (MH "Peer Group") | 13,419 |
| S3 | (MH "Leadership") | 43,397 |
| S2 | (MH "Peer Pressure") | 946 |
| S1 | (champion*) | 5,483 |

**5. Database:** Nursing and Allied Health

**Platform:** ProQuest

**Date of Search:** October 26, 2020

**Limits: None; Filters:** None

S1: noft((champion*))- Results: 9538

S2: mainsubject.Exact("peer influence") - Results: 38

S3: mainsubject.Exact("leadership")- Results: 23456

S4: mainsubject.Exact("peer group") - Results: 1711

S5: S3 AND S4 - Results: 33

S6: S1 OR S2 OR S5 - Results: 9609

S7: noft((ebp or ebm or ebn or cpg* or best practice* or (evidence Near/2 practice*))) –

Results: 89797

S8: noft((guideline* Near/2 (implement* or adher*))) - Results: 7254

S9: noft((quality Near/1 (improv* or manag*))) - Results: 47049

S10: noft((research Near/2 (use or utili* or adopt* or implement* or disseminat* or uptake or transfer* or translat* or support))) - Results: 50631

S11: noft((knowledge Near/2 (use or utili* or adopt* or implement* or disseminat* or uptake or transfer* or translat* or support))) - Results: 7913

S12: noft((evidence Near/2 (use or utili* or adopt* or implement* or disseminat* or uptake or transfer* or translat* or support))) - Results: 22716

S13: noft(((innovation Near/2 (adopt* or diffus*)) or (implementation Near/2 (science* or research)) or (innovation or diffusion))) - Results: 76766

S14: mainsubject.Exact("evidence-based practice" OR "evidence-based nursing" OR "information dissemination" OR "evidence based practice" OR "evidence based" OR "evidence-based medicine")- Results: 29036

S15: mainsubject.Exact("process assessment (health care)" OR "safety management" OR "outcome & process assessment (health care)" OR "guideline adherence" OR "quality improvement" OR "outcome assessment (health care)" OR "quality assurance, health care") -Results: 21658

S16: mainsubject.Exact("organizational innovation" OR "implementation" OR "diffusion of innovation") - Results: 6045

S17: S7 OR S8 OR S9 OR S10 OR S11 OR S12 OR S13 OR S14 OR S15 OR S16 - Results: 310192

S18: noft((healthcare OR health Near/2 care)) - Results: 668296

S19: noft(((health care Near/2 (professional* or personnel* or provider* or occupation*)) ) OR ( (healthcare Near/2 (professional* or personnel* or provider* or occupation*)) ) OR ( nurse* or physician* or dentist* or (allied Near/2 profession*))) - Results: 625394

S20: mainsubject.Exact("health services research" OR "hospitals" OR "quality of health care" OR "ambulatory care facilities" OR "health resources" OR "health services needs & demand" OR "health services administration" OR "health services" OR "patient care" OR "delivery of health care") - Results: 230358

S21: mainsubject.Exact("health knowledge, attitudes, practice" OR "health occupations" OR "attitude of health personnel" OR "interprofessional relations" OR "health personnel") - Results: 25378

S22: S18 OR S19 OR S20 OR S21 - Results: 1215547

S23: S6 AND S17 AND S22 - Results: 635

**6. Database:** Dissertations and Theses Global

**Platform:** ProQuest

**Date of Search:** October 26, 2020

**Limits: None; Filters:** None

S1: noft(champion*) - Results: 4975

S2: noft((ebp or ebm or ebn or cpg* or best practice* or (evidence Near/2 practice*))) - Results: 89725

S3: noft((guideline* Near/2 (implement* OR adher*))) - Results: 1607

S4: noft((quality Near/1 (improv* OR manag*))) - Results: 34540

S5: noft((research Near/2 (use OR utili* OR adopt* OR implement* OR disseminat* OR uptake OR transfer* OR translat* OR support))) - Results: 59277

S6: noft((knowledge Near/2 (use OR utili* OR adopt* OR implement* OR disseminat* OR uptake OR transfer* OR translat* OR support))) - Results: 16978

S7: noft((evidence Near/2 (use OR utili* OR adopt* OR implement* OR disseminat* OR uptake OR transfer* OR translat* OR support))) - Results: 19347

S8: noft(((innovation Near/2 (adopt* OR diffus*)) OR (implementation Near/2 (science* OR research)) OR (innovation OR diffusion))) - Results: 133870

S9: S2 OR S3 OR S4 OR S5 OR S6 OR S7 OR S8 - Results: 323847

S10: noft((healthcare OR health Near/2 care)) - Results: 72243

S11: noft(((health care Near/2 (professional* or personnel* or provider* or occupation*)) ) OR ( (healthcare Near/2 (professional* or personnel* or provider* or occupation*)) ) OR ( nurse* or physician* or dentist* or (allied Near/2 profession*))) - Results: 74797

S12: S10 OR S11 - Results: 118765

S13: S1 AND S9 AND S12 - Results: 104

**7. Database:** Scopus

**Platform:** Scopus

**Date of Search:** October 26, 2020

**Limits: None; Filters:** None

1. TITLE-ABS-KEY ((champion*)) - 24,276 document results

2. TITLE-ABS-KEY (((ebp OR ebm OR ebn OR cpg* OR “best practice*” OR (evidence W/2 practice* )))) - 239,243 document results

3. TITLE-ABS-KEY (((guideline* W/2 (implement* OR adher*)))) - 47,176 document results

4. TITLE-ABS-KEY (((quality W/1 (improv* OR manag*)))) - 390,089 document results

5. TITLE-ABS-KEY (((research W/2 (use OR utili* OR adopt* OR implement* OR disseminat* OR uptake OR transfer* OR translat* OR support)))) - 160,148 document results

6. TITLE-ABS-KEY (((knowledge W/2 (use OR utili* OR adopt* OR implement* OR disseminat* OR uptake OR transfer* OR translat* OR support)))) -71,312 document results

7. TITLE-ABS-KEY (((evidence W/2 (use OR utili* OR adopt* OR implement* OR disseminat* OR uptake OR transfer* OR translat* OR support )))) - 117,191 document results

8. TITLE-ABS-KEY ((((innovation W/2 (adopt* OR diffus*)) OR (implementation W/2 (science* OR research )) OR ( innovation OR diffusion )))) - 1,415,950 document results

9. #2 OR # 3 OR #4 OR #5 OR #6 OR #7 OR #8 - 2,337,377 document results

10. TITLE-ABS-KEY (((healthcare OR health W/2 care))) - 1,742,498 document results

11. TITLE-ABS-KEY ( ( ( health AND care W/2 ( professional* OR personnel* OR provider* OR occupation* ) ) OR ( healthcare W/2 ( professional* OR personnel* OR provider* OR occupation* ) ) OR nurse* OR physician* OR dentist* OR ( allied W/2 profession* ) ) ) - 1,537,780 document results

12. #10 OR #11- 2,781,641 document results

13. #1 AND #9 AND #12 - 1,104 document results

**8. Database:** Business Source Complete

**Platform:** EBSCO

**Date of Search:** October 26, 2020

**Limits: None; Filters:** None

| **#** | **Query** | **Results** |
| --- | --- | --- |
| S22 | S5 AND S16 AND S21 | 93 |
| S21 | S17 OR S18 OR S19 OR S20 | 607,907 |
| S20 | (DE "MEDICAL personnel") OR (DE "HOSPITAL personnel") | 14,962 |
| S19 | (((DE "MEDICAL care") OR (DE "HEALTH facilities")) OR (DE "HEALTH services administrators")) OR (DE "HOSPITALS") OR (DE "MEDICAL offices") | 136,941 |
| S18 | ( (health care n2 (professional* or personnel* or provider* or occupation*)) ) OR ( (healthcare n2 (professional* or personnel* or provider* or occupation*)) ) OR ( nurse* or physician* or dentist* or allied profession* ) | 198,239 |
| S17 | (healthcare OR health n2 care) | 486,635 |
| S16 | S6 OR S7 OR S8 OR S9 OR S10 OR S11 OR S12 OR S13 OR S14 OR S15 | 596,150 |
| S15 | DE "DIFFUSION of innovations" OR DE "INNOVATION adoption" | 9,911 |
| S14 | DE "QUALITY assurance" | 8,021 |
| S13 | DE "INFORMATION resources management" OR DE "EVIDENCE-based management" | 29,407 |
| S12 | ((innovation n2 (adopt* or diffus*)) or (implementation n2 (science* or research)) or (innovation or diffusion) | 417,653 |
| S11 | (evidence n2 (use or utili* or adopt* or implement* or disseminat* or uptake or transfer* or translat* or support)) | 11,527 |
| S10 | (knowledge n2 (use or utili* or adopt* or implement* or disseminat* or uptake or transfer* or translat* or support)) | 16,109 |
| S9 | (research n2 (use or utili* or adopt* or implement* or disseminat* or uptake or transfer* or translat* or support)) | 27,572 |
| S8 | (quality n1 (improv* or manag*)) | 59,356 |
| S7 | (guideline* n2 (implement* or adher*)) | 1,857 |
| S6 | (ebp or ebm or ebn or cpg* or best practice* or (evidence n2 practice*)) | 52,381 |
| S5 | S1 OR S4 | 44,348 |
| S4 | S2 AND S3 | 219 |
| S3 | DE "LEADERSHIP" | 71,145 |
| S2 | DE "PROFESSIONAL relationships" OR DE "COWORKER relationships" | 3,369 |
| S1 | (champion*) | 44,129 |

**PEER REVIEW ASSESSMENT: THIS SECTION TO BE FILLED IN BY THE REVIEWER (Tamara R)**

**Champions Scoping Review Search Strategy Draft**

**Scoping review objective**: To examine published and grey literature pertaining to individuals explicitly called champions, who are tasked with performing knowledge translation within a health care context.

**Research Questions:** 1) What are the definitions and attributes used to describe champions? 2) What are the roles of champions? 3) What KT strategies do champions perform? 4) How can the KT performed by champions be mapped out according to the phases of the Knowledge to Action (KTA) framework (Graham et al., 2006)? 5) What are the barriers and facilitators affecting how champions perform their role or KT strategies? A systematic review of a subset of the articles will be performed to answer the research question: How effective are champions in translating knowledge within a health care context?

OVID Medline 10.6.2020 (1946 to October 01, 2020)

| **PCC (Population, Concept, Context)** | **Search terms** | **Results** |
| --- | --- | --- |
| Population: Champions | 1. (champion*).mp. | 7740 |
| Concept: Knowledge translation/evidence base practice | 2. (ebp or ebm or ebn or cpg* or best practice* or (evidence adj2 practice*)).mp.  3. (guideline* adj2 (implement* or adher*)).mp.  4. (quality adj1 (improv* or manag*)).mp.  5. (research adj2 (‘use’ or utili* or adopt* or implement* or disseminat* or uptake or transfer* or translat* or support)).mp.  6. (knowledge adj2 (‘use’ or utili* or adopt* or implement* or disseminat* or uptake or transfer* or translat* or support)).mp.  7. (evidence adj2 (‘use’ or utili* or adopt* or implement* or disseminat* or uptake or transfer* or translat* or support)).mp.  8. ((innovation adj2 (adopt* or diffus*)) or (implementation adj2 (science* or research)) or (innovation or diffusion)).mp.  9. exp Evidence-Based Practice/  10. guideline adherence/ or quality assurance, health care/ or quality improvement/ or Outcome Assessment, Health Care/ or Process Assessment, Health Care/ or "Outcome and Process Assessment, Health Care"/ or Safety Management/  11. "diffusion of innovation"/ or implementation science/ or technology transfer/ or Organizational Innovation/ | 102790  39460  103744  9249011  18699  53212  312984  88926 225160  43190 |
| Context: Healthcare | 12. (health adj2 care or healthcare).mp.  13. (((health care adj2 (professional* or personnel* or provider* or occupation*)) or (healthcare adj2 (professional* or personnel* or provider* or occupation*)) or nurse* or physician* or dentist* or allied) adj2 profession*).mp.  14. Patient care/ or "Delivery of Health Care"/ or Health Services Administration/ or Quality of Health Care/ or health resources/ or "health services needs and demand"/ or Health Services/ or Health Services Research/ or Hospitals/ or Ambulatory Care Facilities/  15. Health Personnel/ or Health Occupations/ or practice patterns, dentists'/ or practice patterns, nurses'/ or practice patterns, physicians'/ or professional practice gaps/ or "attitude of health personnel"/ | 1023682  276583  364884  301715 |
| Combinations | 16. OR/2-11  17. OR/12-15  18. 1 and 16 and 17 | 9799099  2081395  1376 |
| Sensitivity test | 19. Role of Champions in the Implementation of Patient Safety Practice Change.m_titl.  20. Correlates of the Sustainability of Community-Based Heart Health Promotion Interventions.m_titl.  21. (Adoption of Reminder and Recall Messages for Immunizations by Pediatricians and Public Health Clinics).m_titl.  22. Champions in context: which attributes matter for change efforts in healthcare?.m_titl.  23. Evidence-Based Practice Change Champion Program Improves Quality Care.m_titl.  24. The role of the organizational champion in achieving health system change.m_titl.  25. (Characteristics and behaviours of formal versus informal nurse champions and their relationship to innovation success).m_titl.  26. Inside help: An integrative review of champions in healthcare-related implementation.m_titl.  27. The Role of Nursing Best Practice Champions in Diffusing Practice Guidelines: A Mixed Methods Study.m_titl.  28. (What is the extent of research on the characteristics, behaviors, and impacts of health information technology champions? A scoping review).m_titl.  29. "What is the impact of research champions on integrating research in mental health clinical practice? A quasiexperimental study in South London, UK.".m_titl.  30. "Research Champions: An Initiative to Improve Use of Research Evidence in Nursing Practice.".m_titl.  31. or/18-29  32. 17 and 30 | 1  1  1  1  1  1  1  1  1  1  1  1  12  12 |

|  | Reviewer: Tamara | Email: tamara.rader@gmail.com | Date completed: 20 Oct 2020 | | |
| --- | --- | --- | --- | --- | --- |
|  |  |  |  | | |
|  | **1. TRANSLATION** |  |  | | |
|  | | A -­‐No revisions | | X |  |
|  | | B -­‐ Revision(s) suggested | | ☐ |  |
|  | | C -­‐ Revision(s) required | | ☐ |  |

**2. BOOLEAN AND PROXIMITY OPERATORS**

| A -­‐No revisions | X |
| --- | --- |
| B -­‐ Revision(s) suggested | ☐ |
| C -­‐ Revision(s) required | ☐ |

**3. SUBJECT HEADINGS**

| A -­‐No revisions | ☐ |
| --- | --- |
| B -­‐ Revision(s) suggested | X |
| C -­‐ Revision(s) required | ☐ |

**4. TEXT WORD SEARCHING**

| A -­‐No revisions | X |
| --- | --- |
| B -­‐ Revision(s)suggested | ☐ |
| C -­‐ Revision(s) required | ☐ |

**5. SPELLING, SYNTAX, AND LINE NUMBERS**

| A -­‐No revisions | X |
| --- | --- |
| B -­‐ Revision(s)suggested | ☐ |
| C -­‐ Revision(s) required | ☐ |

**6. LIMITS AND FILTERS**

| A -­‐No revisions | X |
| --- | --- |
| B -­‐ Revision(s) suggested | ☐ |
| C -­‐ Revision(s) required | ☐ |

OVERALL EVALUATION (Note: If one or more “revision required” is noted above, the response below must be “revisions required”.)

| A -­‐No revisions | ☐ |
| --- | --- |
| B -­‐ Revision(s) suggested | X |
| C -­‐ Revision(s) required | ☐ |

Comments:

I think the search approach is appropriate, and almost all the concepts are well developed using controlled vocabulary (MeSH) and text word searching

There is no direct MeSH heading relevant for “Champion” but I have suggested two MeSH headings which may cover the concept. It is recommended to have both MeSH and text words to describe each concept.

In reviewing the indexing for the known titles in the sensitivity set, I have identified a couple of additional MeSH terms to consider.

-Tamara
